# Global, regional, and national disease burden of arthropod-borne diseases: Projections to 2030 based on the global burden of disease study 2021

**DOI:** 10.1371/journal.pntd.0014235

**Published:** 2026-05-05

**Authors:** Mengqing Li, Yang Yang, Chuizhao Xue, Qingqiu Zuo, Ying Wang, Hua Liu, Yefei Pu, Yujuan Shen, Xu Wang, Jianhai Yin, Jianping Cao

**Affiliations:** 1 National Key Laboratory of Intelligent Tracking and Forecasting for Infectious Diseases, National Institute of Parasitic Diseases at Chinese Center for Disease Control and Prevention (Chinese Center for Tropical Diseases Research), Shanghai, China; 2 Key Laboratory of Parasite and Vector Biology, National Health Commission of the People’s Republic of China, Shanghai, China; 3 World Health Organization Collaborating Centre for Tropical Diseases, Shanghai, China; 4 The School of Global Health, Chinese Center for Tropical Diseases Research, Shanghai Jiao Tong University School of Medicine, Shanghai, China; Air Force Medical University, CHINA

## Abstract

**Background:**

Arthropod-borne diseases (ABDs) represent an ongoing threat to global public health, affecting millions annually with viral, bacterial and parasitic infections worldwide.

**Methods:**

Data on prevalence and disability-adjusted life years (DALYs) for ABDs were obtained from the Global Burden of Disease (GBD) 2021 study. Based on DALYs, the nine ABDs were categorized into protozoan, helminthic, and viral diseases. Temporal trends were quantified using the average annual percentage change (AAPC) in age-standardized prevalence rates (ASPRs) and age-standardized DALY rates (ASDRs). Frontier analysis was applied to evaluate deviations from the expected disease burden according to sociodemographic index (SDI), and Bayesian age–period–cohort models were used to project future disease burdens through 2030.

**Results:**

From 1990 to 2021, the global ASDR for ABDs declined from 1,219.26 to 884.16 per 100,000 population. Protozoiasis caused the most substantial burden (ASDR 819.83), followed by helminthiases and viral diseases. Although ASDRs declined across the three major disease categories overall, dengue fever exhibited a significant upward trend within the viral disease group (AAPC = 0.83%). Burdens remained concentrated in low and lower-middle SDI regions, though High-income Asia Pacific and Australasia experienced notable increases. Frontier analysis indicated that while parasitic burdens declined with rising SDI, viral disease patterns varied: dengue peaked at middle SDI, and Zika increased with SDI. Adolescents and young adults bore the greatest burden, with distinct sex-specific differences. Projections suggest that by 2030, ASPRs for malaria and African trypanosomiasis, as well as ASDRs for malaria, African trypanosomiasis, and leishmaniasis, will increase.

**Conclusions:**

Protozoan infections, particularly malaria, continue to dominate the burden of arthropod-borne diseases. Although the overall burden of viral diseases declined modestly, certain arboviral infections, including dengue, showed increasing trends. The distinct geographic concentration of these infections, coupled with the rising threat of arboviral diseases, underscores the urgent need for enhanced surveillance systems, expanded vaccination coverage, and strengthened global collaboration to mitigate future risks.

## Author summary

Arthropod-borne diseases, infections transmitted by vectors such as mosquitoes, ticks, and flies, remain a critical challenge to global public health, particularly in resource-limited settings. In this study, we utilized data from the Global Burden of Disease (GBD) Study 2021 to assess the evolving landscape of these diseases over the past three decades and project their trajectories through 2030. We found that while the overall health burden has declined since 1990, driven largely by successful interventions against parasitic infections like malaria, new challenges are emerging. Viral diseases, especially dengue, are increasing globally, expanding even into regions with higher socioeconomic development. Our analysis reveals that adolescents and young adults bear the heaviest disease burden, with distinct patterns observed between males and females. Most concerningly, our projections indicate that the historical decline in major parasitic diseases may stall or reverse, with potential increases in malaria and African trypanosomiasis predicted by 2030. These findings underscore the urgency of strengthening surveillance systems, advancing vaccine development, and sustaining international cooperation to safeguard future generations against these persistent and evolving threats.

## Introduction

Arthropods, the largest phylum of animals on Earth, comprise over 1.2 million described species, accounting for approximately 80% of all known animal species [1]. Major arthropod vectors of disease, such as mosquitoes, ticks, black flies, fleas, lice, triatomine bugs, tsetse flies, and Culicoides midges, can transmit diverse pathogens, including viruses, bacteria, protozoa, and helminths [2]. Arthropod-borne viral diseases such as dengue fever [3], chikungunya fever [4], and tick-borne encephalitis [5] cause billions of infections and hundreds of thousands of deaths annually [1]. For example, recently, an outbreak of chikungunya fever in Foshan City, Guangdong Province, China, resulted in 4,824 confirmed cases reported across 12 cities as of July 26^th^ 2025 [6]. Arthropod-borne bacterial diseases include Lyme disease, plague, and typhus. Lyme disease is predominantly endemic in the United States and Europe and is one of the most common vector-borne diseases in the United States, with approximately 476,000 reported cases each year [7]. Referring to protozoiasis, malaria [8], Chagas disease [9], and leishmaniasis [10] are also transmitted by arthropods. According to the World Health Organization (WHO) World Malaria Report 2024, about 263 million malaria cases and 597,000 deaths were reported in 83 endemic countries in 2023 [11]. In addition, helminthic diseases such as lymphatic filariasis and onchocerciasis [12] are also transmitted by arthropods. An estimated one billion people in 72 countries or territories remain at risk of lymphatic filariasis. Since the launch of the WHO Global Programme to Eliminate Lymphatic Filariasis (GPELF), at least 36 million people have developed related complications [13].

Arthropod-borne diseases (ABDs) are primarily concentrated in economically disadvantaged regions of Africa, Asia, and Latin America [14], where poor health conditions and inadequate medical facilities contribute to high morbidity and mortality rates [15]. Beyond their high baseline burden, some ABDs are demonstrating upward trends. Globalization, climate change [16], land use changes [17], and the expansion of global transportation networks have facilitated the geographical spread of arthropod vectors, thereby increasing the risk of outbreaks of ABDs [18]. For example, the proliferation of midges and mosquitoes has contributed to outbreaks of Oropouche fever in Latin America [19]. Moreover, chikungunya fever, transmitted by *Aedes albopictus* and *Aedes aegypti*, has caused outbreaks in Guangdong, China [6].

The diverse and expanding burden of ABDs, compounded by resource inequities and increasing antimicrobial resistance, constitutes a major global health threat. Given these threats, a comprehensive assessment of their burden is urgently required, addressing global and regional patterns, population-specific impacts, temporal trends, and socioeconomic consequences. Based on data from the Global Burden of Disease Study (GBD) 2021, the present study systematically analyzes the prevalence and disability-adjusted life years (DALYs) of ABDs from 1990 to 2021. By reviewing historical data and projecting future burdens, we aim to provide a comprehensive and comparable epidemiological evaluation to inform targeted global prevention and control strategies.

## Materials and methods

### Ethics statement

This article does not contain any studies with human participants or animals performed by any of the authors. The data used in this research are publicly available and de-identified, retrieved from the Global Burden of Disease (GBD) study database (https://vizhub.healthdata.org/gbd-results/). Therefore, ethical approval and informed consent were not required.

### Data source

Based on the latest epidemiological data and improved standardized methodologies, the Global Burden of Disease Study 2021 (GBD 2021) systematically estimated the global burden of 371 diseases and injuries, 288 causes of death, and 88 risk factors across 204 countries and territories from 1990 to 2021 [20]. Herein, we obtained data on the burden of ABDs from the Global Burden of Disease (GBD) study database (https://vizhub.healthdata.org/gbd-results/). platform for the period 1990–2021. The dataset includes GBD Estimate (cases of death or injury), measures (prevalence, DALYs), metrics (rate, number), causes (malaria, Chagas disease, leishmaniasis, African trypanosomiasis, lymphatic filariasis, onchocerciasis, dengue, yellow fever, and Zika virus disease), location (global, Sociodemographic Index (SDI) regions, GBD sub-regions, and 204 countries and territories), age (all ages, age-standardized, and 5-year age groups from < 5 years to 95 + years at a 5-year interval), sex (both sexes, female, male), and year (1990–2021).

The SDI, ranging from 0 to 1, is a composite indicator used to assess a country’s level of socio-economic development in relation to health. Based on national SDI values, GBD categorizes the 204 countries and territories into five quintiles (high, high-middle, middle, low-middle, and low).

Age categories were defined according to the World Health Organization (WHO) standard classification. “Adolescents” were defined as individuals aged 10–19 years, and “young people” as those aged 10–24 years [21].

The selection of diseases was based on the International Classification of Diseases (ICD), tenth revision (ICD-10), and ICD-9. This study encompassed nine biologically transmitted diseases that are spread via arthropod bites and were modeled independently in the GBD 2021 study. Diseases were included if they met the following criteria: (1) biological transmission through arthropod vectors via bites; (2) independent cause-specific estimates in the GBD 2021 framework; and (3) sufficient data availability for age- and sex-specific burden estimation. Diseases transmitted solely through mechanical carriage of pathogens on arthropod surfaces were excluded. These diseases were further categorized according to their pathogenic agents into protozoiasis (malaria, Chagas disease, leishmaniasis, African trypanosomiasis), helminthiases (lymphatic filariasis, onchocerciasis), and viral diseases (dengue, yellow fever, and Zika virus disease). Given the presence of comorbidities, we could not combine prevalence estimates. Therefore, this study only aggregated DALYs, as expressed in the following formula:


$$\hat{p_{\text{total}}}=\sum_{k=1}^{i}\hat{p_{k}},\;SE=\frac{U_{k}-L_{k}}{2\times z_{0.975}},\;SE_{\text{total}}=\sqrt{\sum_{k=1}^{i}S\overset{2}{\underset{k}{E}}}$$

$$95\%\;CI=\hat{p_{\text{total}}}\pm 1.96\times SE_{\text{total}}$$

where,${\;p}_{k}$ represents the point estimate of the prevalence of the *k*-th disease;$\text{ and }{[L}_{k},\;U_{k}]\;$denotes its 95% confidence interval (CI); $z_{0.975}\approx 1.96$.

### Statistical analysis

To characterize the burden of ABDs from 1990 to 2021, we conducted a longitudinal analysis of temporal trends at the global, regional, and national levels, paying particular attention to changes in health inequalities and future projections. In addition, a cross-sectional comparison was performed to assess the differential burden across diseases, regions, sexes, and age groups.

This study used the number of prevalent cases, DALYs, and their corresponding age-standardized rates (ASRs) as the primary metrics to assess the burden of ABDs, all of which are reported with 95% uncertainty intervals (UIs).

We utilized Joinpoint regression analysis to evaluate temporal trends in the burden of ABDs from 1990 to 2021 by estimating the Average Annual Percentage Change (AAPC) in age-standardized prevalence (ASPR) and DALY rates (ASDR). A log-linear segmented regression model was applied, using the natural logarithm of the ASR as the dependent variable to identify significant shifts in trends (joinpoints). The optimal number and timing of these joinpoints were determined using the Grid Search Method (GSM) based on the minimum mean squared error. We validated the joinpoints using a Monte Carlo permutation test, allowing for a maximum of five points. Finally, the AAPC and its 95% confidence interval (CI) were calculated to quantify the average annual change over the study period [22].

Formula for Average Annual Percentage Change (AAPC):


$$\text{AAPC}=\left(e^{\frac{\sum w\text{i}}{\sum wi\cdot \beta i}}-1\right)\times 100\%$$

where, βi represents the regression slope for the i time segment, and wi is the length (in years) of the corresponding time segment.

### Frontier analysis

To evaluate the relationship between the burden of ABDs and SDI, we constructed a frontier model based on the ASDR. The model considers the theoretically lowest ASDR achievable at the current SDI level for each region as the optimal performance benchmark. Data Envelopment Analysis (DEA) was performed using the Free Disposal Hull (FDH) method to build a nonlinear frontier, while excluding super-efficient countries to reduce the influence of outliers. The absolute gap between the ASDR of each country or region in 2021 and the frontier line was measured to assess the potential room for improvement under the current development level [23].

### Forecasting analysis

We used a Bayesian age-period-cohort (BAPC) model to project the global burden of each arthropod-borne disease up to 2030 [23]. The model, based on a Bayesian generalized linear framework, accounts for age, period, and cohort effects, and is well-suited for high-dimensional and sparse data typical of GBD studies. The model was implemented using Integrated Nested Laplace Approximation (INLA), with age, period, and cohort effects specified as second-order random walks to ensure smooth temporal trends. Weakly informative Gaussian priors were assigned to model hyperparameters. Model performance was evaluated using posterior summaries and the deviance information criterion (DIC), and model stability was assessed by examining posterior marginal distributions. Uncertainty from the GBD estimates was propagated within the Bayesian framework, and projections are presented with 95% credible intervals (CrIs). The population estimates were obtained from the GBD database.

The AAPC was calculated using the Joinpoint Regression Program (version 5.1.0.0, National Cancer Institute, Rockville, MD, USA). Other analyses were conducted in R (version 4.5.0) and Microsoft Excel 2019 (Redmond, WA, USA). *P* < 0.05 was considered statistically significant. This study used publicly available secondary data and did not require ethical approval.

## Results

### Global burden and trends

The global DALYs of ABDs reached 71,749,012.92 (95% UI 29,800,709.35–113,697,316.49) in 1990 and 61,227,826.19 (95% UI 17,912,189.53–104,543,462.85) in 2021, with corresponding ASDRs of 1,219.26 per 100,000 population (95% UI 512.21–1,926.32) and 884.16 per 100,000 population (95% UI 258.21–1,510.10) (Table 1). Among the three categories, protozoiasis contributed 64,057,769.37 DALYs (95% UI 22,150,317.59–105,965,221.14) in 1990 and 56,255,800.08 DALYs (95% UI 12,963,474.53–99,548,125.63) in 2021, with ASDRs of 1,074.79 per 100,000 population (95% UI 368.60–1,780.99) and 819.83 per 100,000 population (95% UI 194.16–1,445.51), respectively. In particular, malaria accounted for the majority of this burden, with DALYs of 57,888,052.59 (95% UI 30,256,058.77–113,079,353.06) in 1990 and 55,174,060.70 (95% UI 21,761,299.60–108,337,905.75) in 2021, and ASDRs of 965.67 per 100,000 population (95% UI 502.56–1,898.07) and 806.00 per 100,000 population (95% UI 318.93–1,570.18), respectively. Second, helminthiases accounted for 5,454,609.47 DALYs (95% UI 3,849,056.99–7,060,161.95) in 1990 and 2,577,551.52 DALYs (95% UI 1,648,634.65–3,506,468.40) in 2021, with ASDRs of 105.77 per 100,000 population (95% UI 74.82–136.72) and 32.27 per 100,000 population (95% UI 20.59–43.95), respectively. Third, viral diseases contributed 2,236,634.08 DALYs (95% UI 1,315,841.30–3,157,426.87) in 1990 and 2,394,474.59 DALYs (95% UI 1,319,281.11–3,469,668.07) in 2021, with ASDRs of 38.70 per 100,000 population (95% UI 22.73–54.66) and 32.05 per 100,000 population (95% UI 17.80–46.29), respectively (Table A in S1 Text). Given the high contribution of malaria, we also calculated the burden of ABDs excluding malaria (Table B in S1 Text). The global DALYs were 13,860,960.33 (95% UI 7,172,488.68–20,549,431.99) in 1990 and 6,053,765.49 (95% UI 4,515,196.00–7,592,334.98) in 2021, with ASDRs of 253.59 per 100,000 population (95% UI 139.28–367.91) and 78.16 per 100,000 population (95% UI 58.06–98.26), respectively. Overall, both the DALYs and ASDR of ABDs declined between 1990 and 2021, with a 14.66% reduction in DALYs and an AAPC of –0.97% (95% CI –1.39% to –0.56%, *P* < 0.05) for the ASDR. In contrast, ABDs excluding malaria showed a markedly greater reduction, with DALYs decreasing by 56.3% and an AAPC of –3.74% (95% CI –4.15% to –3.33%, *P* < 0.05) (Table B in S1 Text). Protozoiasis decreased slightly (–12.18%, AAPC –0.81%, 95% CI –1.25% to –0.36%, *P* < 0.05), helminthiases declined markedly (–52.73%, AAPC –3.73%, 95% CI –3.93% to –3.53%, *P* < 0.05), while viral diseases showed increasing DALYs (6.59%) but a decreasing ASDR (AAPC –0.63%, 95% CI –1.23% to –0.03%, *P* < 0.05) (Table 1).

**Table 1:** DALYs of arthropod-borne diseases in 1990 and 2021, and AAPCs from 1990 to 2021.

Location	1990 ASDRs (1/100, 000, 95% UI)	2021 ASDRs (1/100, 000, 95% UI)	AAPCs (95% CI)	*P* Value
Global	1219.26 (512.21, 1926.32)	884.16 (258.21, 1510.10)	−0.97 (−1.39, −0.56)	< 0.05
High SDI	2.67 (0.29, 5.05)	1.72 (0.96, 2.49)	−1.13 (−2.46, 0.23)	0.10
High-middle SDI	30.47 (3.78, 57.16)	16.91 (11.81, 22.02)	−2.05 (−2.76, −1.33)	< 0.05
Middle SDI	444.42 (163.81, 725.03)	255.62 (121.17, 390.07)	−1.81 (−2.36, −1.27)	< 0.05
Low-middle SDI	1362.07 (425.73, 2298.40)	735.61 (222.25, 1248.96)	−1.88 (−2.26, −1.50)	< 0.05
Low SDI	6158.08 (3098.97, 9217.20)	3080.09 (791.09, 5369.08)	−2.13 (−2.37, −1.89)	< 0.05
East Asia	10.55 (0.00, 41.15)	0.93 (0.00, 3.09)	−7.77 (−8.69, −6.83)	< 0.05
Southeast Asia	537.49 (191.93, 883.05)	191.03 (130.98, 251.08)	−3.20 (−3.92, −2.48)	< 0.05
Oceania	3245.18 (0.00, 6973.16)	1217.03 (486.03, 1948.03)	−3.41 (−6.24, −0.48)	< 0.05
Central Asia	46.76 (4.16, 89.36)	9.43 (0.00, 21.21)	−5.89 (−8.51, −3.19)	< 0.05
Central Europe	8.47 (0.00, 48.72)	0.43 (0.00, 2.31)	−9.09 (−10.18, −7.98)	< 0.05
Eastern Europe	0.00 (0.00, 0.00)	0.00 (0.00, 0.00)	NA	NA
High-income Asia Pacific	2.59 (0.88, 4.29)	3.05 (0.00, 6.48)	0.52 (0.02, 1.02)	< 0.05
Australasia	0.41 (0.00, 0.90)	0.66 (0.00, 1.38)	1.52 (0.76, 2.28)	< 0.05
Western Europe	1.73 (0.00, 6.94)	0.56 (0.00, 1.84)	−3.45 (−4.29, −2.61)	< 0.05
Southern Latin America	106.75 (76.68, 136.82)	28.74 (20.38, 37.09)	−4.13 (−4.27, −3.98)	< 0.05
High-income North America	0.62 (0.37, 0.87)	0.54 (0.32, 0.77)	−0.38 (−0.58, −0.18)	< 0.05
Caribbean	267.06 (116.65, 417.47)	134.35 (0.00, 288.13)	−4.65 (−9.66, 0.63)	0.08
Andean Latin America	447.06 (142.23, 751.90)	78.86 (60.43, 97.29)	−5.48 (−7.68, −3.24)	< 0.05
Tropical Latin America	574.76 (399.62, 749.89)	182.49 (96.04, 268.94)	−3.72 (−4.44, −3.00)	< 0.05
North Africa and Middle East	342.75 (26.95, 658.55)	178.44 (58.36, 298.52)	−2.18 (−2.99, −1.37)	< 0.05
South Asia	1002.08 (70.81, 1933.35)	203.07 (28.47, 377.67)	−5.34 (−6.31, −4.37)	< 0.05
Central Sub-Saharan Africa	11,352.97 (7014.40, 15691.55)	4808.84 (1906.21, 7711.48)	−2.79 (−3.23, −2.34)	< 0.05
Eastern Sub-Saharan Africa	6513.20 (3672.28, 9354.13)	2336.02 (600.10, 4071.93)	−3.19 (−3.73, −2.64)	< 0.05
Southern Sub-Saharan Africa	382.33 (38.75, 725.91)	184.68 (35.07, 334.28)	−1.30 (−9.15, 7.22)	0.76
Western Sub-Saharan Africa	9319.66 (4354.38, 14284.93)	5857.55 (1401.48, 10313.61)	−1.42 (−1.68, −1.16)	< 0.05

DALYs: disability-adjusted life-years, ASDRs: age-standardized DALYs rates, AAPCs: average annual percentage changes, UI: uncertainty intervals, CI: confidence interval, SDI: Socio-demographic Index.

At the disease-specific level, malaria imposed the highest burden, and Zika virus disease the lowest. In 1990, lymphatic filariasis accounted for the largest share of prevalence cases (51.76%), whereas malaria contributed the highest proportion of DALYs (80.68%). By 2021, malaria dominated both prevalence and DALYs, representing 66.05% and 89.61%, respectively. Dengue showed rising trends in both ASPR and ASDR, with AAPCs of 1.45% (95% CI 1.30%–1.60%, *P* < 0.05) and 0.83% (95% CI 0.48%–1.19%, *P* < 0.05), while leishmaniasis increased in ASPR but declined in ASDR, with AAPCs of 0.64% (95% CI 0.59%–0.68%, *P* < 0.05) and –6.65% (95% CI 7–.68% to –5.62%, *P* < 0.05) (Tables C and D and Figs A and B in S1 Text).

### Regional burden and trends

**Fig 1:**
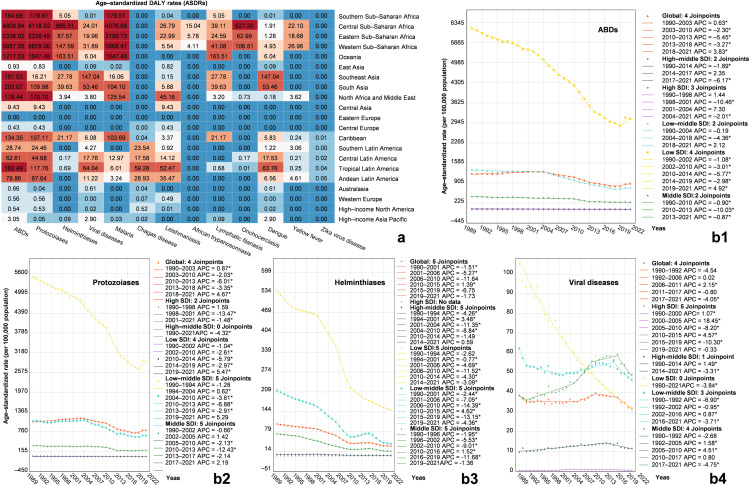
Temporal trends and geographical variations in the global burden of arthropod-borne diseases (1990–2021). **(a)** Ranking of ASDRs for arthropod-borne diseases by 21 Regions. ASDRs, age-standardized DALY rates **(b)** Global and five SDI regions’ changes ASDR for arthropod-borne diseases from 1990 − 2021. The panels represent: (b1) total arthropod-borne diseases, (b2) protozoiasis, (b3) helminthiases, and (b4) viral diseases. Abbreviations: ASDR, age-standardized DALY rate; APC, annual percentage change; AAPC, average annual percentage change; SDI, Socio-demographic Index. *P < 0.05.

The overall burden of ABDs was highest in low-SDI regions, with an ASDR of 3,080.09 per 100,000 population (95% UI 791.09–5,369.08) in 2021 (Table 1). Among the 21 subregions, the highest ASDR was observed in Western Sub-Saharan Africa at 5,857.55 per 100,000 population (95% UI 1,401.48–10,313.61) (Fig 1a), (Table 1). Both protozoal and helminth infections were most burdensome in low-SDI regions, with ASDRs of 2,898.47 per 100,000 population (95% UI 610.17–5,186.76) and 150.97 per 100,000 population (95% UI 97.31–204.63), respectively. At the subregional level, the highest burdens were recorded in Western Sub-Saharan Africa for protozoiasis (5,678.06 per 100,000 population, 95% UI 1,222.41–10,133.71) and in Central Sub-Saharan Africa for helminthiases (666.31 per 100,000 population, 95% UI 406.57–926.05) (Fig 1a). For viral diseases, the highest ASDR was found in middle-SDI regions (49.10 per 100,000 population, 95% UI 27.26–70.95), with the greatest subregional burden in Southeast Asia (147.04 per 100,000 population, 95% UI 94.22–199.86) (Fig 1a).

In terms of temporal trends, the burden of ABDs and each of the three categories declined significantly in low-SDI regions, with helminthiases showing the largest reduction (AAPC –4.06%, 95% CI –4.19% to –3.93%, *P* < 0.05) (Fig 1b3), (Table 1). The overall burden of ABDs increased only in High-income Asia Pacific and Australasia, with AAPCs of 0.52% (95% CI 0.02%–1.02%, *P* < 0.05) and 1.52% (95% CI 0.76%–2.28%, *P* < 0.05), respectively (Fig 1b1), (Table 1). In low-middle SDI and low SDI regions, the ASDR for ABDs excluding malaria declined more markedly (AAPC –4.03%, 95% CI –4.75% to –3.31%; and –5.64%, 95% CI –6.05% to –5.23%, respectively) than the ASDR for overall ABDs (AAPC –1.88%, 95% CI –2.26% to –1.50%; and –2.13%, 95% CI –2.37% to –1.89%, respectively). A similar pattern was observed in Central Sub-Saharan Africa (AAPC –4.58%, 95% CI –5.19% to –3.97%), Eastern Sub-Saharan Africa (AAPC –7.35%, 95% CI –7.99% to –6.72%), and Western Sub-Saharan Africa (AAPC –5.47%, 95% CI –6.21% to –4.73%) (Table B in S1 Text). In comparison, the corresponding declines in overall ABD burden in these regions were more modest (AAPC –2.79%, 95% CI –3.23% to –2.34%; –3.19%, 95% CI –3.73% to –2.64%; and –1.42%, 95% CI –1.68% to –1.16%, respectively) (Table 1). In these regions, helminthiases in High-income Asia Pacific and viral diseases in both regions also showed significant upward trends (AAPC > 0, *P* < 0.05) (Fig 1b3), (Table 1). Additionally, viral diseases increased significantly in High-income North America, Central Latin America, Tropical Latin America, and South Asia (AAPC > 0, *P* < 0.05) (Fig 1b4), (Table 1). Further trend analysis revealed that the overall burden of ABDs and the burden of protozoiasis showed an upward trend from 2018 to 2021, particularly in low-SDI regions. The APC was 4.92% (95% CI 2.01%–7.91%, *P* < 0.05) for the overall burden and 5.47% (95% CI 1.88%–9.19%, *P* < 0.05) for protozoiasis (Fig 1b1 and 1b2). Helminthiases exhibited an increasing trend between 2010 and 2015 at the global level and in low-middle SDI regions, with APCs of 1.39% (95% CI 0.86%–1.92%, *P* < 0.05) and 4.62% (95% CI 3.96%–5.28%, *P* < 0.05), respectively (Fig 1b3). Viral diseases began to decline significantly around 2017 in both global and all *SDI* regions (AAPC < 0, *P* < 0.05) (Fig 1b4).

Among the nine ABDs, the ASDR burden was concentrated in low-SDI regions for six diseases. Exceptions include Chagas disease, which had the highest ASDR in low-middle SDI regions (5.76 per 100,000 population, 95% UI 4.87–6.79), and dengue (48.78 per 100,000 population, 95% UI 27.32–71.02) and Zika virus disease (0.01 per 100,000 population, 95% UI 0.00–0.01), which peaked in middle-SDI regions. In terms of trends in DALYs, from 2019 to 2021, malaria in low-SDI regions showed a substantial increase (AAPC 5.48%, 95% CI: 1.98%–9.09%, *P* < 0.05). The burden of Chagas disease rose significantly in High-income Asia Pacific and Western Europe (AAPC > 2%, *P* < 0.05), while leishmaniasis increased in Western Sub-Saharan Africa (AAPC 2.08%, 95% CI: 1.86%–2.31%, *P* < 0.05). Dengue DALYs continued to rise in most regions, but Western Europe experienced a marked decline (AAPC –13.30%, 95% CI: –18.90% to –7.32%, *P* < 0.05). Detailed prevalence data are provided in the supplementary materials. (Table D and Fig B in S1 Text).

### National burden and trends

**Fig 2:**
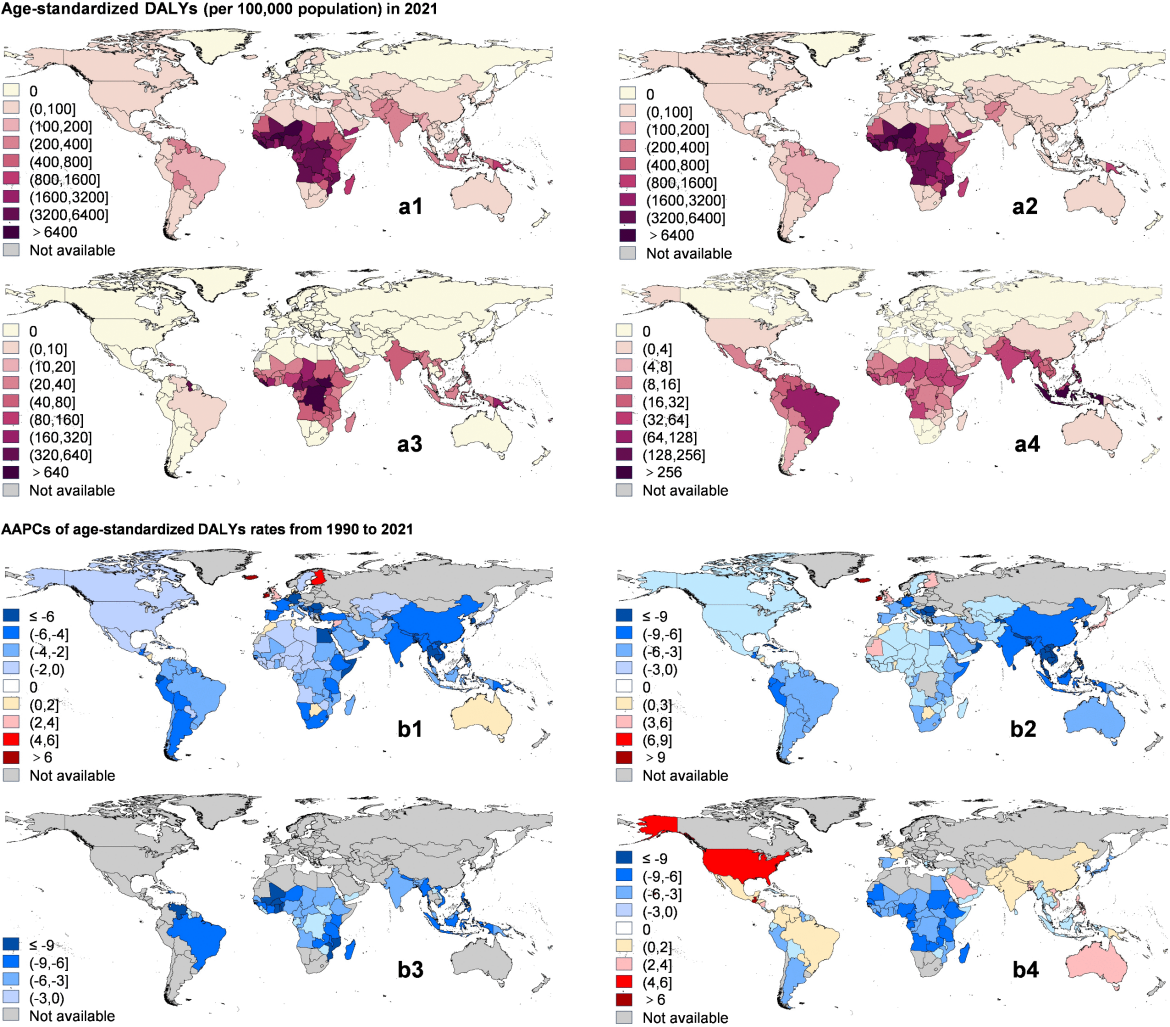
Global map of arthropod-borne diseases at both country and territorial levels. **(a)** The ASDRs for NTDs among different countries. **(b)** The ASDR AAPCs for among different countries. The panels correspond to: (a1, b1) total arthropod-borne diseases; (a2, b2) protozoiasis; (a3, b3) helminthiases; and (a4, b4) viral diseases. Abbreviations: ASDR, age-standardized DALY rate; AAPC, average annual percentage change. Country boundary data were obtained from the Global Administrative Areas (GADM) database (version 4.1; https://gadm.org/download_country.html; license information: https://gadm.org/license.html). Maps were generated using ArcGIS software.

The overall DALY burden of ABDs was highest in the Republic of Sierra Leone, at 9,469.11 per 100,000 population (95% UI 2,299.46–16,638.75) (Table G in S1 Text and Fig 2a1). The fastest increases were observed in Ireland, Iceland, and Monaco (all high-SDI countries), with AAPCs of 14.33% (95% CI 13.69%–14.97%, *P* < 0.05), 11.20% (95% CI 10.59%–11.82%, *P* < 0.05), and 9.38% (95% CI 7.35%–11.45%, *P* < 0.05), respectively (Fig 2b1). For protozoiasis, Burkina Faso had the highest ASDR burden (8,958.65 per 100,000 population; 95% UI 3,166.64–14,750.66), while Ireland showed the fastest increase (AAPC 14.33%, 95% CI 13.69%–14.97%, *P* < 0.05) (Fig 2a2 and 2b2). Among helminth diseases, the highest burden was observed in the Republic of Liberia (1,561.40 per 100,000 population; 95% UI 904.47–2,218.32), although all countries exhibited declining trends (Fig 2a3 and 2b3). For viral diseases, the Republic of Indonesia had the highest burden (279.79 per 100,000 population; 95% UI 163.04–396.54), and Nauru showed the fastest increase (AAPC 7.84%, 95% CI 6.58%–9.12%, *P* < 0.05) (Fig 2a4 and 2b4). Finally, for non-malaria diseases, the Republic of South Sudan had the highest burden (1,937.46 per 100,000 population; 95% UI 1,297.65–2,577.27), with Ireland showing the fastest rise in ASDR (AAPC 14.33%, 95% CI 13.69%–14.97%, *P* < 0.05) (Table G in S1 Text).

Among specific ABDs, malaria showed remarkable geographic heterogeneity. The Republic of Liberia had the highest ASPR (29248.89 per 100,000 population; 95% UI 13845.02–47808.36), while the Republic of Sierra Leone had the highest age-standardized DALY rate (ASDR 8940.31 per 100,000 population; 95% UI 3029.75–17358.47). The Republic of Djibouti showed the fastest ASPR increase (AAPC 5.20%, 95% CI 0.51%–10.10%, *P* < 0.05). The Republic of Nicaragua had the largest increase in ASDR (AAPC 3.38%, 95% CI –3.15%–10.36%), although this was not statistically significant (*P* > 0.05). Nearly all positive AAPCs for malaria DALYs were not significant. Chagas disease was highly concentrated in the Plurinational State of Bolivia (ASPR 4132.52 per 100,000 population; 95% UI 3749.15–4522.73; ASDR 137.04 per 100,000 population; 95% UI 93.21–203.59). Ireland showed a marked increase, with AAPCs exceeding 14% for both indicators (*P* < 0.05). For leishmaniasis, the Syrian Arab Republic had the highest ASPR (4734.43 per 100,000 population; 95% UI 4143.02–5554.19), while the Republic of South Sudan had the highest ASDR (443.04 per 100,000 population; 95% UI 230.74–725.39). The Kingdom of Morocco showed the fastest increase (ASPR AAPC 4.27%, 95% CI 3.95–4.58, *P* < 0.05; ASDR AAPC 1.87%, 95% CI 1.56–2.19, *P* < 0.05). The African trypanosomiasis burden was highest in the Gabonese Republic (ASPR 10.68 per 100,000 population; 95% UI 3.96–22.13; ASDR 204.90 per 100,000 population; 95% UI 76.47–437.80). No country showed increasing ASPR, but the Republic of Zambia had a notable ASDR rise (AAPC 14.3%, 95% CI –40.43–119.30, *P* > 0.05). Lymphatic filariasis had the highest burden in the Republic of Guyana (ASPR 16158.49 per 100,000 population; 95% UI 5921.82–33023.58; ASDR 322.77 per 100,000 population; 95% UI 219.64–443.01). Onchocerciasis burden was highest in the Republic of South Sudan (ASPR 22097.74 per 100,000 population; 95% UI 19937.61–24463.65; ASDR 1387.88 per 100,000 population; 95% UI 873.08–2043.42). Both diseases showed overall declines, with notable reductions in the Republic of Malawi and Côte d’Ivoire (AAPCs below -10%, *P* < 0.05). For dengue, the Republic of Indonesia had the highest ASDR (279.79 per 100,000 population; 95% UI 170.92–404.42), while its ASPR was relatively low (53.64 per 100,000 population; 95% UI 20.99-114.25). The Republic of Nauru showed the fastest increase (ASPR AAPC 12.13%, 95% CI 6.78–17.74; ASDR AAPC 7.84%, 95% CI 6.58–9.12, both *P* < 0.05). For yellow fever, the Republic of Burundi showed a low ASPR (0.79 per 100,000 population; 95% UI 0.21–2.12) but a high ASDR (93.38 per 100,000 population; 95% UI 19.30–282.51). The disease showed a declining trend in all countries. For Zika virus disease, the Republic of El Salvador had the highest burden (ASPR 2.68 per 100,000 population; 95% UI 0.42–9.78; ASDR 0.16 per 100,000 population; 95% UI 0.08–0.36) (Tables E, F, H, and J in S1 Text).

### Age and sex patterns

**Fig 3:**
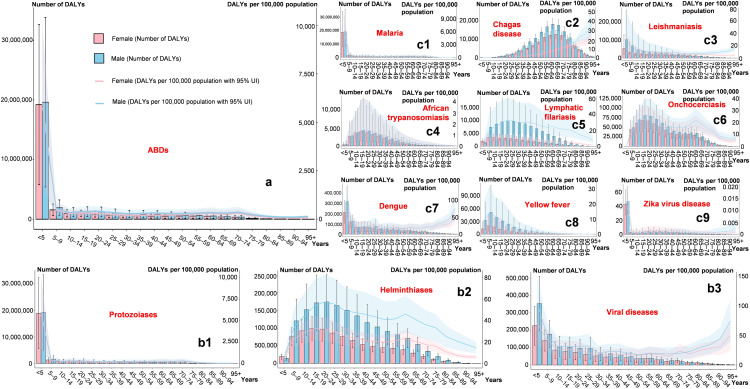
Number of DALYs and ASDRs of arthropod-borne diseases across different age groups globally for both sexes in 2021. The legend in panel (a) applies to all subsequent panels. The panels correspond to: (b1–b3) major disease categories (protozoiasis, helminthiases, and viral diseases); and (c1–c9) specific diseases (malaria, Chagas disease, leishmaniasis, African trypanosomiasis, lymphatic filariasis, onchocerciasis, dengue, yellow fever, and Zika virus disease). Abbreviations: DALYs, disability-adjusted life years; ASDR, age-standardized DALY rate.

For ABDs, the overall trend was consistent with that of protozoiasis, with the DALYs burden mainly concentrated among children aged < 5 years, accounting for 63.37% and 65.41% of DALYs number, respectively. After age 5, the disease burden decreases sharply; however, the gender burden ratio is generally similar (Fig 3a and 3b1). The burden of helminthic diseases increases gradually before age 24 and decreases gradually thereafter; sex differences are evident: except for the < 5 and ≥ 95 age groups, males bear a significantly higher burden than females, peaking at 40–44 years (DALYs number 214.38%) (Fig 3b2). For viral diseases, the DALYs burden decreases with age, but the trends of DALYs number and DALYs rate differ: the DALYs rate increases again after age 65. The male-to-female ratio is highest in the < 5 age group (156.66%) and then decreases with age (Fig 3b3). Among the nine specific diseases, malaria, leishmaniasis, dengue, yellow fever, and Zika have the number of DALYs concentrated in younger children, while the DALYs rate of leishmaniasis, dengue, and Zika increases gradually after age ≥ 80; the remaining diseases are mainly concentrated in adolescents and middle-aged populations, with Chagas disease showing an increase in the DALYs rate after age ≥ 80. For lymphatic filariasis, the male burden is significantly higher than that of females, with the number of DALYs exceeding 200% across the 5–94 age range, peaking at 60–64 years (387.36%) (Fig 3c1-c9).

### Frontier analysis

**Fig 4:**
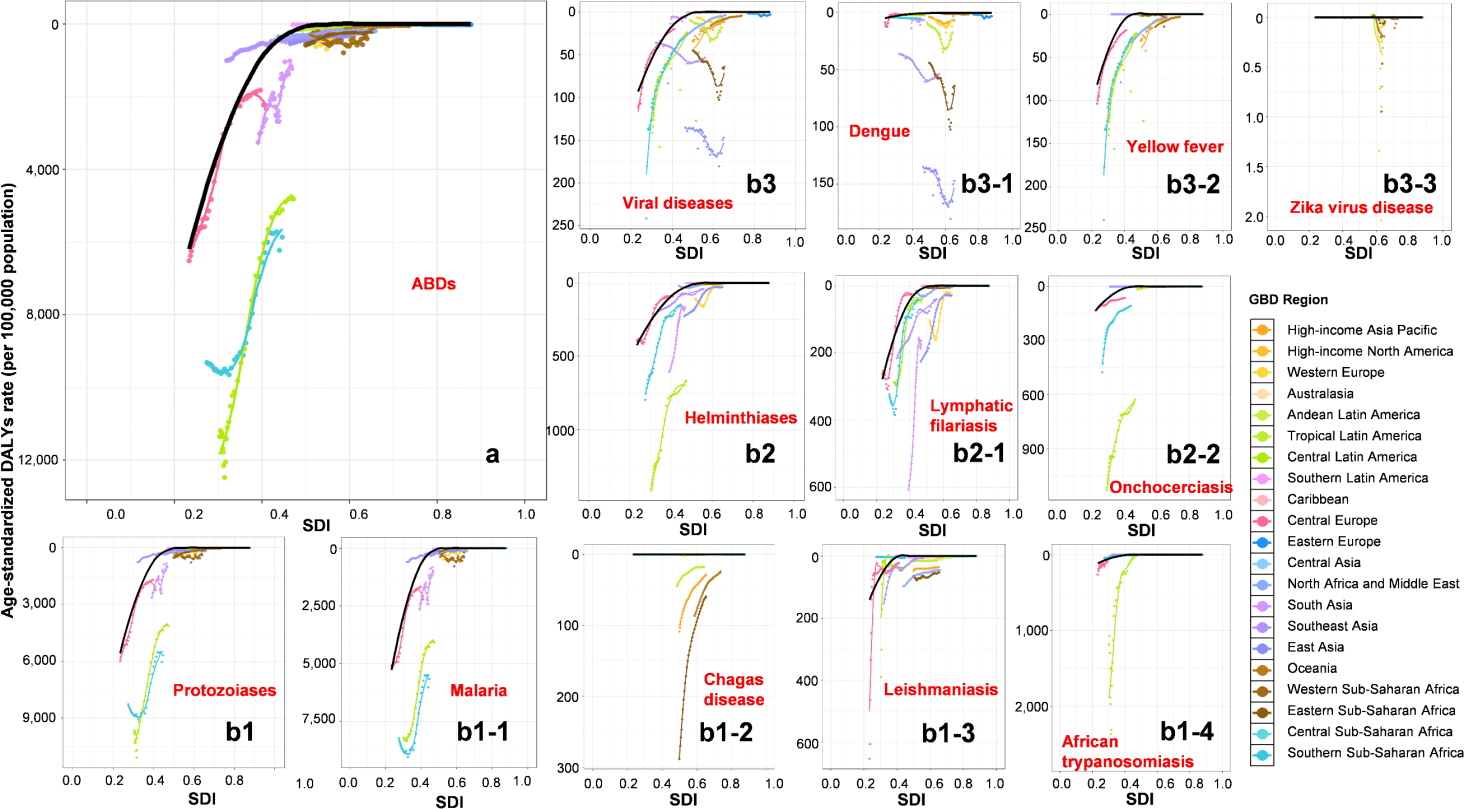
Frontier Analysis of ASDRs of Arthropod-Borne Diseases Across 21 Regions in Relation to SDI. **(a)** Overall arthropod-borne diseases. **(b)** Specific disease categories and conditions: (b1–b3) major categories (protozoiasis, helminthiases, and viral diseases). Specific diseases are displayed as: (b1-1) malaria, (b1-2) Chagas disease, (b1-3) leishmaniasis, (b1-4) African trypanosomiasis; (b2-1) lymphatic filariasis, (b2-2) onchocerciasis; (b3-1) dengue, (b3-2) yellow fever, and (b3-3) Zika virus disease. The solid curved line represents the frontier, indicating the lowest potentially achievable ASDR at each level of SDI. Abbreviations: ASDR, age-standardized DALY rate; SDI, Socio-demographic Index.

In the frontier analysis, DALYs from overall, protozoal, and helminth diseases decreased with rising SDI (Fig 4a, 4b1 and 4b2). For viral diseases, dengue increased with SDI in Tropical Latin America, Eastern Sub-Saharan Africa, and East Asia, peaked near 0.6, and then declined (Fig 4b3). Yellow fever declined steadily with SDI, whereas Zika virus disease rose consistently with higher SDI. The results showed the greatest potential for malaria burden reduction in Central Latin America and Southern Sub-Saharan Africa. Chagas disease exhibited the largest improvement space in Eastern Sub-Saharan Africa; leishmaniasis in Central Europe; lymphatic filariasis in South Asia; and onchocerciasis in Central Latin America. Notably, African trypanosomiasis and Zika virus disease revealed significant control gaps in non-endemic regions—Central Latin America and Western Europe, respectively—while dengue in East Asia had burdens significantly exceeding those expected based on SDI levels (Fig 4b).

### Projections of parasitic diseases up to 2030

**Fig 5:**
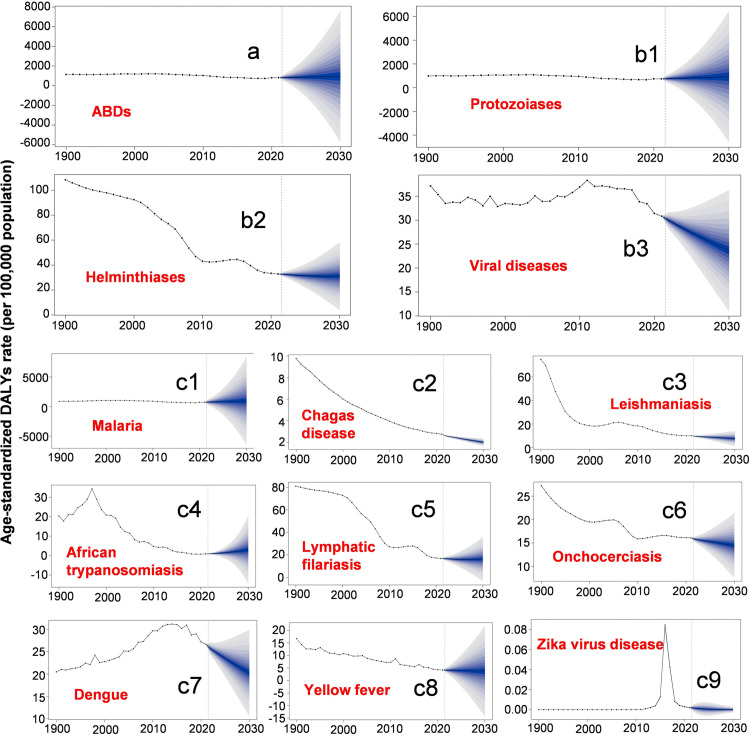
Predicted ASDRs of arthropod-borne diseases worldwide from 2022 to 2030. **(a)** Overall arthropod-borne diseases. **(b)** Major disease categories: (b1) protozoiasis, (b2) helminthiases, and (b3) viral diseases. **(c)** Specific diseases: (c1) malaria, (c2) Chagas disease, (c3) leishmaniasis, (c4) African trypanosomiasis, (c5) lymphatic filariasis, (c6) onchocerciasis, (c7) dengue, (c8) yellow fever, and (c9) Zika virus disease. The horizontal axis represents the year. Abbreviations: ASDR, age-standardized DALY rate.

Using the INLA framework combined with the BAPC model, we projected the global burden of ABDs up to 2030. The results indicated that from 2021 to 2030, the total disease burden will gradually increase, with an estimated rise of about 16.78% (Table K in S1 Text and Fig 5a). The burden of protozoiasis is expected to increase steadily, while that of helminth and viral diseases is predicted to decline (Fig 5b1-5b3). The ASDRs for malaria and African trypanosomiasis show slight increases. In contrast, the ASDRs for other diseases are projected to decrease, with dengue showing the largest decline, estimated as a 31.59% reduction by 2030 (Fig 5c). The ASPRs for malaria, leishmaniasis, and African trypanosomiasis are expected to rise, whereas the ASPR for the remaining diseases is projected to fall (Table F in S1 Text). In the figures, blue shaded areas represent the 95% credible intervals of the projections. The corresponding numerical results are provided in Table L (Fig 5 and Table L in S1 Text).

## Discussion

This study systematically evaluated the epidemiological burden and spatiotemporal trends of ABDs worldwide from 1990 to 2021 and further projected their trajectories up to 2030. Overall, the burden of these diseases exhibits marked geographical clustering and is highly sensitive to regional SDIs. Their transmission and dynamics are jointly driven by factors such as urbanization, climate change, and public health interventions, making ABDs a continuing global public health challenge.

### Disease types and temporal trends

Parasitic diseases, particularly protozoan infections, represent the largest share of the arthropod-borne disease burden [24]. Malaria accounts for nearly 90% of total DALYs, with its burden concentrated in low-SDI regions of Central and Western sub-Saharan Africa [8]. From 2019 to 2021, malaria in low-SDI regions showed a substantial increase. This resurgence might be related to the spread of drug resistance [25], disruption of healthcare services during the COVID-19 pandemic [26], and the expansion of vector-ecological niches resulting from climate change [16]. These findings highlight that progress in malaria control remains fragile, and sustained vigilance is required to address emerging risk factors. Leishmaniasis demonstrated a pattern of increasing ASPR, but declining ASDR in certain regions, suggesting that while transmission remains inadequately controlled, improved access to early diagnosis and treatment has reduced the proportion of severe cases and mortality, thereby lowering overall disability and death burden [27]. Among helminthic diseases, lymphatic filariasis accounted for the largest burden in 1990, but had markedly declined by 2021, largely attributable to the Global Programme to Eliminate Lymphatic Filariasis (GPELF) launched in 2000 and the widespread use of mass drug administration (MDA) [13]. Notably, between 2010 and 2015, the helminthiasis burden in low-middle SDI regions increased temporarily. This was partly associated with shifts in countries’ SDI levels; for example, Sudan and Pakistan moving from low to low-middle SDI and Indonesia from low-middle to middle SDI, which likely redistributed the burden across regions and contributed to the observed fluctuations. For viral diseases, dengue has shown a consistently increasing burden, particularly in high- and high-middle SDI regions, such as High-income Asia Pacific and North America. This rise is closely linked to the strong adaptation of its vector *Aedes* mosquitoes to urban environments and the accelerating effects of global warming [28]. Frontier analysis revealed that in Tropical Latin America, Eastern sub-Saharan Africa, and East Asia, the dengue burden increased with SDI, peaked at around an SDI of 0.6, and then declined. This “inverted V” relationship indicates that during the middle-SDI stage, rapid urbanization, high population density, and lagging public health infrastructure facilitate transmission [29]; however, with further SDI improvement, better urban management, environmental control, and healthcare capacity are likely to drive a subsequent decline [30]. Yellow fever, which posed a substantial burden in 1990 because of limited vaccine coverage and weak health systems, has steadily declined since the 1990s. This reduction reflects the widespread implementation of vaccination and large-scale immunization campaigns, demonstrating the critical role of vaccines in viral disease control [31].

In studies of ABDs, malaria overwhelmingly dominates the total disease burden [32], creating a structural imbalance in aggregated estimates. Consequently, changes in malaria alone can largely drive overall trends, potentially obscuring important epidemiological shifts among other ABDs. On the one hand, the dominance of malaria may mask substantial declines observed in several helminthic and protozoan diseases, such as Chagas disease and African trypanosomiasis [33]. These reductions likely reflect sustained control efforts, improved surveillance, enhanced vector management, and expanded access to diagnosis and treatment. However, when evaluated solely through aggregated ABD indicators, these achievements may appear less pronounced, potentially underestimating the effectiveness of disease-specific control programs. On the other hand, some non-malarial diseases, including leishmaniasis and dengue [34], have shown increasing ASDR trends in certain regions. If policy decisions rely primarily on summary burden estimates, resource allocation may continue to disproportionately prioritize malaria control, potentially overlooking the emerging risks posed by other vector-borne diseases. Therefore, although overall ABD trends provide valuable guidance for public health planning, disease-specific analyses are essential for informing more balanced and forward-looking prevention and control strategies.

### Regional disparities and spatial patterns

The burden of ABDs remains concentrated in low-SDI and lower-middle SDI regions, particularly in Africa, Latin America, and Southeast Asia, where many diseases continue to exert persistent or increasing impacts. This distribution highlights the strong link between socioeconomic development and vulnerability to ABDs [14]. Limited access to healthcare, inadequate vector control programs, rapid urbanization, and fragile public health infrastructure contribute to sustained transmission in these regions. Moreover, environmental and climatic conditions in tropical and subtropical zones favor the proliferation of disease vectors, further amplifying the risk [35]. Notably, rising burdens have also been observed in high-income regions such as High-income Asia Pacific and Australasia. Contributing factors include favorable climatic conditions, global warming, increasing frequency of extreme weather events, high levels of urbanization, and intensive international travel [16]. Importantly, populations in these regions often lack prior immunity and established control mechanisms, making them more susceptible to localized outbreaks. This emphasizes that ABDs are no longer confined to the traditional category of “tropical diseases”, but are increasingly emerging as globalized re-emerging infectious diseases [36], threatening populations even in high-income countries. In addition, certain diseases demonstrated burdens that exceeded their expected levels based on SDI. For example, dengue in East Asia and yellow fever in parts of sub-Saharan Africa presented higher-than-anticipated burdens [37], while control gaps remain evident for African trypanosomiasis in Central Latin America and Zika virus disease in Western Europe. These findings further challenge the conventional geographical classification of ABDs risk.

### Population characteristics

The burden of ABDs shows distinctive demographic patterns. Adolescents and young adults are the primary affected groups [38], while children aged 0–5 years demonstrate the highest DALY rates [39], reflecting their heightened vulnerability due to immature immune systems [40]. Although the overall burden between sexes is relatively balanced, some diseases exhibit clear sex-specific differences [41]. For example, men bear a higher burden of Chagas disease and lymphatic filariasis, primarily resulting from occupational exposure in agriculture and forestry, where prolonged outdoor activities increase contact with disease vectors. In addition to occupational factors, men in endemic regions may have lower uptake of preventive measures, such as insecticide-treated bed nets, and reduced healthcare-seeking behavior during the early stages of infection, which can lead to delayed diagnosis and more advanced disease. Alcohol consumption and migratory labor patterns, which are more common among adult males in some settings, may further increase exposure risk and disrupt continuity of care. Conversely, women show a higher burden of certain parasitic infections, such as echinococcosis [42], likely linked to gender-specific responsibilities in animal husbandry and household activities [43]. These patterns underscore a distinctive feature of arthropod-borne parasitic diseases, where sex differences arise not only from biological susceptibility, but also from social and occupational roles. Moreover, an increase in DALY rates among individuals aged over 80 was observed, likely influenced by smaller population size, but nevertheless highlighting continued vulnerability in elderly populations.

### Future projections and implications

Based on BAPC model predictions, the ASDR of malaria and African trypanosomiasis is projected to increase slightly by 2030, reflecting ongoing challenges, such as drug resistance [44], climate variability, and fragile health systems [45]. In contrast, the ASDR of dengue is expected to decline by approximately 31.6%, driven by integrated efforts in vector control, community engagement, and the adoption of innovative approaches, such as Wolbachia-based interventions [46].

These findings underscore the need for future control strategies to be more targeted, differentiated, and integrated. A strengthened “One Health” approach, fostering cross-sector collaboration between human, animal, and environmental health, will be essential [47]. Key priorities include enhancing surveillance of vector resistance [48], managing animal reservoirs, and assessing ecological impacts. In parallel, integrating climatic, geographical, and mobility data into big-data forecasting platforms could substantially improve early warning and precise interventions. The spectrum of infectious disease threats is evolving dynamically, with increasing complexity [49]. This is reflected not only in the emergence of novel mosquito-borne diseases, such as chikungunya, but also in the expansion of traditional tick-borne diseases into new regions. Addressing such rapidly shifting and intertwined challenges requires a forward-looking, responsive, and adaptable framework. For low-SDI regions, priorities should include building basic entomological and disease surveillance capacity, improving access to diagnostics and treatment, and strengthening community-based vector control measures [50] that are sustainable under resource-limited conditions. Efforts should also focus on strengthening urban infrastructure and vector control in middle-SDI countries, improving preparedness for imported and autochthonous outbreaks in high-income regions, and ensuring equitable vaccine distribution, drug development, and health system support globally [51]. Only through sustained investment, global coordination, and innovation can the control goals for ABDs be achieved, ultimately reducing their worldwide burden.

## Limitations

This study has several limitations. First, constraints in data availability and the GBD classification hierarchy prevented the separate analysis of certain specific ABDs. Pathogens such as tick-borne encephalitis, Ross River virus disease, and loiasis are aggregated into broader categories (e.g., “encephalitis” or “other neglected tropical diseases”) within the GBD 2021 framework, thereby limiting the granularity of our assessment. Second, malaria accounts for over 90% of the total DALY burden, effectively dominating overall trends and potentially obscuring the distinct epidemiological profiles of viral and helminthic infections. This statistical dominance is further amplified by the robust surveillance systems for malaria relative to other neglected diseases. To mitigate this, we stratified ABDs into three pathogenic categories and conducted disease-specific analyses; nevertheless, uneven data coverage suggests that the burden of less-monitored neglected ABDs may remain underestimated. Third, variations in reporting quality, characterized by underreporting and inconsistent diagnostic standards across national systems, complicate global comparisons. Fourth, while the standardized GBD modeling framework addresses data gaps, its underlying assumptions may not fully capture localized transmission dynamics, necessitating caution in interpreting long-term projections. Finally, the BAPC model relies on historical trends and assumes continuity in age, period, and cohort effects; as such, it may not adequately capture abrupt epidemiological shifts caused by pandemics, climate-related events, or novel public health interventions. Future projections could be improved by integrating more dynamic or mechanistic models capable of better reflecting rapidly evolving epidemiological conditions.

## Conclusions

The global burden of ABDs remains predominantly driven by protozoan infections, particularly malaria, which is heavily concentrated in the low-SDI regions of sub-Saharan Africa. Concurrently, viral diseases such as dengue are exhibiting concerning upward trends in middle- and high-SDI regions, fueled by rapid urbanization, globalization, and climate change. Our analysis reveals that in specific contexts, such as dengue in East Asia and yellow fever in sub-Saharan Africa, the disease burden significantly exceeds levels expected for the region’s sociodemographic development, thereby identifying critical priorities for intervention. Vulnerability remains most pronounced among children and the elderly, further emphasizing the need for targeted protection. Ultimately, the strong geographical clustering and expanding reach of ABDs underscore the necessity of a “One Health” approach: strengthening surveillance, expanding vaccine coverage, and fostering cross-sectoral collaboration will be essential to sustainably mitigate this evolving global threat.

## Supporting information

S1 Text Table A. DALYs, ASDRs in 1990 and 2021, and AAPCs from 1990 to 2021 for arthropod-borne diseases, protozoiasis, helminthiases, and viral diseases in global and 21 regions. **Table B**. DALYs, ASDRs in 1990 and 2021, and AAPCs from 1990 to 2021 for arthropod-borne diseases other than malaria in global and 21 regions. **Table C.** Prevalence, ASPRs in 1990 and 2021, and AAPC from 1990 to 2021 for nine arthropod-borne diseases in global and 21 regions. T**able D.** DALYs, ASDRs in 1990 and 2021, and AAPCs from 1990 to 2021 for nine arthropod-borne diseases in global and 21 regions. **Table E.** ASPRs in 2021 for nine arthropod-borne diseases in 204 countries and territories. **Table F**. ASPR AAPCs (1990–2021) for nine arthropod-borne diseases in 204 countries and territories. **Table G**. ASDRs in 2021 for arthropod-borne diseases, protozoiasis, helminthiases, viral diseases and other than malaria in 204 countries and territories. **Table H.** ASDRs in 2021 for nine arthropod-borne diseases in 204 countries and territories. Table I. ASDR AAPCs (1990–2021) for arthropod-borne diseases, protozoiasis, helminthiases, viral diseases, and other than malaria in 204 countries and territories. **Table J.** ASDR AAPCs (1990–2021) for nine arthropod-borne diseases in 204 countries and territories. **Table K**. Estimated ASDRs for selected infectious diseases through 2030. **Table L.** Projections of age-standardized death rates (ASDR) for ABDs, 1990–2030, with 95% credible intervals. **Fig A**. Global and five SDI regions’ changes in ASPRs per 100,000 population for arthropod-borne diseases from 1990 to 2021. ASPR, age-standardized prevalence rates; APC, annual percentage change; AAPC, average annual percentage change; SDI, Socio-demographic Index, *P < 0.05. No data available for Zika virus. **Fig B**. Global and five SDI regions’ changes in ASDRs per 100,000 population for arthropod-borne diseases from 1990 to 2021. ASDR age-standardized DALYs rates, APC annual percentage change, AAPC average annual percentage change, SDI Socio-demographic Index, *P < 0.05. No data available for Zika virus. **Fig C.** Ranking of ASPRs for Arthropod-Borne Diseases by 21 Regions. **Fig D**. Number of prevalence and ASPRs of nine arthropod-borne diseases across different age groups globally for both sexes in 2021. ASPRs, age-standardized prevalence rates. **Fig E**. Frontier Analysis of ASDRs of Arthropod-Borne Diseases Across 21 Regions in Relation to SDI. ASPRs, age-standardized prevalence rates; SDI, Socio-demographic Index. **Fig F**. Predicted ASPRs of nine arthropod-borne diseases worldwide from 2022 to 2030. ASPRs, age-standardized prevalence rates.
(DOCX)
