# Supplementary material for: Global, regional, and national disease burden of arthropod-borne diseases: Projections to 2030 based on the global burden of disease study 2021
Source: PLoS Negl Trop Dis. 2026 May 5;20(5):e0014235. doi: 10.1371/journal.pntd.0014235 (PMC13143084; doi:10.1371/journal.pntd.0014235)
Supplement: S1 Text — DALYs, ASDRs in 1990 and 2021, and AAPCs from 1990 to 2021 for arthropod-borne diseases, protozoiasis, helminthiases, and viral diseases in global and 21 regions. Table B. DALYs, ASDRs in 1990 and 2021, and AAPCs from 1990 to 2021 for arthropod-borne diseases other than malaria in global and 21 regions. Table C. Prevalence, ASPRs in 1990 and 2021, and AAPC from 1990 to 2021 for nine arthropod-borne diseases in global and 21 regions. Table D. DALYs, ASDRs in 1990 and 2021, and AAPCs from 1990 to 2021 for nine arthropod-borne diseases in global and 21 regions. Table E. ASPRs in 2021 for nine arthropod-borne diseases in 204 countries and territories. Table F. ASPR AAPCs (1990–2021) for nine arthropod-borne diseases in 204 countries and territories. Table G. ASDRs in 2021 for arthropod-borne diseases, protozoiasis, helminthiases, viral diseases and other than malaria in 204 countries and territories. Table H. ASDRs in 2021 for nine arthropod-borne diseases in 204 countries and territories. Table I. ASDR AAPCs (1990–2021) for arthropod-borne diseases, protozoiasis, helminthiases, viral diseases, and other than malaria in 204 countries and territories. Table J. ASDR AAPCs (1990–2021) for nine arthropod-borne diseases in 204 countries and territories. Table K. Estimated ASDRs for selected infectious diseases through 2030. Table L. Projections of age-standardized death rates (ASDR) for ABDs, 1990–2030, with 95% credible intervals. Fig A. Global and five SDI regions’ changes in ASPRs per 100,000 population for arthropod-borne diseases from 1990 to 2021. ASPR, age-standardized prevalence rates; APC, annual percentage change; AAPC, average annual percentage change; SDI, Socio-demographic Index, *P < 0.05. No data available for Zika virus. Fig B. Global and five SDI regions’ changes in ASDRs per 100,000 population for arthropod-borne diseases from 1990 to 2021. ASDR age-standardized DALYs rates, APC annual percentage change, AAPC average annual percentage change, SDI Socio-demo [file pntd.0014235.s001.docx]

**Global, regional, and national disease burden of arthropod-borne diseases: projections to 2030 based on the Global Burden of Disease Study 2021**

**Short title**:

Mengqing Li^1,2,3^, Yang Yang^1,2,3,4^, Chuizhao Xue^1,2,3^, Qingqiu Zuo^1,2,3^, Ying Wang^1,2,3^, Hua Liu^1,2,3^, Yefei Pu^1,2,3,4^, Yujuan Shen^1,2,3^, Xu Wang^1,2,3*^, Jianhai Yin^1,2,3*^, Jianping Cao^1,2,3,4*^

**Table A** DALYs, ASDRs in 1990 and 2021, and AAPCs from 1990 to 2021 for arthropod-borne diseases, protozoiasis, helminthiases, viral diseases in global and 21 regions

**Table B** DALYs, ASDRs in 1990 and 2021, and AAPCs from 1990 to 2021 for arthropod-borne diseases other than malaria in global and 21 regions

**Table C** Prevalence, ASPRs in 1990 and 2021, and AAPC from 1990 to 2021 for nine arthropod-borne diseases in global and 21 regions

**Table D** DALYs, ASDRs in 1990 and 2021, and AAPCs from 1990 to 2021 for nine arthropod-borne diseases in global and 21 regions

**Table E** ASPRs in 2021 for nine arthropod-borne diseases in 204 countries and territories

**Table F** ASPR AAPCs (1990–2021) for nine arthropod-borne diseases in 204 countries and territories

**Table G** ASDRs in 2021 for arthropod-borne diseases, protozoiasis, helminthiases, viral diseases and other than malaria in 204 countries and territories

**Table H** ASDRs in 2021 for nine arthropod-borne diseases in 204 countries and territories

**Table I** ASDR AAPCs (1990–2021) for arthropod-borne diseases, protozoiasis, helminthiases, viral diseases and other than malaria in 204 countries and territories

**Table J** ASDR AAPCs (1990–2021) for nine arthropod-borne diseases in 204 countries and territories

**Table K** Estimated ASDRs for selected infectious diseases through 2030

**Table L** Projections of age-standardized death rates (ASDR) for ABDs, 1990–2030, with 95% credible intervals

**Fig A** Global and five SDI regions’ changes in ASPRs per 100,000 population for arthropod-borne diseases from 1990 to 2021. ASPR age-standardized prevalence rates, APC annual percentage change, AAPC average annual percentage change, SDI Socio-demographic Index, **P* < 0.05. No data available for Zika virus

**Fig B** Global and five SDI regions’ changes in ASDRs per 100,000 population for arthropod-borne diseases from 1990 to 2021. ASDR age-standardized DALYs rates, APC annual percentage change, AAPC average annual percentage change, SDI Socio-demographic Index, **P* < 0.05. No data available for Zika virus

**Fig C** Ranking of ASPRs for Arthropod-Borne Diseases by 21 Regions

**Fig D** Number of prevalence and ASPRs of nine arthropod-borne diseases across different age groups globally for both sexes in 2021. ASPRs age-standardized prevalence rates

**Fig E** Frontier Analysis of ASDRs of Arthropod-Borne Diseases Across 21 Regions in Relation to SDI. ASPRs age-standardized prevalence rates, SDI Socio-demographic Index

**Fig F** Predicted ASPRs of nine arthropod-borne diseases worldwide from 2022 to 2030. ASPRs age-standardized prevalence rates

**Table A** DALYs, ASDRs in 1990 and 2021, and AAPCs from 1990 to 2021 for arthropod-borne diseases, protozoiasis, helminthiases, viral diseases in global and 21 regions

| **Location** | **1990 DALYs** | **2021 DALYs** | **1990 ASDRs**  **(1/100,000, 95% UI)** | **2021 ASDRs**  **(1/100,000, 95% UI)** | **AAPCs**  **(95% CI)** | ***P* Value** |
| --- | --- | --- | --- | --- | --- | --- |
| Global | 71,749,012.92 (29,800,709.35, 113,697,316.49) | 61,227,826.19 (17,912,189.53, 104,543,462.85) | 1219.26 (512.21, 1926.32) | 884.16 (258.21, 1510.10) | -0.97  (-1.39, -0.56) | <0.05 |
| High SDI | 22,737.31 (5323.62, 40,151.00) | 20,530.34 (11,690.74, 29,369.93) | 2.67 (0.29, 5.05) | 1.72 (0.96, 2.49) | -1.13  (-2.46, 0.23) | 0.1 |
| High-middle SDI | 308,299.00 (44,531.14, 572,066.86) | 184,347.71 (132,899.32, 235,796.10) | 30.47 (3.78, 57.16) | 16.91 (11.81, 22.02) | -2.05  (-2.76, -1.33) | <0.05 |
| Middle SDI | 7,968,343.01 (2,762,446.30, 13,174,239.71) | 5,304,620.47 (2,501,473.64, 8,107,767.30) | 444.42 (163.81, 725.03) | 255.62 (121.17, 390.07) | -1.81  (-2.36, -1.27) | <0.05 |
| Low-middle SDI | 18,837,374.91 (4,976,337.83, 32,698,412.00) | 13,893,858.37 (4,238,687.62, 23,549,029.11) | 1362.07 (425.73, 2298.40) | 735.61 (222.25, 1248.96) | -1.88  (-2.26, -1.50) | <0.05 |
| Low SDI | 44,577,034.31 (22,147,678.81, 67,006,389.82) | 41,801,130.47 (11,657,802.83, 71,944,458.10) | 6158.08 (3098.97, 9217.20) | 3080.09 (791.09, 5369.08) | -2.13  (-2.37, -1.89) | <0.05 |
| East Asia | 131,447.41 (0.00, 522,368.95) | 11,406.58 (0.00, 35,463.93) | 10.55 (0.00, 41.15) | 0.93 (0.00, 3.09) | -7.77  (-8.69, -6.83) | <0.05 |
| Southeast Asia | 2,570,754.34 (905,926.01, 4,235,582.67) | 1,219,618.91 (836,056.00, 1,603,181.83) | 537.49 (191.93, 883.05) | 191.03 (130.98, 251.08) | -3.20  (-3.92, -2.48) | <0.05 |
| Oceania | 207,771.98 (0.00, 437,427.87) | 167,035.24 (67,081.16, 266,989.32) | 3245.18 (0.00, 6973.16) | 1217.03 (486.03, 1948.03) | -3.41  (-6.24, -0.48) | <0.05 |
| Central Asia | 38,898.29 (0.00, 78,429.10) | 8870.79 (0.00, 20,295.86) | 46.76 (4.16, 89.36) | 9.43 (0.00, 21.21) | -5.89  (-8.51, -3.19) | <0.05 |
| Central Europe | 9269.18 (0.00, 53,200.39) | 385.72 (0.00, 1851.83) | 8.47 (0.00, 48.72) | 0.43 (0.00, 2.31) | -9.09  (-10.18, -7.98) | <0.05 |
| Eastern Europe | 0.00 (0.00, 0.00) | 0.00 (0.00, 0.00) | 0.00 (0.00, 0.00) | 0.00 (0.00, 0.00) | NA | NA |
| High-income Asia Pacific | 4433.62 (1568.29, 7298.96) | 4975.66 (0.00, 10,560.81) | 2.59 (0.88, 4.29) | 3.05 (0.00, 6.48) | 0.52  (0.02, 1.02) | <0.05 |
| Australasia | 85.05 (0.00, 183.88) | 210.01 (0.00, 434.38) | 0.41 (0.00, 0.90) | 0.66 (0.00, 1.38) | 1.52  (0.76, 2.28) | <0.05 |
| Western Europe | 5581.29 (0.00, 20,931.01) | 2113.79 (0.00, 5970.85) | 1.73 (0.00, 6.94) | 0.56 (0.00, 1.84) | -3.45  (-4.29, -2.61) | <0.05 |
| Southern Latin America | 50,078.35 (35,398.74, 64,757.95) | 22,768.41 (16,508.59, 29,028.22) | 106.75 (76.68, 136.82) | 28.74 (20.38, 37.09) | -4.13  (-4.27, -3.98) | <0.05 |
| High-income North America | 2018.90 (1209.33, 2828.47) | 2714.50 (1618.43, 3810.57) | 0.62 (0.37, 0.87) | 0.54 (0.32, 0.77) | -0.38  (-0.58, -0.18) | <0.05 |
| Caribbean | 97,147.10 (40,503.73, 153,790.47) | 62,436.52 (0.00, 133,330.40) | 267.06 (116.65, 417.47) | 134.35 (0.00, 288.13) | -4.65  (-9.66, 0.63) | 0.08 |
| Andean Latin America | 163,897.19 (37,057.53, 290,736.85) | 50,484.81 (38,613.62, 62,356.00) | 447.06 (142.23, 751.90) | 78.86 (60.43, 97.29) | -5.48  (-7.68, -3.24) | <0.05 |
| Central Latin America | 180,150.74 (119,974.29, 240,327.20) | 156,784.91 (112,455.76, 201,114.05) | 127.99 (95.04, 160.94) | 62.61 (44.67, 80.55) | -2.16  (-2.80, -1.52) | <0.05 |
| Tropical Latin America | 766,590.22 (482,730.10, 1,050,450.34) | 426,665.09 (243,613.90, 609,716.29) | 574.76 (399.62, 749.89) | 182.49 (96.04, 268.94) | -3.72  (-4.44, -3.00) | <0.05 |
| North Africa and Middle East | 1,285,633.97 (4701.64, 2,566,566.30) | 1,108,420.54 (360,160.58, 1,856,680.50) | 342.75 (26.95, 658.55) | 178.44 (58.36, 298.52) | -2.18  (-2.99, -1.37) | <0.05 |
| South Asia | 12,816,869.62 (287,029.87, 25,346,709.38) | 3,593,767.12 (544,024.99, 6,643,509.24) | 1002.08 (70.81, 1933.35) | 203.07 (28.47, 377.67) | -5.34  (-6.31, -4.37) | <0.05 |
| Central Sub-Saharan Africa | 9,465,812.19 (5,399,381.56, 13,532,242.83) | 8,068,049.20 (3,305,532.26, 12,830,566.14) | 11,352.97 (7014.40, 15,691.55) | 4808.84 (1906.21, 7711.48) | -2.79  (-3.23, -2.34) | <0.05 |
| Eastern Sub-Saharan Africa | 18,572,692.84 (10,242,094.01, 26,903,291.68) | 12,159,510.10 (3,523,829.78, 20,795,190.42) | 6513.20 (3672.28, 9354.13) | 2336.02 (600.10, 4071.93) | -3.19  (-3.73, -2.64) | <0.05 |
| Southern Sub-Saharan Africa | 248,718.69 (37,005.34, 460,432.03) | 149,827.51 (28,627.28, 271,027.74) | 382.33 (38.75, 725.91) | 184.68 (35.07, 334.28) | -1.30  (-9.15, 7.22) | 0.76 |
| Western Sub-Saharan Africa | 25,131,161.95 (11,196,974.28, 39,065,349.62) | 34,011,780.78 (8,980,313.15, 59,043,248.41) | 9319.66 (4354.38, 14,284.93) | 5857.55 (1401.48, 10,313.61) | -1.42  (-1.68, -1.16) | <0.05 |
| **Protozoiasis** |  |  |  |  |  |  |
| Global | 64,057,769.37 (22,150,317.59, 105,965,221.14) | 56,255,800.08 (12,963,474.53, 99,548,125.63) | 1074.79 (368.60, 1780.99) | 819.83 (194.16, 1445.51) | -0.81  (-1.25, -0.36) | <0.05 |
| High SDI | 19,197.36 (2291.36, 36,103.37) | 14,035.56 (8440.75, 19,630.37) | 2.25 (0.00, 4.58) | 1.14 (0.70, 1.59) | -1.93  (-3.26, -0.59) | <0.05 |
| High-middle SDI | 154,900.63 (0.00, 415,609.11) | 45,530.53 (29,619.17, 61,441.89) | 15.51 (0.00, 41.90) | 3.18 (1.64, 4.72) | -4.32  (-5.09, -3.54) | <0.05 |
| Middle SDI | 6,093,060.02 (907,272.04, 11,278,848.00) | 3,857,562.72 (1,103,933.27, 6,611,192.17) | 333.90 (54.63, 613.17) | 190.87 (58.45, 323.29) | -1.73  (-2.59, -0.87) | <0.05 |
| Low-middle SDI | 15,920,199.28 (2,080,242.16, 29,760,156.40) | 12,288,883.52 (2,650,797.99, 21,926,969.04) | 1085.33 (151.86, 2018.81) | 649.58 (137.12, 1162.03) | -1.64  (-2.18, -1.08) | <0.05 |
| Low SDI | 41,841,861.37 (19,425,739.71, 64,257,983.03) | 40,029,766.33 (9,891,703.58, 70,167,829.08) | 5510.21 (2456.09, 8564.33) | 2898.47 (610.17, 5186.76) | -1.98  (-2.28, -1.68) | <0.05 |
| East Asia | 127,557.62 (0.00, 518,475.44) | 10,046.56 (0.00, 34,083.69) | 10.21 (0.00, 40.81) | 0.83 (0.00, 3.00) | -8.01  (-8.95, -7.06) | <0.05 |
| Southeast Asia | 884,676.28 (0.00, 2,474,881.93) | 111,552.33 (0.00, 275,780.80) | 177.13 (0.00, 507.66) | 16.21 (0.00, 39.66) | -7.18  (-8.97, -5.36) | <0.05 |
| Oceania | 173,720.07 (0.00, 403,060.71) | 146,292.01 (46,668.93, 245,915.09) | 2624.23 (0.00, 6345.53) | 1047.48 (319.38, 1775.58) | -3.59  (-6.52, -0.57) | <0.05 |
| Central Asia | 38,898.29 (0.00, 78,429.10) | 8870.79 (0.00, 20,295.86) | 46.76 (4.16, 89.36) | 9.43 (0.00, 21.21) | -5.89  (-8.52, -3.19) | <0.05 |
| Central Europe | 9269.18 (0.00, 53,200.39) | 385.72 (0.00, 1851.83) | 8.47 (0.00, 48.72) | 0.43 (0.00, 2.31) | -9.09  (-10.18, -7.98) | <0.05 |
| Eastern Europe | 0.00 (0.00, 0.00) | 0.00 (0.00, 0.00) | 0.00 (0.00, 0.00) | 0.00 (0.00, 0.00) | NA | NA |
| High-income Asia Pacific | 2164.81 (1699.87, 2629.75) | 123.42 (93.46, 153.37) | 1.23 (0.96, 1.50) | 0.05 (0.04, 0.06) | -9.76  (-10.25, -9.27) | <0.05 |
| Australasia | 25.35 (14.66, 36.05) | 18.28 (10.52, 26.03) | 0.11 (0.07, 0.16) | 0.04 (0.03, 0.06) | -3.08  (-3.73, -2.42) | <0.05 |
| Western Europe | 5490.51 (0.00, 20,840.13) | 2112.30 (0.00, 5969.36) | 1.72 (0.00, 6.92) | 0.56 (0.00, 1.83) | -3.41  (-4.30, -2.52) | <0.05 |
| Southern Latin America | 42,113.77 (32,594.84, 51,632.71) | 20,032.71 (14,694.77, 25,370.65) | 91.00 (70.49, 111.51) | 24.46 (17.87, 31.05) | -4.13  (-4.26, -4.01) | <0.05 |
| High-income North America | 2011.71 (1202.16, 2821.27) | 2672.41 (1577.60, 3767.21) | 0.61 (0.36, 0.86) | 0.53 (0.31, 0.75) | -0.47  (-0.65, -0.29) | <0.05 |
| Caribbean | 57,827.80 (2808.99, 112,846.62) | 49,449.86 (0.00, 120,078.98) | 152.33 (7.14, 297.52) | 107.11 (0.00, 260.34) | -4.70  (-11.42, 2.53) | 0.2 |
| Andean Latin America | 148,196.26 (22,396.13, 273,996.38) | 43,039.50 (32,244.72, 53,834.28) | 409.29 (106.71, 711.88) | 67.64 (50.77, 84.52) | -5.77  (-7.35, -4.17) | <0.05 |
| Central Latin America | 143,774.51 (85,442.31, 202,106.70) | 112,690.38 (73,999.54, 151,381.22) | 104.45 (72.79, 136.12) | 44.68 (29.07, 60.28) | -2.74  (-3.50, -1.97) | <0.05 |
| Tropical Latin America | 688,096.67 (417,579.07, 958,614.27) | 280,633.73 (149,597.18, 411,670.29) | 524.17 (357.96, 690.37) | 117.76 (52.59, 182.92) | -4.75  (-5.59, -3.90) | <0.05 |
| North Africa and Middle East | 1,106,162.67 (0.00, 2,373,850.54) | 1,059,150.99 (311,766.51, 1,806,535.48) | 292.03 (0.00, 604.49) | 170.70 (50.75, 290.65) | -1.63  (-2.53, -0.73) | <0.05 |
| South Asia | 10,421,829.60 (0.00, 22,928,173.20) | 1,926,391.38 (0.00, 4,877,608.98) | 750.80 (0.00, 1678.63) | 109.98 (0.00, 279.41) | -6.67  (-8.55, -4.75) | <0.05 |
| Central Sub-Saharan Africa | 8,792,061.91 (4,731,836.83, 12,852,287.00) | 7,303,091.27 (2,550,157.70, 12,056,024.83) | 9796.52 (5487.30, 14,105.74) | 4118.52 (1227.65, 7009.39) | -2.81  (-3.12, -2.51) | <0.05 |
| Eastern Sub-Saharan Africa | 17,767,964.49 (9,441,811.23, 26,094,117.75) | 11,761,659.96 (3,127,188.92, 20,396,131.00) | 6004.50 (3167.44, 8841.57) | 2228.49 (492.97, 3964.01) | -3.07  (-3.65, -2.50) | <0.05 |
| Southern Sub-Saharan Africa | 245,418.18 (33,713.62, 457,122.73) | 145,636.17 (24,461.31, 266,811.03) | 375.92 (32.36, 719.48) | 179.61 (30.04, 329.19) | 0.05  (-1.89, 2.03) | 0.96 |
| Western Sub-Saharan Africa | 23,400,509.68 (9,478,945.71, 37,322,073.64) | 33,271,950.31 (8,241,845.02, 58,302,055.59) | 8280.02 (3325.22, 13,234.82) | 5678.06 (1222.41, 10,133.71) | -1.17  (-1.47, -0.88) | <0.05 |
| **Helminths** |  |  |  |  |  |  |
| Global | 5,454,609.47 (3,849,056.99, 7,060,161.95) | 2,577,551.52 (1,648,634.65, 3,506,468.40) | 105.77 (74.82, 136.72) | 32.27 (20.59, 43.95) | -3.73  (-3.93, -3.53) | <0.05 |
| High SDI | 0.00 (0.00, 0.00) | 0.00 (0.00, 0.00) | 0.00 (0.00, 0.00) | 0.00 (0.00, 0.00) | NA | NA |
| High-middle SDI | 58,104.31 (34,006.06, 82,202.57) | 26,735.44 (10,527.56, 42,943.33) | 5.31 (3.11, 7.51) | 2.16 (0.86, 3.46) | -2.77  (-3.10, -2.43) | <0.05 |
| Middle SDI | 1,156,169.28 (754,637.68, 1,557,700.87) | 393,966.46 (192,983.05, 594,949.86) | 72.16 (47.37, 96.96) | 15.65 (7.66, 23.63) | -4.80  (-5.46, -4.14) | <0.05 |
| Low-middle SDI | 2,103,850.47 (1,421,117.32, 2,786,583.63) | 746,450.01 (422,932.68, 1,069,967.33) | 214.39 (146.24, 282.55) | 39.52 (22.61, 56.44) | -5.29  (-5.53, -5.05) | <0.05 |
| Low SDI | 2,131,286.02 (1,522,906.56, 2,739,665.49) | 1,408,592.85 (897,207.92, 1,919,977.79) | 542.74 (388.87, 696.60) | 150.97 (97.31, 204.63) | -4.06  (-4.19, -3.93) | <0.05 |
| East Asia | 0.00 (0.00, 0.00) | 0.00 (0.00, 0.00) | 0.00 (0.00, 0.00) | 0.00 (0.00, 0.00) | NA | NA |
| Southeast Asia | 943,883.71 (591,215.67, 1,296,551.75) | 198,952.38 (81,994.06, 315,910.70) | 225.37 (142.92, 307.82) | 27.78 (11.47, 44.09) | -6.48  (-6.91, -6.05) | <0.05 |
| Oceania | 33,106.12 (21,083.27, 45,128.97) | 19,976.45 (11,870.66, 28,082.24) | 607.52 (384.68, 830.35) | 163.51 (98.62, 228.39) | -4.18  (-4.42, -3.95) | <0.05 |
| Central Asia | 0.00 (0.00, 0.00) | 0.00 (0.00, 0.00) | 0.00 (0.00, 0.00) | 0.00 (0.00, 0.00) | NA | NA |
| Central Europe | 0.00 (0.00, 0.00) | 0.00 (0.00, 0.00) | 0.00 (0.00, 0.00) | 0.00 (0.00, 0.00) | NA | NA |
| Eastern Europe | 0.00 (0.00, 0.00) | 0.00 (0.00, 0.00) | 0.00 (0.00, 0.00) | 0.00 (0.00, 0.00) | NA | NA |
| High-income Asia Pacific | 93.58 (36.14, 151.02) | 139.03 (52.26, 225.79) | 0.06 (0.02, 0.09) | 0.09 (0.04, 0.15) | 1.74  (1.50, 1.98) | <0.05 |
| Australasia | 0.00 (0.00, 0.00) | 0.00 (0.00, 0.00) | 0.00 (0.00, 0.00) | 0.00 (0.00, 0.00) | NA | NA |
| Western Europe | 0.00 (0.00, 0.00) | 0.00 (0.00, 0.00) | 0.00 (0.00, 0.00) | 0.00 (0.00, 0.00) | NA | NA |
| Southern Latin America | 0.00 (0.00, 0.00) | 0.00 (0.00, 0.00) | 0.00 (0.00, 0.00) | 0.00 (0.00, 0.00) | NA | NA |
| High-income North America | 0.00 (0.00, 0.00) | 0.00 (0.00, 0.00) | 0.00 (0.00, 0.00) | 0.00 (0.00, 0.00) | NA | NA |
| Caribbean | 36,970.97 (23,748.26, 50,193.69) | 10,123.51 (4992.52, 15,254.50) | 108.22 (69.60, 146.85) | 21.17 (10.38, 31.95) | -5.04  (-6.15, -3.93) | <0.05 |
| Andean Latin America | 1143.62 (699.22, 1588.01) | 0.00 (0.00, 0.00) | 3.54 (2.20, 4.88) | 0.00 (0.00, 0.00) | NA | NA |
| Central Latin America | 20,348.18 (13,236.96, 27,459.39) | 451.34 (137.35, 765.34) | 14.22 (9.40, 19.04) | 0.17 (0.05, 0.29) | -13.35  (-13.81, -12.89) | <0.05 |
| Tropical Latin America | 8198.90 (5307.47, 11,090.32) | 1565.56 (992.21, 2138.92) | 5.55 (3.59, 7.51) | 0.69 (0.43, 0.95) | -6.52  (-6.94, -6.11) | <0.05 |
| North Africa and Middle East | 52,030.42 (26,775.45, 77,285.39) | 24,773.06 (12,062.91, 37,483.20) | 17.94 (9.57, 26.32) | 3.94 (1.94, 5.93) | -4.83  (-5.37, -4.29) | <0.05 |
| South Asia | 2,006,046.43 (1,269,423.37, 2,742,669.49) | 735,707.66 (330,003.96, 1,141,411.36) | 215.49 (137.93, 293.04) | 39.63 (17.99, 61.27) | -5.27  (-5.71, -4.83) | <0.05 |
| Central Sub-Saharan Africa | 586,561.36 (385,701.52, 787,421.19) | 728,108.19 (428,771.41, 1,027,444.98) | 1415.23 (936.73, 1893.74) | 666.31 (406.57, 926.05) | -2.42  (-2.58, -2.27) | <0.05 |
| Eastern Sub-Saharan Africa | 562,510.68 (402,730.00, 722,291.36) | 298,895.16 (189,651.05, 408,139.27) | 396.98 (286.56, 507.41) | 87.57 (55.51, 119.63) | -4.77  (-4.98, -4.56) | <0.05 |
| Southern Sub-Saharan Africa | 3291.93 (1362.88, 5220.98) | 4179.06 (1699.66, 6658.46) | 6.40 (2.62, 10.17) | 5.05 (2.08, 8.02) | -0.74  (-0.99, -0.50) | <0.05 |
| Western Sub-Saharan Africa | 1,200,423.58 (826,464.76, 1,574,382.39) | 554,680.12 (336,328.95, 773,031.29) | 797.85 (555.40, 1040.30) | 147.59 (91.95, 203.24) | -5.42  (-6.07, -4.77) | <0.05 |
| **Viruses** |  |  |  |  |  |  |
| Global | 2,236,634.08 (1,315,841.30, 3,157,426.87) | 2,394,474.59 (1,319,281.11, 3,469,668.07) | 38.70 (22.73, 54.66) | 32.05 (17.80, 46.29) | -0.63  (-1.23, -0.03) | <0.05 |
| High SDI | 3539.95 (0.00, 7714.09) | 6494.78 (0.00, 13,338.50) | 0.42 (0.00, 0.91) | 0.58 (0.00, 1.21) | 1.00  (0.60, 1.40) | <0.05 |
| High-middle SDI | 95,294.06 (63,296.47, 127,291.65) | 112,081.73 (65,918.22, 158,245.24) | 9.65 (6.28, 13.02) | 11.57 (6.88, 16.26) | 0.39  (-0.11, 0.88) | 0.12 |
| Middle SDI | 719,113.71 (500,628.15, 937,599.28) | 1,053,091.30 (568,568.16, 1,537,614.43) | 38.35 (26.71, 49.98) | 49.10 (27.26, 70.95) | 0.75  (-0.12, 1.62) | 0.09 |
| Low-middle SDI | 813,325.16 (470,088.52, 1,156,561.81) | 858,524.85 (384,219.00, 1,332,830.69) | 62.34 (35.82, 88.87) | 46.50 (21.27, 71.74) | -0.98  (-1.45, -0.51) | <0.05 |
| Low SDI | 603,886.92 (131,285.38, 1,076,488.46) | 362,771.28 (126,424.49, 599,118.07) | 105.14 (22.47, 187.80) | 30.65 (12.18, 49.13) | -3.84  (-4.03, -3.65) | <0.05 |
| East Asia | 3889.78 (2182.56, 5597.01) | 1360.02 (373.99, 2346.04) | 0.34 (0.19, 0.49) | 0.09 (0.03, 0.16) | -4.10  (-5.01, -3.20) | <0.05 |
| Southeast Asia | 742,194.35 (397,920.33, 1,086,468.36) | 909,114.20 (582,816.38, 1,235,412.03) | 134.99 (77.00, 192.98) | 147.04 (94.22, 199.86) | 0.44  (-0.22, 1.10) | 0.19 |
| Oceania | 945.79 (556.15, 1335.44) | 766.78 (170.29, 1363.27) | 13.43 (7.31, 19.55) | 6.04 (1.47, 10.62) | -2.67  (-3.92, -1.40) | <0.05 |
| Central Asia | 0.00 (0.00, 0.00) | 0.00 (0.00, 0.00) | 0.00 (0.00, 0.00) | 0.00 (0.00, 0.00) | NA | NA |
| Central Europe | 0.00 (0.00, 0.00) | 0.00 (0.00, 0.00) | 0.00 (0.00, 0.00) | 0.00 (0.00, 0.00) | NA | NA |
| Eastern Europe | 0.00 (0.00, 0.00) | 0.00 (0.00, 0.00) | 0.00 (0.00, 0.00) | 0.00 (0.00, 0.00) | NA | NA |
| High-income Asia Pacific | 2175.23 (0.00, 5002.01) | 4713.22 (0.00, 10,297.61) | 1.30 (0.00, 2.99) | 2.90 (0.00, 6.34) | 2.54  (1.80, 3.28) | <0.05 |
| Australasia | 59.70 (0.00, 157.94) | 191.74 (0.00, 415.97) | 0.30 (0.00, 0.78) | 0.61 (0.00, 1.33) | 2.53  (2.06, 3.01) | <0.05 |
| Western Europe | 90.77 (33.46, 148.08) | 1.49 (0.37, 2.60) | 0.02 (0.01, 0.03) | 0.00 (0.00, 0.00) | -13.30  (-18.90, -7.32) | <0.05 |
| Southern Latin America | 7964.58 (0.00, 19,139.58) | 2735.70 (0.00, 6005.51) | 15.75 (0.00, 37.73) | 4.27 (0.00, 9.40) | -4.10  (-4.44, -3.76) | <0.05 |
| High-income North America | 7.18 (1.99, 12.38) | 42.09 (0.00, 94.73) | 0.00 (0.00, 0.00) | 0.02 (0.00, 0.03) | 5.74  (4.74, 6.76) | <0.05 |
| Caribbean | 2348.32 (0.00, 4909.72) | 2863.15 (0.00, 6201.32) | 6.50 (0.00, 13.71) | 6.08 (0.00, 13.24) | 0.49  (-0.63, 1.62) | 0.39 |
| Andean Latin America | 14,557.32 (0.00, 30,757.01) | 7445.31 (2505.89, 12,384.73) | 34.23 (0.00, 71.18) | 11.22 (3.80, 18.63) | -4.10  (-4.87, -3.32) | <0.05 |
| Central Latin America | 16,028.06 (3066.90, 28,989.23) | 43,643.18 (22,010.02, 65,276.34) | 9.32 (1.59, 17.05) | 17.76 (8.92, 26.60) | 1.84  (0.38, 3.32) | <0.05 |
| Tropical Latin America | 70,294.65 (0.00, 156,250.64) | 144,465.80 (16,650.18, 272,281.42) | 45.04 (0.00, 100.21) | 64.04 (7.24, 120.85) | 1.02  (0.47, 1.56) | <0.05 |
| North Africa and Middle East | 127,440.88 (0.00, 309,422.40) | 24,496.49 (0.00, 58,376.51) | 32.78 (0.00, 77.83) | 3.80 (0.00, 9.01) | -6.58  (-7.03, -6.12) | <0.05 |
| South Asia | 388,993.59 (175,357.47, 602,629.71) | 931,668.08 (278,486.45, 1,584,849.71) | 35.79 (16.06, 55.51) | 53.46 (17.27, 89.65) | 1.34  (1.18, 1.49) | <0.05 |
| Central Sub-Saharan Africa | 87,188.92 (0.00, 187,616.26) | 36,849.74 (0.00, 76,694.60) | 141.22 (0.00, 299.00) | 24.01 (0.00, 50.02) | -5.15  (-6.00, -4.29) | <0.05 |
| Eastern Sub-Saharan Africa | 242,217.67 (21,947.48, 462,487.87) | 98,954.98 (4349.46, 193,560.49) | 111.72 (13.23, 210.21) | 19.96 (1.17, 38.75) | -5.42  (-5.70, -5.14) | <0.05 |
| Southern Sub-Saharan Africa | 8.58 (0.00, 33.12) | 12.28 (0.00, 51.40) | 0.02 (0.00, 0.06) | 0.01 (0.00, 0.06) | -0.49  (-1.68, 0.72) | 0.43 |
| Western Sub-Saharan Africa | 530,228.70 (70,013.93, 990,443.47) | 185,150.35 (41,888.40, 328,412.31) | 241.79 (29.33, 454.25) | 31.89 (7.99, 55.80) | -5.36  (-5.73, -4.99) | <0.05 |

DALYs disability-adjusted life-years, ASDRs age-standardized DALYs rates, AAPCs average annual percentage changes, UI uncertainty intervals, CI confidence interval, SDI Socio-demographic Index

**Table B** DALYs, ASDRs in 1990 and 2021, and AAPCs from 1990 to 2021 for arthropod-borne diseases other than malaria in global and 21 regions

| **Location** | **1990 DALYs** | **2021 DALYs** | **1990 ASDRs**  **(1/100,000, 95% UI)** | **2021 ASDRs**  **(1/100,000, 95% UI)** | **AAPCs**  **(95% CI)** | ***P* Value** |
| --- | --- | --- | --- | --- | --- | --- |
| Global | 13,860,960.33 (7,172,488.68, 20,549,431.99) | 6,053,765.49 (4,515,196.00, 7,592,334.98) | 253.59 (139.28, 367.91) | 78.16 (58.06, 98.26) | -3.74  (-4.15, -3.33) | <0.05 |
| High SDI | 15,656.67 (0.00, 32,648.32) | 20,044.68 (11,206.54, 28,882.82) | 1.84 (0.00, 4.17) | 1.68 (0.91, 2.45) | -0.16  (-0.50, 0.19) | 0.36 |
| High-middle SDI | 246,692.92 (77,983.59, 415,402.25) | 177,258.06 (126,519.65, 227,996.47) | 24.21 (7.60, 40.83) | 16.17 (11.15, 21.20) | -1.31  (-1.59, -1.03) | <0.05 |
| Middle SDI | 2,395,945.13 (1,694,427.50, 3,097,462.75) | 1,783,921.35 (1,247,642.34, 2,320,200.35) | 146.98 (108.21, 185.75) | 78.00 (54.23, 101.76) | -2.01  (-2.26, -1.75) | <0.05 |
| Low-middle SDI | 4,235,093.66 (2,198,478.56, 6,271,708.76) | 1,931,511.33 (1,301,164.39, 2,561,858.26) | 383.32 (234.69, 531.94) | 104.67 (71.59, 137.75) | -4.03  (-4.75, -3.31) | <0.05 |
| Low SDI | 6,958,911.94 (3,014,676.50, 10,903,147.39) | 2,137,258.57 (1,529,356.13, 2,745,161.01) | 1346.62 (719.95, 1973.29) | 210.92 (151.60, 270.24) | -5.64  (-6.05, -5.23) | <0.05 |
| East Asia | 67,214.09 (0.00, 240,445.09) | 11,181.97 (0.00, 35,239.21) | 5.54 (0.00, 19.58) | 0.91 (0.00, 3.08) | -5.75  (-6.24, -5.25) | <0.05 |
| Southeast Asia | 1,686,867.82 (1,194,017.90, 2,179,717.74) | 1,109,094.59 (762,468.39, 1,455,720.78) | 360.53 (259.73, 461.33) | 174.97 (119.69, 230.25) | -2.32  (-3.05, -1.58) | <0.05 |
| Oceania | 34,051.91 (22,022.75, 46,081.07) | 20,743.23 (12,615.52, 28,870.94) | 620.95 (398.03, 843.87) | 169.55 (104.50, 234.60) | -4.11  (-4.36, -3.87) | <0.05 |
| Central Asia | 9950.64 (2733.64, 17,167.63) | 8870.79 (0.00, 20,295.86) | 15.47 (5.82, 25.13) | 9.43 (0.00, 21.21) | -1.63  (-1.75, -1.50) | <0.05 |
| Central Europe | 9269.18 (0.00, 53,200.39) | 385.72 (0.00, 1851.83) | 8.47 (0.00, 48.72) | 0.43 (0.00, 2.31) | -9.04  (-9.99, -8.07) | <0.05 |
| Eastern Europe | 0.00 (0.00, 0.00) | 0.00 (0.00, 0.00) | 0.00 (0.00, 0.00) | 0.00 (0.00, 0.00) | NA | NA |
| High-income Asia Pacific | 2288.05 (0.00, 5115.43) | 4913.66 (0.00, 10,498.78) | 1.37 (0.00, 3.05) | 3.02 (0.00, 6.45) | 2.51  (1.83, 3.21) | <0.05 |
| Australasia | 85.05 (0.00, 183.88) | 210.01 (0.00, 434.38) | 0.41 (0.00, 0.90) | 0.66 (0.00, 1.38) | 1.52  (0.76, 2.28) | <0.05 |
| Western Europe | 5083.24 (0.00, 20,430.96) | 2113.79 (0.00, 5970.85) | 1.62 (0.00, 6.83) | 0.56 (0.00, 1.84) | -3.35  (-4.15, -2.55) | <0.05 |
| Southern Latin America | 48,497.89 (33,822.78, 63,173.01) | 22,768.41 (16,508.59, 29,028.22) | 103.55 (73.50, 133.61) | 28.74 (20.38, 37.09) | -4.06  (-4.15, -3.97) | <0.05 |
| High-income North America | 2018.90 (1209.33, 2828.47) | 2714.50 (1618.43, 3810.57) | 0.62 (0.37, 0.87) | 0.54 (0.32, 0.77) | -0.38  (-0.58, -0.18) | <0.05 |
| Caribbean | 40,907.28 (27,412.24, 54,402.32) | 14,671.32 (8512.06, 20,830.59) | 120.22 (80.85, 159.60) | 30.66 (17.64, 43.68) | -4.21  (-5.23, -3.19) | <0.05 |
| Andean Latin America | 56,857.79 (32,523.40, 81,192.19) | 48,330.84 (36,927.93, 59,733.74) | 193.71 (131.57, 255.86) | 75.62 (57.85, 93.38) | -2.92  (-3.09, -2.75) | <0.05 |
| Central Latin America | 100,776.72 (76,731.43, 124,822.01) | 124,799.03 (96,541.66, 153,056.40) | 85.39 (68.86, 101.91) | 49.64 (38.26, 61.01) | -1.72  (-2.28, -1.14) | <0.05 |
| Tropical Latin America | 488,192.03 (307,954.04, 668,430.02) | 413,638.77 (231,231.85, 596,045.69) | 403.49 (295.36, 511.62) | 176.48 (90.32, 262.64) | -2.66  (-3.14, -2.18) | <0.05 |
| North Africa and Middle East | 526,441.79 (0.00, 1,588,839.34) | 321,795.14 (156,768.68, 486,821.60) | 147.09 (0.00, 397.07) | 52.90 (26.58, 79.22) | -3.28  (-3.81, -2.74) | <0.05 |
| South Asia | 4,397,840.07 (0.00, 9,098,211.06) | 1,773,687.48 (970,671.98, 2,576,702.97) | 404.25 (49.12, 759.39) | 98.97 (54.95, 142.98) | -4.40  (-4.74, -4.06) | <0.05 |
| Central Sub-Saharan Africa | 1,543,598.39 (1,117,701.04, 1,969,495.74) | 831,009.71 (527,664.64, 1,134,354.79) | 3126.97 (2248.29, 4005.65) | 732.16 (470.55, 993.77) | -4.58  (-5.19, -3.97) | <0.05 |
| Eastern Sub-Saharan Africa | 3,021,857.59 (2,242,897.33, 3,800,817.84) | 549,771.36 (383,051.17, 716,491.55) | 1423.80 (1095.39, 1752.21) | 136.29 (95.23, 177.35) | -7.35  (-7.99, -6.72) | <0.05 |
| Southern Sub-Saharan Africa | 3320.44 (1391.14, 5249.74) | 4223.05 (1743.30, 6702.79) | 6.46 (2.68, 10.24) | 5.11 (2.13, 8.08) | -1.00  (-1.55, -0.45) | <0.05 |
| Western Sub-Saharan Africa | 1,815,841.45 (1,219,965.79, 2,411,717.11) | 788,842.13 (526,904.60, 1,050,779.66) | 1087.97 (763.92, 1412.03) | 189.13 (128.44, 249.83) | -5.47  (-6.21, -4.73) | <0.05 |

DALYs disability-adjusted life-years, ASDRs age-standardized DALYs rates, AAPCs average annual percentage changes, UI uncertainty intervals, CI confidence interval, SDI Socio-demographic Index

**Table C** Prevalence, ASPRs in 1990 and 2021, and AAPC from 1990 to 2021 for nine arthropod-borne diseases in global and 21 regions

| **Location** | **1990 Number** | **2021 Number** | **1990 ASPRs**  **(1/100,000, 95% UI)** | **2021 ASPRs**  **(1/100,000, 95% UI)** | **AAPCs**  **(95% CI)** | ***P* Value** |
| --- | --- | --- | --- | --- | --- | --- |
| **Malaria** |  |  |  |  |  |  |
| Global | 159,481,292.20 (148,604,988.68, 175,161,169.45) | 173,885,570.15 (157,990,967.38, 194,458,400.29) | 2797.57 (2605.87, 3076.72) | 2336.79 (2122.92, 2612.21) | -0.58  (-0.69, -0.47) | <0.05 |
| High SDI | 38,365.23 (30,418.43, 47,836.26) | 11,488.80 (6887.05, 18,400.22) | 4.76 (3.79, 5.92) | 1.09 (0.66, 1.74) | -4.48  (-9.00, 0.27) | 0.064 |
| High-middle SDI | 615,151.71 (411,238.21, 1,128,079.79) | 120,594.21 (79,291.57, 185,409.34) | 58.65 (39.29, 107.67) | 11.03 (7.25, 17.01) | -5.34  (-6.48, -4.20) | <0.05 |
| Middle SDI | 15,713,658.31 (13,669,606.32, 18,841,652.21) | 12,413,998.10 (8,589,655.59, 17,691,132.12) | 869.05 (755.68, 1042.59) | 552.32 (380.01, 789.51) | -1.34  (-1.64, -1.05) | <0.05 |
| Low-middle SDI | 47,826,096.19 (42,026,592.48, 56,641,961.02) | 43,604,448.55 (36,115,327.71, 50,598,052.60) | 3754.00 (3294.93, 4457.31) | 2188.76 (1813.34, 2539.43) | -1.80  (-1.97, -1.64) | <0.05 |
| Low SDI | 95,190,448.88 (87,906,963.81, 104,367,527.73) | 117,659,303.16 (105,676,893.26, 130,159,532.39) | 17,155.37 (15,844.07, 18,797.53) | 9458.61 (8494.21, 10,473.82) | -1.92  (-2.01, -1.82) | <0.05 |
| East Asia | 787,610.70 (445,839.35, 961,471.84) | 31,885.02 (28,396.57, 35,605.67) | 63.85 (36.02, 78.01) | 2.44 (2.17, 2.72) | -9.99  (-11.60, -8.35) | <0.05 |
| Southeast Asia | 4,537,752.14 (3,690,289.40, 6,094,724.20) | 1,704,659.68 (1,211,199.29, 1,951,376.15) | 918.41 (750.52, 1229.00) | 248.30 (176.01, 284.11) | -4.15  (-4.86, -3.43) | <0.05 |
| Oceania | 906,620.32 (401,713.18, 2,533,467.44) | 830,376.38 (745,698.53, 921,833.19) | 12,770.76 (5653.69, 35,627.68) | 5622.34 (5046.97, 6242.96) | -2.51  (-3.65, -1.36) | <0.05 |
| Central Asia | 24,646.83 (9335.14, 67,965.43) | 0.00 (0.00, 0.00) | 32.97 (12.65, 90.42) | 0.00 (0.00, 0.00) | NA | NA |
| Central Europe | 0.00 (0.00, 0.00) | 0.00 (0.00, 0.00) | 0.00 (0.00, 0.00) | 0.00 (0.00, 0.00) | NA | NA |
| Eastern Europe | 0.00 (0.00, 0.00) | 0.00 (0.00, 0.00) | 0.00 (0.00, 0.00) | 0.00 (0.00, 0.00) | NA | NA |
| High-income Asia Pacific | 21,348.07 (14,641.27, 30,073.61) | 10,469.25 (5898.16, 17,478.91) | 13.23 (9.02, 18.71) | 6.57 (3.70, 10.97) | -6.56  (NA, NA) | NA |
| Australasia | 0.00 (0.00, 0.00) | 0.00 (0.00, 0.00) | 0.00 (0.00, 0.00) | 0.00 (0.00, 0.00) | NA | NA |
| Western Europe | 0.00 (0.00, 0.00) | 0.00 (0.00, 0.00) | 0.00 (0.00, 0.00) | 0.00 (0.00, 0.00) | NA | NA |
| Southern Latin America | 22,714.23 (22,370.31, 23,065.19) | 0.00 (0.00, 0.00) | 45.44 (44.75, 46.14) | 0.00 (0.00, 0.00) | NA | NA |
| High-income North America | 0.00 (0.00, 0.00) | 0.00 (0.00, 0.00) | 0.00 (0.00, 0.00) | 0.00 (0.00, 0.00) | NA | NA |
| Caribbean | 69,732.20 (55,442.33, 109,895.89) | 65,303.62 (37,392.52, 129,234.08) | 187.12 (148.53, 297.70) | 144.46 (82.28, 287.20) | -0.61  (-3.28, 2.13) | 0.659 |
| Andean Latin America | 539,064.42 (362,464.36, 1,030,029.02) | 159,180.09 (57,045.76, 205,797.93) | 1333.04 (895.30, 2551.24) | 240.96 (86.31, 311.50) | -5.23  (-5.80, -4.67) | <0.05 |
| Central Latin America | 833,613.74 (791,349.79, 870,755.85) | 507,785.28 (392,898.21, 649,040.07) | 472.44 (448.19, 493.77) | 203.49 (156.73, 260.55) | -2.46  (-3.38, -1.54) | <0.05 |
| Tropical Latin America | 798,854.43 (688,783.36, 924,330.99) | 271,433.74 (199,857.53, 361,295.01) | 484.74 (418.88, 560.83) | 126.60 (92.69, 168.10) | -3.88  (-4.54, -3.21) | <0.05 |
| North Africa and Middle East | 2,429,301.19 (1,767,132.39, 3,729,701.25) | 2,867,996.48 (1,962,984.98, 4,292,673.59) | 647.25 (474.66, 979.72) | 450.32 (308.20, 674.13) | -1.11  (-1.78, -0.43) | <0.05 |
| South Asia | 16,969,511.22 (11,768,041.18, 28,307,421.84) | 5,970,785.23 (4,920,191.90, 7,803,469.05) | 1453.30 (1007.44, 2430.45) | 321.31 (264.80, 419.90) | -5.72  (-7.59, -3.80) | <0.05 |
| Central Sub-Saharan Africa | 22,911,353.64 (20,131,469.89, 25,732,342.05) | 31,617,345.06 (23,854,192.23, 42,362,284.59) | 38,056.50 (33,491.80, 42,691.76) | 21,328.79 (16,092.29, 28,557.44) | -1.89  (-2.49, -1.30) | <0.05 |
| Eastern Sub-Saharan Africa | 38,001,013.65 (35,198,086.41, 41,807,152.52) | 34,555,455.84 (29,688,749.23, 39,078,451.77) | 18,142.20 (16,791.96, 19,958.63) | 7386.25 (6348.66, 8353.98) | -2.85  (-3.08, -2.61) | <0.05 |
| Southern Sub-Saharan Africa | 313,009.81 (223,632.82, 515,737.50) | 199,122.66 (151,753.76, 258,891.59) | 534.74 (380.28, 887.49) | 239.74 (182.67, 311.47) | -1.88  (-6.37, 2.82) | 0.426 |
| Western Sub-Saharan Africa | 70,315,145.62 (63,353,399.04, 79,211,438.10) | 95,093,771.82 (78,815,387.69, 110,970,343.25) | 33,437.33 (30,122.49, 37,563.47) | 17,793.05 (14,749.24, 20,773.12) | -2.09  (-2.26, -1.92) | <0.05 |
| **Chagas disease** |  |  |  |  |  |  |
| Global | 7,070,826.69 (6,062,941.49, 8,167,747.08) | 6,297,840.66 (5,422,631.74, 7,206,339.44) | 145.19 (125.35, 168.24) | 75.62 (65.15, 86.52) | -2.09  (-2.18, -1.99) | <0.05 |
| High SDI | 191,169.72 (160,525.49, 226,366.23) | 263,538.94 (221,095.99, 308,596.15) | 19.05 (16.00, 22.59) | 19.49 (16.20, 22.89) | 0.07  (-0.09, 0.23) | 0.409 |
| High-middle SDI | 1,948,504.49 (1,712,210.31, 2,175,243.03) | 1,103,481.40 (963,170.81, 1,259,426.31) | 181.69 (159.63, 202.52) | 72.07 (62.86, 82.39) | -2.92  (-3.21, -2.63) | <0.05 |
| Middle SDI | 3,024,791.45 (2,534,021.18, 3,594,784.67) | 2,994,996.13 (2,532,438.05, 3,475,122.16) | 211.23 (178.98, 249.43) | 112.80 (95.39, 131.09) | -2.00  (-2.05, -1.94) | <0.05 |
| Low-middle SDI | 1,906,186.42 (1,640,825.08, 2,227,874.21) | 1,935,660.44 (1,685,550.39, 2,189,988.06) | 211.71 (183.44, 248.51) | 109.86 (96.01, 123.89) | -2.10  (-2.19, -2.01) | <0.05 |
| Low SDI | 0.00 (0.00, 0.00) | 0.00 (0.00, 0.00) | 0.00 (0.00, 0.00) | 0.00 (0.00, 0.00) | NA | NA |
| East Asia | 0.00 (0.00, 0.00) | 0.00 (0.00, 0.00) | 0.00 (0.00, 0.00) | 0.00 (0.00, 0.00) | NA | NA |
| Southeast Asia | 0.00 (0.00, 0.00) | 0.00 (0.00, 0.00) | 0.00 (0.00, 0.00) | 0.00 (0.00, 0.00) | NA | NA |
| Oceania | 0.00 (0.00, 0.00) | 0.00 (0.00, 0.00) | 0.00 (0.00, 0.00) | 0.00 (0.00, 0.00) | NA | NA |
| Central Asia | 0.00 (0.00, 0.00) | 0.00 (0.00, 0.00) | 0.00 (0.00, 0.00) | 0.00 (0.00, 0.00) | NA | NA |
| Central Europe | 0.00 (0.00, 0.00) | 10.94 (8.54, 13.93) | 0.00 (0.00, 0.00) | 0.01 (0.01, 0.01) | NA | NA |
| Eastern Europe | 0.00 (0.00, 0.00) | 0.00 (0.00, 0.00) | 0.00 (0.00, 0.00) | 0.00 (0.00, 0.00) | NA | NA |
| High-income Asia Pacific | 1660.97 (1426.41, 1937.43) | 5587.94 (4835.76, 6440.66) | 0.84 (0.73, 0.99) | 2.47 (2.10, 2.86) | 3.61  (3.31, 3.91) | <0.05 |
| Australasia | 2429.40 (2127.15, 2754.11) | 1698.31 (1433.00, 1994.82) | 11.05 (9.68, 12.52) | 4.62 (3.89, 5.41) | -2.76  (-2.83, -2.69) | <0.05 |
| Western Europe | 17,122.82 (14,912.09, 19,627.06) | 43,595.19 (37,958.27, 49,431.47) | 3.80 (3.31, 4.37) | 8.37 (7.25, 9.51) | 2.57  (2.21, 2.93) | <0.05 |
| Southern Latin America | 1,914,060.30 (1,682,962.36, 2,136,121.28) | 1,023,447.63 (890,660.93, 1,170,380.92) | 3944.84 (3466.54, 4402.68) | 1346.14 (1176.08, 1538.41) | -3.38  (-3.63, -3.13) | <0.05 |
| High-income North America | 178,640.15 (149,542.60, 212,061.46) | 246,107.70 (205,725.58, 289,035.79) | 56.92 (47.63, 67.62) | 55.86 (46.45, 65.70) | -0.05  (-0.21, 0.12) | 0.593 |
| Caribbean | 1935.14 (1660.48, 2245.82) | 1916.56 (1646.45, 2211.74) | 6.75 (5.86, 7.80) | 3.73 (3.20, 4.30) | -1.91  (-2.01, -1.80) | <0.05 |
| Andean Latin America | 1,039,154.67 (903,019.46, 1,204,892.61) | 810,181.13 (720,545.01, 901,577.04) | 3328.06 (2887.54, 3875.36) | 1224.55 (1089.32, 1362.42) | -3.19  (-3.33, -3.04) | <0.05 |
| Central Latin America | 1,894,445.84 (1,575,814.15, 2,269,046.05) | 2,074,658.13 (1,738,420.81, 2,416,133.87) | 1516.23 (1277.16, 1810.20) | 791.03 (663.29, 920.24) | -2.07  (-2.14, -1.99) | <0.05 |
| Tropical Latin America | 2,021,377.41 (1,708,160.80, 2,366,909.18) | 2,090,637.11 (1,799,849.68, 2,384,371.94) | 1602.36 (1368.23, 1868.66) | 830.76 (715.10, 945.92) | -2.09  (-2.12, -2.06) | <0.05 |
| North Africa and Middle East | 0.00 (0.00, 0.00) | 0.00 (0.00, 0.00) | 0.00 (0.00, 0.00) | 0.00 (0.00, 0.00) | NA | NA |
| South Asia | 0.00 (0.00, 0.00) | 0.00 (0.00, 0.00) | 0.00 (0.00, 0.00) | 0.00 (0.00, 0.00) | NA | NA |
| Central Sub-Saharan Africa | 0.00 (0.00, 0.00) | 0.00 (0.00, 0.00) | 0.00 (0.00, 0.00) | 0.00 (0.00, 0.00) | NA | NA |
| Eastern Sub-Saharan Africa | 0.00 (0.00, 0.00) | 0.00 (0.00, 0.00) | 0.00 (0.00, 0.00) | 0.00 (0.00, 0.00) | NA | NA |
| Southern Sub-Saharan Africa | 0.00 (0.00, 0.00) | 0.00 (0.00, 0.00) | 0.00 (0.00, 0.00) | 0.00 (0.00, 0.00) | NA | NA |
| Western Sub-Saharan Africa | 0.00 (0.00, 0.00) | 0.00 (0.00, 0.00) | 0.00 (0.00, 0.00) | 0.00 (0.00, 0.00) | NA | NA |
| **Leishmaniasis** |  |  |  |  |  |  |
| Global | 3,184,236.00 (2,658,032.49, 3,883,765.63) | 6,209,395.69 (5,825,066.39, 6,694,050.93) | 62.94 (52.92, 76.63) | 76.96 (71.89, 82.99) | 0.64  (0.59, 0.68) | <0.05 |
| High SDI | 104,688.19 (44,756.66, 208,182.64) | 165,816.85 (119,646.02, 236,689.08) | 11.79 (4.99, 23.45) | 13.93 (10.40, 19.31) | 0.54  (0.50, 0.57) | <0.05 |
| High-middle SDI | 127,128.20 (73,540.76, 224,189.72) | 172,416.79 (139,434.37, 227,240.67) | 11.96 (6.93, 21.06) | 11.91 (10.00, 14.92) | -0.02  (-0.07, 0.04) | 0.571 |
| Middle SDI | 1,273,348.63 (955,701.25, 1,762,633.30) | 2,691,876.49 (2,506,744.75, 2,975,572.44) | 84.02 (63.27, 116.97) | 106.40 (99.00, 117.23) | 0.76  (0.66, 0.87) | <0.05 |
| Low-middle SDI | 1,285,310.89 (964,356.71, 1,851,904.19) | 1,756,324.71 (1,567,186.67, 2,023,530.22) | 136.16 (102.60, 192.53) | 99.68 (88.71, 115.88) | -1.01  (-1.03, -0.98) | <0.05 |
| Low SDI | 392,118.66 (276,494.53, 573,569.66) | 1,418,428.04 (1,282,333.99, 1,567,000.18) | 98.34 (66.25, 146.40) | 139.66 (125.52, 156.55) | 1.16  (1.09, 1.23) | <0.05 |
| East Asia | 27,416.64 (12,029.75, 65,837.00) | 20,540.30 (10,591.78, 44,225.60) | 2.43 (1.08, 5.72) | 1.07 (0.59, 2.20) | -2.62  (-2.65, -2.58) | <0.05 |
| Southeast Asia | 4064.58 (2035.68, 7308.44) | 14,510.27 (12,369.95, 16,710.44) | 1.10 (0.55, 1.96) | 2.10 (1.79, 2.43) | 2.09  (1.98, 2.21) | <0.05 |
| Oceania | 0.00 (0.00, 0.00) | 0.00 (0.00, 0.00) | 0.00 (0.00, 0.00) | 0.00 (0.00, 0.00) | NA | NA |
| Central Asia | 135,942.02 (103,471.39, 180,370.45) | 103,350.24 (86,278.08, 124,641.14) | 218.92 (168.59, 288.81) | 111.87 (93.49, 135.47) | -2.16  (-2.19, -2.12) | <0.05 |
| Central Europe | 1760.61 (1232.33, 2556.82) | 1021.47 (840.69, 1295.20) | 1.36 (0.97, 1.94) | 0.67 (0.55, 0.83) | -2.27  (-2.33, -2.20) | <0.05 |
| Eastern Europe | 0.00 (0.00, 0.00) | 0.00 (0.00, 0.00) | 0.00 (0.00, 0.00) | 0.00 (0.00, 0.00) | NA | NA |
| High-income Asia Pacific | 0.00 (0.00, 0.00) | 0.00 (0.00, 0.00) | 0.00 (0.00, 0.00) | 0.00 (0.00, 0.00) | NA | NA |
| Australasia | 0.00 (0.00, 0.00) | 0.00 (0.00, 0.00) | 0.00 (0.00, 0.00) | 0.00 (0.00, 0.00) | NA | NA |
| Western Europe | 4242.54 (2535.91, 7161.54) | 4138.68 (3223.99, 5622.11) | 0.99 (0.60, 1.66) | 0.75 (0.60, 0.97) | -0.86  (-0.93, -0.80) | <0.05 |
| Southern Latin America | 6265.14 (2479.92, 13,786.93) | 9481.11 (7454.77, 13,116.51) | 13.02 (5.16, 28.55) | 12.85 (10.32, 17.45) | -0.02  (-0.11, 0.06) | 0.572 |
| High-income North America | 266.16 (62.37, 695.27) | 412.04 (254.43, 692.05) | 0.09 (0.02, 0.23) | 0.10 (0.06, 0.16) | 0.34  (0.31, 0.37) | <0.05 |
| Caribbean | 18,121.17 (9853.10, 31,602.36) | 26,183.02 (21,334.12, 33,100.83) | 55.31 (30.04, 97.56) | 52.92 (43.21, 66.17) | -0.13  (-0.17, -0.09) | <0.05 |
| Andean Latin America | 172,242.47 (121,746.85, 246,948.30) | 357,493.90 (317,878.18, 403,132.27) | 551.91 (389.38, 778.49) | 548.72 (487.32, 619.84) | -0.03  (-0.05, -0.00) | <0.05 |
| Central Latin America | 321,445.61 (228,286.64, 458,657.36) | 552,350.13 (494,120.90, 640,115.64) | 241.10 (170.49, 351.89) | 215.86 (193.17, 249.71) | -0.37  (-0.41, -0.32) | <0.05 |
| Tropical Latin America | 409,733.53 (312,997.08, 529,906.88) | 736,074.70 (667,661.71, 817,839.63) | 302.62 (234.49, 390.31) | 297.45 (269.82, 330.72) | -0.07  (-0.13, -0.02) | <0.05 |
| North Africa and Middle East | 1,741,350.95 (1,285,782.66, 2,366,455.00) | 3,831,680.48 (3,531,869.99, 4,202,149.36) | 645.66 (480.84, 883.73) | 642.90 (592.06, 712.93) | -0.01  (-0.08, 0.06) | 0.767 |
| South Asia | 227,062.11 (151,552.04, 324,908.98) | 342,973.67 (292,951.54, 406,609.23) | 23.78 (15.20, 35.41) | 18.86 (16.21, 22.61) | -0.72  (-0.90, -0.52) | <0.05 |
| Central Sub-Saharan Africa | 13,596.87 (6531.69, 25,788.85) | 17,412.82 (13,745.81, 22,288.78) | 27.28 (13.47, 50.12) | 16.16 (12.26, 22.26) | -1.68  (-1.83, -1.52) | <0.05 |
| Eastern Sub-Saharan Africa | 49,991.96 (33,223.56, 71,156.35) | 36,553.02 (31,755.60, 42,207.78) | 25.76 (17.05, 36.83) | 11.00 (9.20, 13.59) | -2.69  (-2.74, -2.64) | <0.05 |
| Southern Sub-Saharan Africa | 309.57 (132.81, 602.04) | 497.51 (390.40, 640.20) | 0.73 (0.31, 1.45) | 0.66 (0.50, 0.87) | -0.32  (-0.35, -0.29) | <0.05 |
| Western Sub-Saharan Africa | 50,424.07 (31,213.37, 77,806.14) | 154,722.32 (137,889.55, 173,270.84) | 35.65 (22.12, 55.13) | 41.88 (36.63, 48.37) | 0.53  (0.45, 0.61) | <0.05 |
| **African trypanosomiasis** |  |  |  |  |  |  |
| Global | 61,737.74 (27,640.45, 118,259.55) | 2367.93 (1130.44, 4391.73) | 1.17 (0.52, 2.23) | 0.03 (0.01, 0.06) | -11.37  (-12.23, -10.51) | <0.05 |
| High SDI | 0.00 (0.00, 0.00) | 0.00 (0.00, 0.00) | 0.00 (0.00, 0.00) | 0.00 (0.00, 0.00) | NA | NA |
| High-middle SDI | 0.00 (0.00, 0.00) | 0.00 (0.00, 0.00) | 0.00 (0.00, 0.00) | 0.00 (0.00, 0.00) | NA | NA |
| Middle SDI | 630.86 (253.14, 1307.92) | 191.26 (72.66, 392.53) | 0.04 (0.02, 0.08) | 0.01 (0.00, 0.02) | -5.17  (-5.83, -4.50) | <0.05 |
| Low-middle SDI | 14,010.36 (6286.18, 26,197.36) | 701.62 (359.08, 1214.95) | 1.34 (0.60, 2.51) | 0.04 (0.02, 0.06) | -10.99  (-12.27, -9.69) | <0.05 |
| Low SDI | 47,085.87 (20,961.93, 92,242.79) | 1474.69 (698.36, 2769.68) | 11.36 (5.05, 22.27) | 0.15 (0.07, 0.29) | -13.11  (-14.02, -12.20) | <0.05 |
| East Asia | 0.00 (0.00, 0.00) | 0.00 (0.00, 0.00) | 0.00 (0.00, 0.00) | 0.00 (0.00, 0.00) | NA | NA |
| Southeast Asia | 0.00 (0.00, 0.00) | 0.00 (0.00, 0.00) | 0.00 (0.00, 0.00) | 0.00 (0.00, 0.00) | NA | NA |
| Oceania | 0.00 (0.00, 0.00) | 0.00 (0.00, 0.00) | 0.00 (0.00, 0.00) | 0.00 (0.00, 0.00) | NA | NA |
| Central Asia | 0.00 (0.00, 0.00) | 0.00 (0.00, 0.00) | 0.00 (0.00, 0.00) | 0.00 (0.00, 0.00) | NA | NA |
| Central Europe | 0.00 (0.00, 0.00) | 0.00 (0.00, 0.00) | 0.00 (0.00, 0.00) | 0.00 (0.00, 0.00) | NA | NA |
| Eastern Europe | 0.00 (0.00, 0.00) | 0.00 (0.00, 0.00) | 0.00 (0.00, 0.00) | 0.00 (0.00, 0.00) | NA | NA |
| High-income Asia Pacific | 0.00 (0.00, 0.00) | 0.00 (0.00, 0.00) | 0.00 (0.00, 0.00) | 0.00 (0.00, 0.00) | NA | NA |
| Australasia | 0.00 (0.00, 0.00) | 0.00 (0.00, 0.00) | 0.00 (0.00, 0.00) | 0.00 (0.00, 0.00) | NA | NA |
| Western Europe | 0.00 (0.00, 0.00) | 0.00 (0.00, 0.00) | 0.00 (0.00, 0.00) | 0.00 (0.00, 0.00) | NA | NA |
| Southern Latin America | 0.00 (0.00, 0.00) | 0.00 (0.00, 0.00) | 0.00 (0.00, 0.00) | 0.00 (0.00, 0.00) | NA | NA |
| High-income North America | 0.00 (0.00, 0.00) | 0.00 (0.00, 0.00) | 0.00 (0.00, 0.00) | 0.00 (0.00, 0.00) | NA | NA |
| Caribbean | 0.00 (0.00, 0.00) | 0.00 (0.00, 0.00) | 0.00 (0.00, 0.00) | 0.00 (0.00, 0.00) | NA | NA |
| Andean Latin America | 0.00 (0.00, 0.00) | 0.00 (0.00, 0.00) | 0.00 (0.00, 0.00) | 0.00 (0.00, 0.00) | NA | NA |
| Central Latin America | 0.00 (0.00, 0.00) | 0.00 (0.00, 0.00) | 0.00 (0.00, 0.00) | 0.00 (0.00, 0.00) | NA | NA |
| Tropical Latin America | 0.00 (0.00, 0.00) | 0.00 (0.00, 0.00) | 0.00 (0.00, 0.00) | 0.00 (0.00, 0.00) | NA | NA |
| North Africa and Middle East | 0.00 (0.00, 0.00) | 0.00 (0.00, 0.00) | 0.00 (0.00, 0.00) | 0.00 (0.00, 0.00) | NA | NA |
| South Asia | 0.00 (0.00, 0.00) | 0.00 (0.00, 0.00) | 0.00 (0.00, 0.00) | 0.00 (0.00, 0.00) | NA | NA |
| Central Sub-Saharan Africa | 41,285.11 (18,260.90, 80,124.96) | 1027.43 (524.09, 1805.51) | 94.38 (41.73, 183.22) | 0.93 (0.47, 1.63) | -13.93  (-14.45, -13.41) | <0.05 |
| Eastern Sub-Saharan Africa | 14,628.93 (6937.01, 27,231.86) | 404.65 (242.00, 654.76) | 9.69 (4.60, 18.04) | 0.11 (0.07, 0.18) | -13.38  (-14.46, -12.29) | <0.05 |
| Southern Sub-Saharan Africa | 0.00 (0.00, 0.00) | 0.00 (0.00, 0.00) | 0.00 (0.00, 0.00) | 0.00 (0.00, 0.00) | NA | NA |
| Western Sub-Saharan Africa | 5823.70 (2230.41, 11,946.85) | 935.85 (356.29, 1914.05) | 3.69 (1.41, 7.59) | 0.23 (0.09, 0.48) | -8.51  (-8.86, -8.17) | <0.05 |
| **Lymphatic filariasis** |  |  |  |  |  | 0 |
| Global | 209,062,177.57 (179,977,279.31, 248,835,158.93) | 56,902,555.80 (48,666,870.20, 67,914,348.40) | 4054.97 (3493.54, 4829.06) | 705.97 (603.68, 841.83) | -5.51  (-5.73, -5.28) | <0.05 |
| High SDI | 0.00 (0.00, 0.00) | 0.00 (0.00, 0.00) | 0.00 (0.00, 0.00) | 0.00 (0.00, 0.00) | NA | NA |
| High-middle SDI | 2,803,405.76 (1,328,524.86, 6,238,454.43) | 990,839.30 (486,070.51, 2,349,853.23) | 256.94 (121.61, 572.20) | 75.31 (37.79, 177.57) | -3.75  (-4.27, -3.23) | <0.05 |
| Middle SDI | 53,420,026.88 (37,525,032.70, 79,710,350.65) | 14,886,385.90 (11,828,205.01, 21,273,817.28) | 3346.18 (2336.31, 5033.76) | 579.98 (461.00, 826.49) | -5.49  (-5.94, -5.05) | <0.05 |
| Low-middle SDI | 95,329,998.83 (83,753,231.43, 110,553,787.17) | 25,505,048.31 (21,399,819.03, 30,991,947.03) | 9744.74 (8575.49, 11,330.64) | 1370.16 (1150.05, 1664.52) | -6.15  (-6.41, -5.89) | <0.05 |
| Low SDI | 57,240,439.56 (47,975,437.30, 68,094,105.94) | 15,449,342.10 (12,641,468.05, 19,521,387.37) | 14,389.38 (12,098.02, 17,028.63) | 1669.80 (1370.97, 2093.63) | -6.81  (-7.09, -6.53) | <0.05 |
| East Asia | 0.00 (0.00, 0.00) | 0.00 (0.00, 0.00) | 0.00 (0.00, 0.00) | 0.00 (0.00, 0.00) | NA | NA |
| Southeast Asia | 45,939,562.48 (24,693,428.25, 78,657,418.66) | 7,877,839.92 (3,890,013.43, 15,733,426.53) | 11,094.41 (5972.50, 18,901.61) | 1089.06 (539.69, 2167.24) | -7.23  (-7.94, -6.52) | <0.05 |
| Oceania | 1,812,261.23 (940,375.07, 2,924,906.23) | 948,179.16 (360,722.39, 2,268,683.24) | 33,028.44 (17,334.72, 52,695.86) | 7646.47 (2970.42, 18,140.42) | -4.39  (-5.16, -3.62) | <0.05 |
| Central Asia | 0.00 (0.00, 0.00) | 0.00 (0.00, 0.00) | 0.00 (0.00, 0.00) | 0.00 (0.00, 0.00) | NA | NA |
| Central Europe | 0.00 (0.00, 0.00) | 0.00 (0.00, 0.00) | 0.00 (0.00, 0.00) | 0.00 (0.00, 0.00) | NA | NA |
| Eastern Europe | 0.00 (0.00, 0.00) | 0.00 (0.00, 0.00) | 0.00 (0.00, 0.00) | 0.00 (0.00, 0.00) | NA | NA |
| High-income Asia Pacific | 4075.64 (647.94, 30,003.07) | 4487.44 (1037.83, 24,444.26) | 2.33 (0.39, 17.04) | 2.77 (0.70, 14.62) | 0.72  (0.10, 1.35) | <0.05 |
| Australasia | 0.00 (0.00, 0.00) | 0.00 (0.00, 0.00) | 0.00 (0.00, 0.00) | 0.00 (0.00, 0.00) | NA | NA |
| Western Europe | 0.00 (0.00, 0.00) | 0.00 (0.00, 0.00) | 0.00 (0.00, 0.00) | 0.00 (0.00, 0.00) | NA | NA |
| Southern Latin America | 0.00 (0.00, 0.00) | 0.00 (0.00, 0.00) | 0.00 (0.00, 0.00) | 0.00 (0.00, 0.00) | NA | NA |
| High-income North America | 0.00 (0.00, 0.00) | 0.00 (0.00, 0.00) | 0.00 (0.00, 0.00) | 0.00 (0.00, 0.00) | NA | NA |
| Caribbean | 2,061,281.56 (752,225.50, 4,595,643.41) | 415,467.85 (226,191.90, 805,439.38) | 6085.03 (2216.30, 13,581.98) | 851.78 (465.21, 1642.64) | -6.31  (-6.98, -5.65) | <0.05 |
| Andean Latin America | 0.00 (0.00, 0.00) | 0.00 (0.00, 0.00) | 0.00 (0.00, 0.00) | 0.00 (0.00, 0.00) | NA | NA |
| Central Latin America | 0.00 (0.00, 0.00) | 0.00 (0.00, 0.00) | 0.00 (0.00, 0.00) | 0.00 (0.00, 0.00) | NA | NA |
| Tropical Latin America | 258,791.09 (186,546.62, 485,474.48) | 18,260.04 (15,418.28, 31,865.92) | 185.25 (132.72, 350.34) | 7.94 (6.76, 13.55) | -9.69  (-10.35, -9.02) | <0.05 |
| North Africa and Middle East | 2,443,672.45 (677,223.76, 7,845,587.28) | 1,062,017.81 (305,385.04, 3,343,336.06) | 853.79 (233.66, 2755.12) | 170.36 (48.55, 537.52) | -5.06  (-6.42, -3.68) | <0.05 |
| South Asia | 103,391,426.77 (96,357,563.41, 111,190,113.59) | 34,671,952.04 (30,247,621.23, 40,389,153.81) | 11,084.20 (10,335.96, 11,905.18) | 1884.30 (1646.08, 2191.41) | -5.56  (-5.82, -5.30) | <0.05 |
| Central Sub-Saharan Africa | 6,403,695.86 (3,852,268.72, 10,098,640.12) | 2,270,255.54 (1,225,773.20, 4,312,847.14) | 14,837.02 (8930.28, 23,403.91) | 1989.86 (1060.32, 3782.61) | -6.24  (-6.41, -6.07) | <0.05 |
| Eastern Sub-Saharan Africa | 19,900,610.11 (14,219,971.03, 27,245,037.07) | 2,584,938.22 (1,760,794.63, 3,932,929.19) | 13,761.16 (9874.49, 18,730.79) | 702.62 (469.98, 1088.79) | -9.14  (-9.72, -8.55) | <0.05 |
| Southern Sub-Saharan Africa | 143,850.46 (38,987.22, 525,413.91) | 122,536.29 (42,972.12, 396,129.13) | 300.48 (77.98, 1111.19) | 150.93 (52.40, 490.52) | -2.06  (-2.69, -1.41) | <0.05 |
| Western Sub-Saharan Africa | 26,702,949.93 (17,405,665.61, 39,619,176.20) | 6,926,621.48 (4,443,188.42, 10,575,998.57) | 17,366.89 (11,288.48, 25,504.07) | 1729.07 (1105.91, 2662.41) | -7.21  (-7.94, -6.47) | <0.05 |
| **Onchocerciasis** |  |  |  |  |  |  |
| Global | 23,425,277.20 (21,549,626.55, 25,631,347.11) | 19,596,822.34 (17,757,021.33, 21,711,344.06) | 444.48 (409.37, 485.62) | 246.24 (222.68, 273.09) | -1.89  (-2.00, -1.78) | <0.05 |
| High SDI | 0.00 (0.00, 0.00) | 0.00 (0.00, 0.00) | 0.00 (0.00, 0.00) | 0.00 (0.00, 0.00) | NA | NA |
| High-middle SDI | 0.00 (0.00, 0.00) | 0.00 (0.00, 0.00) | 0.00 (0.00, 0.00) | 0.00 (0.00, 0.00) | NA | NA |
| Middle SDI | 1,335,663.06 (1,178,075.81, 1,535,809.28) | 641,158.98 (512,084.12, 793,334.34) | 80.84 (71.33, 92.79) | 25.76 (20.49, 31.95) | -3.64  (-3.71, -3.56) | <0.05 |
| Low-middle SDI | 4,828,011.73 (4,472,321.75, 5,284,046.78) | 2,379,006.14 (2,046,073.92, 2,746,472.49) | 466.06 (431.40, 510.28) | 124.70 (107.63, 143.60) | -4.18  (-4.28, -4.08) | <0.05 |
| Low SDI | 17,258,849.61 (15,880,107.41, 18,937,852.77) | 16,573,879.82 (15,113,703.00, 18,202,455.19) | 4242.83 (3924.27, 4629.09) | 1728.55 (1593.09, 1892.09) | -2.86  (-2.97, -2.74) | <0.05 |
| East Asia | 0.00 (0.00, 0.00) | 0.00 (0.00, 0.00) | 0.00 (0.00, 0.00) | 0.00 (0.00, 0.00) | NA | NA |
| Southeast Asia | 0.00 (0.00, 0.00) | 0.00 (0.00, 0.00) | 0.00 (0.00, 0.00) | 0.00 (0.00, 0.00) | NA | NA |
| Oceania | 0.00 (0.00, 0.00) | 0.00 (0.00, 0.00) | 0.00 (0.00, 0.00) | 0.00 (0.00, 0.00) | NA | NA |
| Central Asia | 0.00 (0.00, 0.00) | 0.00 (0.00, 0.00) | 0.00 (0.00, 0.00) | 0.00 (0.00, 0.00) | NA | NA |
| Central Europe | 0.00 (0.00, 0.00) | 0.00 (0.00, 0.00) | 0.00 (0.00, 0.00) | 0.00 (0.00, 0.00) | NA | NA |
| Eastern Europe | 0.00 (0.00, 0.00) | 0.00 (0.00, 0.00) | 0.00 (0.00, 0.00) | 0.00 (0.00, 0.00) | NA | NA |
| High-income Asia Pacific | 0.00 (0.00, 0.00) | 0.00 (0.00, 0.00) | 0.00 (0.00, 0.00) | 0.00 (0.00, 0.00) | NA | NA |
| Australasia | 0.00 (0.00, 0.00) | 0.00 (0.00, 0.00) | 0.00 (0.00, 0.00) | 0.00 (0.00, 0.00) | NA | NA |
| Western Europe | 0.00 (0.00, 0.00) | 0.00 (0.00, 0.00) | 0.00 (0.00, 0.00) | 0.00 (0.00, 0.00) | NA | NA |
| Southern Latin America | 0.00 (0.00, 0.00) | 0.00 (0.00, 0.00) | 0.00 (0.00, 0.00) | 0.00 (0.00, 0.00) | NA | NA |
| High-income North America | 0.00 (0.00, 0.00) | 0.00 (0.00, 0.00) | 0.00 (0.00, 0.00) | 0.00 (0.00, 0.00) | NA | NA |
| Caribbean | 0.00 (0.00, 0.00) | 0.00 (0.00, 0.00) | 0.00 (0.00, 0.00) | 0.00 (0.00, 0.00) | NA | NA |
| Andean Latin America | 16,876.35 (14,515.66, 18,688.21) | 0.00 (0.00, 0.00) | 48.21 (42.27, 53.06) | 0.00 (0.00, 0.00) | NA | NA |
| Central Latin America | 298,715.13 (269,090.90, 316,394.17) | 5040.13 (2324.77, 8740.14) | 193.61 (176.44, 203.83) | 1.93 (0.89, 3.38) | -13.80  (-14.38, -13.21) | <0.05 |
| Tropical Latin America | 21,348.23 (18,274.28, 23,566.44) | 219.67 (20.84, 934.75) | 13.66 (11.80, 14.98) | 0.10 (0.01, 0.41) | -14.82  (-15.50, -14.13) | <0.05 |
| North Africa and Middle East | 101,797.23 (91,038.52, 113,989.71) | 70,795.48 (64,301.58, 78,520.50) | 35.23 (31.54, 39.36) | 11.58 (10.51, 12.83) | -3.55  (-3.71, -3.39) | <0.05 |
| South Asia | 0.00 (0.00, 0.00) | 0.00 (0.00, 0.00) | 0.00 (0.00, 0.00) | 0.00 (0.00, 0.00) | NA | NA |
| Central Sub-Saharan Africa | 7,953,647.19 (7,457,145.39, 8,535,344.43) | 11,200,064.80 (10,468,183.54, 12,057,792.51) | 18,426.06 (17,260.60, 19,762.92) | 9857.70 (9222.91, 10,576.12) | -2.02  (-2.18, -1.87) | <0.05 |
| Eastern Sub-Saharan Africa | 3,228,824.06 (2,879,933.28, 3,647,959.18) | 3,043,682.37 (2,697,822.02, 3,437,058.40) | 2204.24 (1967.74, 2484.92) | 890.30 (801.35, 998.79) | -3.00  (-3.36, -2.63) | <0.05 |
| Southern Sub-Saharan Africa | 0.00 (0.00, 0.00) | 0.00 (0.00, 0.00) | 0.00 (0.00, 0.00) | 0.00 (0.00, 0.00) | NA | NA |
| Western Sub-Saharan Africa | 11,804,069.01 (10,693,505.56, 13,148,425.79) | 5,277,019.90 (4,395,464.04, 6,278,936.91) | 7585.89 (6921.44, 8395.96) | 1393.60 (1193.55, 1636.71) | -5.32  (-5.39, -5.26) | <0.05 |
| **Dengue** |  |  |  |  |  |  |
| Global | 1,577,834.03 (234,722.85, 3,169,006.62) | 3,517,383.51 (928,243.84, 6,430,039.49) | 28.75 (4.23, 57.77) | 44.86 (11.77, 82.13) | 1.45  (1.30, 1.60) | <0.05 |
| High SDI | 19,358.20 (2512.82, 49,165.77) | 35,973.25 (7568.48, 83,314.46) | 2.27 (0.29, 5.75) | 3.27 (0.69, 7.59) | 1.10  (0.58, 1.62) | <0.05 |
| High-middle SDI | 62,125.79 (7360.83, 172,339.65) | 147,982.26 (58,601.41, 266,671.52) | 5.84 (0.69, 16.18) | 12.79 (5.04, 23.03) | 2.61  (2.38, 2.84) | <0.05 |
| Middle SDI | 812,124.45 (52,358.85, 1,773,433.90) | 1,828,907.93 (616,234.54, 3,309,025.77) | 46.71 (3.05, 102.30) | 75.70 (25.59, 136.59) | 1.57  (1.46, 1.67) | <0.05 |
| Low-middle SDI | 555,210.01 (56,562.74, 1,176,068.66) | 1,269,642.71 (137,243.22, 2,706,195.61) | 47.95 (4.73, 101.66) | 66.77 (7.37, 141.34) | 1.07  (0.87, 1.26) | <0.05 |
| Low SDI | 128,324.51 (71,919.03, 193,957.32) | 233,663.38 (7365.56, 592,774.89) | 25.64 (14.03, 39.36) | 21.87 (0.68, 54.56) | -0.48  (-0.77, -0.18) | <0.05 |
| East Asia | 2123.51 (210.76, 6596.53) | 3636.85 (917.95, 9167.12) | 0.18 (0.02, 0.56) | 0.25 (0.06, 0.62) | 1.20  (0.83, 1.57) | <0.05 |
| Southeast Asia | 163,792.51 (31,413.69, 458,791.26) | 400,005.34 (281,600.57, 633,454.92) | 34.71 (6.68, 96.33) | 57.78 (40.63, 91.25) | 1.64  (1.46, 1.82) | <0.05 |
| Oceania | 1425.30 (314.41, 3750.28) | 3810.55 (1669.63, 7578.54) | 21.98 (4.85, 56.47) | 28.95 (12.35, 58.06) | 0.99  (0.71, 1.27) | <0.05 |
| Central Asia | 0.00 (0.00, 0.00) | 0.00 (0.00, 0.00) | 0.00 (0.00, 0.00) | 0.00 (0.00, 0.00) | NA | NA |
| Central Europe | 0.00 (0.00, 0.00) | 0.00 (0.00, 0.00) | 0.00 (0.00, 0.00) | 0.00 (0.00, 0.00) | NA | NA |
| Eastern Europe | 0.00 (0.00, 0.00) | 0.00 (0.00, 0.00) | 0.00 (0.00, 0.00) | 0.00 (0.00, 0.00) | NA | NA |
| High-income Asia Pacific | 13,085.25 (1800.19, 35,761.26) | 29,077.78 (5850.31, 71,641.82) | 7.77 (1.07, 21.24) | 17.60 (3.54, 43.36) | 2.58  (1.81, 3.35) | <0.05 |
| Australasia | 346.11 (23.69, 1107.71) | 1103.00 (304.16, 2901.32) | 1.71 (0.12, 5.49) | 3.53 (0.97, 9.25) | 2.44  (2.03, 2.86) | <0.05 |
| Western Europe | 0.00 (0.00, 0.00) | 0.00 (0.00, 0.00) | 0.00 (0.00, 0.00) | 0.00 (0.00, 0.00) | NA | NA |
| Southern Latin America | 2268.62 (44.52, 8031.45) | 4765.37 (1006.90, 11,400.92) | 4.59 (0.09, 16.23) | 7.07 (1.49, 16.92) | 1.42  (1.23, 1.62) | <0.05 |
| High-income North America | 17.16 (2.85, 58.22) | 80.19 (2.37, 402.08) | 0.01 (0.00, 0.02) | 0.02 (0.00, 0.10) | 4.01  (3.23, 4.80) | <0.05 |
| Caribbean | 8670.34 (267.94, 26,126.28) | 13,593.86 (1663.96, 38,336.69) | 24.79 (0.77, 74.65) | 28.49 (3.49, 80.01) | 0.38  (0.05, 0.70) | <0.05 |
| Andean Latin America | 7040.01 (802.26, 16,841.31) | 23,428.74 (9709.14, 41,697.57) | 18.32 (2.10, 43.68) | 35.48 (14.70, 63.09) | 2.24  (1.96, 2.51) | <0.05 |
| Central Latin America | 68,340.78 (11,324.26, 139,651.16) | 172,010.16 (102,575.61, 247,213.27) | 41.51 (6.89, 85.01) | 67.95 (40.54, 97.63) | 1.58  (0.17, 3.01) | <0.05 |
| Tropical Latin America | 408,763.48 (24,010.55, 1,056,403.99) | 776,465.03 (243,162.08, 1,587,514.78) | 265.61 (15.64, 691.39) | 343.77 (107.96, 700.31) | 0.93  (0.54, 1.32) | <0.05 |
| North Africa and Middle East | 1071.39 (357.98, 3423.54) | 3192.82 (1041.63, 11,472.72) | 0.31 (0.10, 1.00) | 0.51 (0.17, 1.83) | 1.14  (0.06, 2.23) | <0.05 |
| South Asia | 759,103.28 (7931.87, 1,648,564.32) | 1,901,418.05 (107,573.66, 4,067,252.56) | 69.58 (0.73, 150.95) | 103.22 (5.90, 220.59) | 1.31  (1.26, 1.36) | <0.05 |
| Central Sub-Saharan Africa | 4379.26 (53.05, 24,980.33) | 14,947.35 (676.95, 81,023.72) | 8.02 (0.10, 45.52) | 10.86 (0.50, 59.03) | 0.96  (0.82, 1.10) | <0.05 |
| Eastern Sub-Saharan Africa | 94,477.02 (7196.94, 255,561.15) | 22,952.73 (372.13, 88,355.21) | 49.41 (3.77, 133.14) | 5.59 (0.09, 21.71) | -6.92  (-7.60, -6.25) | <0.05 |
| Southern Sub-Saharan Africa | 41.63 (1.81, 282.71) | 63.29 (3.25, 428.08) | 0.07 (0.00, 0.50) | 0.08 (0.00, 0.52) | -0.50  (-1.59, 0.59) | 0.365 |
| Western Sub-Saharan Africa | 42,888.39 (26.10, 148,118.00) | 146,832.38 (7218.32, 530,938.28) | 22.50 (0.01, 78.76) | 30.38 (1.51, 111.89) | 1.05  (0.87, 1.22) | <0.05 |
| **Yellow fever** |  |  |  |  |  |  |
| Global | 9366.84 (3727.07, 20,300.19) | 2369.73 (919.90, 5044.51) | 0.17 (0.07, 0.36) | 0.03 (0.01, 0.07) | -4.76  (-5.10, -4.43) | <0.05 |
| High SDI | 0.00 (0.00, 0.00) | 0.00 (0.00, 0.00) | 0.00 (0.00, 0.00) | 0.00 (0.00, 0.00) | NA | NA |
| High-middle SDI | 76.82 (19.17, 244.31) | 19.70 (4.57, 59.64) | 0.01 (0.00, 0.02) | 0.00 (0.00, 0.01) | -4.23  (-4.47, -3.99) | <0.05 |
| Middle SDI | 879.20 (350.35, 1983.88) | 64.51 (24.98, 141.95) | 0.05 (0.02, 0.11) | 0.00 (0.00, 0.01) | -6.57  (-7.94, -5.19) | <0.05 |
| Low-middle SDI | 3427.67 (1219.92, 7701.76) | 502.09 (171.18, 1103.56) | 0.28 (0.10, 0.61) | 0.02 (0.01, 0.06) | -6.70  (-7.18, -6.21) | <0.05 |
| Low SDI | 4980.66 (2017.32, 11,200.78) | 1782.63 (704.89, 3853.85) | 0.95 (0.38, 2.15) | 0.15 (0.06, 0.32) | -5.83  (-6.38, -5.28) | <0.05 |
| East Asia | 0.00 (0.00, 0.00) | 0.00 (0.00, 0.00) | 0.00 (0.00, 0.00) | 0.00 (0.00, 0.00) | NA | NA |
| Southeast Asia | 0.00 (0.00, 0.00) | 0.00 (0.00, 0.00) | 0.00 (0.00, 0.00) | 0.00 (0.00, 0.00) | NA | NA |
| Oceania | 0.00 (0.00, 0.00) | 0.00 (0.00, 0.00) | 0.00 (0.00, 0.00) | 0.00 (0.00, 0.00) | NA | NA |
| Central Asia | 0.00 (0.00, 0.00) | 0.00 (0.00, 0.00) | 0.00 (0.00, 0.00) | 0.00 (0.00, 0.00) | NA | NA |
| Central Europe | 0.00 (0.00, 0.00) | 0.00 (0.00, 0.00) | 0.00 (0.00, 0.00) | 0.00 (0.00, 0.00) | NA | NA |
| Eastern Europe | 0.00 (0.00, 0.00) | 0.00 (0.00, 0.00) | 0.00 (0.00, 0.00) | 0.00 (0.00, 0.00) | NA | NA |
| High-income Asia Pacific | 0.00 (0.00, 0.00) | 0.00 (0.00, 0.00) | 0.00 (0.00, 0.00) | 0.00 (0.00, 0.00) | NA | NA |
| Australasia | 0.00 (0.00, 0.00) | 0.00 (0.00, 0.00) | 0.00 (0.00, 0.00) | 0.00 (0.00, 0.00) | NA | NA |
| Western Europe | 0.00 (0.00, 0.00) | 0.00 (0.00, 0.00) | 0.00 (0.00, 0.00) | 0.00 (0.00, 0.00) | NA | NA |
| Southern Latin America | 75.91 (18.62, 241.24) | 19.52 (4.50, 59.21) | 0.15 (0.04, 0.48) | 0.03 (0.01, 0.09) | -5.16  (-5.38, -4.94) | <0.05 |
| High-income North America | 0.00 (0.00, 0.00) | 0.00 (0.00, 0.00) | 0.00 (0.00, 0.00) | 0.00 (0.00, 0.00) | NA | NA |
| Caribbean | 5.46 (1.79, 13.92) | 1.04 (0.32, 2.58) | 0.01 (0.00, 0.04) | 0.00 (0.00, 0.01) | -5.80  (-6.18, -5.41) | <0.05 |
| Andean Latin America | 115.52 (39.82, 273.53) | 29.80 (9.42, 70.90) | 0.30 (0.10, 0.70) | 0.04 (0.01, 0.11) | -7.17  (-8.30, -6.03) | <0.05 |
| Central Latin America | 17.65 (6.07, 39.18) | 4.88 (1.64, 11.48) | 0.01 (0.00, 0.02) | 0.00 (0.00, 0.00) | -5.39  (-6.58, -4.19) | <0.05 |
| Tropical Latin America | 23.21 (9.36, 52.82) | 5.13 (1.99, 12.04) | 0.01 (0.01, 0.03) | 0.00 (0.00, 0.01) | -9.12  (-15.91, -1.79) | <0.05 |
| North Africa and Middle East | 1201.35 (315.33, 3816.83) | 181.40 (46.23, 488.00) | 0.34 (0.09, 1.03) | 0.03 (0.01, 0.08) | -8.17  (-8.43, -7.91) | <0.05 |
| South Asia | 0.00 (0.00, 0.00) | 0.00 (0.00, 0.00) | 0.00 (0.00, 0.00) | 0.00 (0.00, 0.00) | NA | NA |
| Central Sub-Saharan Africa | 881.15 (282.18, 2115.50) | 281.83 (94.38, 681.06) | 1.57 (0.52, 3.76) | 0.20 (0.06, 0.46) | -6.22  (-7.11, -5.32) | <0.05 |
| Eastern Sub-Saharan Africa | 2089.55 (783.68, 4939.85) | 667.42 (234.48, 1501.34) | 1.06 (0.41, 2.48) | 0.15 (0.05, 0.34) | -6.29  (-6.57, -6.02) | <0.05 |
| Southern Sub-Saharan Africa | 0.00 (0.00, 0.00) | 0.00 (0.00, 0.00) | 0.00 (0.00, 0.00) | 0.00 (0.00, 0.00) | NA | NA |
| Western Sub-Saharan Africa | 4957.03 (1938.80, 10,726.75) | 1178.70 (461.70, 2521.53) | 2.54 (0.99, 5.55) | 0.22 (0.09, 0.48) | -7.53  (-9.50, -5.52) | <0.05 |
| **Zika virus** |  |  |  |  |  |  |
| Global | 0.00 (0.00, 0.00) | 2935.00 (1172.35, 7212.99) | 0.00 (0.00, 0.00) | 0.04 (0.02, 0.09) | NA | NA |
| High SDI | 0.00 (0.00, 0.00) | 3.34 (0.47, 11.33) | 0.00 (0.00, 0.00) | 0.00 (0.00, 0.00) | NA | NA |
| High-middle SDI | 0.00 (0.00, 0.00) | 15.23 (4.52, 40.95) | 0.00 (0.00, 0.00) | 0.00 (0.00, 0.00) | NA | NA |
| Middle SDI | 0.00 (0.00, 0.00) | 1908.63 (656.79, 5465.11) | 0.00 (0.00, 0.00) | 0.08 (0.03, 0.22) | NA | NA |
| Low-middle SDI | 0.00 (0.00, 0.00) | 1003.52 (433.95, 2247.08) | 0.00 (0.00, 0.00) | 0.05 (0.02, 0.12) | NA | NA |
| Low SDI | 0.00 (0.00, 0.00) | 0.58 (0.08, 2.24) | 0.00 (0.00, 0.00) | 0.00 (0.00, 0.00) | NA | NA |
| East Asia | 0.00 (0.00, 0.00) | 0.00 (0.00, 0.00) | 0.00 (0.00, 0.00) | 0.00 (0.00, 0.00) | NA | NA |
| Southeast Asia | 0.00 (0.00, 0.00) | 0.00 (0.00, 0.00) | 0.00 (0.00, 0.00) | 0.00 (0.00, 0.00) | NA | NA |
| Oceania | 0.00 (0.00, 0.00) | 0.00 (0.00, 0.00) | 0.00 (0.00, 0.00) | 0.00 (0.00, 0.00) | NA | NA |
| Central Asia | 0.00 (0.00, 0.00) | 0.00 (0.00, 0.00) | 0.00 (0.00, 0.00) | 0.00 (0.00, 0.00) | NA | NA |
| Central Europe | 0.00 (0.00, 0.00) | 0.00 (0.00, 0.00) | 0.00 (0.00, 0.00) | 0.00 (0.00, 0.00) | NA | NA |
| Eastern Europe | 0.00 (0.00, 0.00) | 0.00 (0.00, 0.00) | 0.00 (0.00, 0.00) | 0.00 (0.00, 0.00) | NA | NA |
| High-income Asia Pacific | 0.00 (0.00, 0.00) | 0.00 (0.00, 0.00) | 0.00 (0.00, 0.00) | 0.00 (0.00, 0.00) | NA | NA |
| Australasia | 0.00 (0.00, 0.00) | 0.00 (0.00, 0.00) | 0.00 (0.00, 0.00) | 0.00 (0.00, 0.00) | NA | NA |
| Western Europe | 0.00 (0.00, 0.00) | 0.00 (0.00, 0.00) | 0.00 (0.00, 0.00) | 0.00 (0.00, 0.00) | NA | NA |
| Southern Latin America | 0.00 (0.00, 0.00) | 1.83 (0.18, 9.08) | 0.00 (0.00, 0.00) | 0.00 (0.00, 0.01) | NA | NA |
| High-income North America | 0.00 (0.00, 0.00) | 0.02 (0.00, 0.12) | 0.00 (0.00, 0.00) | 0.00 (0.00, 0.00) | NA | NA |
| Caribbean | 0.00 (0.00, 0.00) | 109.35 (22.41, 365.29) | 0.00 (0.00, 0.00) | 0.22 (0.05, 0.75) | NA | NA |
| Andean Latin America | 0.00 (0.00, 0.00) | 517.19 (122.83, 1724.72) | 0.00 (0.00, 0.00) | 0.78 (0.19, 2.60) | NA | NA |
| Central Latin America | 0.00 (0.00, 0.00) | 883.15 (200.38, 3809.13) | 0.00 (0.00, 0.00) | 0.35 (0.08, 1.48) | NA | NA |
| Tropical Latin America | 0.00 (0.00, 0.00) | 1423.45 (611.54, 3526.05) | 0.00 (0.00, 0.00) | 0.62 (0.27, 1.55) | NA | NA |
| North Africa and Middle East | 0.00 (0.00, 0.00) | 0.00 (0.00, 0.00) | 0.00 (0.00, 0.00) | 0.00 (0.00, 0.00) | NA | NA |
| South Asia | 0.00 (0.00, 0.00) | 0.00 (0.00, 0.00) | 0.00 (0.00, 0.00) | 0.00 (0.00, 0.00) | NA | NA |
| Central Sub-Saharan Africa | 0.00 (0.00, 0.00) | 0.00 (0.00, 0.00) | 0.00 (0.00, 0.00) | 0.00 (0.00, 0.00) | NA | NA |
| Eastern Sub-Saharan Africa | 0.00 (0.00, 0.00) | 0.00 (0.00, 0.00) | 0.00 (0.00, 0.00) | 0.00 (0.00, 0.00) | NA | NA |
| Southern Sub-Saharan Africa | 0.00 (0.00, 0.00) | 0.00 (0.00, 0.00) | 0.00 (0.00, 0.00) | 0.00 (0.00, 0.00) | NA | NA |
| Western Sub-Saharan Africa | 0.00 (0.00, 0.00) | 0.00 (0.00, 0.00) | 0.00 (0.00, 0.00) | 0.00 (0.00, 0.00) | NA | NA |

ASPRs age-standardized prevalence rates, AAPC average annual percentage change, UI uncertainty intervals, CI confidence interval, SDI Socio-demographic Index

**Table D** DALYs, ASDRs in 1990 and 2021, and AAPCs from 1990 to 2021 for nine arthropod-borne diseases in global and 21 regions

| **Location** | **1990 DALYs** | **2021 DALYs** | **1990 ASDRs**  **(1/100,000, 95% UI)** | **2021 ASDRs**  **(1/100,000, 95% UI)** | **AAPCs**  **(95% CI)** | ***P* Value** |
| --- | --- | --- | --- | --- | --- | --- |
| **Malaria** |  |  |  |  |  |  |
| Global | 57,888,052.59 (30,256,058.77, 113,079,353.06) | 55,174,060.70 (21,761,299.60, 108,337,905.75) | 965.67 (502.56, 1898.07) | 806.00 (318.93, 1570.18) | -0.56  (-0.98, -0.14) | <0.05 |
| High SDI | 7080.64 (4027.33, 11,648.42) | 485.66 (338.20, 658.76) | 0.83 (0.47, 1.39) | 0.05 (0.03, 0.07) | -8.15  (-11.79, -4.36) | <0.05 |
| High-middle SDI | 61,606.08 (10,680.88, 416,196.09) | 7089.65 (1584.70, 18,620.03) | 6.25 (1.02, 42.80) | 0.74 (0.14, 1.93) | -7.98  (-16.12, 0.95) | 0.079 |
| Middle SDI | 5,572,397.88 (2,540,038.03, 12,856,865.75) | 3,520,699.12 (1,363,904.67, 6,866,644.71) | 297.44 (134.69, 690.53) | 177.62 (70.05, 334.71) | -1.68  (-2.47, -0.88) | <0.05 |
| Low-middle SDI | 14,602,281.25 (6,728,092.81, 34,149,292.49) | 11,962,347.04 (4,752,188.80, 24,021,333.55) | 978.75 (448.70, 2297.63) | 630.94 (249.10, 1273.67) | -1.26  (-1.86, -0.66) | <0.05 |
| Low SDI | 37,618,122.37 (20,712,509.29, 64,872,173.92) | 39,663,871.90 (15,715,986.40, 75,990,380.81) | 4811.47 (2580.65, 8569.12) | 2869.17 (1107.42, 5683.87) | -1.63  (-1.92, -1.33) | <0.05 |
| East Asia | 64,233.31 (3111.51, 703,998.59) | 224.61 (163.67, 308.63) | 5.01 (0.25, 54.63) | 0.02 (0.01, 0.02) | -14.24  (-16.47, -11.94) | <0.05 |
| Southeast Asia | 883,886.51 (255,823.37, 3,436,233.61) | 110,524.33 (34,865.88, 363,321.60) | 176.96 (49.32, 710.39) | 16.06 (5.18, 52.09) | -7.21  (-9.00, -5.38) | <0.05 |
| Oceania | 173,720.07 (40,839.83, 499,521.11) | 146,292.01 (66,771.79, 266,017.95) | 2624.23 (580.93, 8023.55) | 1047.48 (474.82, 1931.01) | -3.59  (-6.52, -0.57) | <0.05 |
| Central Asia | 28,947.65 (4177.57, 81,910.44) | 0.00 (0.00, 0.00) | 31.29 (4.84, 87.82) | 0.00 (0.00, 0.00) | NA | NA |
| Central Europe | 0.00 (0.00, 0.00) | 0.00 (0.00, 0.00) | 0.00 (0.00, 0.00) | 0.00 (0.00, 0.00) | NA | NA |
| Eastern Europe | 0.00 (0.00, 0.00) | 0.00 (0.00, 0.00) | 0.00 (0.00, 0.00) | 0.00 (0.00, 0.00) | NA | NA |
| High-income Asia Pacific | 2145.57 (1712.69, 2642.41) | 62.01 (51.10, 76.51) | 1.22 (0.97, 1.51) | 0.03 (0.02, 0.04) | -11.37  (-11.88, -10.85) | <0.05 |
| Australasia | 0.00 (0.00, 0.00) | 0.00 (0.00, 0.00) | 0.00 (0.00, 0.00) | 0.00 (0.00, 0.00) | NA | NA |
| Western Europe | 498.05 (268.57, 764.22) | 0.00 (0.00, 0.00) | 0.11 (0.06, 0.17) | 0.00 (0.00, 0.00) | NA | NA |
| Southern Latin America | 1580.46 (1239.35, 1965.37) | 0.00 (0.00, 0.00) | 3.19 (2.51, 3.96) | 0.00 (0.00, 0.00) | NA | NA |
| High-income North America | 0.00 (0.00, 0.00) | 0.00 (0.00, 0.00) | 0.00 (0.00, 0.00) | 0.00 (0.00, 0.00) | NA | NA |
| Caribbean | 56,239.82 (21,157.16, 131,181.80) | 47,765.20 (7055.56, 148,307.18) | 146.84 (54.67, 345.00) | 103.69 (15.42, 321.88) | -4.80  (-11.60, 2.53) | 0.194 |
| Andean Latin America | 107,039.40 (24,472.74, 273,439.70) | 2153.97 (798.31, 7401.01) | 253.35 (57.03, 653.89) | 3.24 (1.22, 11.06) | -15.20  (-21.25, -8.67) | <0.05 |
| Central Latin America | 79,374.03 (40,098.85, 150,426.19) | 31,985.88 (7411.69, 75,722.58) | 42.61 (22.16, 79.17) | 12.97 (3.02, 30.76) | -3.32  (-7.49, 1.04) | 0.134 |
| Tropical Latin America | 278,398.18 (105,569.91, 544,162.36) | 13,026.32 (3188.42, 33,877.54) | 171.27 (63.86, 339.38) | 6.01 (1.46, 15.63) | -10.33  (-12.34, -8.28) | <0.05 |
| North Africa and Middle East | 759,192.18 (296,143.71, 1,727,365.66) | 786,625.40 (237,427.86, 1,697,098.01) | 195.66 (74.19, 460.14) | 125.54 (37.81, 272.14) | -1.23  (-2.42, -0.02) | <0.05 |
| South Asia | 8,419,029.55 (3,240,125.32, 26,469,709.63) | 1,820,079.64 (126,366.23, 6,010,614.07) | 597.82 (226.79, 1948.58) | 104.10 (6.91, 344.83) | -6.46  (-8.41, -4.46) | <0.05 |
| Central Sub-Saharan Africa | 7,922,213.81 (4,494,769.08, 12,582,901.01) | 7,237,039.49 (3,416,571.46, 12,922,264.35) | 8226.00 (4631.09, 13,128.43) | 4076.68 (1861.99, 7643.64) | -2.30  (-2.65, -1.96) | <0.05 |
| Eastern Sub-Saharan Africa | 15,550,835.26 (9,185,012.87, 25,773,213.25) | 11,609,738.74 (4,520,723.84, 21,788,865.49) | 5089.41 (2906.41, 8550.17) | 2199.73 (830.69, 4301.55) | -2.63  (-3.17, -2.10) | <0.05 |
| Southern Sub-Saharan Africa | 245,398.24 (91,667.43, 515,076.54) | 145,604.46 (63,098.15, 305,447.87) | 375.87 (136.06, 823.18) | 179.57 (77.69, 376.84) | 0.05  (-1.89, 2.03) | 0.961 |
| Western Sub-Saharan Africa | 23,315,320.50 (12,114,729.28, 39,957,611.19) | 33,222,938.65 (13,351,431.17, 63,411,625.35) | 8231.69 (4281.57, 14,190.95) | 5668.41 (2216.18, 11,127.48) | -1.16  (-1.44, -0.87) | <0.05 |
| **Chagas disease** |  |  |  |  |  |  |
| Global | 413,237.98 (363,137.65, 457,514.62) | 238,589.29 (207,472.14, 271,774.84) | 9.66 (8.47, 10.67) | 2.78 (2.41, 3.17) | -3.92  (-4.07, -3.77) | <0.05 |
| High SDI | 2250.76 (1516.46, 3272.31) | 2834.59 (1835.13, 4181.55) | 0.22 (0.15, 0.32) | 0.18 (0.12, 0.27) | -0.59  (-0.77, -0.42) | <0.05 |
| High-middle SDI | 48,975.78 (39,577.28, 59,385.05) | 24,133.34 (19,196.51, 30,721.61) | 4.92 (3.97, 5.96) | 1.34 (1.06, 1.71) | -4.07  (-4.20, -3.94) | <0.05 |
| Middle SDI | 255,897.45 (226,714.54, 278,402.87) | 127,702.98 (110,900.93, 145,756.27) | 21.47 (18.99, 23.26) | 4.76 (4.13, 5.41) | -4.71  (-4.89, -4.53) | <0.05 |
| Low-middle SDI | 106,101.27 (88,698.19, 123,135.56) | 83,916.07 (71,679.66, 98,392.76) | 16.33 (13.60, 19.01) | 5.76 (4.87, 6.79) | -3.29  (-3.42, -3.15) | <0.05 |
| Low SDI | 0.00 (0.00, 0.00) | 0.00 (0.00, 0.00) | 0.00 (0.00, 0.00) | 0.00 (0.00, 0.00) | NA | NA |
| East Asia | 0.00 (0.00, 0.00) | 0.00 (0.00, 0.00) | 0.00 (0.00, 0.00) | 0.00 (0.00, 0.00) | NA | NA |
| Southeast Asia | 0.00 (0.00, 0.00) | 0.00 (0.00, 0.00) | 0.00 (0.00, 0.00) | 0.00 (0.00, 0.00) | NA | NA |
| Oceania | 0.00 (0.00, 0.00) | 0.00 (0.00, 0.00) | 0.00 (0.00, 0.00) | 0.00 (0.00, 0.00) | NA | NA |
| Central Asia | 0.00 (0.00, 0.00) | 0.00 (0.00, 0.00) | 0.00 (0.00, 0.00) | 0.00 (0.00, 0.00) | NA | NA |
| Central Europe | 0.00 (0.00, 0.00) | 0.11 (0.07, 0.18) | 0.00 (0.00, 0.00) | 0.00 (0.00, 0.00) | NA | NA |
| Eastern Europe | 0.00 (0.00, 0.00) | 0.00 (0.00, 0.00) | 0.00 (0.00, 0.00) | 0.00 (0.00, 0.00) | NA | NA |
| High-income Asia Pacific | 19.24 (12.39, 28.82) | 61.41 (39.78, 94.03) | 0.01 (0.01, 0.01) | 0.02 (0.01, 0.03) | 2.53  (2.10, 2.96) | <0.05 |
| Australasia | 25.35 (16.73, 38.11) | 18.28 (11.86, 27.37) | 0.11 (0.08, 0.17) | 0.04 (0.03, 0.07) | -3.05  (-3.13, -2.98) | <0.05 |
| Western Europe | 184.56 (121.33, 277.48) | 466.74 (305.99, 713.31) | 0.04 (0.02, 0.06) | 0.07 (0.05, 0.11) | 2.16  (1.73-2.60) | <0.05 |
| Southern Latin America | 39,939.94 (31,243.63, 50,136.99) | 19,375.96 (14,832.62, 25,478.41) | 86.60 (67.64, 108.40) | 23.54 (17.91, 31.03) | -4.08  (-4.20, -3.96) | <0.05 |
| High-income North America | 1993.93 (1293.48, 2912.01) | 2644.83 (1713.37, 3902.69) | 0.61 (0.39, 0.89) | 0.52 (0.34, 0.78) | -0.48  (-0.66, -0.30) | <0.05 |
| Caribbean | 313.38 (222.91, 421.46) | 23.22 (15.50, 33.78) | 1.68 (1.16, 2.26) | 0.04 (0.03, 0.06) | -11.97  (-14.44, -9.43) | <0.05 |
| Andean Latin America | 24,385.07 (18,196.21, 32,254.55) | 17,723.87 (12,996.26, 23,932.32) | 108.74 (80.30, 145.59) | 28.93 (21.23, 39.11) | -4.20  (-4.31, -4.09) | <0.05 |
| Central Latin America | 40,556.47 (31,514.34, 49,396.50) | 44,669.99 (34,789.70, 57,449.73) | 44.99 (34.98, 54.40) | 17.58 (13.73, 22.61) | -2.99  (-3.14, -2.83) | <0.05 |
| Tropical Latin America | 305,820.04 (270,228.06, 331,490.18) | 153,604.87 (136,074.65, 172,363.91) | 288.54 (255.35, 311.89) | 59.28 (52.48, 66.55) | -4.95  (-5.13, -4.78) | <0.05 |
| North Africa and Middle East | 0.00 (0.00, 0.00) | 0.00 (0.00, 0.00) | 0.00 (0.00, 0.00) | 0.00 (0.00, 0.00) | NA | NA |
| South Asia | 0.00 (0.00, 0.00) | 0.00 (0.00, 0.00) | 0.00 (0.00, 0.00) | 0.00 (0.00, 0.00) | NA | NA |
| Central Sub-Saharan Africa | 0.00 (0.00, 0.00) | 0.00 (0.00, 0.00) | 0.00 (0.00, 0.00) | 0.00 (0.00, 0.00) | NA | NA |
| Eastern Sub-Saharan Africa | 0.00 (0.00, 0.00) | 0.00 (0.00, 0.00) | 0.00 (0.00, 0.00) | 0.00 (0.00, 0.00) | NA | NA |
| Southern Sub-Saharan Africa | 0.00 (0.00, 0.00) | 0.00 (0.00, 0.00) | 0.00 (0.00, 0.00) | 0.00 (0.00, 0.00) | NA | NA |
| Western Sub-Saharan Africa | 0.00 (0.00, 0.00) | 0.00 (0.00, 0.00) | 0.00 (0.00, 0.00) | 0.00 (0.00, 0.00) | NA | NA |
| **Leishmaniasis** |  |  |  |  |  |  |
| Global | 4,670,786.77 (1,752,402.68, 14,551,294.17) | 781,187.61 (468,168.17, 1,636,591.71) | 79.59 (29.90, 246.55) | 10.27 (5.99, 21.96) | -6.65  (-7.68, -5.62) | <0.05 |
| High SDI | 9865.96 (2861.23, 35,756.33) | 10,715.31 (6453.00, 17,389.14) | 1.20 (0.33, 4.89) | 0.92 (0.55, 1.43) | -0.87  (-1.01, -0.73) | <0.05 |
| High-middle SDI | 44,318.76 (5499.72, 332,670.49) | 14,307.55 (7590.25, 31,873.06) | 4.34 (0.52, 32.70) | 1.10 (0.56, 2.98) | -4.35  (-4.63, -4.08) | <0.05 |
| Middle SDI | 247,700.64 (56,758.44, 1,119,519.17) | 205,241.56 (122,914.80, 343,124.69) | 14.00 (3.70, 58.40) | 8.33 (4.89, 14.51) | -1.70  (-1.88, -1.52) | <0.05 |
| Low-middle SDI | 1,021,707.98 (237,344.31, 4,006,642.11) | 229,553.05 (110,913.32, 630,089.61) | 73.17 (18.50, 276.45) | 12.24 (6.03, 32.11) | -5.73  (-6.29, -5.17) | <0.05 |
| Low SDI | 3,345,536.58 (1,379,709.39, 9,050,696.73) | 320,939.60 (183,604.10, 622,156.58) | 504.88 (200.59, 1383.65) | 25.20 (15.06, 47.50) | -9.55  (-11.56, -7.50) | <0.05 |
| East Asia | 63,324.31 (1122.40, 347,567.57) | 9821.96 (761.60, 48,835.64) | 5.20 (0.10, 28.17) | 0.82 (0.04, 4.38) | -5.93  (-6.44, -5.42) | <0.05 |
| Southeast Asia | 789.76 (147.20, 2755.42) | 1028.00 (626.15, 1517.69) | 0.17 (0.04, 0.52) | 0.15 (0.09, 0.22) | -0.39  (-0.61, -0.17) | <0.05 |
| Oceania | 0.00 (0.00, 0.00) | 0.00 (0.00, 0.00) | 0.00 (0.00, 0.00) | 0.00 (0.00, 0.00) | NA | NA |
| Central Asia | 9950.64 (5548.55, 19,982.54) | 8870.79 (4428.42, 27,278.56) | 15.47 (8.83, 28.13) | 9.43 (4.77, 28.34) | -1.63  (-1.75, -1.50) | <0.05 |
| Central Europe | 9269.18 (93.61, 87,956.03) | 385.60 (48.10, 2980.33) | 8.47 (0.07, 80.58) | 0.43 (0.03, 3.79) | -9.04  (-9.99, -8.07) | <0.05 |
| Eastern Europe | 0.00 (0.00, 0.00) | 0.00 (0.00, 0.00) | 0.00 (0.00, 0.00) | 0.00 (0.00, 0.00) | NA | NA |
| High-income Asia Pacific | 0.00 (0.00, 0.00) | 0.00 (0.00, 0.00) | 0.00 (0.00, 0.00) | 0.00 (0.00, 0.00) | NA | NA |
| Australasia | 0.00 (0.00, 0.00) | 0.00 (0.00, 0.00) | 0.00 (0.00, 0.00) | 0.00 (0.00, 0.00) | NA | NA |
| Western Europe | 4807.91 (1135.45, 31,830.28) | 1645.57 (593.05, 8296.39) | 1.57 (0.35, 10.77) | 0.49 (0.17, 2.71) | -3.69  (-4.63, -2.75) | <0.05 |
| Southern Latin America | 593.37 (144.12, 2370.01) | 656.75 (367.99, 1168.94) | 1.21 (0.30, 4.68) | 0.92 (0.51, 1.82) | -0.87  (-1.14, -0.60) | <0.05 |
| High-income North America | 17.79 (4.18, 47.37) | 27.57 (14.21, 49.98) | 0.01 (0.00, 0.02) | 0.01 (0.00, 0.01) | 0.34  (0.31, 0.37) | <0.05 |
| Caribbean | 1274.60 (637.27, 2316.85) | 1661.45 (1079.00, 2444.27) | 3.82 (1.93, 6.95) | 3.37 (2.19, 4.92) | -0.40  (-0.53, -0.28) | <0.05 |
| Andean Latin America | 16,771.79 (7222.14, 40,696.07) | 23,161.66 (15,488.05, 32,892.54) | 47.21 (22.40, 98.00) | 35.47 (23.75, 50.63) | -0.91  (-0.96, -0.86) | <0.05 |
| Central Latin America | 23,844.01 (12,790.25, 46,237.04) | 36,034.51 (23,656.30, 52,082.89) | 16.86 (9.51, 29.08) | 14.12 (9.26, 20.45) | -0.57  (-0.68, -0.47) | <0.05 |
| Tropical Latin America | 103,878.45 (19,521.36, 330,331.13) | 114,002.54 (36,297.40, 294,025.13) | 64.36 (14.26, 191.41) | 52.47 (14.76, 143.55) | -0.54  (-1.07, -0.01) | <0.05 |
| North Africa and Middle East | 346,970.49 (76,374.26, 2,169,155.64) | 272,525.59 (170,483.77, 492,504.45) | 96.37 (27.75, 519.24) | 45.16 (28.39, 79.84) | -2.52  (-2.74, -2.30) | <0.05 |
| South Asia | 2,002,800.05 (13,705.40, 9,288,452.50) | 106,311.74 (15,610.71, 478,610.30) | 152.98 (1.40, 693.40) | 5.88 (0.85, 26.13) | -10.23  (-11.36, -9.07) | <0.05 |
| Central Sub-Saharan Africa | 303,774.87 (159,648.10, 485,337.28) | 47,360.24 (24,257.45, 77,017.45) | 389.40 (205.09, 617.22) | 26.79 (13.95, 43.09) | -8.59  (-10.38, -6.76) | <0.05 |
| Eastern Sub-Saharan Africa | 1,777,539.97 (1,188,355.44, 2,524,592.53) | 127,772.79 (76,095.49, 194,043.47) | 653.30 (442.46, 909.04) | 22.99 (13.88, 34.59) | -10.50  (-12.38, -8.59) | <0.05 |
| Southern Sub-Saharan Africa | 19.94 (7.40, 44.73) | 31.71 (19.36, 46.75) | 0.05 (0.02, 0.10) | 0.04 (0.03, 0.06) | -0.35  (-0.41, -0.29) | <0.05 |
| Western Sub-Saharan Africa | 5159.65 (3221.49, 7958.84) | 29,889.14 (19,044.34, 44,056.79) | 2.94 (1.77, 4.63) | 5.54 (3.74, 7.81) | 2.08  (1.86, 2.31) | <0.05 |
| **African trypanosomiasis** |  |  |  |  |  |  |
| Global | 1,085,692.03 (541,801.56, 1,733,208.09) | 61,962.48 (22,232.70, 176,263.81) | 19.88 (9.96, 31.86) | 0.79 (0.28, 2.26) | -9.61  (-12.49, -6.64) | <0.05 |
| High SDI | 0.00 (0.00, 0.00) | 0.00 (0.00, 0.00) | 0.00 (0.00, 0.00) | 0.00 (0.00, 0.00) | NA | NA |
| High-middle SDI | 0.00 (0.00, 0.00) | 0.00 (0.00, 0.00) | 0.00 (0.00, 0.00) | 0.00 (0.00, 0.00) | NA | NA |
| Middle SDI | 17,064.04 (7372.58, 30,211.41) | 3919.06 (1472.27, 8209.80) | 1.00 (0.43, 1.79) | 0.16 (0.06, 0.33) | -5.90  (-7.32, -4.47) | <0.05 |
| Low-middle SDI | 190,108.77 (94,324.18, 310,353.38) | 13,067.35 (5710.43, 29,657.89) | 17.08 (8.37, 28.19) | 0.65 (0.28, 1.47) | -10.21  (-14.44, -5.76) | <0.05 |
| Low SDI | 878,202.41 (422,700.12, 1,427,695.14) | 44,954.83 (14,243.24, 142,133.14) | 193.87 (94.41, 316.19) | 4.10 (1.31, 13.03) | -12.75  (-14.06, -11.41) | <0.05 |
| East Asia | 0.00 (0.00, 0.00) | 0.00 (0.00, 0.00) | 0.00 (0.00, 0.00) | 0.00 (0.00, 0.00) | NA | NA |
| Southeast Asia | 0.00 (0.00, 0.00) | 0.00 (0.00, 0.00) | 0.00 (0.00, 0.00) | 0.00 (0.00, 0.00) | NA | NA |
| Oceania | 0.00 (0.00, 0.00) | 0.00 (0.00, 0.00) | 0.00 (0.00, 0.00) | 0.00 (0.00, 0.00) | NA | NA |
| Central Asia | 0.00 (0.00, 0.00) | 0.00 (0.00, 0.00) | 0.00 (0.00, 0.00) | 0.00 (0.00, 0.00) | NA | NA |
| Central Europe | 0.00 (0.00, 0.00) | 0.00 (0.00, 0.00) | 0.00 (0.00, 0.00) | 0.00 (0.00, 0.00) | NA | NA |
| Eastern Europe | 0.00 (0.00, 0.00) | 0.00 (0.00, 0.00) | 0.00 (0.00, 0.00) | 0.00 (0.00, 0.00) | NA | NA |
| High-income Asia Pacific | 0.00 (0.00, 0.00) | 0.00 (0.00, 0.00) | 0.00 (0.00, 0.00) | 0.00 (0.00, 0.00) | NA | NA |
| Australasia | 0.00 (0.00, 0.00) | 0.00 (0.00, 0.00) | 0.00 (0.00, 0.00) | 0.00 (0.00, 0.00) | NA | NA |
| Western Europe | 0.00 (0.00, 0.00) | 0.00 (0.00, 0.00) | 0.00 (0.00, 0.00) | 0.00 (0.00, 0.00) | NA | NA |
| Southern Latin America | 0.00 (0.00, 0.00) | 0.00 (0.00, 0.00) | 0.00 (0.00, 0.00) | 0.00 (0.00, 0.00) | NA | NA |
| High-income North America | 0.00 (0.00, 0.00) | 0.00 (0.00, 0.00) | 0.00 (0.00, 0.00) | 0.00 (0.00, 0.00) | NA | NA |
| Caribbean | 0.00 (0.00, 0.00) | 0.00 (0.00, 0.00) | 0.00 (0.00, 0.00) | 0.00 (0.00, 0.00) | NA | NA |
| Andean Latin America | 0.00 (0.00, 0.00) | 0.00 (0.00, 0.00) | 0.00 (0.00, 0.00) | 0.00 (0.00, 0.00) | NA | NA |
| Central Latin America | 0.00 (0.00, 0.00) | 0.00 (0.00, 0.00) | 0.00 (0.00, 0.00) | 0.00 (0.00, 0.00) | NA | NA |
| Tropical Latin America | 0.00 (0.00, 0.00) | 0.00 (0.00, 0.00) | 0.00 (0.00, 0.00) | 0.00 (0.00, 0.00) | NA | NA |
| North Africa and Middle East | 0.00 (0.00, 0.00) | 0.00 (0.00, 0.00) | 0.00 (0.00, 0.00) | 0.00 (0.00, 0.00) | NA | NA |
| South Asia | 0.00 (0.00, 0.00) | 0.00 (0.00, 0.00) | 0.00 (0.00, 0.00) | 0.00 (0.00, 0.00) | NA | NA |
| Central Sub-Saharan Africa | 566,073.24 (285,269.23, 931,612.39) | 18,691.54 (9275.01, 32,271.17) | 1181.12 (585.29, 1964.79) | 15.04 (7.43, 25.87) | -13.21  (-14.53, -11.86) | <0.05 |
| Eastern Sub-Saharan Africa | 439,589.27 (187,185.75, 774,885.88) | 24,148.43 (3281.59, 120,468.79) | 261.79 (111.40, 466.57) | 5.78 (0.80, 28.93) | -11.26  (-17.00, -5.12) | <0.05 |
| Southern Sub-Saharan Africa | 0.00 (0.00, 0.00) | 0.00 (0.00, 0.00) | 0.00 (0.00, 0.00) | 0.00 (0.00, 0.00) | NA | NA |
| Western Sub-Saharan Africa | 80,029.52 (33,194.02, 150,144.64) | 19,122.51 (6970.20, 38,822.56) | 45.40 (18.68, 84.69) | 4.11 (1.51, 8.36) | -8.97  (-10.04, -7.90) | <0.05 |
| **Lymphatic filariasis** |  |  |  |  |  |  |
| Global | 4,082,608.43 (2,749,036.51, 5,712,220.36) | 1,314,563.40 (768,842.08, 2,224,976.28) | 79.10 (53.45, 110.49) | 16.50 (9.65, 27.96) | -4.92  (-5.14, -4.71) | <0.05 |
| High SDI | 0.00 (0.00, 0.00) | 0.00 (0.00, 0.00) | 0.00 (0.00, 0.00) | 0.00 (0.00, 0.00) | NA | NA |
| High-middle SDI | 58,104.31 (37,489.35, 85,685.86) | 26,735.44 (15,037.23, 47,453.00) | 5.31 (3.43, 7.83) | 2.16 (1.23, 3.83) | -2.77  (-3.10, -2.43) | <0.05 |
| Middle SDI | 1,079,045.69 (728,110.07, 1,528,288.00) | 346,851.97 (199,262.03, 597,948.71) | 67.29 (45.44, 94.84) | 13.78 (7.93, 23.76) | -4.99  (-5.56, -4.43) | <0.05 |
| Low-middle SDI | 1,826,159.93 (1,234,997.47, 2,579,403.04) | 568,754.94 (334,301.70, 958,431.67) | 186.21 (127.24, 261.55) | 30.02 (17.67, 50.32) | -5.71  (-5.99, -5.44) | <0.05 |
| Low SDI | 1,114,257.24 (751,803.49, 1,550,082.15) | 370,596.33 (219,787.94, 620,395.47) | 279.13 (189.86, 389.94) | 37.40 (22.23, 62.02) | -6.25  (-6.61, -5.88) | <0.05 |
| East Asia | 0.00 (0.00, 0.00) | 0.00 (0.00, 0.00) | 0.00 (0.00, 0.00) | 0.00 (0.00, 0.00) | NA | NA |
| Southeast Asia | 943,883.71 (630,656.20, 1,335,992.27) | 198,952.38 (114,300.47, 348,217.11) | 225.37 (150.85, 315.75) | 27.78 (16.00, 48.62) | -6.48  (-6.91, -6.05) | <0.05 |
| Oceania | 33,106.12 (22,910.72, 46,956.41) | 19,976.45 (13,019.26, 29,230.84) | 607.52 (419.56, 865.23) | 163.51 (107.49, 237.26) | -4.18  (-4.42, -3.95) | <0.05 |
| Central Asia | 0.00 (0.00, 0.00) | 0.00 (0.00, 0.00) | 0.00 (0.00, 0.00) | 0.00 (0.00, 0.00) | NA | NA |
| Central Europe | 0.00 (0.00, 0.00) | 0.00 (0.00, 0.00) | 0.00 (0.00, 0.00) | 0.00 (0.00, 0.00) | NA | NA |
| Eastern Europe | 0.00 (0.00, 0.00) | 0.00 (0.00, 0.00) | 0.00 (0.00, 0.00) | 0.00 (0.00, 0.00) | NA | NA |
| High-income Asia Pacific | 93.58 (52.47, 167.34) | 139.03 (77.25, 250.78) | 0.06 (0.03, 0.10) | 0.09 (0.05, 0.17) | 1.74  (1.50-1.98) | <0.05 |
| Australasia | 0.00 (0.00, 0.00) | 0.00 (0.00, 0.00) | 0.00 (0.00, 0.00) | 0.00 (0.00, 0.00) | NA | NA |
| Western Europe | 0.00 (0.00, 0.00) | 0.00 (0.00, 0.00) | 0.00 (0.00, 0.00) | 0.00 (0.00, 0.00) | NA | NA |
| Southern Latin America | 0.00 (0.00, 0.00) | 0.00 (0.00, 0.00) | 0.00 (0.00, 0.00) | 0.00 (0.00, 0.00) | NA | NA |
| High-income North America | 0.00 (0.00, 0.00) | 0.00 (0.00, 0.00) | 0.00 (0.00, 0.00) | 0.00 (0.00, 0.00) | NA | NA |
| Caribbean | 36,970.97 (25,090.49, 51,535.92) | 10,123.51 (6343.74, 16,605.73) | 108.22 (73.69, 150.95) | 21.17 (13.28, 34.85) | -5.04  (-6.15, -3.93) | <0.05 |
| Andean Latin America | 0.00 (0.00, 0.00) | 0.00 (0.00, 0.00) | 0.00 (0.00, 0.00) | 0.00 (0.00, 0.00) | NA | NA |
| Central Latin America | 0.00 (0.00, 0.00) | 0.00 (0.00, 0.00) | 0.00 (0.00, 0.00) | 0.00 (0.00, 0.00) | NA | NA |
| Tropical Latin America | 6775.16 (4429.43, 10,118.69) | 1547.64 (1069.24, 2214.15) | 4.59 (3.01, 6.86) | 0.68 (0.47, 0.98) | -5.98  (-6.37, -5.59) | <0.05 |
| North Africa and Middle East | 45,791.97 (26,437.47, 76,677.42) | 20,401.76 (11,735.10, 36,878.21) | 15.62 (9.00, 25.64) | 3.20 (1.85, 5.79) | -5.07  (-6.00, -4.14) | <0.05 |
| South Asia | 2,006,046.43 (1,353,461.22, 2,826,707.33) | 735,707.66 (428,341.87, 1,239,749.27) | 215.49 (147.28, 302.40) | 39.63 (23.14, 66.41) | -5.27  (-5.71, -4.83) | <0.05 |
| Central Sub-Saharan Africa | 126,549.41 (84,208.85, 177,435.30) | 48,605.70 (27,345.28, 84,824.71) | 286.26 (192.38, 395.92) | 39.11 (22.19, 67.62) | -6.24  (-6.49, -5.98) | <0.05 |
| Eastern Sub-Saharan Africa | 378,285.61 (257,183.42, 532,143.60) | 99,553.21 (58,781.66, 177,185.46) | 263.05 (178.89, 366.93) | 24.59 (14.43, 43.70) | -7.36  (-7.66, -7.07) | <0.05 |
| Southern Sub-Saharan Africa | 3291.93 (1885.94, 5744.04) | 4179.06 (2422.47, 7381.27) | 6.40 (3.66, 11.21) | 5.05 (2.94, 8.88) | -0.74  (-0.99, -0.50) | <0.05 |
| Western Sub-Saharan Africa | 501,813.54 (338,827.49, 701,256.46) | 175,377.00 (106,381.05, 288,534.89) | 322.73 (219.08, 446.79) | 41.08 (25.09, 66.57) | -6.46  (-7.14, -5.77) | <0.05 |
| **Onchocerciasis** |  |  |  |  |  |  |
| Global | 1,372,001.04 (825,527.00, 2,062,756.37) | 1,262,988.13 (753,578.31, 1,907,363.13) | 26.67 (16.01, 40.03) | 15.78 (9.36, 23.86) | -1.68  (-1.76, -1.59) | <0.05 |
| High SDI | 0.00 (0.00, 0.00) | 0.00 (0.00, 0.00) | 0.00 (0.00, 0.00) | 0.00 (0.00, 0.00) | NA | NA |
| High-middle SDI | 0.00 (0.00, 0.00) | 0.00 (0.00, 0.00) | 0.00 (0.00, 0.00) | 0.00 (0.00, 0.00) | NA | NA |
| Middle SDI | 77,123.59 (46,358.90, 114,371.89) | 47,114.49 (25,329.71, 76,576.67) | 4.88 (3.01, 7.26) | 1.87 (1.00, 3.05) | -3.06  (-3.21, -2.91) | <0.05 |
| Low-middle SDI | 277,690.54 (169,772.54, 408,669.85) | 177,695.06 (102,566.96, 273,199.93) | 28.18 (17.85, 41.14) | 9.50 (5.58, 14.44) | -3.45  (-3.50, -3.40) | <0.05 |
| Low SDI | 1,017,028.78 (609,366.57, 1,527,654.90) | 1,037,996.53 (625,521.48, 1,566,569.72) | 263.61 (160.53, 394.33) | 113.57 (69.37, 169.05) | -2.68  (-2.76, -2.59) | <0.05 |
| East Asia | 0.00 (0.00, 0.00) | 0.00 (0.00, 0.00) | 0.00 (0.00, 0.00) | 0.00 (0.00, 0.00) | NA | NA |
| Southeast Asia | 0.00 (0.00, 0.00) | 0.00 (0.00, 0.00) | 0.00 (0.00, 0.00) | 0.00 (0.00, 0.00) | NA | NA |
| Oceania | 0.00 (0.00, 0.00) | 0.00 (0.00, 0.00) | 0.00 (0.00, 0.00) | 0.00 (0.00, 0.00) | NA | NA |
| Central Asia | 0.00 (0.00, 0.00) | 0.00 (0.00, 0.00) | 0.00 (0.00, 0.00) | 0.00 (0.00, 0.00) | NA | NA |
| Central Europe | 0.00 (0.00, 0.00) | 0.00 (0.00, 0.00) | 0.00 (0.00, 0.00) | 0.00 (0.00, 0.00) | NA | NA |
| Eastern Europe | 0.00 (0.00, 0.00) | 0.00 (0.00, 0.00) | 0.00 (0.00, 0.00) | 0.00 (0.00, 0.00) | NA | NA |
| High-income Asia Pacific | 0.00 (0.00, 0.00) | 0.00 (0.00, 0.00) | 0.00 (0.00, 0.00) | 0.00 (0.00, 0.00) | NA | NA |
| Australasia | 0.00 (0.00, 0.00) | 0.00 (0.00, 0.00) | 0.00 (0.00, 0.00) | 0.00 (0.00, 0.00) | NA | NA |
| Western Europe | 0.00 (0.00, 0.00) | 0.00 (0.00, 0.00) | 0.00 (0.00, 0.00) | 0.00 (0.00, 0.00) | NA | NA |
| Southern Latin America | 0.00 (0.00, 0.00) | 0.00 (0.00, 0.00) | 0.00 (0.00, 0.00) | 0.00 (0.00, 0.00) | NA | NA |
| High-income North America | 0.00 (0.00, 0.00) | 0.00 (0.00, 0.00) | 0.00 (0.00, 0.00) | 0.00 (0.00, 0.00) | NA | NA |
| Caribbean | 0.00 (0.00, 0.00) | 0.00 (0.00, 0.00) | 0.00 (0.00, 0.00) | 0.00 (0.00, 0.00) | NA | NA |
| Andean Latin America | 1143.62 (752.76, 1641.56) | 0.00 (0.00, 0.00) | 3.54 (2.37, 5.05) | 0.00 (0.00, 0.00) | NA | NA |
| Central Latin America | 20,348.18 (14,128.19, 28,350.63) | 451.34 (185.58, 813.57) | 14.22 (9.92, 19.56) | 0.17 (0.07, 0.31) | -13.35  (-13.81, -12.89) | <0.05 |
| Tropical Latin America | 1423.74 (962.77, 1998.95) | 17.92 (1.65, 65.81) | 0.96 (0.65, 1.35) | 0.01 (0.00, 0.03) | -14.39  (-14.78, -13.99) | <0.05 |
| North Africa and Middle East | 6238.45 (3899.53, 9115.10) | 4371.29 (2588.79, 6332.40) | 2.32 (1.47, 3.39) | 0.73 (0.44, 1.06) | -3.67  (-3.82, -3.53) | <0.05 |
| South Asia | 0.00 (0.00, 0.00) | 0.00 (0.00, 0.00) | 0.00 (0.00, 0.00) | 0.00 (0.00, 0.00) | NA | NA |
| Central Sub-Saharan Africa | 460,011.95 (285,708.12, 676,460.63) | 679,502.49 (421,054.20, 1,016,962.04) | 1128.97 (722.57, 1657.69) | 627.20 (400.72, 918.20) | -1.88  (-1.94, -1.82) | <0.05 |
| Eastern Sub-Saharan Africa | 184,225.07 (110,310.10, 273,148.54) | 199,341.95 (116,278.21, 299,901.85) | 133.93 (83.69, 199.52) | 62.99 (38.24, 95.28) | -2.44  (-2.66, -2.22) | <0.05 |
| Southern Sub-Saharan Africa | 0.00 (0.00, 0.00) | 0.00 (0.00, 0.00) | 0.00 (0.00, 0.00) | 0.00 (0.00, 0.00) | NA | NA |
| Western Sub-Saharan Africa | 698,610.04 (407,776.91, 1,062,013.88) | 379,303.13 (206,205.24, 603,104.36) | 475.12 (286.10, 714.21) | 106.51 (60.75, 164.03) | -4.71  (-4.76, -4.65) | <0.05 |
| **Dengue** |  |  |  |  |  |  |
| Global | 1,248,669.44 (876,050.15, 1,552,996.46) | 2,076,524.69 (1,056,228.35, 3,130,717.69) | 21.63 (15.09, 26.92) | 27.76 (14.21, 41.65) | 0.83  (0.48-1.19) | <0.05 |
| High SDI | 3539.95 (698.25, 9046.54) | 6494.66 (1606.63, 15,294.08) | 0.42 (0.09, 1.07) | 0.58 (0.14, 1.39) | 1.00  (0.59-1.41) | <0.05 |
| High-middle SDI | 87,623.66 (61,326.63, 121,292.73) | 110,148.34 (64,062.56, 156,180.11) | 8.92 (6.20, 12.61) | 11.39 (6.67, 16.03) | 0.53  (0.01-1.05) | <0.05 |
| Middle SDI | 630,452.75 (403,745.10, 805,798.00) | 1,045,828.36 (575,251.63, 1,544,203.75) | 33.64 (21.46, 42.85) | 48.78 (27.32, 71.02) | 1.28  (0.89-1.67) | <0.05 |
| Low-middle SDI | 463,610.47 (326,499.31, 593,002.68) | 793,856.40 (351,111.93, 1,290,420.25) | 35.92 (24.95, 47.94) | 43.35 (19.69, 69.74) | 0.66  (0.35-0.96) | <0.05 |
| Low SDI | 62,235.85 (43,901.59, 85,211.83) | 118,794.90 (38,238.80, 222,378.23) | 11.84 (8.11, 16.08) | 12.34 (4.44, 22.08) | 0.25  (-0.53-1.04) | 0.529 |
| East Asia | 3889.78 (2247.11, 5661.56) | 1360.02 (568.26, 2540.32) | 0.34 (0.20, 0.49) | 0.09 (0.04, 0.18) | -4.10  (-5.01, -3.20) | <0.05 |
| Southeast Asia | 742,194.35 (469,882.43, 1,158,430.46) | 909,114.20 (589,034.85, 1,241,630.50) | 134.99 (88.76, 204.74) | 147.04 (95.32, 200.97) | 0.44  (-0.22-1.10) | 0.193 |
| Oceania | 945.79 (645.03, 1424.32) | 766.78 (347.48, 1540.46) | 13.43 (8.60, 20.83) | 6.04 (2.73, 11.88) | -2.67  (-3.92, -1.40) | <0.05 |
| Central Asia | 0.00 (0.00, 0.00) | 0.00 (0.00, 0.00) | 0.00 (0.00, 0.00) | 0.00 (0.00, 0.00) | NA | NA |
| Central Europe | 0.00 (0.00, 0.00) | 0.00 (0.00, 0.00) | 0.00 (0.00, 0.00) | 0.00 (0.00, 0.00) | NA | NA |
| Eastern Europe | 0.00 (0.00, 0.00) | 0.00 (0.00, 0.00) | 0.00 (0.00, 0.00) | 0.00 (0.00, 0.00) | NA | NA |
| High-income Asia Pacific | 2175.23 (268.36, 5921.93) | 4713.22 (875.96, 12,044.73) | 1.30 (0.17, 3.53) | 2.90 (0.52, 7.39) | 2.54  (1.80-3.28) | <0.05 |
| Australasia | 59.70 (3.75, 200.24) | 191.74 (49.82, 498.28) | 0.30 (0.02, 0.99) | 0.61 (0.16, 1.60) | 2.53  (2.06-3.01) | <0.05 |
| Western Europe | 90.77 (43.05, 157.67) | 1.49 (0.51, 2.74) | 0.02 (0.01, 0.03) | 0.00 (0.00, 0.00) | -13.30  (-18.90, -7.32) | <0.05 |
| Southern Latin America | 399.89 (14.68, 1508.51) | 819.81 (149.57, 2211.01) | 0.81 (0.03, 3.05) | 1.22 (0.22, 3.28) | 1.29  (1.14-1.44) | <0.05 |
| High-income North America | 7.18 (3.72, 14.11) | 42.09 (15.85, 121.12) | 0.00 (0.00, 0.01) | 0.02 (0.01, 0.04) | 5.74  (4.72-6.77) | <0.05 |
| Caribbean | 1785.22 (304.22, 5235.65) | 2751.25 (546.46, 7216.81) | 5.02 (0.84, 14.80) | 5.83 (1.17, 15.48) | 0.43  (-1.54-2.44) | 0.668 |
| Andean Latin America | 1423.42 (304.36, 3500.82) | 4327.20 (1845.49, 8129.16) | 3.58 (0.74, 8.91) | 6.56 (2.80, 12.31) | 2.31  (0.93-3.71) | <0.05 |
| Central Latin America | 14,065.85 (4220.81, 29,841.91) | 43,074.33 (24,432.76, 67,687.04) | 8.26 (2.37, 17.68) | 17.53 (9.92, 27.60) | 2.31  (0.98-3.65) | <0.05 |
| Tropical Latin America | 67,757.32 (5126.05, 176,964.63) | 143,869.56 (50,995.10, 306,624.28) | 43.56 (3.32, 113.62) | 63.76 (22.43, 136.04) | 1.36  (0.92-1.79) | <0.05 |
| North Africa and Middle East | 894.99 (497.55, 1515.96) | 1035.07 (445.39, 2669.40) | 0.25 (0.15, 0.41) | 0.18 (0.08, 0.45) | -3.40  (-9.93-3.59) | 0.332 |
| South Asia | 388,993.59 (197,761.07, 625,033.32) | 931,668.08 (324,113.44, 1,630,476.70) | 35.79 (17.97, 57.41) | 53.46 (19.54, 91.91) | 1.34  (1.18-1.49) | <0.05 |
| Central Sub-Saharan Africa | 747.48 (12.48, 4636.78) | 2645.58 (125.85, 14,646.47) | 1.36 (0.02, 8.41) | 1.91 (0.09, 10.31) | 1.06  (0.84-1.27) | <0.05 |
| Eastern Sub-Saharan Africa | 16,106.09 (1432.56, 42,353.59) | 5940.78 (1374.58, 17,522.87) | 8.17 (0.69, 21.51) | 1.28 (0.23, 4.12) | -5.83  (-6.76, -4.88) | <0.05 |
| Southern Sub-Saharan Africa | 8.58 (1.05, 50.14) | 12.28 (1.13, 79.37) | 0.02 (0.00, 0.09) | 0.01 (0.00, 0.10) | -0.49  (-1.68-0.72) | 0.426 |
| Western Sub-Saharan Africa | 7124.21 (60.22, 27,505.24) | 24,191.21 (1156.70, 83,576.17) | 3.68 (0.02, 14.27) | 4.93 (0.24, 17.05) | 1.02  (0.72-1.33) | <0.05 |
| **Yellow fever** |  |  |  |  |  |  |
| Global | 987,964.64 (388,560.20, 2,101,213.43) | 317,791.95 (120,003.86, 686,270.60) | 17.06 (6.70, 36.36) | 4.29 (1.61, 9.24) | -3.81  (-4.10, -3.52) | <0.05 |
| High SDI | 0.00 (0.00, 0.00) | 0.00 (0.00, 0.00) | 0.00 (0.00, 0.00) | 0.00 (0.00, 0.00) | NA | NA |
| High-middle SDI | 7670.40 (1386.76, 23,735.16) | 1932.68 (380.80, 6596.69) | 0.73 (0.13, 2.25) | 0.18 (0.04, 0.61) | -4.00  (-4.23, -3.77) | <0.05 |
| Middle SDI | 88,660.96 (33,231.25, 204,395.57) | 7164.92 (2445.08, 15,952.50) | 4.70 (1.77, 10.94) | 0.32 (0.11, 0.70) | -6.00  (-7.27, -4.71) | <0.05 |
| Low-middle SDI | 349,714.70 (130,964.45, 763,595.10) | 64,609.54 (22,062.52, 154,591.80) | 26.43 (10.00, 57.82) | 3.15 (1.07, 7.55) | -5.75  (-6.21, -5.30) | <0.05 |
| Low SDI | 541,651.07 (209,446.24, 1,153,746.15) | 243,976.33 (92,362.31, 527,714.92) | 93.29 (35.84, 200.97) | 18.31 (7.00, 39.47) | -5.04  (-5.50, -4.58) | <0.05 |
| East Asia | 0.00 (0.00, 0.00) | 0.00 (0.00, 0.00) | 0.00 (0.00, 0.00) | 0.00 (0.00, 0.00) | NA | NA |
| Southeast Asia | 0.00 (0.00, 0.00) | 0.00 (0.00, 0.00) | 0.00 (0.00, 0.00) | 0.00 (0.00, 0.00) | NA | NA |
| Oceania | 0.00 (0.00, 0.00) | 0.00 (0.00, 0.00) | 0.00 (0.00, 0.00) | 0.00 (0.00, 0.00) | NA | NA |
| Central Asia | 0.00 (0.00, 0.00) | 0.00 (0.00, 0.00) | 0.00 (0.00, 0.00) | 0.00 (0.00, 0.00) | NA | NA |
| Central Europe | 0.00 (0.00, 0.00) | 0.00 (0.00, 0.00) | 0.00 (0.00, 0.00) | 0.00 (0.00, 0.00) | NA | NA |
| Eastern Europe | 0.00 (0.00, 0.00) | 0.00 (0.00, 0.00) | 0.00 (0.00, 0.00) | 0.00 (0.00, 0.00) | NA | NA |
| High-income Asia Pacific | 0.00 (0.00, 0.00) | 0.00 (0.00, 0.00) | 0.00 (0.00, 0.00) | 0.00 (0.00, 0.00) | NA | NA |
| Australasia | 0.00 (0.00, 0.00) | 0.00 (0.00, 0.00) | 0.00 (0.00, 0.00) | 0.00 (0.00, 0.00) | NA | NA |
| Western Europe | 0.00 (0.00, 0.00) | 0.00 (0.00, 0.00) | 0.00 (0.00, 0.00) | 0.00 (0.00, 0.00) | NA | NA |
| Southern Latin America | 7564.68 (1343.97, 23,644.00) | 1915.81 (366.98, 6573.20) | 14.94 (2.68, 46.54) | 3.06 (0.61, 10.40) | -4.91  (-5.01, -4.80) | <0.05 |
| High-income North America | 0.00 (0.00, 0.00) | 0.00 (0.00, 0.00) | 0.00 (0.00, 0.00) | 0.00 (0.00, 0.00) | NA | NA |
| Caribbean | 563.10 (140.86, 1527.95) | 107.25 (27.01, 309.92) | 1.48 (0.38, 4.00) | 0.24 (0.06, 0.69) | -5.51  (-5.87, -5.14) | <0.05 |
| Andean Latin America | 13,133.90 (3350.50, 35,591.80) | 3088.25 (778.55, 8401.24) | 30.64 (7.92, 81.38) | 4.61 (1.16, 12.54) | -7.04  (-8.14, -5.93) | <0.05 |
| Central Latin America | 1962.21 (617.86, 4558.26) | 520.81 (155.49, 1170.54) | 1.06 (0.33, 2.47) | 0.21 (0.06, 0.47) | -5.27  (-6.28, -4.25) | <0.05 |
| Tropical Latin America | 2537.33 (944.81, 5968.30) | 520.91 (189.82, 1214.86) | 1.48 (0.55, 3.51) | 0.25 (0.09, 0.59) | -9.13  (-15.67, -2.08) | <0.05 |
| North Africa and Middle East | 126,545.89 (25,961.40, 389,923.02) | 23,461.42 (4594.47, 72,318.00) | 32.52 (6.73, 96.84) | 3.62 (0.71, 11.12) | -7.14  (-7.33, -6.96) | <0.05 |
| South Asia | 0.00 (0.00, 0.00) | 0.00 (0.00, 0.00) | 0.00 (0.00, 0.00) | 0.00 (0.00, 0.00) | NA | NA |
| Central Sub-Saharan Africa | 86,441.45 (26,480.66, 227,282.10) | 34,204.16 (10,028.97, 88,384.59) | 139.85 (43.12, 358.57) | 22.10 (6.54, 57.55) | -5.30  (-6.19, -4.39) | <0.05 |
| Eastern Sub-Saharan Africa | 226,111.59 (74,088.40, 512,724.13) | 93,014.20 (31,321.17, 219,841.85) | 103.55 (35.33, 231.20) | 18.68 (6.16, 43.54) | -5.38  (-5.61, -5.16) | <0.05 |
| Southern Sub-Saharan Africa | 0.00 (0.00, 0.00) | 0.00 (0.00, 0.00) | 0.00 (0.00, 0.00) | 0.00 (0.00, 0.00) | NA | NA |
| Western Sub-Saharan Africa | 523,104.49 (207,936.88, 1,127,957.16) | 160,959.14 (60,857.30, 335,271.19) | 238.10 (96.22, 520.90) | 26.96 (10.40, 55.15) | -6.44  (-8.10, -4.75) | <0.05 |
| **Zika virus** |  |  |  |  |  |  |
| Global | 0.00 (0.00, 0.00) | 157.95 (103.34, 260.95) | 0.00 (0.00, 0.00) | 0.00 (0.00, 0.00) | NA | NA |
| High SDI | 0.00 (0.00, 0.00) | 0.12 (0.04, 0.31) | 0.00 (0.00, 0.00) | 0.00 (0.00, 0.00) | NA | NA |
| High-middle SDI | 0.00 (0.00, 0.00) | 0.71 (0.41, 1.28) | 0.00 (0.00, 0.00) | 0.00 (0.00, 0.00) | NA | NA |
| Middle SDI | 0.00 (0.00, 0.00) | 98.01 (61.82, 189.57) | 0.00 (0.00, 0.00) | 0.01 (0.00, 0.01) | NA | NA |
| Low-middle SDI | 0.00 (0.00, 0.00) | 58.91 (39.19, 84.09) | 0.00 (0.00, 0.00) | 0.00 (0.00, 0.00) | NA | NA |
| Low SDI | 0.00 (0.00, 0.00) | 0.04 (0.02, 0.09) | 0.00 (0.00, 0.00) | 0.00 (0.00, 0.00) | NA | NA |
| East Asia | 0.00 (0.00, 0.00) | 0.00 (0.00, 0.00) | 0.00 (0.00, 0.00) | 0.00 (0.00, 0.00) | NA | NA |
| Southeast Asia | 0.00 (0.00, 0.00) | 0.00 (0.00, 0.00) | 0.00 (0.00, 0.00) | 0.00 (0.00, 0.00) | NA | NA |
| Oceania | 0.00 (0.00, 0.00) | 0.00 (0.00, 0.00) | 0.00 (0.00, 0.00) | 0.00 (0.00, 0.00) | NA | NA |
| Central Asia | 0.00 (0.00, 0.00) | 0.00 (0.00, 0.00) | 0.00 (0.00, 0.00) | 0.00 (0.00, 0.00) | NA | NA |
| Central Europe | 0.00 (0.00, 0.00) | 0.00 (0.00, 0.00) | 0.00 (0.00, 0.00) | 0.00 (0.00, 0.00) | NA | NA |
| Eastern Europe | 0.00 (0.00, 0.00) | 0.00 (0.00, 0.00) | 0.00 (0.00, 0.00) | 0.00 (0.00, 0.00) | NA | NA |
| High-income Asia Pacific | 0.00 (0.00, 0.00) | 0.00 (0.00, 0.00) | 0.00 (0.00, 0.00) | 0.00 (0.00, 0.00) | NA | NA |
| Australasia | 0.00 (0.00, 0.00) | 0.00 (0.00, 0.00) | 0.00 (0.00, 0.00) | 0.00 (0.00, 0.00) | NA | NA |
| Western Europe | 0.00 (0.00, 0.00) | 0.00 (0.00, 0.00) | 0.00 (0.00, 0.00) | 0.00 (0.00, 0.00) | NA | NA |
| Southern Latin America | 0.00 (0.00, 0.00) | 0.08 (0.03, 0.24) | 0.00 (0.00, 0.00) | 0.00 (0.00, 0.00) | NA | NA |
| High-income North America | 0.00 (0.00, 0.00) | 0.00 (0.00, 0.00) | 0.00 (0.00, 0.00) | 0.00 (0.00, 0.00) | NA | NA |
| Caribbean | 0.00 (0.00, 0.00) | 4.65 (2.25, 9.85) | 0.00 (0.00, 0.00) | 0.01 (0.01, 0.02) | NA | NA |
| Andean Latin America | 0.00 (0.00, 0.00) | 29.85 (16.52, 55.59) | 0.00 (0.00, 0.00) | 0.05 (0.03, 0.09) | NA | NA |
| Central Latin America | 0.00 (0.00, 0.00) | 48.04 (24.87, 128.26) | 0.00 (0.00, 0.00) | 0.02 (0.01, 0.05) | NA | NA |
| Tropical Latin America | 0.00 (0.00, 0.00) | 75.32 (49.65, 110.60) | 0.00 (0.00, 0.00) | 0.04 (0.03, 0.06) | NA | NA |
| North Africa and Middle East | 0.00 (0.00, 0.00) | 0.00 (0.00, 0.00) | 0.00 (0.00, 0.00) | 0.00 (0.00, 0.00) | NA | NA |
| South Asia | 0.00 (0.00, 0.00) | 0.00 (0.00, 0.00) | 0.00 (0.00, 0.00) | 0.00 (0.00, 0.00) | NA | NA |
| Central Sub-Saharan Africa | 0.00 (0.00, 0.00) | 0.00 (0.00, 0.00) | 0.00 (0.00, 0.00) | 0.00 (0.00, 0.00) | NA | NA |
| Eastern Sub-Saharan Africa | 0.00 (0.00, 0.00) | 0.00 (0.00, 0.00) | 0.00 (0.00, 0.00) | 0.00 (0.00, 0.00) | NA | NA |
| Southern Sub-Saharan Africa | 0.00 (0.00, 0.00) | 0.00 (0.00, 0.00) | 0.00 (0.00, 0.00) | 0.00 (0.00, 0.00) | NA | NA |
| Western Sub-Saharan Africa | 0.00 (0.00, 0.00) | 0.00 (0.00, 0.00) | 0.00 (0.00, 0.00) | 0.00 (0.00, 0.00) | NA | NA |

DALYs disability-adjusted life-years, ASDRs age-standardized DALYs rates, AAPCs average annual percentage changes, UI uncertainty intervals, CI confidence interval, SDI Socio-demographic Index

**Table E** ASPRs in 2021 for nine arthropod-borne diseases in 204 countries and territories

| **Location** | **Malaria** | **Chagas disease** | **Leishmaniasis** | **Lymphatic filariasis** | **Onchocerciasis** | **Dengue** | **Yellow fever** | **Zika virus** |  |
| --- | --- | --- | --- | --- | --- | --- | --- | --- | --- |
| Afghanistan | 577.62  (517.31, 642.1) | 0  (0, 0) | 3577.88  (3120.5, 4174.07) | 0  (0, 0) | 0  (0, 0) | 0.12  (0, 0.77) | 0  (0, 0) | 0  (0, 0) |  |
| Albania | 0  (0, 0) | 0  (0, 0) | 5.52  (4.3, 7.41) | 0  (0, 0) | 0  (0, 0) | 0  (0, 0) | 0  (0, 0) | 0  (0, 0) |  |
| Algeria | 0  (0, 0) | 0  (0, 0) | 430.45  (356.15, 554.89) | 0  (0, 0) | 0  (0, 0) | 0  (0, 0) | 0  (0, 0) | 0  (0, 0) |  |
| American Samoa | 0  (0, 0) | 0  (0, 0) | 0  (0, 0) | 4165.65  (1753.5, 9489.17) | 0  (0, 0) | 136.34  (27.82, 441.36) | 0  (0, 0) | 0  (0, 0) |  |
| Andorra | 0  (0, 0) | 16.49  (11.12, 23.71) | 0  (0, 0) | 0  (0, 0) | 0  (0, 0) | 0  (0, 0) | 0  (0, 0) | 0  (0, 0) |  |
| Angola | 15900.97  (9265.4, 23508.9) | 0  (0, 0) | 0.26  (0.04, 0.8) | 1146.99  (344.87, 3591.49) | 622.84  (516.93, 758.54) | 12.91  (0.43, 82.83) | 0.46  (0.13, 1.2) | 0  (0, 0) |  |
| Antigua and Barbuda | 0  (0, 0) | 0.99  (0.34, 2.6) | 0  (0, 0) | 0  (0, 0) | 0  (0, 0) | 3.02  (0.32, 8.57) | 0  (0, 0) | 0.56  (0.03, 3.67) |  |
| Argentina | 0  (0, 0) | 1549.23  (1347.75, 1771.86) | 19.31  (15.44, 26.37) | 0  (0, 0) | 0  (0, 0) | 10.44  (2.2, 24.98) | 0.04  (0.01, 0.13) | 0  (0, 0.02) |  |
| Armenia | 0  (0, 0) | 0  (0, 0) | 43.75  (18.43, 92.62) | 0  (0, 0) | 0  (0, 0) | 0  (0, 0) | 0  (0, 0) | 0  (0, 0) |  |
| Australia | 0  (0, 0) | 5.54  (4.66, 6.49) | 0  (0, 0) | 0  (0, 0) | 0  (0, 0) | 4.24  (1.17, 11.13) | 0  (0, 0) | 0  (0, 0) |  |
| Austria | 0  (0, 0) | 1.89  (1.6, 2.24) | 0.06  (0.01, 0.17) | 0  (0, 0) | 0  (0, 0) | 0  (0, 0) | 0  (0, 0) | 0  (0, 0) |  |
| Azerbaijan | 0  (0, 0) | 0  (0, 0) | 28.12  (16.92, 50.17) | 0  (0, 0) | 0  (0, 0) | 0  (0, 0) | 0  (0, 0) | 0  (0, 0) |  |
| Bahamas | 0  (0, 0) | 1.81  (1.19, 2.62) | 0  (0, 0) | 0  (0, 0) | 0  (0, 0) | 81.92  (0.75, 292.35) | 0  (0, 0) | 0.31  (0.02, 2.49) |  |
| Bahrain | 0  (0, 0) | 0  (0, 0) | 0  (0, 0) | 0  (0, 0) | 0  (0, 0) | 0  (0, 0) | 0  (0, 0) | 0  (0, 0) |  |
| Bangladesh | 119.67  (108.82, 130.58) | 0  (0, 0) | 0.04  (0.02, 0.06) | 322.58  (268.86, 438.37) | 0  (0, 0) | 25.12  (3.89, 53.96) | 0  (0, 0) | 0  (0, 0) |  |
| Barbados | 0  (0, 0) | 0.06  (0.05, 0.08) | 0  (0, 0) | 0  (0, 0) | 0  (0, 0) | 115.97  (12.36, 325.79) | 0  (0, 0) | 0.29  (0.02, 1.97) |  |
| Belarus | 0  (0, 0) | 0  (0, 0) | 0  (0, 0) | 0  (0, 0) | 0  (0, 0) | 0  (0, 0) | 0  (0, 0) | 0  (0, 0) |  |
| Belgium | 0  (0, 0) | 0.82  (0.67, 1) | 0  (0, 0) | 0  (0, 0) | 0  (0, 0) | 0  (0, 0) | 0  (0, 0) | 0  (0, 0) |  |
| Belize | 0  (0, 0) | 13.91  (9.82, 19.17) | 113.36  (80.32, 170.26) | 0  (0, 0) | 0  (0, 0) | 28.48  (2.71, 82.73) | 0  (0, 0) | 1.8  (0.1, 11.35) |  |
| Benin | 26381.29  (18140.24, 35156.38) | 0  (0, 0) | 0  (0, 0) | 796.87  (526.51, 1245.71) | 298.21  (209.03, 427.62) | 29.6  (0.55, 214.07) | 0.17  (0.05, 0.43) | 0  (0, 0) |  |
| Bermuda | 0  (0, 0) | 0.01  (0, 0.01) | 0  (0, 0) | 0  (0, 0) | 0  (0, 0) | 0  (0, 0) | 0  (0, 0) | 0  (0, 0) |  |
| Bhutan | 95.53  (87.15, 105.13) | 0  (0, 0) | 0.13  (0.05, 0.28) | 0  (0, 0) | 0  (0, 0) | 3.06  (0.08, 9.11) | 0  (0, 0) | 0  (0, 0) |  |
| Bolivia  (Plurinational State of) | 205.34  (177.87, 236.2) | 4132.52  (3749.15, 4522.73) | 836.53  (664.92, 1168.75) | 0  (0, 0) | 0  (0, 0) | 41.65  (7.75, 102.73) | 0.01  (0, 0.03) | 0.1  (0.02, 0.35) |  |
| Bosnia and Herzegovina | 0  (0, 0) | 0  (0, 0) | 3.61  (2.39, 5.32) | 0  (0, 0) | 0  (0, 0) | 0  (0, 0) | 0  (0, 0) | 0  (0, 0) |  |
| Botswana | 46.67  (14.96, 53.74) | 0  (0, 0) | 0  (0, 0) | 0  (0, 0) | 0  (0, 0) | 0  (0, 0) | 0  (0, 0) | 0  (0, 0) |  |
| Brazil | 131.2  (96.06, 174.15) | 841.06  (723.96, 958.22) | 301.65  (273.35, 335.98) | 8.21  (6.98, 13.99) | 0.1  (0.01, 0.42) | 350.38  (104.95, 719.72) | 0  (0, 0) | 0.64  (0.28, 1.6) |  |
| Brunei Darussalam | 0  (0, 0) | 0  (0, 0) | 0  (0, 0) | 946.3  (223.76, 5124.79) | 0  (0, 0) | 7.79  (1.96, 21.66) | 0  (0, 0) | 0  (0, 0) |  |
| Bulgaria | 0  (0, 0) | 0  (0, 0) | 3.93  (2.65, 5.82) | 0  (0, 0) | 0  (0, 0) | 0  (0, 0) | 0  (0, 0) | 0  (0, 0) |  |
| Burkina Faso | 25118.45  (15382.65, 36446.31) | 0  (0, 0) | 323.48  (271.89, 402.71) | 656.65  (361.66, 1417.75) | 1.55  (0.93, 2.4) | 8.6  (0.43, 50.26) | 0.27  (0.07, 0.72) | 0  (0, 0) |  |
| Burundi | 19828.82  (9919.98, 32161.62) | 0  (0, 0) | 0  (0, 0) | 0  (0, 0) | 2482.03  (2231.77, 2763.24) | 1.05  (0.02, 6.72) | 0.79  (0.21, 2.12) | 0  (0, 0) |  |
| Cabo Verde | 61.72  (55.37, 68.44) | 0  (0, 0) | 0  (0, 0) | 0  (0, 0) | 0  (0, 0) | 456.42  (0.98, 2435.52) | 0  (0, 0) | 0  (0, 0) |  |
| Cambodia | 383.46  (349.14, 419.16) | 0  (0, 0) | 0  (0, 0) | 120.04  (2.03, 779.59) | 0  (0, 0) | 14.9  (2.93, 34.36) | 0  (0, 0) | 0  (0, 0) |  |
| Cameroon | 15428.41  (9771.14, 22980.61) | 0  (0, 0) | 48.32  (36.69, 65.6) | 305.78  (221.32, 463.82) | 4238.15  (3936.5, 4574.01) | 20.3  (0.61, 138.3) | 0.03  (0.01, 0.09) | 0  (0, 0) |  |
| Canada | 0  (0, 0) | 9.84  (8.29, 11.52) | 0  (0, 0) | 0  (0, 0) | 0  (0, 0) | 0  (0, 0) | 0  (0, 0) | 0  (0, 0) |  |
| Central African Republic | 22834.91  (10922.44, 49027.43) | 0  (0, 0) | 27.91  (20.73, 38.26) | 4427.9  (1118.06, 13869.14) | 6890.8  (6271.19, 7536.75) | 14.63  (0.06, 109.68) | 0.13  (0.03, 0.36) | 0  (0, 0) |  |
| Chad | 10398.03  (5123.69, 20899.45) | 0  (0, 0) | 19.16  (14.64, 25.32) | 791.21  (304.72, 2443.96) | 1985.57  (1633.73, 2353.37) | 2.66  (0.06, 21.02) | 0.29  (0.08, 0.76) | 0  (0, 0) |  |
| Chile | 0  (0, 0) | 1007.87  (873.22, 1149.23) | 0  (0, 0) | 0  (0, 0) | 0  (0, 0) | 0  (0, 0) | 0  (0, 0) | 0  (0, 0) |  |
| China | 0  (0, 0) | 0  (0, 0) | 0.8  (0.37, 1.81) | 0  (0, 0) | 0  (0, 0) | 0.12  (0.03, 0.37) | 0  (0, 0) | 0  (0, 0) |  |
| Colombia | 333.25  (242.21, 442.78) | 241.98  (206.74, 282.04) | 379.45  (303.04, 507.03) | 0  (0, 0) | 0  (0, 0) | 89.33  (19.89, 190.01) | 0  (0, 0.01) | 0.12  (0.02, 0.48) |  |
| Comoros | 479.17  (33.14, 595.59) | 0  (0, 0) | 0  (0, 0) | 3800.81  (585.64, 18418.36) | 0  (0, 0) | 707.55  (0.13, 4795.15) | 0  (0, 0) | 0  (0, 0) |  |
| Congo | 16135.5  (9118.54, 28398.71) | 0  (0, 0) | 0  (0, 0) | 939.79  (321.61, 2570.71) | 608.44  (467.08, 785.92) | 18.22  (0.76, 143.75) | 0.33  (0.09, 0.88) | 0  (0, 0) |  |
| Cook Islands | 0  (0, 0) | 0  (0, 0) | 0  (0, 0) | 0  (0, 0) | 0  (0, 0) | 6.26  (0.15, 25.13) | 0  (0, 0) | 0  (0, 0) |  |
| Costa Rica | 30.32  (30.23, 30.39) | 408.48  (333.12, 485.65) | 450.46  (370.52, 582.16) | 0  (0, 0) | 0  (0, 0) | 158.36  (21.39, 413.21) | 0  (0, 0) | 0.19  (0.03, 0.72) |  |
| Croatia | 0  (0, 0) | 0  (0, 0) | 1.79  (1.28, 2.64) | 0  (0, 0) | 0  (0, 0) | 0  (0, 0) | 0  (0, 0) | 0  (0, 0) |  |
| Cuba | 0  (0, 0) | 0.16  (0.12, 0.22) | 0  (0, 0) | 0  (0, 0) | 0  (0, 0) | 10.11  (1.75, 27.14) | 0  (0, 0) | 0.78  (0.14, 2.74) |  |
| Cyprus | 0  (0, 0) | 0  (0, 0) | 1.35  (0.99, 1.91) | 0  (0, 0) | 0  (0, 0) | 0  (0, 0) | 0  (0, 0) | 0  (0, 0) |  |
| Czechia | 0  (0, 0) | 0  (0, 0) | 0  (0, 0) | 0  (0, 0) | 0  (0, 0) | 0  (0, 0) | 0  (0, 0) | 0  (0, 0) |  |
| Côte d'Ivoire | 19308.97  (8985.29, 37072.39) | 0  (0, 0) | 15.6  (12.03, 20.24) | 7684.98  (2924.26, 17988.83) | 1.23  (0.59, 2.03) | 12.92  (0.41, 96.9) | 0.07  (0.02, 0.18) | 0  (0, 0) |  |
| Democratic People's Republic of Korea | 128.28  (114.28, 143.15) | 0  (0, 0) | 0  (0, 0) | 0  (0, 0) | 0  (0, 0) | 0  (0, 0) | 0  (0, 0) | 0  (0, 0) |  |
| Democratic Republic of the Congo | 23674.27  (17397.97, 32319.3) | 0  (0, 0) | 22.58  (16.69, 31.46) | 2223.67  (1055.09, 4441.76) | 14123.52  (13227.2, 15113.21) | 9.15  (0.18, 65.6) | 0.1  (0.03, 0.27) | 0  (0, 0) |  |
| Denmark | 0  (0, 0) | 3  (2.53, 3.56) | 0  (0, 0) | 0  (0, 0) | 0  (0, 0) | 0  (0, 0) | 0  (0, 0) | 0  (0, 0) |  |
| Djibouti | 3387.96  (3114.09, 3667.82) | 0  (0, 0) | 16.69  (11.96, 24.05) | 0  (0, 0) | 0  (0, 0) | 36.22  (0.29, 272.53) | 0  (0, 0) | 0  (0, 0) |  |
| Dominica | 0  (0, 0) | 0.68  (0.01, 2.72) | 0  (0, 0) | 0  (0, 0) | 0  (0, 0) | 12.15  (1.02, 35.21) | 0  (0, 0) | 0.57  (0.03, 3.95) |  |
| Dominican Republic | 2.8  (0.65, 11.64) | 7.06  (6.09, 8.17) | 127.19  (91.6, 181.81) | 1338.6  (361.82, 4758.35) | 0  (0, 0) | 32.24  (5.23, 79.71) | 0  (0, 0) | 0.01  (0, 0.05) |  |
| Ecuador | 140.05  (122.95, 159.21) | 806.03  (673.11, 952.39) | 303.57  (227.42, 448.74) | 0  (0, 0) | 0  (0, 0) | 31.12  (6.82, 70.96) | 0.01  (0, 0.03) | 0  (0, 0.02) |  |
| Egypt | 0  (0, 0) | 0  (0, 0) | 314.59  (163.58, 592.09) | 581.81  (62.68, 2526.12) | 0  (0, 0) | 1.54  (0.1, 8.19) | 0  (0, 0) | 0  (0, 0) |  |
| El Salvador | 0  (0, 0) | 588.48  (494.81, 696.22) | 251.06  (113.98, 475.35) | 0  (0, 0) | 0  (0, 0) | 88.59  (15.23, 214.76) | 0  (0, 0) | 2.68  (0.42, 9.78) |  |
| Equatorial Guinea | 16174.44  (7084.13, 26412.93) | 0  (0, 0) | 0  (0, 0) | 1316.66  (321.02, 5762.15) | 2781.64  (2657.26, 2915.01) | 7.01  (0.18, 50.88) | 0.12  (0.03, 0.35) | 0  (0, 0) |  |
| Eritrea | 420.02  (406.47, 433.95) | 0  (0, 0) | 24.18  (17.22, 35.5) | 424.44  (200.19, 1118.15) | 0  (0, 0) | 0.6  (0, 4.3) | 0.06  (0.02, 0.17) | 0  (0, 0) |  |
| Estonia | 0  (0, 0) | 0  (0, 0) | 0  (0, 0) | 0  (0, 0) | 0  (0, 0) | 0  (0, 0) | 0  (0, 0) | 0  (0, 0) |  |
| Eswatini | 39.44  (10.18, 50.32) | 0  (0, 0) | 0  (0, 0) | 0  (0, 0) | 0  (0, 0) | 0  (0, 0) | 0  (0, 0) | 0  (0, 0) |  |
| Ethiopia | 1188.59  (1027.01, 1353.2) | 0  (0, 0) | 25.12  (18.71, 34.52) | 607.26  (311.74, 1295.81) | 547.77  (468.81, 642.03) | 0.89  (0.01, 4.48) | 0.26  (0.08, 0.61) | 0  (0, 0) |  |
| Fiji | 0  (0, 0) | 0  (0, 0) | 0  (0, 0) | 9487.12  (2655.77, 26045.32) | 0  (0, 0) | 99.95  (29.41, 238.46) | 0  (0, 0) | 0  (0, 0) |  |
| Finland | 0  (0, 0) | 1.45  (1.2, 1.73) | 0  (0, 0) | 0  (0, 0) | 0  (0, 0) | 0  (0, 0) | 0  (0, 0) | 0  (0, 0) |  |
| France | 0  (0, 0) | 2.64  (2.28, 3.05) | 0.69  (0.49, 1.02) | 0  (0, 0) | 0  (0, 0) | 0  (0, 0) | 0  (0, 0) | 0  (0, 0) |  |
| Gabon | 17243.5  (6452.62, 35666.44) | 0  (0, 0) | 0  (0, 0) | 1414.34  (335.96, 5706.92) | 0  (0, 0) | 27.29  (0.66, 186.75) | 0.24  (0.07, 0.61) | 0  (0, 0) |  |
| Gambia | 1952.01  (1672.27, 2269.32) | 0  (0, 0) | 11.26  (8.41, 14.79) | 0  (0, 0) | 0  (0, 0) | 28.19  (1.28, 178.06) | 0.15  (0.04, 0.39) | 0  (0, 0) |  |
| Georgia | 0  (0, 0) | 0  (0, 0) | 20.09  (9.86, 38.1) | 0  (0, 0) | 0  (0, 0) | 0  (0, 0) | 0  (0, 0) | 0  (0, 0) |  |
| Germany | 0  (0, 0) | 1.22  (1.05, 1.41) | 0  (0, 0) | 0  (0, 0) | 0  (0, 0) | 0  (0, 0) | 0  (0, 0) | 0  (0, 0) |  |
| Ghana | 9329.13  (7426.37, 11920.74) | 0  (0, 0) | 143.87  (107.61, 194.25) | 804.11  (319.3, 2155.21) | 10.52  (7.43, 14.81) | 41.3  (1.09, 306.22) | 0.06  (0.02, 0.15) | 0  (0, 0) |  |
| Greece | 0  (0, 0) | 0.32  (0.24, 0.42) | 1.01  (0.76, 1.4) | 0  (0, 0) | 0  (0, 0) | 0  (0, 0) | 0  (0, 0) | 0  (0, 0) |  |
| Greenland | 0  (0, 0) | 0  (0, 0) | 0  (0, 0) | 0  (0, 0) | 0  (0, 0) | 0  (0, 0) | 0  (0, 0) | 0  (0, 0) |  |
| Grenada | 0  (0, 0) | 15.05  (8.63, 23.12) | 0  (0, 0) | 0  (0, 0) | 0  (0, 0) | 20.2  (6.63, 50.76) | 0  (0, 0) | 0.04  (0, 0.22) |  |
| Guam | 0  (0, 0) | 0  (0, 0) | 0  (0, 0) | 0  (0, 0) | 0  (0, 0) | 0  (0, 0) | 0  (0, 0) | 0  (0, 0) |  |
| Guatemala | 0.01  (0.01, 0.02) | 714.56  (594.42, 845.45) | 403.15  (249.64, 683.29) | 0  (0, 0) | 0  (0, 0) | 13.54  (2.73, 30.6) | 0  (0, 0) | 0.49  (0.09, 1.74) |  |
| Guinea | 22198.55  (9954.28, 32843.63) | 0  (0, 0) | 18.71  (14.01, 25.08) | 743.03  (302.91, 2038.61) | 218.08  (135.72, 328.73) | 6.27  (0.01, 41.14) | 0.29  (0.08, 0.79) | 0  (0, 0) |  |
| Guinea-Bissau | 4755.88  (1339.87, 12868.17) | 0  (0, 0) | 19.13  (14.66, 25.94) | 1244.14  (294.35, 5350.61) | 0.37  (0.2, 0.52) | 24.59  (0.26, 172.81) | 0.2  (0.05, 0.55) | 0  (0, 0) |  |
| Guyana | 3366.3  (2988.76, 3783.48) | 4.44  (3.47, 5.64) | 309.89  (221.43, 456.15) | 16158.49  (5921.82, 33023.58) | 0  (0, 0) | 14.06  (1.14, 41.62) | 0.05  (0.01, 0.13) | 0.02  (0, 0.16) |  |
| Haiti | 268.5  (66.58, 732.75) | 0  (0, 0) | 0  (0, 0) | 1093.1  (568.88, 2308.35) | 0  (0, 0) | 25.05  (0.1, 137.96) | 0  (0, 0) | 0  (0, 0.02) |  |
| Honduras | 94.09  (0.06, 197.77) | 888.9  (742.23, 1057.83) | 619.99  (476.5, 865.29) | 0  (0, 0) | 0  (0, 0) | 51.76  (4.74, 149.36) | 0  (0, 0) | 0.29  (0.05, 1) |  |
| Hungary | 0  (0, 0) | 0  (0, 0) | 0  (0, 0) | 0  (0, 0) | 0  (0, 0) | 0  (0, 0) | 0  (0, 0) | 0  (0, 0) |  |
| Iceland | 0  (0, 0) | 1.57  (0.97, 2.49) | 0  (0, 0) | 0  (0, 0) | 0  (0, 0) | 0  (0, 0) | 0  (0, 0) | 0  (0, 0) |  |
| India | 296.74  (226.58, 426.49) | 0  (0, 0) | 0.34  (0.26, 0.45) | 2326.06  (2022.48, 2700.89) | 0  (0, 0) | 119.54  (5.97, 258.82) | 0  (0, 0) | 0  (0, 0) |  |
| Indonesia | 402.73  (306.58, 479.52) | 0  (0, 0) | 0  (0, 0) | 1476.16  (605.38, 3845.58) | 0  (0, 0) | 53.64  (20.99, 114.25) | 0  (0, 0) | 0  (0, 0) |  |
| Iran  (Islamic Republic of) | 2.96  (0.64, 15.69) | 0  (0, 0) | 608.26  (472.33, 887.67) | 0  (0, 0) | 0  (0, 0) | 0  (0, 0) | 0  (0, 0) | 0  (0, 0) |  |
| Iraq | 0  (0, 0) | 0  (0, 0) | 821.59  (705.54, 971.52) | 0  (0, 0) | 0  (0, 0) | 0  (0, 0) | 0  (0, 0) | 0  (0, 0) |  |
| Ireland | 0  (0, 0) | 23.75  (20.49, 27.01) | 0  (0, 0) | 0  (0, 0) | 0  (0, 0) | 0  (0, 0) | 0  (0, 0) | 0  (0, 0) |  |
| Israel | 0  (0, 0) | 11.6  (9.96, 13.39) | 9.01  (7.47, 11.17) | 0  (0, 0) | 0  (0, 0) | 0  (0, 0) | 0  (0, 0) | 0  (0, 0) |  |
| Italy | 0  (0, 0) | 0  (0, 0) | 1.05  (0.75, 1.58) | 0  (0, 0) | 0  (0, 0) | 0  (0, 0) | 0  (0, 0) | 0  (0, 0) |  |
| Jamaica | 0  (0, 0) | 0.46  (0.36, 0.57) | 0  (0, 0) | 0  (0, 0) | 0  (0, 0) | 9.18  (1.93, 22.13) | 0  (0, 0) | 0.04  (0, 0.26) |  |
| Japan | 0  (0, 0) | 3.8  (3.23, 4.4) | 0  (0, 0) | 0  (0, 0) | 0  (0, 0) | 0  (0, 0) | 0  (0, 0) | 0  (0, 0) |  |
| Jordan | 0  (0, 0) | 0  (0, 0) | 43.86  (33.39, 62.68) | 0  (0, 0) | 0  (0, 0) | 0.88  (0.03, 3.41) | 0  (0, 0) | 0  (0, 0) |  |
| Kazakhstan | 0  (0, 0) | 0  (0, 0) | 18.6  (12.97, 28.97) | 0  (0, 0) | 0  (0, 0) | 0  (0, 0) | 0  (0, 0) | 0  (0, 0) |  |
| Kenya | 3020.42  (2591.44, 3405.15) | 0  (0, 0) | 1.22  (0.87, 1.78) | 1054.81  (397.58, 3064.43) | 0  (0, 0) | 19.69  (0.33, 113.67) | 0.03  (0.01, 0.07) | 0  (0, 0) |  |
| Kiribati | 0  (0, 0) | 0  (0, 0) | 0  (0, 0) | 1455.49  (297.24, 4976.06) | 0  (0, 0) | 364.9  (25.6, 1507.08) | 0  (0, 0) | 0  (0, 0) |  |
| Kuwait | 0  (0, 0) | 0  (0, 0) | 26.77  (16.36, 45.36) | 0  (0, 0) | 0  (0, 0) | 3.42  (0.22, 12.34) | 0  (0, 0) | 0  (0, 0) |  |
| Kyrgyzstan | 0  (0, 0) | 0  (0, 0) | 54.34  (24.01, 111.34) | 0  (0, 0) | 0  (0, 0) | 0  (0, 0) | 0  (0, 0) | 0  (0, 0) |  |
| Lao People's Democratic Republic | 368.19  (332.85, 405.87) | 0  (0, 0) | 0  (0, 0) | 510.73  (214.88, 1773.68) | 0  (0, 0) | 19.36  (4.62, 42.72) | 0  (0, 0) | 0  (0, 0) |  |
| Latvia | 0  (0, 0) | 0  (0, 0) | 0  (0, 0) | 0  (0, 0) | 0  (0, 0) | 0  (0, 0) | 0  (0, 0) | 0  (0, 0) |  |
| Lebanon | 0  (0, 0) | 0  (0, 0) | 40.55  (23.29, 71.96) | 0  (0, 0) | 0  (0, 0) | 1.92  (0.09, 6.31) | 0  (0, 0) | 0  (0, 0) |  |
| Lesotho | 0  (0, 0) | 0  (0, 0) | 0  (0, 0) | 0  (0, 0) | 0  (0, 0) | 0  (0, 0) | 0  (0, 0) | 0  (0, 0) |  |
| Liberia | 29248.89  (13845.02, 47808.36) | 0  (0, 0) | 0  (0, 0) | 11813.12  (9265.72, 15003.63) | 21423.87  (18233.24, 25347.44) | 33.08  (0.63, 210.33) | 0.41  (0.12, 1.07) | 0  (0, 0) |  |
| Libya | 0  (0, 0) | 0  (0, 0) | 683.78  (529.59, 937.42) | 0  (0, 0) | 0  (0, 0) | 0  (0, 0) | 0  (0, 0) | 0  (0, 0) |  |
| Lithuania | 0  (0, 0) | 0  (0, 0) | 0  (0, 0) | 0  (0, 0) | 0  (0, 0) | 0  (0, 0) | 0  (0, 0) | 0  (0, 0) |  |
| Luxembourg | 0  (0, 0) | 2.01  (1.38, 2.9) | 0  (0, 0) | 0  (0, 0) | 0  (0, 0) | 0  (0, 0) | 0  (0, 0) | 0  (0, 0) |  |
| Madagascar | 4695.62  (2728.45, 7135.03) | 0  (0, 0) | 0  (0, 0) | 1049.81  (420.2, 2510.21) | 0  (0, 0) | 2.02  (0.01, 15.08) | 0  (0, 0) | 0  (0, 0) |  |
| Malawi | 13217.21  (6744.04, 24519.8) | 0  (0, 0) | 17.25  (12.55, 25.41) | 440.9  (305.62, 701.2) | 594.1  (453.39, 760.13) | 1.79  (0.02, 12.41) | 0  (0, 0) | 0  (0, 0) |  |
| Malaysia | 0.13  (0.1, 0.16) | 0  (0, 0) | 0  (0, 0) | 1033.33  (321.49, 3771.44) | 0  (0, 0) | 154.6  (50.13, 388.25) | 0  (0, 0) | 0  (0, 0) |  |
| Maldives | 0  (0, 0) | 0  (0, 0) | 0  (0, 0) | 401.63  (180.39, 955.57) | 0  (0, 0) | 301.55  (0, 1102.61) | 0  (0, 0) | 0  (0, 0) |  |
| Mali | 16025.82  (9895.09, 24537.44) | 0  (0, 0) | 42.28  (30.89, 58.46) | 1548.32  (465.64, 4959.48) | 1.82  (1.09, 2.71) | 3.49  (0.1, 32.21) | 0.1  (0.03, 0.29) | 0  (0, 0) |  |
| Malta | 0  (0, 0) | 0  (0, 0) | 2.38  (1.84, 3.11) | 0  (0, 0) | 0  (0, 0) | 0  (0, 0) | 0  (0, 0) | 0  (0, 0) |  |
| Marshall Islands | 0  (0, 0) | 0  (0, 0) | 0  (0, 0) | 151.63  (141.15, 164.16) | 0  (0, 0) | 618.37  (55.46, 2913.41) | 0  (0, 0) | 0  (0, 0) |  |
| Mauritania | 766.7  (630.18, 925.87) | 0  (0, 0) | 14.48  (10.75, 19.11) | 0  (0, 0) | 0  (0, 0) | 2.47  (0.14, 19.85) | 0.12  (0.03, 0.32) | 0  (0, 0) |  |
| Mauritius | 0  (0, 0) | 0  (0, 0) | 0  (0, 0) | 0  (0, 0) | 0  (0, 0) | 60.74  (1.69, 251.52) | 0  (0, 0) | 0  (0, 0) |  |
| Mexico | 6.85  (4.26, 10.08) | 880.27  (733, 1041.26) | 7.99  (6.55, 10.06) | 0  (0, 0) | 0  (0, 0) | 64.92  (45.66, 84.53) | 0  (0, 0) | 0.36  (0.02, 2.47) |  |
| Micronesia  (Federated States of) | 0  (0, 0) | 0  (0, 0) | 0  (0, 0) | 2284.47  (387.61, 15628.36) | 0  (0, 0) | 25.83  (3.38, 80.92) | 0  (0, 0) | 0  (0, 0) |  |
| Monaco | 0  (0, 0) | 0  (0, 0) | 1.75  (0.68, 4.31) | 0  (0, 0) | 0  (0, 0) | 0  (0, 0) | 0  (0, 0) | 0  (0, 0) |  |
| Mongolia | 0  (0, 0) | 0  (0, 0) | 0  (0, 0) | 0  (0, 0) | 0  (0, 0) | 0  (0, 0) | 0  (0, 0) | 0  (0, 0) |  |
| Montenegro | 0  (0, 0) | 0  (0, 0) | 0.29  (0.14, 0.56) | 0  (0, 0) | 0  (0, 0) | 0  (0, 0) | 0  (0, 0) | 0  (0, 0) |  |
| Morocco | 0  (0, 0) | 0  (0, 0) | 197.69  (166.35, 240.33) | 0  (0, 0) | 0  (0, 0) | 0  (0, 0) | 0  (0, 0) | 0  (0, 0) |  |
| Mozambique | 20953.34  (13694.62, 28503.4) | 0  (0, 0) | 0  (0, 0) | 998.67  (417.93, 2361.26) | 0  (0, 0) | 6.71  (0.01, 49.65) | 0  (0, 0) | 0  (0, 0) |  |
| Myanmar | 716.5  (0.35, 822.76) | 0  (0, 0) | 0  (0, 0) | 1640.53  (369.37, 6331.4) | 0  (0, 0) | 4.7  (0.9, 10.57) | 0  (0, 0) | 0  (0, 0) |  |
| Namibia | 242.92  (11.9, 342.01) | 0  (0, 0) | 15.65  (11.12, 22.17) | 0  (0, 0) | 0  (0, 0) | 0  (0, 0) | 0  (0, 0) | 0  (0, 0) |  |
| Nauru | 0  (0, 0) | 0  (0, 0) | 0  (0, 0) | 0  (0, 0) | 0  (0, 0) | 63.24  (0, 264.45) | 0  (0, 0) | 0  (0, 0) |  |
| Nepal | 91.9  (83.03, 101.01) | 0  (0, 0) | 19.33  (12.03, 33.12) | 2527.9  (1959.66, 3423.1) | 0  (0, 0) | 52.48  (0.5, 170.73) | 0  (0, 0) | 0  (0, 0) |  |
| Netherlands | 0  (0, 0) | 3.62  (3.08, 4.19) | 0  (0, 0) | 0  (0, 0) | 0  (0, 0) | 0  (0, 0) | 0  (0, 0) | 0  (0, 0) |  |
| New Zealand | 0  (0, 0) | 0  (0, 0) | 0  (0, 0) | 0  (0, 0) | 0  (0, 0) | 0  (0, 0) | 0  (0, 0) | 0  (0, 0) |  |
| Nicaragua | 602.73  (1.58, 1929.93) | 655.93  (548.36, 783.96) | 1277.63  (1059.09, 1555.35) | 0  (0, 0) | 0  (0, 0) | 67.28  (21.94, 175.94) | 0  (0, 0) | 0.63  (0.1, 2.19) |  |
| Niger | 17766.75  (8936.79, 29073.23) | 0  (0, 0) | 61.17  (46.41, 80.06) | 1518.44  (735.49, 3711.05) | 0.93  (0.5, 2.04) | 2.12  (0.06, 18.46) | 0.52  (0.14, 1.53) | 0  (0, 0) |  |
| Nigeria | 19855.45  (14265.43, 24608.04) | 0  (0, 0) | 10.44  (7.59, 14.56) | 1503.42  (629.43, 3027.04) | 1503.54  (1210.15, 1846.31) | 44.41  (1.29, 152.99) | 0.28  (0.11, 0.58) | 0  (0, 0) |  |
| Niue | 0  (0, 0) | 0  (0, 0) | 0  (0, 0) | 1462.58  (424.25, 4717.93) | 0  (0, 0) | 6.84  (1.61, 20.47) | 0  (0, 0) | 0  (0, 0) |  |
| North Macedonia | 0  (0, 0) | 0  (0, 0) | 4.63  (3.19, 6.99) | 0  (0, 0) | 0  (0, 0) | 0  (0, 0) | 0  (0, 0) | 0  (0, 0) |  |
| Northern Mariana Islands | 0  (0, 0) | 0  (0, 0) | 0  (0, 0) | 0  (0, 0) | 0  (0, 0) | 44.71  (4.46, 155.24) | 0  (0, 0) | 0  (0, 0) |  |
| Norway | 0  (0, 0) | 0  (0, 0) | 0  (0, 0) | 0  (0, 0) | 0  (0, 0) | 0  (0, 0) | 0  (0, 0) | 0  (0, 0) |  |
| Oman | 27.39  (7.82, 109.41) | 0  (0, 0) | 16.36  (11.51, 23.84) | 0  (0, 0) | 0  (0, 0) | 1.3  (0.01, 7.73) | 0  (0, 0) | 0  (0, 0) |  |
| Pakistan | 616.97  (487.4, 727.34) | 0  (0, 0) | 169.72  (143.07, 207.27) | 0  (0, 0) | 0  (0, 0) | 66.52  (1.38, 191.37) | 0  (0, 0) | 0  (0, 0) |  |
| Palau | 0  (0, 0) | 0  (0, 0) | 0  (0, 0) | 166.82  (152.81, 187.87) | 0  (0, 0) | 198.61  (0.31, 1076.28) | 0  (0, 0) | 0  (0, 0) |  |
| Palestine | 0  (0, 0) | 0  (0, 0) | 83.25  (56.81, 129.18) | 0  (0, 0) | 0  (0, 0) | 1.2  (0.07, 4.54) | 0  (0, 0) | 0  (0, 0) |  |
| Panama | 262.87  (120.15, 485.27) | 480.92  (401.41, 562.86) | 1204.92  (1077.35, 1358) | 0  (0, 0) | 0  (0, 0) | 69.92  (23.02, 196.66) | 0.01  (0, 0.04) | 0.26  (0.03, 1.11) |  |
| Papua New Guinea | 6673.76  (5936.06, 7447.87) | 0  (0, 0) | 0  (0, 0) | 8717.93  (2917.79, 21797.35) | 0  (0, 0) | 1.96  (0.15, 6.87) | 0  (0, 0) | 0  (0, 0) |  |
| Paraguay | 0  (0, 0) | 465.02  (397.02, 546.87) | 155.48  (124.72, 212.21) | 0  (0, 0) | 0  (0, 0) | 149.71  (38.87, 331.57) | 0.04  (0.01, 0.1) | 0.03  (0, 0.16) |  |
| Peru | 303.58  (16.06, 432.53) | 536.57  (461.4, 618.75) | 581.95  (514.48, 662.73) | 0  (0, 0) | 0  (0, 0) | 35.67  (10.4, 90.96) | 0.07  (0.02, 0.18) | 1.38  (0.32, 4.67) |  |
| Philippines | 7.88  (0.76, 13.34) | 0  (0, 0) | 0  (0, 0) | 882.78  (517.37, 1957.59) | 0  (0, 0) | 66.05  (15.64, 234.1) | 0  (0, 0) | 0  (0, 0) |  |
| Poland | 0  (0, 0) | 0  (0, 0) | 0  (0, 0) | 0  (0, 0) | 0  (0, 0) | 0  (0, 0) | 0  (0, 0) | 0  (0, 0) |  |
| Portugal | 0  (0, 0) | 19.7  (16.81, 22.61) | 1.4  (0.92, 2.2) | 0  (0, 0) | 0  (0, 0) | 0  (0, 0) | 0  (0, 0) | 0  (0, 0) |  |
| Puerto Rico | 0  (0, 0) | 21.59  (18.13, 25.52) | 0  (0, 0) | 0  (0, 0) | 0  (0, 0) | 99.95  (0.19, 439.82) | 0  (0, 0) | 0.1  (0.02, 0.34) |  |
| Qatar | 0  (0, 0) | 0  (0, 0) | 0  (0, 0) | 0  (0, 0) | 0  (0, 0) | 0  (0, 0) | 0  (0, 0) | 0  (0, 0) |  |
| Republic of Korea | 22.72  (12.8, 37.95) | 0  (0, 0) | 0  (0, 0) | 0  (0, 0) | 0  (0, 0) | 0  (0, 0) | 0  (0, 0) | 0  (0, 0) |  |
| Republic of Moldova | 0  (0, 0) | 0  (0, 0) | 0  (0, 0) | 0  (0, 0) | 0  (0, 0) | 0  (0, 0) | 0  (0, 0) | 0  (0, 0) |  |
| Romania | 0  (0, 0) | 0.05  (0.04, 0.06) | 0.01  (0, 0.01) | 0  (0, 0) | 0  (0, 0) | 0  (0, 0) | 0  (0, 0) | 0  (0, 0) |  |
| Russian Federation | 0  (0, 0) | 0  (0, 0) | 0  (0, 0) | 0  (0, 0) | 0  (0, 0) | 0  (0, 0) | 0  (0, 0) | 0  (0, 0) |  |
| Rwanda | 1686.27  (1505.69, 1861.94) | 0  (0, 0) | 0  (0, 0) | 0  (0, 0) | 0  (0, 0) | 0.7  (0.03, 4.97) | 0.06  (0.02, 0.15) | 0  (0, 0) |  |
| Saint Kitts and Nevis | 0  (0, 0) | 0  (0, 0) | 0  (0, 0) | 0  (0, 0) | 0  (0, 0) | 4.07  (0.14, 28.23) | 0  (0, 0) | 0.56  (0.03, 4.4) |  |
| Saint Lucia | 0  (0, 0) | 0.77  (0.11, 1.83) | 0  (0, 0) | 0  (0, 0) | 0  (0, 0) | 14.86  (3.37, 34.88) | 0  (0, 0) | 0.02  (0, 0.13) |  |
| Saint Vincent and the Grenadines | 0  (0, 0) | 0.06  (0.04, 0.08) | 0  (0, 0) | 0  (0, 0) | 0  (0, 0) | 20.13  (0.8, 66.25) | 0  (0, 0) | 0.12  (0.01, 0.9) |  |
| Samoa | 0  (0, 0) | 0  (0, 0) | 0  (0, 0) | 6838.01  (1618.22, 25432.36) | 0  (0, 0) | 33.18  (9.2, 95.88) | 0  (0, 0) | 0  (0, 0) |  |
| San Marino | 0  (0, 0) | 0  (0, 0) | 0  (0, 0) | 0  (0, 0) | 0  (0, 0) | 0  (0, 0) | 0  (0, 0) | 0  (0, 0) |  |
| Sao Tome and Principe | 680.07  (590.83, 778.2) | 0  (0, 0) | 0  (0, 0) | 3053.31  (474.88, 16636.29) | 0  (0, 0) | 46.51  (1.59, 334.99) | 0.12  (0.04, 0.32) | 0  (0, 0) |  |
| Saudi Arabia | 2.4  (2.03, 2.81) | 0  (0, 0) | 438.36  (297.69, 664.02) | 0  (0, 0) | 0  (0, 0) | 0.29  (0.08, 0.83) | 0  (0, 0) | 0  (0, 0) |  |
| Senegal | 1826.55  (1564.77, 2114.38) | 0  (0, 0) | 7.56  (5.68, 10.41) | 605.89  (244.13, 2030.94) | 2.25  (1.69, 3.38) | 4.11  (0.7, 16.33) | 0.08  (0.03, 0.23) | 0  (0, 0) |  |
| Serbia | 0  (0, 0) | 0  (0, 0) | 0.01  (0, 0.04) | 0  (0, 0) | 0  (0, 0) | 0  (0, 0) | 0  (0, 0) | 0  (0, 0) |  |
| Seychelles | 0  (0, 0) | 0  (0, 0) | 0  (0, 0) | 0  (0, 0) | 0  (0, 0) | 735.66  (126.68, 3086.71) | 0  (0, 0) | 0  (0, 0) |  |
| Sierra Leone | 24551.27  (11068.68, 42048.6) | 0  (0, 0) | 0  (0, 0) | 4311.47  (967.99, 13927.42) | 4560.54  (3549.65, 6121.61) | 31.05  (0.67, 225.96) | 0.4  (0.11, 1) | 0  (0, 0) |  |
| Singapore | 0  (0, 0) | 0  (0, 0) | 0  (0, 0) | 0  (0, 0) | 0  (0, 0) | 521.72  (104.44, 1286.16) | 0  (0, 0) | 0  (0, 0) |  |
| Slovakia | 0  (0, 0) | 0  (0, 0) | 0  (0, 0) | 0  (0, 0) | 0  (0, 0) | 0  (0, 0) | 0  (0, 0) | 0  (0, 0) |  |
| Slovenia | 0  (0, 0) | 0  (0, 0) | 2.34  (1.5, 3.75) | 0  (0, 0) | 0  (0, 0) | 0  (0, 0) | 0  (0, 0) | 0  (0, 0) |  |
| Solomon Islands | 7767.78  (7134.77, 8400.02) | 0  (0, 0) | 0  (0, 0) | 0  (0, 0) | 0  (0, 0) | 64.33  (12.14, 195.56) | 0  (0, 0) | 0  (0, 0) |  |
| Somalia | 2898.52  (2104.28, 3838.77) | 0  (0, 0) | 26.2  (18.89, 38.47) | 0  (0, 0) | 0  (0, 0) | 2.42  (0.01, 20.52) | 0.46  (0.12, 1.35) | 0  (0, 0) |  |
| South Africa | 15.09  (3.46, 19.62) | 0  (0, 0) | 0.31  (0.2, 0.49) | 0  (0, 0) | 0  (0, 0) | 0  (0, 0) | 0  (0, 0) | 0  (0, 0) |  |
| South Sudan | 18688.87  (10079.98, 30919.13) | 0  (0, 0) | 28.01  (21.47, 36.55) | 1119.74  (418.36, 2785.32) | 22097.74  (19937.61, 24463.65) | 0.06  (0, 0.31) | 0.44  (0.12, 1.35) | 0  (0, 0) |  |
| Spain | 0  (0, 0) | 55.75  (48.55, 63.13) | 2.17  (1.27, 3.81) | 0  (0, 0) | 0  (0, 0) | 0  (0, 0) | 0  (0, 0) | 0  (0, 0) |  |
| Sri Lanka | 0  (0, 0) | 0  (0, 0) | 55.97  (47.45, 64.69) | 134.46  (98.23, 182.83) | 0  (0, 0) | 126.63  (31.55, 438.89) | 0  (0, 0) | 0  (0, 0) |  |
| Sudan | 4056.67  (2200.27, 7025.37) | 0  (0, 0) | 680.6  (442.69, 1086.21) | 967.56  (266.24, 3582.62) | 195.09  (177.41, 215.21) | 0.97  (0.03, 6.18) | 0.4  (0.1, 1.05) | 0  (0, 0) |  |
| Suriname | 195.93  (2.6, 540.77) | 6.72  (5.26, 8.64) | 1469.19  (1147.45, 1913.31) | 0  (0, 0) | 0  (0, 0) | 17.64  (0.31, 56.41) | 0.08  (0.02, 0.23) | 0.05  (0, 0.22) |  |
| Sweden | 0  (0, 0) | 9.41  (8.11, 10.81) | 0  (0, 0) | 0  (0, 0) | 0  (0, 0) | 0  (0, 0) | 0  (0, 0) | 0  (0, 0) |  |
| Switzerland | 0  (0, 0) | 15.6  (13.45, 17.87) | 0  (0, 0) | 0  (0, 0) | 0  (0, 0) | 0  (0, 0) | 0  (0, 0) | 0  (0, 0) |  |
| Syrian Arab Republic | 0  (0, 0) | 0  (0, 0) | 4734.44  (4143.02, 5554.19) | 0  (0, 0) | 0  (0, 0) | 0.22  (0.02, 0.76) | 0  (0, 0) | 0  (0, 0) |  |
| Taiwan  (Province of China) | 0  (0, 0) | 0  (0, 0) | 16.52  (8.34, 33.26) | 0  (0, 0) | 0  (0, 0) | 8.77  (0.53, 32.15) | 0  (0, 0) | 0  (0, 0) |  |
| Tajikistan | 0  (0, 0) | 0  (0, 0) | 207.78  (98.5, 414.76) | 0  (0, 0) | 0  (0, 0) | 0  (0, 0) | 0  (0, 0) | 0  (0, 0) |  |
| Thailand | 105.51  (0.65, 119.99) | 0  (0, 0) | 1.79  (1.28, 2.7) | 437.85  (23.41, 2185.86) | 0  (0, 0) | 37.37  (10.22, 83.42) | 0  (0, 0) | 0  (0, 0) |  |
| Timor-Leste | 0  (0, 0) | 0  (0, 0) | 0  (0, 0) | 10359.28  (1998.52, 47601.85) | 0  (0, 0) | 13.13  (3.31, 34.82) | 0  (0, 0) | 0  (0, 0) |  |
| Togo | 15114.24  (8596.64, 23451.29) | 0  (0, 0) | 0  (0, 0) | 198.46  (23.67, 842.59) | 31.14  (18.18, 51.11) | 30.67  (0.02, 204.13) | 0.05  (0.01, 0.12) | 0  (0, 0) |  |
| Tokelau | 0  (0, 0) | 0  (0, 0) | 0  (0, 0) | 0  (0, 0) | 0  (0, 0) | 2.35  (0, 10) | 0  (0, 0) | 0  (0, 0) |  |
| Tonga | 0  (0, 0) | 0  (0, 0) | 0  (0, 0) | 450.53  (190.3, 1792.92) | 0  (0, 0) | 854.37  (7.35, 3178.87) | 0  (0, 0) | 0  (0, 0) |  |
| Trinidad and Tobago | 0  (0, 0) | 1.91  (1.27, 2.69) | 0  (0, 0) | 0  (0, 0) | 0  (0, 0) | 34.81  (1.5, 110.1) | 0.01  (0, 0.04) | 0.06  (0, 0.49) |  |
| Tunisia | 0  (0, 0) | 0  (0, 0) | 861.01  (737.97, 1033.3) | 0  (0, 0) | 0  (0, 0) | 0  (0, 0) | 0  (0, 0) | 0  (0, 0) |  |
| Turkmenistan | 0  (0, 0) | 0  (0, 0) | 910.81  (699.03, 1155.28) | 0  (0, 0) | 0  (0, 0) | 0  (0, 0) | 0  (0, 0) | 0  (0, 0) |  |
| Tuvalu | 0  (0, 0) | 0  (0, 0) | 0  (0, 0) | 0  (0, 0) | 0  (0, 0) | 26.63  (0, 120.36) | 0  (0, 0) | 0  (0, 0) |  |
| T眉rkiye | 0  (0, 0) | 0  (0, 0) | 106.81  (75.23, 162.83) | 0  (0, 0) | 0  (0, 0) | 0  (0, 0) | 0  (0, 0) | 0  (0, 0) |  |
| Uganda | 17069.25  (13612.06, 21130.27) | 0  (0, 0) | 14.83  (10.5, 21.52) | 317  (206.38, 627.2) | 946.9  (788.6, 1111.04) | 2.92  (0.03, 22.11) | 0.1  (0.03, 0.26) | 0  (0, 0) |  |
| Ukraine | 0  (0, 0) | 0  (0, 0) | 0  (0, 0) | 0  (0, 0) | 0  (0, 0) | 0  (0, 0) | 0  (0, 0) | 0  (0, 0) |  |
| United Arab Emirates | 0  (0, 0) | 0  (0, 0) | 0  (0, 0) | 0  (0, 0) | 0  (0, 0) | 0  (0, 0) | 0  (0, 0) | 0  (0, 0) |  |
| United Kingdom | 0  (0, 0) | 0.45  (0.39, 0.52) | 0  (0, 0) | 0  (0, 0) | 0  (0, 0) | 0  (0, 0) | 0  (0, 0) | 0  (0, 0) |  |
| United Republic of Tanzania | 6261.42  (4221.89, 9360.71) | 0  (0, 0) | 0  (0, 0) | 606.25  (383.12, 1011.4) | 496.52  (429.87, 572.77) | 0.99  (0.02, 7.85) | 0.06  (0.02, 0.15) | 0  (0, 0) |  |
| United States of America | 0  (0, 0) | 61.17  (50.87, 71.97) | 0.11  (0.07, 0.18) | 0  (0, 0) | 0  (0, 0) | 0.02  (0, 0.12) | 0  (0, 0) | 0  (0, 0) |  |
| United States Virgin Islands | 0  (0, 0) | 0  (0, 0) | 0  (0, 0) | 0  (0, 0) | 0  (0, 0) | 11.15  (0.17, 63.2) | 0  (0, 0) | 0.05  (0, 0.32) |  |
| Uruguay | 0  (0, 0) | 421.51  (350.28, 493.8) | 0  (0, 0) | 0  (0, 0) | 0  (0, 0) | 0  (0, 0) | 0  (0, 0) | 0  (0, 0) |  |
| Uzbekistan | 0  (0, 0) | 0  (0, 0) | 103.38  (76.9, 149.68) | 0  (0, 0) | 0  (0, 0) | 0  (0, 0) | 0  (0, 0) | 0  (0, 0) |  |
| Vanuatu | 496.05  (462.09, 534.41) | 0  (0, 0) | 0  (0, 0) | 2699.75  (336.15, 20612.67) | 0  (0, 0) | 47.49  (12.16, 136.76) | 0  (0, 0) | 0  (0, 0) |  |
| Venezuela  (Bolivarian Republic of) | 1070.27  (743.66, 1434.41) | 1578.82  (1371.2, 1799.9) | 233.25  (212.1, 258.51) | 0  (0, 0) | 18.72  (8.55, 32.35) | 60.96  (7.12, 155.55) | 0.01  (0, 0.03) | 0.07  (0.01, 0.35) |  |
| Viet Nam | 26.23  (0.19, 32) | 0  (0, 0) | 0  (0, 0) | 767.09  (258.93, 2977.92) | 0  (0, 0) | 66.63  (21.99, 154.52) | 0  (0, 0) | 0  (0, 0) |  |
| Yemen | 2177.53  (1728.99, 2742.81) | 0  (0, 0) | 828.26  (590.55, 1206.57) | 407.55  (208.61, 1173.86) | 2.67  (2.64, 2.7) | 1.41  (0.14, 6.99) | 0  (0, 0) | 0  (0, 0) |  |
| Zambia | 10326.96  (6329.9, 14719.81) | 0  (0, 0) | 0.23  (0.04, 0.71) | 2148.68  (727.47, 6289.39) | 0  (0, 0) | 2.5  (0.04, 16.76) | 0.04  (0.01, 0.1) | 0  (0, 0) |  |
| Zimbabwe | 1092.76  (808.79, 1440.38) | 0  (0, 0) | 0  (0, 0) | 895.91  (296.34, 2977.76) | 0  (0, 0) | 0.4  (0.02, 2.73) | 0  (0, 0) | 0  (0, 0) |  |

ASPRs age-standardized prevalence rates

**Table F** ASPR AAPCs (1990–2021) for nine arthropod-borne diseases in 204 countries and territories

| **Location** | **Malaria** | **Chagas disease** | **Leishmaniasis** | **African trypanosomiasis** | **Lymphatic filariasis** | **Onchocerciasis** | **Dengue** | **Yellow fever** | **Zika virus** |  |
| --- | --- | --- | --- | --- | --- | --- | --- | --- | --- | --- |
| Afghanistan | -3.79  (-5.05, -2.52) | #N/A | 3.19  (2.98, 3.41) | #N/A | #N/A | #N/A | 1  (0.54, 1.47) | #N/A | #N/A |  |
| Albania | #N/A | #N/A | -0.82  (-1.1, -0.54) | #N/A | #N/A | #N/A | #N/A | #N/A | #N/A |  |
| Algeria | #N/A | #N/A | 0.79  (0.52, 1.06) | #N/A | #N/A | #N/A | #N/A | #N/A | #N/A |  |
| American Samoa | #N/A | #N/A | #N/A | #N/A | -2.53  (-3.9, -1.14) | #N/A | 0.39  (0.19, 0.6) | #N/A | #N/A |  |
| Andorra | #N/A | 1.25  (0.96, 1.54) | #N/A | #N/A | #N/A | #N/A | #N/A | #N/A | #N/A |  |
| Angola | -0.86  (-1.1, -0.63) | #N/A | -11.39  (-12.34, -10.44) | -14.84  (-17.21, -12.4) | -6.1  (-6.6, -5.6) | -2.03  (-2.17, -1.89) | 6.6  (6.17, 7.02) | -7.17  (-8.14, -6.19) | #N/A |  |
| Antigua and Barbuda | #N/A | -0.52  (-1.03, -0.01) | #N/A | #N/A | #N/A | #N/A | -0.25  (-1.58, 1.09) | #N/A | #N/A |  |
| Argentina | #N/A | -3.34  (-3.65, -3.02) | 0.03  (-0.06, 0.12) | #N/A | #N/A | #N/A | 1.37  (1.19, 1.54) | -5.23  (-5.44, -5.02) | #N/A |  |
| Armenia | #N/A | #N/A | -3.82  (-3.85, -3.8) | #N/A | #N/A | #N/A | #N/A | #N/A | #N/A |  |
| Australia | #N/A | -2.77  (-2.84, -2.7) | #N/A | #N/A | #N/A | #N/A | 2.44  (2.04, 2.85) | #N/A | #N/A |  |
| Austria | #N/A | -1.39  (-1.46, -1.33) | -1.94  (-2.09, -1.78) | #N/A | #N/A | #N/A | #N/A | #N/A | #N/A |  |
| Azerbaijan | #N/A | #N/A | -2.39  (-2.44, -2.33) | #N/A | #N/A | #N/A | #N/A | #N/A | #N/A |  |
| Bahamas | #N/A | 3.37  (2.88, 3.87) | #N/A | #N/A | #N/A | #N/A | 0.51  (0.44, 0.59) | #N/A | #N/A |  |
| Bahrain | #N/A | #N/A | #N/A | #N/A | #N/A | #N/A | #N/A | #N/A | #N/A |  |
| Bangladesh | -6.88  (-8.41, -5.32) | #N/A | -14.61  (-15.45, -13.77) | #N/A | -8.42  (-8.78, -8.06) | #N/A | 1.82  (1.77, 1.88) | #N/A | #N/A |  |
| Barbados | #N/A | -5.02  (-5.21, -4.82) | #N/A | #N/A | #N/A | #N/A | 0.59  (-0.93, 2.13) | #N/A | #N/A |  |
| Belgium | #N/A | -0.23  (-0.3, -0.16) | #N/A | #N/A | #N/A | #N/A | #N/A | #N/A | #N/A |  |
| Belize | #N/A | -4.38  (-4.58, -4.18) | -1.18  (-1.21, -1.15) | #N/A | #N/A | #N/A | 1.16  (-0.44, 2.8) | #N/A | #N/A |  |
| Benin | -0.31  (-0.68, 0.06) | #N/A | #N/A | #N/A | -4.66  (-5.42, -3.9) | -12.49  (-13.05, -11.93) | 0.18  (-0.25, 0.61) | -6.11  (-6.73, -5.49) | #N/A |  |
| Bermuda | #N/A | -2.36  (-2.53, -2.18) | #N/A | #N/A | #N/A | #N/A | #N/A | #N/A | #N/A |  |
| Bhutan | -15.91  (-18.21, -13.55) | #N/A | -11.06  (-11.59, -10.53) | #N/A | #N/A | #N/A | 1.83  (0.12, 3.58) | #N/A | #N/A |  |
| BOLIVIA | -4.98  (-5.45, -4.5) | -3.42  (-3.53, -3.31) | -0.22  (-0.25, -0.18) | #N/A | #N/A | #N/A | 1.86  (1.6, 2.12) | -7.03  (-8.17, -5.87) | #N/A |  |
| Bosnia and Herzegovina | #N/A | #N/A | -1.72  (-1.77, -1.67) | #N/A | #N/A | #N/A | #N/A | #N/A | #N/A |  |
| Botswana | -0.66  (-3.87, 2.64) | #N/A | #N/A | #N/A | #N/A | #N/A | #N/A | #N/A | #N/A |  |
| Brazil | -3.69  (-4.33, -3.06) | -2.07  (-2.1, -2.04) | -0.07  (-0.12, -0.01) | #N/A | -9.67  (-10.33, -9) | -14.79  (-15.47, -14.11) | 0.93  (0.54, 1.31) | -10.3  (-20.16, 0.78) | #N/A |  |
| Brunei | #N/A | #N/A | #N/A | #N/A | -1.8  (-2.46, -1.13) | #N/A | 3.72  (2.93, 4.51) | #N/A | #N/A |  |
| Bulgaria | #N/A | #N/A | -1.7  (-1.72, -1.68) | #N/A | #N/A | #N/A | #N/A | #N/A | #N/A |  |
| Burkina Faso | -2.63  (-3.01, -2.24) | #N/A | 1.66  (1.51, 1.81) | #N/A | -10.98  (-11.86, -10.08) | -24.23  (-25.18, -23.26) | 3.23  (2.98, 3.48) | -5.29  (-6.83, -3.72) | #N/A |  |
| Burundi | -2.44  (-2.96, -1.92) | #N/A | #N/A | #N/A | #N/A | -2.4  (-2.77, -2.02) | -9.32  (-10.54, -8.08) | -4.92  (-5.35, -4.48) | #N/A |  |
| Cambodia | -6.69  (-8.05, -5.31) | #N/A | #N/A | #N/A | -9.46  (-12.94, -5.83) | #N/A | 0.95  (0.72, 1.19) | #N/A | #N/A |  |
| Cameroon | -2.65  (-2.87, -2.43) | #N/A | 0.2  (0.13, 0.28) | -4.74  (-5.9, -3.56) | -8.76  (-10.12, -7.39) | -4.8  (-4.88, -4.72) | 1.59  (1.17, 2.01) | -10.12  (-12.96, -7.18) | #N/A |  |
| Canada | #N/A | -1.15  (-1.22, -1.09) | #N/A | #N/A | #N/A | #N/A | #N/A | #N/A | #N/A |  |
| CAPE VERDE | -0.05  (-0.75, 0.67) | #N/A | #N/A | #N/A | #N/A | #N/A | 0.81  (0.68, 0.94) | #N/A | #N/A |  |
| Central African Republic | -1.47  (-1.57, -1.36) | #N/A | -1.48  (-1.57, -1.4) | -12.43  (-13.28, -11.56) | -5.61  (-6.13, -5.09) | -4  (-4.09, -3.91) | 0.34  (0.13, 0.55) | -4.74  (-5.36, -4.11) | #N/A |  |
| Chad | -1.76  (-1.93, -1.58) | #N/A | 0.41  (0.35, 0.47) | -24.57  (-26.34, -22.76) | -5.21  (-5.49, -4.93) | -3.84  (-4.13, -3.54) | 1.18  (1.05, 1.31) | -5.58  (-6.29, -4.86) | #N/A |  |
| Chile | #N/A | -3.76  (-3.77, -3.75) | #N/A | #N/A | #N/A | #N/A | #N/A | #N/A | #N/A |  |
| China | #N/A | #N/A | -2.38  (-2.42, -2.35) | #N/A | #N/A | #N/A | 5.57  (5.38, 5.76) | #N/A | #N/A |  |
| Colombia | -2.06  (-2.72, -1.4) | -2.44  (-2.52, -2.37) | -0.07  (-0.15, 0.01) | #N/A | #N/A | #N/A | 1.5  (0.48, 2.53) | -5.61  (-7.26, -3.94) | #N/A |  |
| Comoros | -7.6  (-12.8, -2.1) | #N/A | #N/A | #N/A | -3.85  (-4.08, -3.62) | #N/A | 0.15  (-0.16, 0.46) | #N/A | #N/A |  |
| Congo | -1.5  (-1.68, -1.31) | #N/A | #N/A | -10.76  (-12.08, -9.41) | -5.15  (-5.6, -4.7) | -4.98  (-5.09, -4.87) | -0.62  (-0.92, -0.33) | -6.5  (-7.62, -5.36) | #N/A |  |
| CONGO,THE DEMOCRATIC REPUBLIC OF THE | -1.99  (-2.81, -1.17) | #N/A | -1.24  (-1.35, -1.12) | -17.56  (-18.79, -16.32) | -6.29  (-6.5, -6.09) | -1.77  (-1.93, -1.62) | 0.18  (0.02, 0.35) | -5.13  (-6.5, -3.73) | #N/A |  |
| Cook Islands | #N/A | #N/A | #N/A | #N/A | #N/A | #N/A | 4.16  (3.52, 4.8) | #N/A | #N/A |  |
| Costa Rica | #N/A | -2.74  (-2.82, -2.67) | 0.68  (0.64, 0.72) | #N/A | #N/A | #N/A | -0.05  (-0.98, 0.89) | #N/A | #N/A |  |
| COTE D’IVOIRE | -2.46  (-3.6, -1.31) | #N/A | 0.8  (0.74, 0.86) | -21.18  (-21.81, -20.54) | -5.06  (-5.4, -4.72) | -26.15  (-28.24, -24) | 1.69  (0.84, 2.54) | -6.11  (-7.37, -4.84) | #N/A |  |
| Croatia | #N/A | #N/A | -0.57  (-0.62, -0.52) | #N/A | #N/A | #N/A | #N/A | #N/A | #N/A |  |
| Cuba | #N/A | -6.16  (-6.32, -6) | #N/A | #N/A | #N/A | #N/A | 1.64  (1.16, 2.11) | #N/A | #N/A |  |
| Cyprus | #N/A | #N/A | 0.01  (-0.04, 0.05) | #N/A | #N/A | #N/A | #N/A | #N/A | #N/A |  |
| Denmark | #N/A | 0.71  (0.64, 0.77) | #N/A | #N/A | #N/A | #N/A | #N/A | #N/A | #N/A |  |
| Djibouti | 5.2  (0.51, 10.1) | #N/A | -2.64  (-2.76, -2.52) | #N/A | #N/A | #N/A | -8.91  (-9.89, -7.91) | #N/A | #N/A |  |
| Dominica | #N/A | 11.23  (8.84, 13.68) | #N/A | #N/A | #N/A | #N/A | 0.48  (-3.48, 4.6) | #N/A | #N/A |  |
| Dominican Republic | -7.98  (-11.52, -4.29) | -3.78  (-3.84, -3.73) | -0.94  (-0.96, -0.91) | #N/A | -5.06  (-6.1, -4.02) | #N/A | 1.62  (0.95, 2.29) | #N/A | #N/A |  |
| EAST TIMOR | #N/A | #N/A | #N/A | #N/A | -4  (-7.58, -0.3) | #N/A | 3.49  (2.84, 4.15) | #N/A | #N/A |  |
| Ecuador | -6.17  (-7.81, -4.5) | -3.19  (-3.23, -3.15) | -0.91  (-0.92, -0.9) | #N/A | #N/A | #N/A | 1.74  (0.51, 2.99) | -6.81  (-7.9, -5.69) | #N/A |  |
| Egypt | #N/A | #N/A | -3.08  (-3.11, -3.05) | #N/A | -5.31  (-7.66, -2.9) | #N/A | 0.2  (-0.96, 1.38) | #N/A | #N/A |  |
| El Salvador | #N/A | -2.58  (-2.64, -2.53) | -3.4  (-3.44, -3.37) | #N/A | #N/A | #N/A | 1.24  (0.79, 1.68) | #N/A | #N/A |  |
| Equatorial Guinea | -1.29  (-1.67, -0.92) | #N/A | #N/A | -10.95  (-11.41, -10.49) | -7.68  (-8.37, -6.99) | -3.34  (-3.49, -3.2) | 7.98  (5.42, 10.6) | -11.93  (-12.59, -11.27) | #N/A |  |
| Eritrea | -1.22  (-2.4, -0.02) | #N/A | -2.14  (-2.26, -2.02) | #N/A | -4.12  (-4.82, -3.41) | #N/A | -9.01  (-10.3, -7.71) | -6.68  (-6.85, -6.52) | #N/A |  |
| Ethiopia | 1.93  (0.68, 3.19) | #N/A | -2.36  (-2.45, -2.26) | #N/A | -7.07  (-7.51, -6.63) | -3.67  (-4.03, -3.3) | -10.52  (-12.16, -8.85) | -7.04  (-7.23, -6.86) | #N/A |  |
| Fiji | #N/A | #N/A | #N/A | #N/A | -3.09  (-4.02, -2.15) | #N/A | 1.52  (1.42, 1.62) | #N/A | #N/A |  |
| Finland | #N/A | 4.53  (4.23, 4.84) | #N/A | #N/A | #N/A | #N/A | #N/A | #N/A | #N/A |  |
| France | #N/A | -1.25  (-1.32, -1.18) | -1.24  (-1.27, -1.21) | #N/A | #N/A | #N/A | #N/A | #N/A | #N/A |  |
| Gabon | -1.21  (-1.42, -0.99) | #N/A | #N/A | -5.56  (-5.86, -5.26) | -3.8  (-4.48, -3.12) | #N/A | 0.48  (-0.02, 0.97) | -7.92  (-8.99, -6.84) | #N/A |  |
| Gambia | -2.47  (-5.18, 0.33) | #N/A | 0.79  (0.75, 0.83) | #N/A | #N/A | #N/A | 1.86  (1.5, 2.23) | -6.58  (-6.87, -6.29) | #N/A |  |
| Georgia | #N/A | #N/A | -3.49  (-3.52, -3.46) | #N/A | #N/A | #N/A | #N/A | #N/A | #N/A |  |
| Germany | #N/A | -0.66  (-0.76, -0.56) | #N/A | #N/A | #N/A | #N/A | #N/A | #N/A | #N/A |  |
| Ghana | -4.6  (-5.22, -3.99) | #N/A | -0.67  (-0.74, -0.6) | #N/A | -7.38  (-8.07, -6.68) | -19.53  (-20.91, -18.13) | 1.75  (1.43, 2.07) | -6.79  (-7.95, -5.63) | #N/A |  |
| Greece | #N/A | -2.89  (-3.07, -2.72) | -0.56  (-0.66, -0.47) | #N/A | #N/A | #N/A | #N/A | #N/A | #N/A |  |
| Grenada | #N/A | 7.06  (6.68, 7.44) | #N/A | #N/A | #N/A | #N/A | 5.65  (4.96, 6.35) | #N/A | #N/A |  |
| Guam | #N/A | #N/A | #N/A | #N/A | #N/A | #N/A | #N/A | #N/A | #N/A |  |
| Guatemala | -7.08  (-8.94, -5.18) | -2.96  (-3.01, -2.91) | -2.38  (-2.41, -2.36) | #N/A | #N/A | #N/A | 0.37  (-0.35, 1.1) | #N/A | #N/A |  |
| Guinea | -1.47  (-1.69, -1.25) | #N/A | 0.6  (0.55, 0.65) | -1.07  (-2.17, 0.04) | -6.95  (-7.5, -6.4) | -10.27  (-10.68, -9.85) | 0.74  (-0.25, 1.73) | -6.5  (-7.84, -5.13) | #N/A |  |
| Guinea-Bissau | -5.47  (-5.92, -5.01) | #N/A | 0.76  (0.69, 0.83) | #N/A | -8.48  (-9.56, -7.39) | -28.37  (-30.28, -26.4) | 1.86  (0.55, 3.19) | -6.08  (-6.22, -5.94) | #N/A |  |
| Guyana | -1.25  (-4.06, 1.64) | -5.12  (-5.33, -4.9) | -1.04  (-1.09, -0.99) | #N/A | -3.01  (-3.26, -2.75) | #N/A | 0.82  (0.2, 1.45) | -6.11  (-6.26, -5.96) | #N/A |  |
| Haiti | 1.86  (-2.38, 6.28) | #N/A | #N/A | #N/A | -9.71  (-10.92, -8.48) | #N/A | 1.07  (0.5, 1.65) | #N/A | #N/A |  |
| Honduras | -8.45  (-11.21, -5.6) | -2.28  (-2.32, -2.23) | -0.62  (-0.63, -0.61) | #N/A | #N/A | #N/A | 1.12  (-0.16, 2.42) | #N/A | #N/A |  |
| Iceland | #N/A | 10.72  (10.13, 11.3) | #N/A | #N/A | #N/A | #N/A | #N/A | #N/A | #N/A |  |
| India | -6.52  (-8.19, -4.83) | #N/A | -8.47  (-9.15, -7.79) | #N/A | -5.42  (-5.66, -5.18) | #N/A | 1.39  (1.34, 1.44) | #N/A | #N/A |  |
| Indonesia | -2.93  (-3.35, -2.51) | #N/A | #N/A | #N/A | -6.85  (-7.34, -6.36) | #N/A | 0  (-0.07, 0.07) | #N/A | #N/A |  |
| IRAN | -13.75  (-15.3, -12.17) | #N/A | -0.8  (-0.84, -0.77) | #N/A | #N/A | #N/A | #N/A | #N/A | #N/A |  |
| Iraq | #N/A | #N/A | 0.57  (0.45, 0.69) | #N/A | #N/A | #N/A | #N/A | #N/A | #N/A |  |
| Ireland | #N/A | 14.38  (13.72, 15.05) | #N/A | #N/A | #N/A | #N/A | #N/A | #N/A | #N/A |  |
| Israel | #N/A | -3.98  (-4.16, -3.8) | 1.12  (1.06, 1.17) | #N/A | #N/A | #N/A | #N/A | #N/A | #N/A |  |
| Italy | #N/A | #N/A | -0.75  (-0.84, -0.65) | #N/A | #N/A | #N/A | #N/A | #N/A | #N/A |  |
| Jamaica | #N/A | -2.29  (-2.43, -2.16) | #N/A | #N/A | #N/A | #N/A | 0.76  (-2.36, 3.97) | #N/A | #N/A |  |
| Japan | #N/A | 4.02  (3.6, 4.44) | #N/A | #N/A | #N/A | #N/A | #N/A | #N/A | #N/A |  |
| Jordan | #N/A | #N/A | -0.81  (-0.95, -0.67) | #N/A | #N/A | #N/A | 1.36  (-1.1, 3.89) | #N/A | #N/A |  |
| Kazakhstan | #N/A | #N/A | -1.77  (-1.86, -1.68) | #N/A | #N/A | #N/A | #N/A | #N/A | #N/A |  |
| Kenya | -5.33  (-5.75, -4.91) | #N/A | -1.81  (-1.97, -1.65) | #N/A | -7.79  (-8.57, -7.01) | #N/A | -5.76  (-6.98, -4.52) | -6.79  (-7.05, -6.52) | #N/A |  |
| Kiribati | #N/A | #N/A | #N/A | #N/A | -10.01  (-10.64, -9.37) | #N/A | 0.1  (-0.05, 0.25) | #N/A | #N/A |  |
| KOREA, REPUBLIC OF | -2.37  (NA, NA) | #N/A | #N/A | #N/A | #N/A | #N/A | #N/A | #N/A | #N/A |  |
| Kuwait | #N/A | #N/A | -2.31  (-2.37, -2.26) | #N/A | #N/A | #N/A | -1.68  (-3.49, 0.16) | #N/A | #N/A |  |
| Kyrgyzstan | #N/A | #N/A | -3.66  (-3.68, -3.65) | #N/A | #N/A | #N/A | #N/A | #N/A | #N/A |  |
| Laos | -4.87  (-7.61, -2.05) | #N/A | #N/A | #N/A | -6.39  (-7.57, -5.19) | #N/A | 1.99  (0.93, 3.07) | #N/A | #N/A |  |
| Lebanon | #N/A | #N/A | -2.7  (-2.72, -2.68) | #N/A | #N/A | #N/A | 0.83  (-2.3, 4.07) | #N/A | #N/A |  |
| Liberia | -1.03  (-1.43, -0.64) | #N/A | #N/A | #N/A | -2.49  (-3.76, -1.21) | -1.81  (-1.88, -1.73) | 0.93  (0.29, 1.57) | -7.63  (-9.05, -6.19) | #N/A |  |
| Libya | #N/A | #N/A | 0.09  (0, 0.17) | #N/A | #N/A | #N/A | #N/A | #N/A | #N/A |  |
| Luxembourg | #N/A | -0.65  (-0.84, -0.47) | #N/A | #N/A | #N/A | #N/A | #N/A | #N/A | #N/A |  |
| Madagascar | -3.78  (-4.07, -3.48) | #N/A | #N/A | #N/A | -8.13  (-9.39, -6.85) | #N/A | -6.6  (-7.84, -5.34) | #N/A | #N/A |  |
| Malawi | -3.34  (-3.7, -2.98) | #N/A | -1.02  (-1.06, -0.97) | -4.82  (-5.24, -4.39) | -11.89  (-12.59, -11.2) | -4.44  (-4.58, -4.3) | -9.03  (-10, -8.06) | #N/A | #N/A |  |
| Malaysia | -25.05  (-28.61, -21.31) | #N/A | #N/A | #N/A | -4.56  (-5.28, -3.83) | #N/A | 3.65  (2.8, 4.51) | #N/A | #N/A |  |
| Maldives | #N/A | #N/A | #N/A | #N/A | -6.48  (-9.7, -3.15) | #N/A | 9.32  (8.64, 9.99) | #N/A | #N/A |  |
| Mali | -2.37  (-2.71, -2.02) | #N/A | 0.08  (0.03, 0.12) | #N/A | -9.77  (-11.4, -8.11) | -20  (-20.77, -19.21) | 0.9  (0.78, 1.01) | -5.96  (-6.15, -5.77) | #N/A |  |
| Malta | #N/A | #N/A | 0.44  (0.14, 0.74) | #N/A | #N/A | #N/A | #N/A | #N/A | #N/A |  |
| Marshall Islands | #N/A | #N/A | #N/A | #N/A | -6.53  (-6.91, -6.14) | #N/A | 0.64  (0.47, 0.8) | #N/A | #N/A |  |
| Mauritania | -0.34  (-3.31, 2.71) | #N/A | 0.44  (0.39, 0.48) | #N/A | #N/A | #N/A | 1.79  (1.22, 2.37) | -6.57  (-6.74, -6.4) | #N/A |  |
| Mauritius | #N/A | #N/A | #N/A | #N/A | #N/A | #N/A | 4.18  (3.83, 4.53) | #N/A | #N/A |  |
| Mexico | -9.08  (-10.47, -7.66) | -2.02  (-2.3, -1.73) | -1.68  (-1.71, -1.65) | #N/A | #N/A | #N/A | 1.94  (0.88, 3.02) | #N/A | #N/A |  |
| Micronesia  (Federated States of) | #N/A | #N/A | #N/A | #N/A | -6.04  (-7.3, -4.75) | #N/A | -0.11  (-0.46, 0.24) | #N/A | #N/A |  |
| Monaco | #N/A | #N/A | 3.25  (2.98, 3.52) | #N/A | #N/A | #N/A | #N/A | #N/A | #N/A |  |
| Montenegro | #N/A | #N/A | 0.9  (0.41, 1.4) | #N/A | #N/A | #N/A | #N/A | #N/A | #N/A |  |
| Morocco | #N/A | #N/A | 4.27  (3.95, 4.58) | #N/A | #N/A | #N/A | #N/A | #N/A | #N/A |  |
| Mozambique | -1.74  (-1.93, -1.54) | #N/A | #N/A | #N/A | -11.77  (-12.68, -10.84) | #N/A | -4.97  (-6.32, -3.6) | #N/A | #N/A |  |
| Myanmar | -2  (-3.73, -0.24) | #N/A | #N/A | #N/A | -7.06  (-7.86, -6.25) | #N/A | 0.2  (-0.48, 0.88) | #N/A | #N/A |  |
| Namibia | -6.88  (-12.14, -1.32) | #N/A | -0.9  (-0.92, -0.87) | #N/A | #N/A | #N/A | #N/A | #N/A | #N/A |  |
| Nauru | #N/A | #N/A | #N/A | #N/A | #N/A | #N/A | 12.13  (6.78, 17.74) | #N/A | #N/A |  |
| Nepal | -7.92  (-9.34, -6.49) | #N/A | -2.78  (-2.81, -2.74) | #N/A | -5.55  (-5.76, -5.34) | #N/A | 1.91  (1.69, 2.12) | #N/A | #N/A |  |
| Netherlands | #N/A | 0.83  (0.79, 0.88) | #N/A | #N/A | #N/A | #N/A | #N/A | #N/A | #N/A |  |
| Nicaragua | -2.96  (-3.31, -2.6) | -2.77  (-2.82, -2.72) | 1.37  (1.2, 1.55) | #N/A | #N/A | #N/A | 2.53  (2.18, 2.88) | #N/A | #N/A |  |
| Niger | -1.03  (-1.24, -0.81) | #N/A | 4.05  (3.73, 4.36) | #N/A | -8.33  (-8.59, -8.05) | -17.92  (-19.15, -16.68) | -0.1  (-0.35, 0.14) | -4.83  (-4.94, -4.72) | #N/A |  |
| Nigeria | -1.81  (-1.95, -1.66) | #N/A | -0.4  (-0.47, -0.32) | #N/A | -8.32  (-9.88, -6.75) | -4.53  (-4.6, -4.47) | 1.03  (0.81, 1.26) | -8.33  (-8.74, -7.91) | #N/A |  |
| Niue | #N/A | #N/A | #N/A | #N/A | -4.04  (-4.37, -3.71) | #N/A | 1.24  (0.77, 1.71) | #N/A | #N/A |  |
| North Macedonia | #N/A | #N/A | -1.29  (-1.31, -1.28) | #N/A | #N/A | #N/A | #N/A | #N/A | #N/A |  |
| Northern Mariana Islands | #N/A | #N/A | #N/A | #N/A | #N/A | #N/A | 0.86  (-0.5, 2.23) | #N/A | #N/A |  |
| Oman | #N/A | #N/A | -0.32  (-0.4, -0.24) | #N/A | #N/A | #N/A | 2.05  (1.11, 3) | #N/A | #N/A |  |
| Pakistan | -2.92  (-3.28, -2.56) | #N/A | -0.41  (-0.44, -0.38) | #N/A | #N/A | #N/A | 0.51  (0.47, 0.55) | #N/A | #N/A |  |
| Palau | #N/A | #N/A | #N/A | #N/A | -0.45  (-0.46, -0.44) | #N/A | 0.17  (-0.32, 0.66) | #N/A | #N/A |  |
| Palestine | #N/A | #N/A | -1.66  (-1.71, -1.62) | #N/A | #N/A | #N/A | -0.13  (-4.09, 4) | #N/A | #N/A |  |
| Panama | 1.53  (0.31, 2.77) | -2.64  (-2.74, -2.54) | 0.31  (0.23, 0.39) | #N/A | #N/A | #N/A | 2.92  (2.38, 3.47) | -5.62  (-5.83, -5.41) | #N/A |  |
| Papua New Guinea | -3.01  (-4.36, -1.64) | #N/A | #N/A | #N/A | -5  (-5.32, -4.67) | #N/A | 2.38  (1.63, 3.14) | #N/A | #N/A |  |
| Paraguay | #N/A | -3.01  (-3.07, -2.95) | -0.12  (-0.19, -0.05) | #N/A | #N/A | #N/A | 2.5  (1.08, 3.93) | -6.05  (-6.51, -5.59) | #N/A |  |
| Peru | -6.07  (-6.62, -5.51) | -2.98  (-3.01, -2.95) | 0.33  (0.29, 0.37) | #N/A | #N/A | #N/A | 2.33  (1.91, 2.74) | -7.05  (-8.34, -5.75) | #N/A |  |
| Philippines | -11.66  (-12.51, -10.81) | #N/A | #N/A | #N/A | -7.82  (-8.72, -6.91) | #N/A | 6.33  (6.05, 6.61) | #N/A | #N/A |  |
| Portugal | #N/A | 2.36  (2.27, 2.46) | -2.06  (-2.1, -2.03) | #N/A | #N/A | #N/A | #N/A | #N/A | #N/A |  |
| Puerto Rico | #N/A | 2.59  (2, 3.18) | #N/A | #N/A | #N/A | #N/A | -0.37  (-0.71, -0.04) | #N/A | #N/A |  |
| Qatar | #N/A | #N/A | #N/A | #N/A | #N/A | #N/A | #N/A | #N/A | #N/A |  |
| Romania | #N/A | #N/A | -15.22  (-15.77, -14.67) | #N/A | #N/A | #N/A | #N/A | #N/A | #N/A |  |
| Rwanda | -6.84  (-10.95, -2.54) | #N/A | #N/A | #N/A | #N/A | #N/A | -10.92  (-11.76, -10.07) | -6.16  (-6.36, -5.96) | #N/A |  |
| Saint Kitts and Nevis | #N/A | #N/A | #N/A | #N/A | #N/A | #N/A | -0.94  (-1.25, -0.63) | #N/A | #N/A |  |
| Saint Lucia | #N/A | 0.08  (-0.19, 0.36) | #N/A | #N/A | #N/A | #N/A | 2.46  (1.59, 3.34) | #N/A | #N/A |  |
| Saint Vincent and the Grenadines | #N/A | -3.22  (-3.74, -2.69) | #N/A | #N/A | #N/A | #N/A | 0.39  (-0.57, 1.36) | #N/A | #N/A |  |
| Samoa | #N/A | #N/A | #N/A | #N/A | -2.32  (-3.2, -1.42) | #N/A | 3.8  (3.34, 4.26) | #N/A | #N/A |  |
| Sao Tome and Principe | -4.66  (-9.31, 0.23) | #N/A | #N/A | #N/A | -2.89  (-3.2, -2.58) | #N/A | -0.15  (-0.48, 0.17) | -6.86  (-6.96, -6.76) | #N/A |  |
| Saudi Arabia | -10.34  (-11.55, -9.12) | #N/A | -1.83  (-1.88, -1.79) | #N/A | #N/A | #N/A | 4.56  (4.26, 4.85) | #N/A | #N/A |  |
| Senegal | -1.16  (-2.34, 0.04) | #N/A | -0.42  (-0.47, -0.37) | #N/A | -7.65  (-7.94, -7.36) | -13.19  (-14.29, -12.07) | 1.9  (1.54, 2.27) | -7.35  (-8.74, -5.93) | #N/A |  |
| Serbia | #N/A | #N/A | -11.39  (-11.99, -10.78) | #N/A | #N/A | #N/A | #N/A | #N/A | #N/A |  |
| Seychelles | #N/A | #N/A | #N/A | #N/A | #N/A | #N/A | 1.83  (1.63, 2.03) | #N/A | #N/A |  |
| Sierra Leone | -1.36  (-1.54, -1.18) | #N/A | #N/A | #N/A | -6.24  (-6.49, -6) | -3.31  (-3.53, -3.09) | 0.04  (-0.24, 0.31) | -5.48  (-6.78, -4.15) | #N/A |  |
| Singapore | #N/A | #N/A | #N/A | #N/A | #N/A | #N/A | 0.49  (-0.27, 1.26) | #N/A | #N/A |  |
| Slovenia | #N/A | #N/A | -2.03  (-2.05, -2.02) | #N/A | #N/A | #N/A | #N/A | #N/A | #N/A |  |
| Solomon Islands | -3.23  (-4.27, -2.19) | #N/A | #N/A | #N/A | #N/A | #N/A | 3.12  (2.71, 3.52) | #N/A | #N/A |  |
| Somalia | -3.59  (-4.28, -2.9) | #N/A | -4.74  (-4.84, -4.64) | #N/A | #N/A | #N/A | -8.66  (-9.44, -7.88) | -3.46  (-3.5, -3.42) | #N/A |  |
| South Africa | -9.2  (-13.84, -4.3) | #N/A | 0.2  (0.14, 0.26) | #N/A | #N/A | #N/A | #N/A | #N/A | #N/A |  |
| South Sudan | -1.65  (-1.97, -1.32) | #N/A | -2  (-2.17, -1.83) | -4.95  (-7.67, -2.15) | -4.29  (-4.92, -3.65) | -0.31  (-0.38, -0.25) | -19.27  (-21.58, -16.88) | -5.16  (-5.58, -4.74) | #N/A |  |
| Spain | #N/A | 4.58  (4.24, 4.92) | -2.38  (-2.43, -2.34) | #N/A | #N/A | #N/A | #N/A | #N/A | #N/A |  |
| Sri Lanka | #N/A | #N/A | 3.73  (3.65, 3.81) | #N/A | -6.58  (-7.27, -5.87) | #N/A | 5.08  (4.84, 5.33) | #N/A | #N/A |  |
| Sudan | -0.92  (-1.35, -0.48) | #N/A | -1.99  (-2.04, -1.94) | #N/A | -5.12  (-5.29, -4.95) | -3.73  (-3.82, -3.63) | 1.43  (1.03, 1.83) | -8.63  (-8.85, -8.41) | #N/A |  |
| Suriname | -6.1  (-9.88, -2.17) | -4.67  (-4.81, -4.53) | 0.22  (0.17, 0.26) | #N/A | #N/A | #N/A | -0.33  (-1.05, 0.39) | -5.25  (-5.88, -4.63) | #N/A |  |
| SWAZILAND | -7.4  (-10.38, -4.31) | #N/A | #N/A | #N/A | #N/A | #N/A | #N/A | #N/A | #N/A |  |
| Sweden | #N/A | -1.8  (-1.85, -1.76) | #N/A | #N/A | #N/A | #N/A | #N/A | #N/A | #N/A |  |
| Switzerland | #N/A | 0  (-0.11, 0.1) | #N/A | #N/A | #N/A | #N/A | #N/A | #N/A | #N/A |  |
| Syrian Arab Republic | #N/A | #N/A | 2.22  (2.08, 2.36) | #N/A | #N/A | #N/A | -0.09  (-4.13, 4.11) | #N/A | #N/A |  |
| Tajikistan | #N/A | #N/A | -3.69  (-3.72, -3.66) | #N/A | #N/A | #N/A | #N/A | #N/A | #N/A |  |
| Tanzania | -4.44  (-4.93, -3.94) | #N/A | #N/A | #N/A | -10.67  (-11.48, -9.85) | -4.36  (-4.42, -4.29) | -12.22  (-14.79, -9.58) | -6.1  (-6.23, -5.96) | #N/A |  |
| Thailand | -8.54  (-9.05, -8.02) | #N/A | -1.4  (-1.46, -1.33) | #N/A | -8.06  (-8.61, -7.51) | #N/A | 1.79  (0.93, 2.65) | #N/A | #N/A |  |
| Togo | -3.23  (-4.14, -2.31) | #N/A | #N/A | #N/A | -9.76  (-12.02, -7.44) | -15.54  (-16.58, -14.49) | 0.8  (0.72, 0.88) | -5.61  (-6.12, -5.09) | #N/A |  |
| Tokelau | #N/A | #N/A | #N/A | #N/A | #N/A | #N/A | 4.41  (3.55, 5.28) | #N/A | #N/A |  |
| Tonga | #N/A | #N/A | #N/A | #N/A | -5.15  (-7.26, -2.99) | #N/A | 9.2  (6.59, 11.86) | #N/A | #N/A |  |
| Trinidad and Tobago | #N/A | -3.37  (-3.44, -3.29) | #N/A | #N/A | #N/A | #N/A | 0.2  (-0.14, 0.54) | -5.52  (-5.69, -5.34) | #N/A |  |
| Tunisia | #N/A | #N/A | 1.68  (1.43, 1.94) | #N/A | #N/A | #N/A | #N/A | #N/A | #N/A |  |
| Turkey | #N/A | #N/A | -1.33  (-1.44, -1.23) | #N/A | #N/A | #N/A | #N/A | #N/A | #N/A |  |
| Turkmenistan | #N/A | #N/A | -2.52  (-2.6, -2.44) | #N/A | #N/A | #N/A | #N/A | #N/A | #N/A |  |
| Tuvalu | #N/A | #N/A | #N/A | #N/A | #N/A | #N/A | 7.13  (6.28, 7.99) | #N/A | #N/A |  |
| Uganda | -2.29  (-2.66, -1.92) | #N/A | -1.45  (-1.51, -1.39) | -29.39  (-30.47, -28.29) | -10.44  (-11.21, -9.66) | -5.21  (-5.29, -5.13) | -9.5  (-10.75, -8.23) | -7.92  (-9.04, -6.78) | #N/A |  |
| United Kingdom | #N/A | 3.99  (3.86, 4.12) | #N/A | #N/A | #N/A | #N/A | #N/A | #N/A | #N/A |  |
| United States | #N/A | -0.01  (-0.18, 0.17) | 0.35  (0.32, 0.38) | #N/A | #N/A | #N/A | 4.02  (3.24, 4.81) | #N/A | #N/A |  |
| United States Virgin Islands | #N/A | #N/A | #N/A | #N/A | #N/A | #N/A | -0.38  (-0.86, 0.1) | #N/A | #N/A |  |
| Uruguay | #N/A | -3.31  (-3.35, -3.27) | #N/A | #N/A | #N/A | #N/A | #N/A | #N/A | #N/A |  |
| Uzbekistan | #N/A | #N/A | -1.05  (-1.11, -0.99) | #N/A | #N/A | #N/A | #N/A | #N/A | #N/A |  |
| Vanuatu | -9.83  (-10.71, -8.95) | #N/A | #N/A | #N/A | -4.41  (-6.42, -2.36) | #N/A | 4.37  (3.89, 4.85) | #N/A | #N/A |  |
| VENEZUELA | 2.12  (1.36, 2.89) | -1.49  (-1.54, -1.45) | -0.24  (-0.27, -0.21) | #N/A | #N/A | -10.97  (-11.75, -10.17) | 0.22  (-0.6, 1.05) | -5.01  (-5.85, -4.17) | #N/A |  |
| Viet Nam | -6.09  (-7.07, -5.1) | #N/A | #N/A | #N/A | -8.29  (-8.84, -7.74) | #N/A | 2.07  (1.66, 2.49) | #N/A | #N/A |  |
| Yemen | -2.07  (-2.71, -1.42) | #N/A | -1.19  (-1.21, -1.17) | #N/A | -2.55  (-3.34, -1.75) | -2.52  (-2.55, -2.48) | 1.77  (1.62, 1.92) | #N/A | #N/A |  |
| Zambia | -3.48  (-3.8, -3.16) | #N/A | -13.77  (-14.59, -12.94) | -0.4  (-1.4, 0.61) | -7.08  (-7.31, -6.85) | #N/A | -8.66  (-10.05, -7.24) | -6.88  (-7.11, -6.66) | #N/A |  |
| Zimbabwe | -1.54  (-5.24, 2.31) | #N/A | #N/A | #N/A | -2.1  (-2.6, -1.6) | #N/A | -0.34  (-1.55, 0.89) | #N/A | #N/A |  |

ASPRs age-standardized prevalence rates

**Table G** ASDRs in 2021 for arthropod-borne diseases, protozoiasis, helminthiases, viral diseases and other than malaria in 204 countries and territories

| **Location** | **ABDs** | **Protozoiasis** | **Helminthiases** | **Viral diseases** | **Other than malaria** |
| --- | --- | --- | --- | --- | --- |
| Afghanistan | 253.93 (168.35, 339.5) | 253.88 (168.31, 339.46) | 0 (0, 0) | 0.05 (0, 0.12) | 225.06 (140.54, 309.58) |
| Albania | 7.64 (0, 41.4) | 7.64 (0, 41.4) | 0 (0, 0) | 0 (0, 0) | 7.64 (0, 41.4) |
| Algeria | 28.02 (15.72, 40.31) | 28.02 (15.72, 40.31) | 0 (0, 0) | 0 (0, 0) | 28.02 (15.72, 40.31) |
| American Samoa | 96.77 (41.21, 152.33) | 0 (0, 0) | 73.12 (33.48, 112.77) | 23.64 (0, 62.57) | 96.77 (41.21, 152.33) |
| Andorra | 0.15 (0.06, 0.23) | 0.15 (0.06, 0.23) | 0 (0, 0) | 0 (0, 0) | 0.15 (0.06, 0.23) |
| Angola | 3566.72 (694.82, 6438.63) | 3445.57 (574.89, 6316.26) | 66.91 (41.58, 92.24) | 54.24 (0, 134.19) | 149.6 (64.69, 234.5) |
| Antigua and Barbuda | 0.74 (0, 1.56) | 0.01 (0, 0.02) | 0 (0, 0) | 0.73 (0, 1.55) | 0.74 (0, 1.56) |
| Argentina | 36.25 (24.51, 47.99) | 30 (20.99, 39.01) | 0 (0, 0) | 6.25 (0, 13.78) | 36.25 (24.51, 47.99) |
| Armenia | 7.53 (0, 29.64) | 7.53 (0, 29.64) | 0 (0, 0) | 0 (0, 0) | 7.53 (0, 29.64) |
| Australia | 0.79 (0, 1.66) | 0.05 (0.03, 0.07) | 0 (0, 0) | 0.74 (0, 1.61) | 0.79 (0, 1.66) |
| Austria | 1.36 (0, 7.37) | 1.36 (0, 7.37) | 0 (0, 0) | 0 (0, 0) | 1.36 (0, 7.37) |
| Azerbaijan | 5.61 (0, 23.89) | 5.61 (0, 23.89) | 0 (0, 0) | 0 (0, 0) | 5.61 (0, 23.89) |
| Bahamas | 13.33 (0, 37.44) | 0.02 (0.01, 0.03) | 0 (0, 0) | 13.31 (0, 37.42) | 13.33 (0, 37.44) |
| Bahrain | 0.12 (0.03, 0.21) | 0 (0, 0) | 0 (0, 0) | 0.12 (0.03, 0.21) | 0.12 (0.03, 0.21) |
| Bangladesh | 51.33 (28.06, 74.59) | 23.18 (4.52, 41.85) | 23.32 (10.33, 36.32) | 4.82 (0, 9.69) | 30.5 (15.16, 45.84) |
| Barbados | 23.02 (0, 53.04) | 0 (0, 0) | 0 (0, 0) | 23.02 (0, 53.04) | 23.02 (0, 53.04) |
| Belarus | 0 (0, 0) | 0 (0, 0) | 0 (0, 0) | 0 (0, 0) | 0 (0, 0) |
| Belgium | 0.01 (0, 0.01) | 0.01 (0, 0.01) | 0 (0, 0) | 0 (0, 0) | 0.01 (0, 0.01) |
| Belize | 12.45 (4.2, 20.7) | 7.57 (3.61, 11.52) | 0 (0, 0) | 4.88 (0, 12.13) | 12.45 (4.2, 20.7) |
| Benin | 6965.74 (2390.61, 11540.88) | 6891.46 (2316.49, 11466.44) | 48.74 (27.67, 69.8) | 25.54 (0, 57.58) | 74.28 (35.94, 112.62) |
| Bermuda | 0 (0, 0) | 0 (0, 0) | 0 (0, 0) | 0 (0, 0) | 0 (0, 0) |
| Bhutan | 22.72 (0, 48.17) | 11.45 (0, 34.66) | 0 (0, 0) | 11.27 (0.82, 21.72) | 19.76 (0, 45.19) |
| Bolivia (Plurinational State of) | 204.29 (142.37, 266.2) | 195.34 (134.26, 256.43) | 0 (0, 0) | 8.94 (0, 19.04) | 202.56 (140.65, 264.47) |
| Bosnia and Herzegovina | 0.69 (0, 3) | 0.69 (0, 3) | 0 (0, 0) | 0 (0, 0) | 0.69 (0, 3) |
| Botswana | 27.85 (12.53, 43.16) | 27.85 (12.53, 43.16) | 0 (0, 0) | 0 (0, 0) | 0 (0, 0) |
| Brazil | 185.7 (96.98, 274.42) | 120.06 (53.14, 186.99) | 0.71 (0.45, 0.98) | 64.92 (6.68, 123.17) | 179.47 (91.06, 267.89) |
| Brunei Darussalam | 34.31 (15.42, 53.21) | 0 (0, 0) | 29.7 (11.36, 48.04) | 4.61 (0.06, 9.17) | 34.31 (15.42, 53.21) |
| Bulgaria | 0.59 (0, 2.28) | 0.59 (0, 2.28) | 0 (0, 0) | 0 (0, 0) | 0.59 (0, 2.28) |
| Burkina Faso | 9017.7 (3225.49, 14809.91) | 8958.65 (3166.64, 14750.66) | 26.64 (11.02, 42.27) | 32.4 (0, 78.24) | 79.52 (30.32, 128.71) |
| Burundi | 6251.39 (1908.81, 10593.96) | 5978.63 (1638.79, 10318.48) | 179.17 (99.03, 259.3) | 93.59 (0, 225.19) | 272.75 (118.67, 426.83) |
| Cabo Verde | 97.75 (0, 308.9) | 24.02 (16.08, 31.96) | 0 (0, 0) | 73.73 (0, 284.73) | 73.73 (0, 284.73) |
| Cambodia | 26.21 (9.98, 42.44) | 5.74 (3.85, 7.63) | 0 (0, 0) | 20.47 (4.35, 36.58) | 20.47 (4.35, 36.58) |
| Cameroon | 6059.95 (1550.06, 10569.83) | 5680.99 (1173.57, 10188.41) | 371.7 (223.37, 520.02) | 7.26 (0, 21.07) | 391.88 (242.81, 540.94) |
| Canada | 0.09 (0.05, 0.13) | 0.09 (0.05, 0.13) | 0 (0, 0) | 0 (0, 0) | 0.09 (0.05, 0.13) |
| Central African Republic | 6391.42 (1104.96, 11677.88) | 5779.33 (497.46, 11061.2) | 597.09 (377.71, 816.48) | 15 (0, 34.91) | 727.76 (500.71, 954.81) |
| Chad | 3057.26 (672.47, 5442.05) | 2851.79 (468.59, 5234.99) | 169.73 (99.37, 240.09) | 35.75 (0, 87.03) | 226.09 (138.37, 313.8) |
| Chile | 15.71 (10.72, 20.7) | 15.71 (10.72, 20.7) | 0 (0, 0) | 0 (0, 0) | 15.71 (10.72, 20.7) |
| China | 4.38 (0.44, 8.32) | 1.85 (0, 4.24) | 0 (0, 0) | 2.52 (0, 5.66) | 4.38 (0.44, 8.32) |
| Colombia | 70.75 (48.6, 92.91) | 46.56 (32.48, 60.64) | 0 (0, 0) | 24.19 (7.08, 41.3) | 59.54 (39.12, 79.96) |
| Comoros | 417.35 (0, 844.63) | 234.27 (102.3, 366.23) | 65.51 (29.82, 101.19) | 117.57 (0, 522.4) | 183.08 (0, 589.47) |
| Congo | 3190.56 (978.09, 5403.02) | 3075.23 (863.63, 5286.83) | 74.29 (43.14, 105.43) | 41.04 (0, 94.48) | 198.9 (116.08, 281.71) |
| Cook Islands | 1.25 (0, 3.46) | 0 (0, 0) | 0 (0, 0) | 1.25 (0, 3.46) | 1.25 (0, 3.46) |
| Costa Rica | 58.7 (21.25, 96.15) | 32.9 (20.8, 45.01) | 0 (0, 0) | 25.8 (0, 61.23) | 58.44 (21, 95.89) |
| Côte d'Ivoire | 6293.21 (1924.52, 10661.9) | 6122.66 (1754.49, 10490.83) | 160.3 (94.31, 226.3) | 10.25 (0, 24.76) | 172.5 (104.92, 240.08) |
| Croatia | 0.6 (0, 2.98) | 0.6 (0, 2.98) | 0 (0, 0) | 0 (0, 0) | 0.6 (0, 2.98) |
| Cuba | 1.79 (0, 4.32) | 0 (0, 0) | 0 (0, 0) | 1.79 (0, 4.32) | 1.79 (0, 4.32) |
| Cyprus | 1.34 (0, 6.86) | 1.34 (0, 6.86) | 0 (0, 0) | 0 (0, 0) | 1.34 (0, 6.86) |
| Czechia | 0 (0, 0) | 0 (0, 0) | 0 (0, 0) | 0 (0, 0) | 0 (0, 0) |
| Democratic People's Republic of Korea | 0.88 (0.58, 1.18) | 0.88 (0.58, 1.18) | 0 (0, 0) | 0 (0, 0) | 0 (0, 0) |
| Democratic Republic of the Congo | 5331.87 (2514.65, 8149.1) | 4384.38 (1591.68, 7177.07) | 934.86 (564.24, 1305.47) | 12.64 (0, 29.05) | 983.19 (611.84, 1354.54) |
| Denmark | 0.03 (0.02, 0.04) | 0.03 (0.02, 0.04) | 0 (0, 0) | 0 (0, 0) | 0.03 (0.02, 0.04) |
| Djibouti | 934.41 (542.69, 1326.13) | 928.31 (537.29, 1319.33) | 0 (0, 0) | 6.1 (0, 29.51) | 88.79 (34.58, 143.01) |
| Dominica | 2.21 (0, 5.68) | 0.01 (0, 0.02) | 0 (0, 0) | 2.21 (0, 5.67) | 2.21 (0, 5.68) |
| Dominican Republic | 51.34 (28.62, 74.06) | 12.46 (3.01, 21.91) | 32.32 (13.08, 51.57) | 6.56 (0, 14.08) | 46.96 (25.92, 68) |
| Ecuador | 39.51 (27.27, 51.76) | 31.08 (20.8, 41.36) | 0 (0, 0) | 8.44 (1.78, 15.1) | 38.8 (26.56, 51.04) |
| Egypt | 20.52 (6.19, 34.84) | 20.23 (5.93, 34.53) | 0 (0, 0) | 0.28 (0, 1.11) | 20.52 (6.19, 34.84) |
| El Salvador | 46.57 (23.66, 69.48) | 27.13 (14.1, 40.17) | 0 (0, 0) | 19.44 (0.59, 38.28) | 46.57 (23.66, 69.48) |
| Equatorial Guinea | 4665.43 (1071.31, 8259.55) | 4422.11 (828.87, 8015.35) | 228.2 (151.57, 304.83) | 15.12 (0, 35.97) | 261.1 (180.63, 341.57) |
| Eritrea | 232.25 (153.36, 311.14) | 197.16 (121.25, 273.06) | 24.84 (9.95, 39.73) | 10.25 (0, 25.75) | 74.97 (44.2, 105.74) |
| Estonia | 0 (0, 0) | 0 (0, 0) | 0 (0, 0) | 0 (0, 0) | 0 (0, 0) |
| Eswatini | 25.16 (0, 50.81) | 25.16 (0, 50.81) | 0 (0, 0) | 0 (0, 0) | 0 (0, 0) |
| Ethiopia | 483.76 (129.68, 837.84) | 378.39 (27.19, 729.6) | 70.48 (43.89, 97.06) | 34.89 (0, 71.25) | 131.82 (85.56, 178.08) |
| Fiji | 225.53 (145.59, 305.47) | 0 (0, 0) | 201.55 (124.11, 278.99) | 23.98 (4.15, 43.81) | 225.53 (145.59, 305.47) |
| Finland | 0.01 (0.01, 0.02) | 0.01 (0.01, 0.02) | 0 (0, 0) | 0 (0, 0) | 0.01 (0.01, 0.02) |
| France | 0.28 (0, 1.21) | 0.28 (0, 1.21) | 0 (0, 0) | 0 (0, 0) | 0.28 (0, 1.21) |
| Gabon | 2911.08 (361.29, 5460.88) | 2845.41 (296.02, 5394.81) | 32.19 (13.16, 51.22) | 33.48 (0, 74.49) | 270.57 (84.34, 456.81) |
| Gambia | 1509.06 (828.59, 2189.52) | 1482.71 (803.32, 2162.1) | 0 (0, 0) | 26.34 (0, 64.52) | 33.7 (0, 72.05) |
| Georgia | 18.97 (0, 95.13) | 18.97 (0, 95.13) | 0 (0, 0) | 0 (0, 0) | 18.97 (0, 95.13) |
| Germany | 0.01 (0.01, 0.02) | 0.01 (0.01, 0.02) | 0 (0, 0) | 0 (0, 0) | 0.01 (0.01, 0.02) |
| Ghana | 2933.54 (997.13, 4869.96) | 2891.51 (955.34, 4827.68) | 28.18 (12.43, 43.94) | 13.85 (0, 40.27) | 51.1 (20.01, 82.2) |
| Greece | 4.33 (0, 22.77) | 4.33 (0, 22.77) | 0 (0, 0) | 0 (0, 0) | 4.33 (0, 22.77) |
| Greenland | 0 (0, 0) | 0 (0, 0) | 0 (0, 0) | 0 (0, 0) | 0 (0, 0) |
| Grenada | 3.47 (0, 7.27) | 0.16 (0.05, 0.27) | 0 (0, 0) | 3.31 (0, 7.11) | 3.47 (0, 7.27) |
| Guam | 0.09 (0.02, 0.16) | 0 (0, 0) | 0 (0, 0) | 0.09 (0.02, 0.16) | 0.09 (0.02, 0.16) |
| Guatemala | 63.53 (39.37, 87.7) | 34.33 (17.06, 51.61) | 0 (0, 0) | 29.2 (12.3, 46.1) | 63.53 (39.36, 87.69) |
| Guinea | 5656.24 (1637.04, 9675.45) | 5577.92 (1559.01, 9596.83) | 42.71 (22.56, 62.85) | 35.61 (0, 79.89) | 214.04 (84.75, 343.33) |
| Guinea-Bissau | 1830.54 (101.01, 3560.07) | 1769.76 (40.85, 3498.68) | 30.82 (12.52, 49.13) | 29.95 (0, 72.2) | 61.99 (15.94, 108.03) |
| Guyana | 785.06 (493.46, 1076.67) | 452.94 (183.71, 722.18) | 322.77 (211.08, 434.45) | 9.36 (0.93, 17.79) | 351.84 (239.42, 464.25) |
| Haiti | 350.01 (0, 843.53) | 316.08 (0, 809.15) | 29.57 (11.52, 47.61) | 4.36 (0, 15.07) | 33.93 (12.95, 54.91) |
| Honduras | 81.45 (47.78, 115.13) | 51.98 (31.54, 72.43) | 0 (0, 0) | 29.47 (2.71, 56.23) | 80.85 (47.18, 114.52) |
| Hungary | 0 (0, 0) | 0 (0, 0) | 0 (0, 0) | 0 (0, 0) | 0 (0, 0) |
| Iceland | 0.01 (0.01, 0.02) | 0.01 (0.01, 0.02) | 0 (0, 0) | 0 (0, 0) | 0.01 (0.01, 0.02) |
| India | 206.99 (35.48, 378.5) | 96.82 (0, 260.5) | 47.4 (21.6, 73.2) | 62.77 (18.52, 107.02) | 115.99 (62.52, 169.45) |
| Indonesia | 344.01 (213.28, 474.75) | 28.46 (0, 83.4) | 35.76 (14.72, 56.81) | 279.79 (163.04, 396.54) | 315.56 (196.92, 434.19) |
| Iran (Islamic Republic of) | 39.09 (21.41, 56.77) | 39.09 (21.41, 56.77) | 0 (0, 0) | 0 (0, 0) | 38.77 (21.09, 56.45) |
| Iraq | 55.03 (29.69, 80.38) | 55.03 (29.69, 80.38) | 0 (0, 0) | 0 (0, 0) | 55.03 (29.69, 80.38) |
| Ireland | 0.23 (0.13, 0.33) | 0.23 (0.13, 0.33) | 0 (0, 0) | 0 (0, 0) | 0.23 (0.13, 0.33) |
| Israel | 0.82 (0.19, 1.46) | 0.82 (0.19, 1.46) | 0 (0, 0) | 0 (0, 0) | 0.82 (0.19, 1.46) |
| Italy | 1.42 (1.07, 1.77) | 1.42 (1.07, 1.77) | 0 (0, 0) | 0 (0, 0) | 1.42 (1.07, 1.77) |
| Jamaica | 2.92 (0.26, 5.59) | 0 (0, 0.01) | 0 (0, 0) | 2.92 (0.25, 5.58) | 2.92 (0.26, 5.59) |
| Japan | 0.04 (0.03, 0.06) | 0.03 (0.02, 0.05) | 0 (0, 0) | 0.01 (0, 0.01) | 0.04 (0.03, 0.06) |
| Jordan | 3.07 (1.64, 4.5) | 2.89 (1.49, 4.28) | 0 (0, 0) | 0.18 (0, 0.48) | 3.07 (1.64, 4.5) |
| Kazakhstan | 1.77 (0, 4.86) | 1.77 (0, 4.86) | 0 (0, 0) | 0 (0, 0) | 1.77 (0, 4.86) |
| Kenya | 1128.09 (619.95, 1636.23) | 1090.46 (582.74, 1598.18) | 30.54 (12.38, 48.7) | 7.08 (0, 16.95) | 56.53 (33.88, 79.19) |
| Kiribati | 94.99 (0, 224.9) | 0 (0, 0) | 32.58 (13.08, 52.07) | 62.41 (0, 190.86) | 94.99 (0, 224.9) |
| Kuwait | 2.25 (0.75, 3.75) | 1.66 (0.66, 2.66) | 0 (0, 0) | 0.59 (0, 1.7) | 2.25 (0.75, 3.75) |
| Kyrgyzstan | 8.48 (0, 31.59) | 8.48 (0, 31.59) | 0 (0, 0) | 0 (0, 0) | 8.48 (0, 31.59) |
| Lao People's Democratic Republic | 48.48 (28.7, 68.26) | 8.25 (5.03, 11.47) | 25.84 (10.17, 41.51) | 14.39 (2.75, 26.03) | 40.23 (20.71, 59.75) |
| Latvia | 0 (0, 0) | 0 (0, 0) | 0 (0, 0) | 0 (0, 0) | 0 (0, 0) |
| Lebanon | 3.24 (0.62, 5.86) | 2.9 (0.35, 5.45) | 0 (0, 0) | 0.34 (0, 0.94) | 3.24 (0.62, 5.86) |
| Lesotho | 0 (0, 0) | 0 (0, 0) | 0 (0, 0) | 0 (0, 0) | 0 (0, 0) |
| Liberia | 8447.13 (2972.09, 13922.16) | 6836.6 (1401.46, 12271.75) | 1561.4 (904.47, 2218.32) | 49.13 (0, 109.36) | 1610.52 (950.84, 2270.2) |
| Libya | 47.05 (19.61, 74.49) | 47.05 (19.61, 74.49) | 0 (0, 0) | 0 (0, 0) | 47.05 (19.61, 74.49) |
| Lithuania | 0 (0, 0) | 0 (0, 0) | 0 (0, 0) | 0 (0, 0) | 0 (0, 0) |
| Luxembourg | 0.02 (0.01, 0.03) | 0.02 (0.01, 0.03) | 0 (0, 0) | 0 (0, 0) | 0.02 (0.01, 0.03) |
| Madagascar | 1531.27 (119.61, 2942.93) | 1501.44 (89.9, 2912.99) | 29.46 (11.67, 47.25) | 0.37 (0, 1.82) | 29.83 (11.98, 47.68) |
| Malawi | 3044.27 (626.84, 5461.71) | 2970.63 (553.39, 5387.87) | 70.57 (40.2, 100.94) | 3.07 (0, 7.31) | 172.69 (0, 454.14) |
| Malaysia | 88.14 (48.11, 128.17) | 0.05 (0.03, 0.07) | 30.11 (11.75, 48.46) | 57.98 (22.41, 93.56) | 88.09 (48.06, 128.12) |
| Maldives | 113.03 (10.55, 215.5) | 0 (0, 0) | 26.69 (9.75, 43.63) | 86.34 (0, 187.4) | 113.03 (10.55, 215.5) |
| Mali | 5402.51 (1355.84, 9449.19) | 5355.72 (1309.13, 9402.3) | 34.43 (13.98, 54.88) | 12.37 (0, 29.99) | 49.45 (22.42, 76.47) |
| Malta | 6.99 (0, 36.86) | 6.99 (0, 36.86) | 0 (0, 0) | 0 (0, 0) | 6.99 (0, 36.86) |
| Marshall Islands | 115.03 (0, 331.34) | 0 (0, 0) | 14.76 (9.17, 20.34) | 100.28 (0, 316.51) | 115.03 (0, 331.34) |
| Mauritania | 550.86 (41.46, 1060.26) | 535.69 (26.8, 1044.58) | 0 (0, 0) | 15.17 (0, 37.84) | 18.88 (0, 41.61) |
| Mauritius | 9.83 (0, 31.19) | 0 (0, 0) | 0 (0, 0) | 9.83 (0, 31.19) | 9.83 (0, 31.19) |
| Mexico | 20.59 (14.68, 26.5) | 9.29 (6.09, 12.5) | 0 (0, 0) | 11.3 (6.33, 16.27) | 20.5 (14.59, 26.42) |
| Micronesia (Federated States of) | 47.48 (20.92, 74.04) | 0 (0, 0) | 42.77 (17, 68.54) | 4.72 (0, 11.16) | 47.48 (20.92, 74.04) |
| Monaco | 27.86 (19.93, 35.79) | 27.86 (19.93, 35.79) | 0 (0, 0) | 0 (0, 0) | 27.86 (19.93, 35.79) |
| Mongolia | 0 (0, 0) | 0 (0, 0) | 0 (0, 0) | 0 (0, 0) | 0 (0, 0) |
| Montenegro | 5.35 (0, 30.58) | 5.35 (0, 30.58) | 0 (0, 0) | 0 (0, 0) | 5.35 (0, 30.58) |
| Morocco | 14.77 (2.36, 27.19) | 14.77 (2.36, 27.19) | 0 (0, 0) | 0 (0, 0) | 14.77 (2.36, 27.19) |
| Mozambique | 5667.12 (467.2, 10867.04) | 5637.63 (437.74, 10837.52) | 28.34 (11.82, 44.85) | 1.15 (0, 5.83) | 29.49 (12.33, 46.66) |
| Myanmar | 131.69 (74.46, 188.92) | 47.32 (7.92, 86.72) | 34.51 (14, 55.01) | 49.86 (13.77, 85.95) | 84.36 (42.85, 125.87) |
| Namibia | 98.74 (0, 303.44) | 98.74 (0, 303.44) | 0 (0, 0) | 0 (0, 0) | 0.99 (0.46, 1.52) |
| Nauru | 10.68 (0, 33.77) | 0 (0, 0) | 0 (0, 0) | 10.68 (0, 33.77) | 10.68 (0, 33.77) |
| Nepal | 109.62 (55.86, 163.38) | 13.66 (0, 42.75) | 43.84 (18.59, 69.09) | 52.12 (14.62, 89.61) | 107.5 (53.76, 161.25) |
| Netherlands | 0.03 (0.02, 0.05) | 0.03 (0.02, 0.05) | 0 (0, 0) | 0 (0, 0) | 0.03 (0.02, 0.05) |
| New Zealand | 0 (0, 0) | 0 (0, 0) | 0 (0, 0) | 0 (0, 0) | 0 (0, 0) |
| Nicaragua | 177.54 (103.95, 251.12) | 165.57 (93.16, 237.97) | 0 (0, 0) | 11.97 (0, 25.07) | 101.18 (66.29, 136.07) |
| Niger | 7464.21 (2052.27, 12876.15) | 7374.3 (1963.12, 12785.48) | 33.95 (13.09, 54.82) | 55.96 (0, 144.27) | 119.29 (27.27, 211.32) |
| Nigeria | 6211.54 (1100.32, 11322.75) | 6018.01 (907.31, 11128.71) | 152.47 (88.1, 216.83) | 41.06 (7.84, 74.28) | 194.54 (122.11, 266.97) |
| Niue | 35.06 (14.62, 55.51) | 0 (0, 0) | 33.45 (13.1, 53.8) | 1.61 (0, 3.55) | 35.06 (14.62, 55.51) |
| North Macedonia | 3.75 (0, 20.72) | 3.75 (0, 20.72) | 0 (0, 0) | 0 (0, 0) | 3.75 (0, 20.72) |
| Northern Mariana Islands | 8.02 (0, 20.11) | 0 (0, 0) | 0 (0, 0) | 8.02 (0, 20.11) | 8.02 (0, 20.11) |
| Norway | 0 (0, 0) | 0 (0, 0) | 0 (0, 0) | 0 (0, 0) | 0 (0, 0) |
| Oman | 16.46 (7.42, 25.49) | 2.62 (1.34, 3.9) | 0 (0, 0) | 13.84 (4.89, 22.78) | 15.07 (6.05, 24.1) |
| Pakistan | 280.17 (0, 606.17) | 250.35 (0, 575.38) | 0 (0, 0) | 29.82 (4.63, 55.01) | 40.61 (15.06, 66.16) |
| Palau | 49.38 (0, 145.05) | 0 (0, 0) | 14.64 (9.09, 20.18) | 34.75 (0, 130.26) | 49.38 (0, 145.05) |
| Palestine | 6.98 (0, 14.64) | 6.64 (0, 14.28) | 0 (0, 0) | 0.34 (0, 0.78) | 6.98 (0, 14.64) |
| Panama | 96.18 (62.79, 129.57) | 82.65 (53.16, 112.14) | 0 (0, 0) | 13.53 (0, 29.2) | 95.02 (61.65, 128.39) |
| Papua New Guinea | 1447.46 (543.01, 2351.91) | 1259.2 (357.8, 2160.6) | 187.5 (113.32, 261.68) | 0.76 (0.13, 1.4) | 188.26 (114.07, 262.44) |
| Paraguay | 79.89 (43.94, 115.84) | 40.36 (17.32, 63.39) | 0 (0, 0) | 39.53 (11.93, 67.13) | 79.89 (43.94, 115.84) |
| Peru | 62.97 (42.2, 83.75) | 49.56 (32.84, 66.28) | 0 (0, 0) | 13.41 (1.08, 25.74) | 57.98 (39.17, 76.79) |
| Philippines | 157.47 (118.68, 196.26) | 1.36 (0, 4.02) | 29.51 (12.01, 47.01) | 126.6 (92.09, 161.12) | 156.11 (117.42, 194.81) |
| Poland | 0 (0, 0) | 0 (0, 0) | 0 (0, 0) | 0 (0, 0) | 0 (0, 0) |
| Portugal | 1.07 (0, 4.69) | 1.07 (0, 4.69) | 0 (0, 0) | 0 (0, 0) | 1.07 (0, 4.69) |
| Puerto Rico | 17.22 (0, 54.23) | 0.2 (0.11, 0.29) | 0 (0, 0) | 17.02 (0, 54.03) | 17.22 (0, 54.23) |
| Qatar | 0.07 (0.01, 0.12) | 0 (0, 0) | 0 (0, 0) | 0.07 (0.01, 0.12) | 0.07 (0.01, 0.12) |
| Republic of Korea | 0.1 (0.08, 0.12) | 0.1 (0.08, 0.12) | 0 (0, 0) | 0 (0, 0) | 0 (0, 0) |
| Republic of Moldova | 0 (0, 0) | 0 (0, 0) | 0 (0, 0) | 0 (0, 0) | 0 (0, 0) |
| Romania | 0.13 (0, 0.74) | 0.13 (0, 0.74) | 0 (0, 0) | 0 (0, 0) | 0.13 (0, 0.74) |
| Russian Federation | 0 (0, 0) | 0 (0, 0) | 0 (0, 0) | 0 (0, 0) | 0 (0, 0) |
| Rwanda | 1463.62 (626.88, 2300.37) | 1455.25 (618.6, 2291.9) | 0 (0, 0) | 8.37 (0, 20.92) | 8.37 (0, 20.92) |
| Saint Kitts and Nevis | 0.85 (0, 3.66) | 0 (0, 0) | 0 (0, 0) | 0.85 (0, 3.66) | 0.85 (0, 3.66) |
| Saint Lucia | 4.25 (0.79, 7.71) | 0.01 (0, 0.02) | 0 (0, 0) | 4.24 (0.78, 7.7) | 4.25 (0.79, 7.71) |
| Saint Vincent and the Grenadines | 3.38 (0, 9) | 0 (0, 0) | 0 (0, 0) | 3.38 (0, 9) | 3.38 (0, 9) |
| Samoa | 141.48 (81.16, 201.8) | 0 (0, 0) | 135.65 (75.89, 195.41) | 5.83 (0, 14.01) | 141.48 (81.16, 201.8) |
| San Marino | 0 (0, 0) | 0 (0, 0) | 0 (0, 0) | 0 (0, 0) | 0 (0, 0) |
| Sao Tome and Principe | 369.96 (203.94, 535.98) | 292.22 (133.39, 451.04) | 53.7 (23.01, 84.4) | 24.04 (0, 61.4) | 77.75 (29.39, 126.1) |
| Saudi Arabia | 29.1 (13.85, 44.35) | 28.31 (13.07, 43.55) | 0 (0, 0) | 0.79 (0.22, 1.37) | 28.1 (12.86, 43.35) |
| Senegal | 1879.61 (925.2, 2834.02) | 1839.96 (885.85, 2794.07) | 26.53 (10.64, 42.42) | 13.12 (0, 31.06) | 47.9 (23.55, 72.25) |
| Serbia | 0.32 (0, 1.88) | 0.32 (0, 1.88) | 0 (0, 0) | 0 (0, 0) | 0.32 (0, 1.88) |
| Seychelles | 124.1 (0, 345.4) | 0 (0, 0) | 0 (0, 0) | 124.1 (0, 345.4) | 124.1 (0, 345.4) |
| Sierra Leone | 9469.11 (2299.46, 16638.75) | 8940.31 (1775.95, 16104.67) | 480.2 (212.08, 748.32) | 48.6 (0, 110.7) | 528.8 (253.58, 804.02) |
| Singapore | 85.3 (0, 185.86) | 0 (0, 0) | 0 (0, 0) | 85.3 (0, 185.86) | 85.3 (0, 185.86) |
| Slovakia | 0 (0, 0) | 0 (0, 0) | 0 (0, 0) | 0 (0, 0) | 0 (0, 0) |
| Slovenia | 0.63 (0, 2.97) | 0.63 (0, 2.97) | 0 (0, 0) | 0 (0, 0) | 0.63 (0, 2.97) |
| Solomon Islands | 1442.96 (636.99, 2248.92) | 1432.1 (626.25, 2237.94) | 0 (0, 0) | 10.86 (0, 24.86) | 10.86 (0, 24.86) |
| Somalia | 783.17 (122.64, 1443.71) | 734.24 (77.83, 1390.65) | 0 (0, 0) | 48.93 (0, 122.63) | 86.81 (9.68, 163.95) |
| South Africa | 6.81 (0, 18.05) | 6.81 (0, 18.05) | 0 (0, 0) | 0 (0, 0) | 0.02 (0.01, 0.03) |
| South Sudan | 6360.46 (3002.18, 9718.75) | 4890.81 (1584.74, 8196.88) | 1417.75 (832.3, 2003.2) | 51.91 (0, 124.13) | 1937.46 (1297.65, 2577.27) |
| Spain | 1.67 (0, 6.33) | 1.67 (0, 6.33) | 0 (0, 0) | 0 (0, 0) | 1.67 (0, 6.33) |
| Sri Lanka | 63.26 (25.99, 100.53) | 3.72 (2.19, 5.25) | 0 (0, 0) | 59.54 (22.3, 96.78) | 63.26 (25.99, 100.53) |
| Sudan | 772.6 (200.74, 1344.47) | 682.81 (115.28, 1250.34) | 42.45 (23.87, 61.02) | 47.34 (0, 115.09) | 177.27 (0, 387.94) |
| Suriname | 116.1 (73.3, 158.9) | 97.98 (57.96, 138) | 0 (0, 0) | 18.12 (2.95, 33.3) | 113.23 (70.52, 155.94) |
| Sweden | 0.08 (0.05, 0.12) | 0.08 (0.05, 0.12) | 0 (0, 0) | 0 (0, 0) | 0.08 (0.05, 0.12) |
| Switzerland | 0.14 (0.08, 0.2) | 0.14 (0.08, 0.2) | 0 (0, 0) | 0 (0, 0) | 0.14 (0.08, 0.2) |
| Syrian Arab Republic | 303.1 (191.76, 414.45) | 303.05 (191.7, 414.39) | 0 (0, 0) | 0.05 (0, 0.12) | 303.1 (191.76, 414.45) |
| Tajikistan | 15.51 (0, 31.08) | 15.51 (0, 31.08) | 0 (0, 0) | 0 (0, 0) | 15.51 (0, 31.08) |
| Thailand | 21.63 (11.76, 31.5) | 0.84 (0.32, 1.36) | 0 (0, 0) | 20.79 (10.94, 30.64) | 21.07 (11.21, 30.93) |
| Timor-Leste | 245.34 (159.84, 330.84) | 0 (0, 0) | 225.37 (140.94, 309.8) | 19.96 (6.5, 33.43) | 245.34 (159.84, 330.84) |
| Togo | 4338.54 (1107.65, 7569.43) | 4325.44 (1094.62, 7556.26) | 2.73 (0.69, 4.77) | 10.37 (0, 31.41) | 13.1 (0, 34.24) |
| Tokelau | 0.87 (0, 1.74) | 0 (0, 0) | 0 (0, 0) | 0.87 (0, 1.74) | 0.87 (0, 1.74) |
| Tonga | 193.96 (0, 468.93) | 0 (0, 0) | 25.31 (10.38, 40.24) | 168.65 (0, 443.21) | 193.96 (0, 468.93) |
| Trinidad and Tobago | 22.17 (5.57, 38.76) | 0.02 (0.01, 0.03) | 0 (0, 0) | 22.15 (5.55, 38.74) | 22.17 (5.57, 38.76) |
| Tunisia | 56.08 (32.83, 79.32) | 56.08 (32.83, 79.32) | 0 (0, 0) | 0 (0, 0) | 56.08 (32.83, 79.32) |
| Türkiye | 7 (3.03, 10.96) | 7 (3.03, 10.96) | 0 (0, 0) | 0 (0, 0) | 7 (3.03, 10.96) |
| Turkmenistan | 60.78 (30.24, 91.32) | 60.78 (30.24, 91.32) | 0 (0, 0) | 0 (0, 0) | 60.78 (30.24, 91.32) |
| Tuvalu | 4.81 (0, 15.3) | 0 (0, 0) | 0 (0, 0) | 4.81 (0, 15.3) | 4.81 (0, 15.3) |
| Uganda | 6127.67 (1617.49, 10637.85) | 6011.76 (1501.78, 10521.73) | 102.8 (64.03, 141.58) | 13.11 (0, 32.26) | 119.04 (75.77, 162.31) |
| Ukraine | 0 (0, 0) | 0 (0, 0) | 0 (0, 0) | 0 (0, 0) | 0 (0, 0) |
| United Arab Emirates | 0 (0, 0) | 0 (0, 0) | 0 (0, 0) | 0 (0, 0) | 0 (0, 0) |
| United Kingdom | 0 (0, 0.01) | 0 (0, 0.01) | 0 (0, 0) | 0 (0, 0) | 0 (0, 0.01) |
| United Republic of Tanzania | 1699.23 (316.74, 3081.72) | 1623.13 (240.92, 3005.34) | 67.09 (42.72, 91.47) | 9 (0, 22.32) | 76.09 (48.32, 103.87) |
| United States of America | 0.6 (0.35, 0.84) | 0.58 (0.34, 0.83) | 0 (0, 0) | 0.02 (0, 0.03) | 0.6 (0.35, 0.84) |
| United States Virgin Islands | 1.93 (0, 7.82) | 0 (0, 0) | 0 (0, 0) | 1.93 (0, 7.82) | 1.93 (0, 7.82) |
| Uruguay | 5.5 (3.59, 7.41) | 5.5 (3.59, 7.41) | 0 (0, 0) | 0 (0, 0) | 5.5 (3.59, 7.41) |
| Uzbekistan | 7.48 (1.96, 12.99) | 7.48 (1.96, 12.99) | 0 (0, 0) | 0 (0, 0) | 7.48 (1.96, 12.99) |
| Vanuatu | 65.01 (34.93, 95.08) | 8.25 (5.78, 10.73) | 48.58 (20.64, 76.52) | 8.17 (0, 19.02) | 56.76 (26.78, 86.73) |
| Venezuela (Bolivarian Republic of) | 212.82 (103.01, 322.63) | 184.95 (77.18, 292.71) | 1.63 (0.5, 2.77) | 26.24 (5.16, 47.31) | 126.07 (92.02, 160.13) |
| Viet Nam | 44.86 (22.9, 66.83) | 0.27 (0.16, 0.39) | 27.75 (11.04, 44.46) | 16.84 (2.58, 31.09) | 44.59 (22.62, 66.55) |
| Yemen | 1720.22 (0, 3502.28) | 1695.11 (0, 3477.1) | 24.86 (9.96, 39.75) | 0.26 (0, 0.94) | 78.39 (44.7, 112.07) |
| Zambia | 2295.94 (233.83, 4358.05) | 2250.15 (188.19, 4312.11) | 40.27 (16.7, 63.84) | 5.51 (0, 12.89) | 79.74 (26.98, 132.5) |
| Zimbabwe | 713.07 (172.19, 1253.95) | 685.3 (144.67, 1225.93) | 27.7 (11.41, 43.99) | 0.08 (0, 0.33) | 27.77 (11.48, 44.07) |

ASDRs age-standardized DALYs rates

**Table H** ASDRs in 2021 for nine arthropod-borne diseases in 204 countries and territories

| **Location** | **Malaria** | **Chagas disease** | **Lymphatic filariasis** | **African trypanosomiasis** | **Leishmaniasis** | **Onchocerciasis** | **Dengue** | **Yellow fever** | **Zika virus** |
| --- | --- | --- | --- | --- | --- | --- | --- | --- | --- |
| Afghanistan | 28.87  (15.48, 42.25) | 0.00  (0.00, 0.00) | 0.00  (0.00, 0.00) | 0.00  (0.00, 0.00) | 0.00  (0.00, 0.00) | 0.00  (0.00, 0.00) | 0.05  (0.00, 0.12) | 0.00  (0.00, 0.00) | 0.00  (0.00, 0.00) |
| Albania | 0.00  (0.00, 0.00) | 0.00  (0.00, 0.00) | 0.00  (0.00, 0.00) | 0.00  (0.00, 0.00) | 0.00  (0.00, 0.00) | 0.00  (0.00, 0.00) | 0.00  (0.00, 0.00) | 0.00  (0.00, 0.00) | 0.00  (0.00, 0.00) |
| Algeria | 0.00  (0.00, 0.00) | 0.00  (0.00, 0.00) | 0.00  (0.00, 0.00) | 0.00  (0.00, 0.00) | 0.00  (0.00, 0.00) | 0.00  (0.00, 0.00) | 0.00  (0.00, 0.00) | 0.00  (0.00, 0.00) | 0.00  (0.00, 0.00) |
| American Samoa | 0.00  (0.00, 0.00) | 0.00  (0.00, 0.00) | 73.12  (33.48, 112.77) | 0.00  (0.00, 0.00) | 73.12  (33.48, 112.77) | 0.00  (0.00, 0.00) | 23.64  (0.00, 62.57) | 0.00  (0.00, 0.00) | 0.00  (0.00, 0.00) |
| Andorra | 0.00  (0.00, 0.00) | 0.15  (0.06, 0.23) | 0.00  (0.00, 0.00) | 0.00  (0.00, 0.00) | 0.00  (0.00, 0.00) | 0.00  (0.00, 0.00) | 0.00  (0.00, 0.00) | 0.00  (0.00, 0.00) | 0.00  (0.00, 0.00) |
| Angola | 3417.13  (546.47, 6287.78) | 0.00  (0.00, 0.00) | 29.70  (11.81, 47.58) | 11.75  (3.66, 19.84) | 29.70  (11.81, 47.58) | 37.21  (19.28, 55.15) | 2.30  (0.00, 9.63) | 51.94  (0.00, 131.55) | 0.00  (0.00, 0.00) |
| Antigua and Barbuda | 0.00  (0.00, 0.00) | 0.01  (0.00, 0.02) | 0.00  (0.00, 0.00) | 0.00  (0.00, 0.00) | 0.00  (0.00, 0.00) | 0.00  (0.00, 0.00) | 0.69  (0.00, 1.51) | 0.00  (0.00, 0.00) | 0.03  (0.00, 0.09) |
| Argentina | 0.00  (0.00, 0.00) | 28.62  (19.66, 37.58) | 0.00  (0.00, 0.00) | 0.00  (0.00, 0.00) | 0.00  (0.00, 0.00) | 0.00  (0.00, 0.00) | 1.80  (0.00, 4.06) | 4.45  (0.00, 11.63) | 0.00  (0.00, 0.00) |
| Armenia | 0.00  (0.00, 0.00) | 0.00  (0.00, 0.00) | 0.00  (0.00, 0.00) | 0.00  (0.00, 0.00) | 0.00  (0.00, 0.00) | 0.00  (0.00, 0.00) | 0.00  (0.00, 0.00) | 0.00  (0.00, 0.00) | 0.00  (0.00, 0.00) |
| Australia | 0.00  (0.00, 0.00) | 0.05  (0.03, 0.07) | 0.00  (0.00, 0.00) | 0.00  (0.00, 0.00) | 0.00  (0.00, 0.00) | 0.00  (0.00, 0.00) | 0.74  (0.00, 1.61) | 0.00  (0.00, 0.00) | 0.00  (0.00, 0.00) |
| Austria | 0.00  (0.00, 0.00) | 0.02  (0.01, 0.02) | 0.00  (0.00, 0.00) | 0.00  (0.00, 0.00) | 0.00  (0.00, 0.00) | 0.00  (0.00, 0.00) | 0.00  (0.00, 0.00) | 0.00  (0.00, 0.00) | 0.00  (0.00, 0.00) |
| Azerbaijan | 0.00  (0.00, 0.00) | 0.00  (0.00, 0.00) | 0.00  (0.00, 0.00) | 0.00  (0.00, 0.00) | 0.00  (0.00, 0.00) | 0.00  (0.00, 0.00) | 0.00  (0.00, 0.00) | 0.00  (0.00, 0.00) | 0.00  (0.00, 0.00) |
| Bahamas | 0.00  (0.00, 0.00) | 0.02  (0.01, 0.03) | 0.00  (0.00, 0.00) | 0.00  (0.00, 0.00) | 0.00  (0.00, 0.00) | 0.00  (0.00, 0.00) | 13.29  (0.00, 37.40) | 0.00  (0.00, 0.00) | 0.02  (0.00, 0.06) |
| Bahrain | 0.00  (0.00, 0.00) | 0.00  (0.00, 0.00) | 0.00  (0.00, 0.00) | 0.00  (0.00, 0.00) | 0.00  (0.00, 0.00) | 0.00  (0.00, 0.00) | 0.12  (0.03, 0.21) | 0.00  (0.00, 0.00) | 0.00  (0.00, 0.00) |
| Bangladesh | 20.83  (3.34, 38.31) | 0.00  (0.00, 0.00) | 23.32  (10.33, 36.32) | 0.00  (0.00, 0.00) | 23.32  (10.33, 36.32) | 0.00  (0.00, 0.00) | 4.82  (0.00, 9.69) | 0.00  (0.00, 0.00) | 0.00  (0.00, 0.00) |
| Barbados | 0.00  (0.00, 0.00) | 0.00  (0.00, 0.00) | 0.00  (0.00, 0.00) | 0.00  (0.00, 0.00) | 0.00  (0.00, 0.00) | 0.00  (0.00, 0.00) | 23.00  (0.00, 53.02) | 0.00  (0.00, 0.00) | 0.02  (0.00, 0.05) |
| Belarus | 0.00  (0.00, 0.00) | 0.00  (0.00, 0.00) | 0.00  (0.00, 0.00) | 0.00  (0.00, 0.00) | 0.00  (0.00, 0.00) | 0.00  (0.00, 0.00) | 0.00  (0.00, 0.00) | 0.00  (0.00, 0.00) | 0.00  (0.00, 0.00) |
| Belgium | 0.00  (0.00, 0.00) | 0.01  (0.00, 0.01) | 0.00  (0.00, 0.00) | 0.00  (0.00, 0.00) | 0.00  (0.00, 0.00) | 0.00  (0.00, 0.00) | 0.00  (0.00, 0.00) | 0.00  (0.00, 0.00) | 0.00  (0.00, 0.00) |
| Belize | 0.00  (0.00, 0.00) | 0.42  (0.29, 0.54) | 0.00  (0.00, 0.00) | 0.00  (0.00, 0.00) | 0.00  (0.00, 0.00) | 0.00  (0.00, 0.00) | 4.77  (0.00, 12.01) | 0.00  (0.00, 0.00) | 0.11  (0.00, 0.28) |
| Benin | 6891.46  (2316.49, 11466.44) | 0.00  (0.00, 0.00) | 27.37  (11.08, 43.67) | 0.00  (0.00, 0.00) | 27.37  (11.08, 43.67) | 21.36  (8.02, 34.71) | 4.63  (0.00, 21.11) | 20.91  (0.00, 48.39) | 0.00  (0.00, 0.00) |
| Bermuda | 0.00  (0.00, 0.00) | 0.00  (0.00, 0.00) | 0.00  (0.00, 0.00) | 0.00  (0.00, 0.00) | 0.00  (0.00, 0.00) | 0.00  (0.00, 0.00) | 0.00  (0.00, 0.00) | 0.00  (0.00, 0.00) | 0.00  (0.00, 0.00) |
| Bhutan | 2.96  (1.94, 3.97) | 0.00  (0.00, 0.00) | 0.00  (0.00, 0.00) | 0.00  (0.00, 0.00) | 0.00  (0.00, 0.00) | 0.00  (0.00, 0.00) | 11.27  (0.82, 21.72) | 0.00  (0.00, 0.00) | 0.00  (0.00, 0.00) |
| Bolivia  (Plurinational State of) | 1.73  (1.02, 2.44) | 137.04  (81.85, 192.23) | 0.00  (0.00, 0.00) | 0.00  (0.00, 0.00) | 0.00  (0.00, 0.00) | 0.00  (0.00, 0.00) | 7.41  (0.00, 17.24) | 1.53  (0.00, 3.83) | 0.01  (0.00, 0.01) |
| Bosnia and Herzegovina | 0.00  (0.00, 0.00) | 0.00  (0.00, 0.00) | 0.00  (0.00, 0.00) | 0.00  (0.00, 0.00) | 0.00  (0.00, 0.00) | 0.00  (0.00, 0.00) | 0.00  (0.00, 0.00) | 0.00  (0.00, 0.00) | 0.00  (0.00, 0.00) |
| Botswana | 27.85  (12.53, 43.16) | 0.00  (0.00, 0.00) | 0.00  (0.00, 0.00) | 0.00  (0.00, 0.00) | 0.00  (0.00, 0.00) | 0.00  (0.00, 0.00) | 0.00  (0.00, 0.00) | 0.00  (0.00, 0.00) | 0.00  (0.00, 0.00) |
| Brazil | 6.22  (0.00, 13.56) | 60.22  (53.01, 67.43) | 0.70  (0.44, 0.97) | 0.00  (0.00, 0.00) | 0.70  (0.44, 0.97) | 0.01  (0.00, 0.02) | 64.77  (6.53, 123.02) | 0.11  (0.01, 0.22) | 0.04  (0.03, 0.06) |
| Brunei Darussalam | 0.00  (0.00, 0.00) | 0.00  (0.00, 0.00) | 29.70  (11.36, 48.04) | 0.00  (0.00, 0.00) | 29.70  (11.36, 48.04) | 0.00  (0.00, 0.00) | 4.61  (0.06, 9.17) | 0.00  (0.00, 0.00) | 0.00  (0.00, 0.00) |
| Bulgaria | 0.00  (0.00, 0.00) | 0.00  (0.00, 0.00) | 0.00  (0.00, 0.00) | 0.00  (0.00, 0.00) | 0.00  (0.00, 0.00) | 0.00  (0.00, 0.00) | 0.00  (0.00, 0.00) | 0.00  (0.00, 0.00) | 0.00  (0.00, 0.00) |
| Burkina Faso | 8938.18  (3146.18, 14730.18) | 0.00  (0.00, 0.00) | 26.28  (10.66, 41.90) | 0.00  (0.00, 0.00) | 26.28  (10.66, 41.90) | 0.36  (0.05, 0.68) | 1.50  (0.00, 5.89) | 30.91  (0.00, 76.53) | 0.00  (0.00, 0.00) |
| Burundi | 5978.63  (1638.79, 10318.48) | 0.00  (0.00, 0.00) | 0.00  (0.00, 0.00) | 0.00  (0.00, 0.00) | 0.00  (0.00, 0.00) | 179.17  (99.03, 259.30) | 0.20  (0.00, 0.78) | 93.38  (0.00, 224.99) | 0.00  (0.00, 0.00) |
| Cabo Verde | 24.02  (16.08, 31.96) | 0.00  (0.00, 0.00) | 0.00  (0.00, 0.00) | 0.00  (0.00, 0.00) | 0.00  (0.00, 0.00) | 0.00  (0.00, 0.00) | 73.73  (0.00, 284.73) | 0.00  (0.00, 0.00) | 0.00  (0.00, 0.00) |
| Cambodia | 5.74  (3.85, 7.63) | 0.00  (0.00, 0.00) | 0.00  (0.00, 0.00) | 0.00  (0.00, 0.00) | 0.00  (0.00, 0.00) | 0.00  (0.00, 0.00) | 20.47  (4.35, 36.58) | 0.00  (0.00, 0.00) | 0.00  (0.00, 0.00) |
| Cameroon | 5668.07  (1160.65, 10175.49) | 0.00  (0.00, 0.00) | 23.25  (10.03, 36.47) | 6.40  (1.80, 11.00) | 23.25  (10.03, 36.47) | 348.44  (200.71, 496.18) | 3.40  (0.00, 16.22) | 3.86  (0.00, 8.99) | 0.00  (0.00, 0.00) |
| Canada | 0.00  (0.00, 0.00) | 0.09  (0.05, 0.13) | 0.00  (0.00, 0.00) | 0.00  (0.00, 0.00) | 0.00  (0.00, 0.00) | 0.00  (0.00, 0.00) | 0.00  (0.00, 0.00) | 0.00  (0.00, 0.00) | 0.00  (0.00, 0.00) |
| Central African Republic | 5663.66  (382.08, 10945.25) | 0.00  (0.00, 0.00) | 74.29  (35.66, 112.92) | 30.07  (8.71, 51.43) | 74.29  (35.66, 112.92) | 522.81  (306.85, 738.77) | 2.44  (0.00, 11.08) | 12.55  (0.00, 30.50) | 0.00  (0.00, 0.00) |
| Chad | 2831.17  (448.00, 5214.35) | 0.00  (0.00, 0.00) | 27.57  (10.63, 44.51) | 0.46  (0.15, 0.77) | 27.57  (10.63, 44.51) | 142.16  (73.87, 210.45) | 0.50  (0.00, 2.53) | 35.24  (0.00, 86.48) | 0.00  (0.00, 0.00) |
| Chile | 0.00  (0.00, 0.00) | 15.71  (10.72, 20.70) | 0.00  (0.00, 0.00) | 0.00  (0.00, 0.00) | 0.00  (0.00, 0.00) | 0.00  (0.00, 0.00) | 0.00  (0.00, 0.00) | 0.00  (0.00, 0.00) | 0.00  (0.00, 0.00) |
| China | 0.00  (0.00, 0.00) | 0.00  (0.00, 0.00) | 0.00  (0.00, 0.00) | 0.00  (0.00, 0.00) | 0.00  (0.00, 0.00) | 0.00  (0.00, 0.00) | 2.52  (0.00, 5.66) | 0.00  (0.00, 0.00) | 0.00  (0.00, 0.00) |
| Colombia | 11.21  (2.62, 19.81) | 10.63  (7.78, 13.49) | 0.00  (0.00, 0.00) | 0.00  (0.00, 0.00) | 0.00  (0.00, 0.00) | 0.00  (0.00, 0.00) | 23.86  (6.76, 40.97) | 0.32  (0.00, 0.73) | 0.01  (0.00, 0.01) |
| Comoros | 234.27  (102.30, 366.23) | 0.00  (0.00, 0.00) | 65.51  (29.82, 101.19) | 0.00  (0.00, 0.00) | 65.51  (29.82, 101.19) | 0.00  (0.00, 0.00) | 117.57  (0.00, 522.40) | 0.00  (0.00, 0.00) | 0.00  (0.00, 0.00) |
| Congo | 2991.66  (780.74, 5202.58) | 0.00  (0.00, 0.00) | 28.59  (11.68, 45.50) | 83.57  (28.49, 138.65) | 28.59  (11.68, 45.50) | 45.70  (19.54, 71.86) | 2.99  (0.00, 15.05) | 38.05  (0.00, 90.11) | 0.00  (0.00, 0.00) |
| Cook Islands | 0.00  (0.00, 0.00) | 0.00  (0.00, 0.00) | 0.00  (0.00, 0.00) | 0.00  (0.00, 0.00) | 0.00  (0.00, 0.00) | 0.00  (0.00, 0.00) | 1.25  (0.00, 3.46) | 0.00  (0.00, 0.00) | 0.00  (0.00, 0.00) |
| Costa Rica | 0.26  (0.20, 0.31) | 3.87  (2.32, 5.43) | 0.00  (0.00, 0.00) | 0.00  (0.00, 0.00) | 0.00  (0.00, 0.00) | 0.00  (0.00, 0.00) | 25.79  (0.00, 61.22) | 0.00  (0.00, 0.00) | 0.01  (0.00, 0.02) |
| Côte d'Ivoire | 6120.71  (1752.54, 10488.88) | 0.00  (0.00, 0.00) | 159.96  (93.96, 225.96) | 0.42  (0.05, 0.80) | 159.96  (93.96, 225.96) | 0.35  (0.06, 0.63) | 2.26  (0.00, 11.18) | 7.99  (0.00, 19.44) | 0.00  (0.00, 0.00) |
| Croatia | 0.00  (0.00, 0.00) | 0.00  (0.00, 0.00) | 0.00  (0.00, 0.00) | 0.00  (0.00, 0.00) | 0.00  (0.00, 0.00) | 0.00  (0.00, 0.00) | 0.00  (0.00, 0.00) | 0.00  (0.00, 0.00) | 0.00  (0.00, 0.00) |
| Cuba | 0.00  (0.00, 0.00) | 0.00  (0.00, 0.00) | 0.00  (0.00, 0.00) | 0.00  (0.00, 0.00) | 0.00  (0.00, 0.00) | 0.00  (0.00, 0.00) | 1.74  (0.00, 4.27) | 0.00  (0.00, 0.00) | 0.05  (0.01, 0.08) |
| Cyprus | 0.00  (0.00, 0.00) | 0.00  (0.00, 0.00) | 0.00  (0.00, 0.00) | 0.00  (0.00, 0.00) | 0.00  (0.00, 0.00) | 0.00  (0.00, 0.00) | 0.00  (0.00, 0.00) | 0.00  (0.00, 0.00) | 0.00  (0.00, 0.00) |
| Czechia | 0.00  (0.00, 0.00) | 0.00  (0.00, 0.00) | 0.00  (0.00, 0.00) | 0.00  (0.00, 0.00) | 0.00  (0.00, 0.00) | 0.00  (0.00, 0.00) | 0.00  (0.00, 0.00) | 0.00  (0.00, 0.00) | 0.00  (0.00, 0.00) |
| Democratic People's Republic of Korea | 0.88  (0.58, 1.18) | 0.00  (0.00, 0.00) | 0.00  (0.00, 0.00) | 0.00  (0.00, 0.00) | 0.00  (0.00, 0.00) | 0.00  (0.00, 0.00) | 0.00  (0.00, 0.00) | 0.00  (0.00, 0.00) | 0.00  (0.00, 0.00) |
| Democratic Republic of the Congo | 4348.68  (1556.04, 7141.33) | 0.00  (0.00, 0.00) | 41.12  (17.04, 65.21) | 6.28  (2.46, 10.10) | 41.12  (17.04, 65.21) | 893.73  (523.90, 1263.57) | 1.64  (0.00, 7.11) | 11.00  (0.00, 26.48) | 0.00  (0.00, 0.00) |
| Denmark | 0.00  (0.00, 0.00) | 0.03  (0.02, 0.04) | 0.00  (0.00, 0.00) | 0.00  (0.00, 0.00) | 0.00  (0.00, 0.00) | 0.00  (0.00, 0.00) | 0.00  (0.00, 0.00) | 0.00  (0.00, 0.00) | 0.00  (0.00, 0.00) |
| Djibouti | 845.62  (457.67, 1233.57) | 0.00  (0.00, 0.00) | 0.00  (0.00, 0.00) | 0.00  (0.00, 0.00) | 0.00  (0.00, 0.00) | 0.00  (0.00, 0.00) | 6.10  (0.00, 29.51) | 0.00  (0.00, 0.00) | 0.00  (0.00, 0.00) |
| Dominica | 0.00  (0.00, 0.00) | 0.01  (0.00, 0.02) | 0.00  (0.00, 0.00) | 0.00  (0.00, 0.00) | 0.00  (0.00, 0.00) | 0.00  (0.00, 0.00) | 2.17  (0.00, 5.64) | 0.00  (0.00, 0.00) | 0.04  (0.00, 0.09) |
| Dominican Republic | 4.38  (0.00, 12.95) | 0.08  (0.05, 0.12) | 32.32  (13.08, 51.57) | 0.00  (0.00, 0.00) | 32.32  (13.08, 51.57) | 0.00  (0.00, 0.00) | 6.56  (0.00, 14.08) | 0.00  (0.00, 0.00) | 0.00  (0.00, 0.00) |
| Ecuador | 0.71  (0.43, 1.00) | 11.18  (7.77, 14.59) | 0.00  (0.00, 0.00) | 0.00  (0.00, 0.00) | 0.00  (0.00, 0.00) | 0.00  (0.00, 0.00) | 7.04  (0.61, 13.46) | 1.40  (0.00, 3.17) | 0.00  (0.00, 0.00) |
| Egypt | 0.00  (0.00, 0.00) | 0.00  (0.00, 0.00) | 0.00  (0.00, 0.00) | 0.00  (0.00, 0.00) | 0.00  (0.00, 0.00) | 0.00  (0.00, 0.00) | 0.28  (0.00, 1.11) | 0.00  (0.00, 0.00) | 0.00  (0.00, 0.00) |
| El Salvador | 0.00  (0.00, 0.00) | 10.99  (8.04, 13.93) | 0.00  (0.00, 0.00) | 0.00  (0.00, 0.00) | 0.00  (0.00, 0.00) | 0.00  (0.00, 0.00) | 19.28  (0.44, 38.12) | 0.00  (0.00, 0.00) | 0.16  (0.02, 0.30) |
| Equatorial Guinea | 4404.33  (811.11, 7997.55) | 0.00  (0.00, 0.00) | 32.11  (12.13, 52.09) | 17.78  (4.79, 30.76) | 32.11  (12.13, 52.09) | 196.09  (122.11, 270.07) | 1.24  (0.00, 6.07) | 13.88  (0.00, 34.16) | 0.00  (0.00, 0.00) |
| Eritrea | 157.27  (84.63, 229.91) | 0.00  (0.00, 0.00) | 24.84  (9.95, 39.73) | 0.00  (0.00, 0.00) | 24.84  (9.95, 39.73) | 0.00  (0.00, 0.00) | 0.12  (0.00, 0.52) | 10.12  (0.00, 25.62) | 0.00  (0.00, 0.00) |
| Estonia | 0.00  (0.00, 0.00) | 0.00  (0.00, 0.00) | 0.00  (0.00, 0.00) | 0.00  (0.00, 0.00) | 0.00  (0.00, 0.00) | 0.00  (0.00, 0.00) | 0.00  (0.00, 0.00) | 0.00  (0.00, 0.00) | 0.00  (0.00, 0.00) |
| Eswatini | 25.16  (0.00, 50.81) | 0.00  (0.00, 0.00) | 0.00  (0.00, 0.00) | 0.00  (0.00, 0.00) | 0.00  (0.00, 0.00) | 0.00  (0.00, 0.00) | 0.00  (0.00, 0.00) | 0.00  (0.00, 0.00) | 0.00  (0.00, 0.00) |
| Ethiopia | 351.94  (0.90, 702.99) | 0.00  (0.00, 0.00) | 26.34  (10.48, 42.20) | 0.00  (0.00, 0.00) | 26.34  (10.48, 42.20) | 44.14  (22.81, 65.47) | 0.96  (0.35, 1.57) | 33.93  (0.00, 70.29) | 0.00  (0.00, 0.00) |
| Fiji | 0.00  (0.00, 0.00) | 0.00  (0.00, 0.00) | 201.55  (124.11, 278.99) | 0.00  (0.00, 0.00) | 201.55  (124.11, 278.99) | 0.00  (0.00, 0.00) | 23.98  (4.15, 43.81) | 0.00  (0.00, 0.00) | 0.00  (0.00, 0.00) |
| Finland | 0.00  (0.00, 0.00) | 0.01  (0.01, 0.02) | 0.00  (0.00, 0.00) | 0.00  (0.00, 0.00) | 0.00  (0.00, 0.00) | 0.00  (0.00, 0.00) | 0.00  (0.00, 0.00) | 0.00  (0.00, 0.00) | 0.00  (0.00, 0.00) |
| France | 0.00  (0.00, 0.00) | 0.02  (0.01, 0.03) | 0.00  (0.00, 0.00) | 0.00  (0.00, 0.00) | 0.00  (0.00, 0.00) | 0.00  (0.00, 0.00) | 0.00  (0.00, 0.00) | 0.00  (0.00, 0.00) | 0.00  (0.00, 0.00) |
| Gabon | 2640.51  (97.52, 5183.50) | 0.00  (0.00, 0.00) | 32.19  (13.16, 51.22) | 204.90  (24.24, 385.56) | 32.19  (13.16, 51.22) | 0.00  (0.00, 0.00) | 4.29  (0.00, 21.28) | 29.19  (0.00, 66.52) | 0.00  (0.00, 0.00) |
| Gambia | 1475.35  (795.97, 2154.73) | 0.00  (0.00, 0.00) | 0.00  (0.00, 0.00) | 0.00  (0.00, 0.00) | 0.00  (0.00, 0.00) | 0.00  (0.00, 0.00) | 4.65  (0.00, 21.63) | 21.69  (0.00, 55.89) | 0.00  (0.00, 0.00) |
| Georgia | 0.00  (0.00, 0.00) | 0.00  (0.00, 0.00) | 0.00  (0.00, 0.00) | 0.00  (0.00, 0.00) | 0.00  (0.00, 0.00) | 0.00  (0.00, 0.00) | 0.00  (0.00, 0.00) | 0.00  (0.00, 0.00) | 0.00  (0.00, 0.00) |
| Germany | 0.00  (0.00, 0.00) | 0.01  (0.01, 0.02) | 0.00  (0.00, 0.00) | 0.00  (0.00, 0.00) | 0.00  (0.00, 0.00) | 0.00  (0.00, 0.00) | 0.00  (0.00, 0.00) | 0.00  (0.00, 0.00) | 0.00  (0.00, 0.00) |
| Ghana | 2882.44  (946.28, 4818.61) | 0.00  (0.00, 0.00) | 27.27  (11.52, 43.02) | 0.00  (0.00, 0.00) | 27.27  (11.52, 43.02) | 0.91  (0.36, 1.46) | 6.65  (0.00, 31.07) | 7.20  (0.00, 17.29) | 0.00  (0.00, 0.00) |
| Greece | 0.00  (0.00, 0.00) | 0.00  (0.00, 0.00) | 0.00  (0.00, 0.00) | 0.00  (0.00, 0.00) | 0.00  (0.00, 0.00) | 0.00  (0.00, 0.00) | 0.00  (0.00, 0.00) | 0.00  (0.00, 0.00) | 0.00  (0.00, 0.00) |
| Greenland | 0.00  (0.00, 0.00) | 0.00  (0.00, 0.00) | 0.00  (0.00, 0.00) | 0.00  (0.00, 0.00) | 0.00  (0.00, 0.00) | 0.00  (0.00, 0.00) | 0.00  (0.00, 0.00) | 0.00  (0.00, 0.00) | 0.00  (0.00, 0.00) |
| Grenada | 0.00  (0.00, 0.00) | 0.16  (0.05, 0.27) | 0.00  (0.00, 0.00) | 0.00  (0.00, 0.00) | 0.00  (0.00, 0.00) | 0.00  (0.00, 0.00) | 3.31  (0.00, 7.11) | 0.00  (0.00, 0.00) | 0.00  (0.00, 0.01) |
| Guam | 0.00  (0.00, 0.00) | 0.00  (0.00, 0.00) | 0.00  (0.00, 0.00) | 0.00  (0.00, 0.00) | 0.00  (0.00, 0.00) | 0.00  (0.00, 0.00) | 0.09  (0.02, 0.16) | 0.00  (0.00, 0.00) | 0.00  (0.00, 0.00) |
| Guatemala | 0.01  (0.00, 0.01) | 8.99  (6.32, 11.66) | 0.00  (0.00, 0.00) | 0.00  (0.00, 0.00) | 0.00  (0.00, 0.00) | 0.00  (0.00, 0.00) | 29.17  (12.26, 46.07) | 0.00  (0.00, 0.00) | 0.03  (0.01, 0.05) |
| Guinea | 5442.20  (1425.08, 9459.33) | 0.00  (0.00, 0.00) | 26.92  (10.70, 43.15) | 134.54  (14.75, 254.32) | 26.92  (10.70, 43.15) | 15.79  (3.84, 27.73) | 1.18  (0.00, 4.55) | 34.43  (0.00, 78.58) | 0.00  (0.00, 0.00) |
| Guinea-Bissau | 1768.55  (39.64, 3497.47) | 0.00  (0.00, 0.00) | 30.73  (12.43, 49.03) | 0.00  (0.00, 0.00) | 30.73  (12.43, 49.03) | 0.10  (0.02, 0.17) | 3.91  (0.00, 17.58) | 26.05  (0.00, 66.02) | 0.00  (0.00, 0.00) |
| Guyana | 433.23  (164.16, 702.29) | 0.35  (0.21, 0.50) | 322.77  (211.08, 434.45) | 0.00  (0.00, 0.00) | 322.77  (211.08, 434.45) | 0.00  (0.00, 0.00) | 4.37  (0.00, 9.17) | 4.99  (0.00, 11.91) | 0.00  (0.00, 0.00) |
| Haiti | 316.08  (0.00, 809.15) | 0.00  (0.00, 0.00) | 29.57  (11.52, 47.61) | 0.00  (0.00, 0.00) | 29.57  (11.52, 47.61) | 0.00  (0.00, 0.00) | 4.36  (0.00, 15.07) | 0.00  (0.00, 0.00) | 0.00  (0.00, 0.00) |
| Honduras | 0.60  (0.00, 1.29) | 11.47  (7.82, 15.12) | 0.00  (0.00, 0.00) | 0.00  (0.00, 0.00) | 0.00  (0.00, 0.00) | 0.00  (0.00, 0.00) | 29.45  (2.69, 56.21) | 0.00  (0.00, 0.00) | 0.02  (0.01, 0.03) |
| Hungary | 0.00  (0.00, 0.00) | 0.00  (0.00, 0.00) | 0.00  (0.00, 0.00) | 0.00  (0.00, 0.00) | 0.00  (0.00, 0.00) | 0.00  (0.00, 0.00) | 0.00  (0.00, 0.00) | 0.00  (0.00, 0.00) | 0.00  (0.00, 0.00) |
| Iceland | 0.00  (0.00, 0.00) | 0.01  (0.01, 0.02) | 0.00  (0.00, 0.00) | 0.00  (0.00, 0.00) | 0.00  (0.00, 0.00) | 0.00  (0.00, 0.00) | 0.00  (0.00, 0.00) | 0.00  (0.00, 0.00) | 0.00  (0.00, 0.00) |
| India | 91.00  (0.00, 253.97) | 0.00  (0.00, 0.00) | 47.40  (21.60, 73.20) | 0.00  (0.00, 0.00) | 47.40  (21.60, 73.20) | 0.00  (0.00, 0.00) | 62.77  (18.52, 107.02) | 0.00  (0.00, 0.00) | 0.00  (0.00, 0.00) |
| Indonesia | 28.46  (0.00, 83.40) | 0.00  (0.00, 0.00) | 35.76  (14.72, 56.81) | 0.00  (0.00, 0.00) | 35.76  (14.72, 56.81) | 0.00  (0.00, 0.00) | 279.79  (163.04, 396.54) | 0.00  (0.00, 0.00) | 0.00  (0.00, 0.00) |
| Iran  (Islamic Republic of) | 0.32  (0.20, 0.44) | 0.00  (0.00, 0.00) | 0.00  (0.00, 0.00) | 0.00  (0.00, 0.00) | 0.00  (0.00, 0.00) | 0.00  (0.00, 0.00) | 0.00  (0.00, 0.00) | 0.00  (0.00, 0.00) | 0.00  (0.00, 0.00) |
| Iraq | 0.00  (0.00, 0.00) | 0.00  (0.00, 0.00) | 0.00  (0.00, 0.00) | 0.00  (0.00, 0.00) | 0.00  (0.00, 0.00) | 0.00  (0.00, 0.00) | 0.00  (0.00, 0.00) | 0.00  (0.00, 0.00) | 0.00  (0.00, 0.00) |
| Ireland | 0.00  (0.00, 0.00) | 0.23  (0.13, 0.33) | 0.00  (0.00, 0.00) | 0.00  (0.00, 0.00) | 0.00  (0.00, 0.00) | 0.00  (0.00, 0.00) | 0.00  (0.00, 0.00) | 0.00  (0.00, 0.00) | 0.00  (0.00, 0.00) |
| Israel | 0.00  (0.00, 0.00) | 0.11  (0.06, 0.16) | 0.00  (0.00, 0.00) | 0.00  (0.00, 0.00) | 0.00  (0.00, 0.00) | 0.00  (0.00, 0.00) | 0.00  (0.00, 0.00) | 0.00  (0.00, 0.00) | 0.00  (0.00, 0.00) |
| Italy | 0.00  (0.00, 0.00) | 0.00  (0.00, 0.00) | 0.00  (0.00, 0.00) | 0.00  (0.00, 0.00) | 0.00  (0.00, 0.00) | 0.00  (0.00, 0.00) | 0.00  (0.00, 0.00) | 0.00  (0.00, 0.00) | 0.00  (0.00, 0.00) |
| Jamaica | 0.00  (0.00, 0.00) | 0.00  (0.00, 0.01) | 0.00  (0.00, 0.00) | 0.00  (0.00, 0.00) | 0.00  (0.00, 0.00) | 0.00  (0.00, 0.00) | 2.92  (0.25, 5.58) | 0.00  (0.00, 0.00) | 0.00  (0.00, 0.01) |
| Japan | 0.00  (0.00, 0.00) | 0.03  (0.02, 0.05) | 0.00  (0.00, 0.00) | 0.00  (0.00, 0.00) | 0.00  (0.00, 0.00) | 0.00  (0.00, 0.00) | 0.01  (0.00, 0.01) | 0.00  (0.00, 0.00) | 0.00  (0.00, 0.00) |
| Jordan | 0.00  (0.00, 0.00) | 0.00  (0.00, 0.00) | 0.00  (0.00, 0.00) | 0.00  (0.00, 0.00) | 0.00  (0.00, 0.00) | 0.00  (0.00, 0.00) | 0.18  (0.00, 0.48) | 0.00  (0.00, 0.00) | 0.00  (0.00, 0.00) |
| Kazakhstan | 0.00  (0.00, 0.00) | 0.00  (0.00, 0.00) | 0.00  (0.00, 0.00) | 0.00  (0.00, 0.00) | 0.00  (0.00, 0.00) | 0.00  (0.00, 0.00) | 0.00  (0.00, 0.00) | 0.00  (0.00, 0.00) | 0.00  (0.00, 0.00) |
| Kenya | 1071.56  (563.92, 1579.19) | 0.00  (0.00, 0.00) | 30.54  (12.38, 48.70) | 0.00  (0.00, 0.00) | 30.54  (12.38, 48.70) | 0.00  (0.00, 0.00) | 3.20  (0.00, 12.39) | 3.88  (0.27, 7.49) | 0.00  (0.00, 0.00) |
| Kiribati | 0.00  (0.00, 0.00) | 0.00  (0.00, 0.00) | 32.58  (13.08, 52.07) | 0.00  (0.00, 0.00) | 32.58  (13.08, 52.07) | 0.00  (0.00, 0.00) | 62.41  (0.00, 190.86) | 0.00  (0.00, 0.00) | 0.00  (0.00, 0.00) |
| Kuwait | 0.00  (0.00, 0.00) | 0.00  (0.00, 0.00) | 0.00  (0.00, 0.00) | 0.00  (0.00, 0.00) | 0.00  (0.00, 0.00) | 0.00  (0.00, 0.00) | 0.59  (0.00, 1.70) | 0.00  (0.00, 0.00) | 0.00  (0.00, 0.00) |
| Kyrgyzstan | 0.00  (0.00, 0.00) | 0.00  (0.00, 0.00) | 0.00  (0.00, 0.00) | 0.00  (0.00, 0.00) | 0.00  (0.00, 0.00) | 0.00  (0.00, 0.00) | 0.00  (0.00, 0.00) | 0.00  (0.00, 0.00) | 0.00  (0.00, 0.00) |
| Lao People's Democratic Republic | 8.25  (5.03, 11.47) | 0.00  (0.00, 0.00) | 25.84  (10.17, 41.51) | 0.00  (0.00, 0.00) | 25.84  (10.17, 41.51) | 0.00  (0.00, 0.00) | 14.39  (2.75, 26.03) | 0.00  (0.00, 0.00) | 0.00  (0.00, 0.00) |
| Latvia | 0.00  (0.00, 0.00) | 0.00  (0.00, 0.00) | 0.00  (0.00, 0.00) | 0.00  (0.00, 0.00) | 0.00  (0.00, 0.00) | 0.00  (0.00, 0.00) | 0.00  (0.00, 0.00) | 0.00  (0.00, 0.00) | 0.00  (0.00, 0.00) |
| Lebanon | 0.00  (0.00, 0.00) | 0.00  (0.00, 0.00) | 0.00  (0.00, 0.00) | 0.00  (0.00, 0.00) | 0.00  (0.00, 0.00) | 0.00  (0.00, 0.00) | 0.34  (0.00, 0.94) | 0.00  (0.00, 0.00) | 0.00  (0.00, 0.00) |
| Lesotho | 0.00  (0.00, 0.00) | 0.00  (0.00, 0.00) | 0.00  (0.00, 0.00) | 0.00  (0.00, 0.00) | 0.00  (0.00, 0.00) | 0.00  (0.00, 0.00) | 0.00  (0.00, 0.00) | 0.00  (0.00, 0.00) | 0.00  (0.00, 0.00) |
| Liberia | 6836.60  (1401.46, 12271.75) | 0.00  (0.00, 0.00) | 259.41  (166.65, 352.16) | 0.00  (0.00, 0.00) | 259.41  (166.65, 352.16) | 1301.99  (651.65, 1952.33) | 5.31  (0.00, 23.58) | 43.82  (0.00, 101.22) | 0.00  (0.00, 0.00) |
| Libya | 0.00  (0.00, 0.00) | 0.00  (0.00, 0.00) | 0.00  (0.00, 0.00) | 0.00  (0.00, 0.00) | 0.00  (0.00, 0.00) | 0.00  (0.00, 0.00) | 0.00  (0.00, 0.00) | 0.00  (0.00, 0.00) | 0.00  (0.00, 0.00) |
| Lithuania | 0.00  (0.00, 0.00) | 0.00  (0.00, 0.00) | 0.00  (0.00, 0.00) | 0.00  (0.00, 0.00) | 0.00  (0.00, 0.00) | 0.00  (0.00, 0.00) | 0.00  (0.00, 0.00) | 0.00  (0.00, 0.00) | 0.00  (0.00, 0.00) |
| Luxembourg | 0.00  (0.00, 0.00) | 0.02  (0.01, 0.03) | 0.00  (0.00, 0.00) | 0.00  (0.00, 0.00) | 0.00  (0.00, 0.00) | 0.00  (0.00, 0.00) | 0.00  (0.00, 0.00) | 0.00  (0.00, 0.00) | 0.00  (0.00, 0.00) |
| Madagascar | 1501.44  (89.90, 2912.99) | 0.00  (0.00, 0.00) | 29.46  (11.67, 47.25) | 0.00  (0.00, 0.00) | 29.46  (11.67, 47.25) | 0.00  (0.00, 0.00) | 0.37  (0.00, 1.82) | 0.00  (0.00, 0.00) | 0.00  (0.00, 0.00) |
| Malawi | 2871.58  (470.58, 5272.57) | 0.00  (0.00, 0.00) | 24.82  (10.27, 39.37) | 97.96  (0.00, 377.73) | 24.82  (10.27, 39.37) | 45.75  (19.10, 72.40) | 3.07  (0.00, 7.31) | 0.00  (0.00, 0.00) | 0.00  (0.00, 0.00) |
| Malaysia | 0.05  (0.03, 0.07) | 0.00  (0.00, 0.00) | 30.11  (11.75, 48.46) | 0.00  (0.00, 0.00) | 30.11  (11.75, 48.46) | 0.00  (0.00, 0.00) | 57.98  (22.41, 93.56) | 0.00  (0.00, 0.00) | 0.00  (0.00, 0.00) |
| Maldives | 0.00  (0.00, 0.00) | 0.00  (0.00, 0.00) | 26.69  (9.75, 43.63) | 0.00  (0.00, 0.00) | 26.69  (9.75, 43.63) | 0.00  (0.00, 0.00) | 86.34  (0.00, 187.40) | 0.00  (0.00, 0.00) | 0.00  (0.00, 0.00) |
| Mali | 5353.07  (1306.48, 9399.65) | 0.00  (0.00, 0.00) | 33.98  (13.53, 54.43) | 0.00  (0.00, 0.00) | 33.98  (13.53, 54.43) | 0.45  (0.11, 0.79) | 0.63  (0.00, 3.40) | 11.74  (0.00, 29.14) | 0.00  (0.00, 0.00) |
| Malta | 0.00  (0.00, 0.00) | 0.00  (0.00, 0.00) | 0.00  (0.00, 0.00) | 0.00  (0.00, 0.00) | 0.00  (0.00, 0.00) | 0.00  (0.00, 0.00) | 0.00  (0.00, 0.00) | 0.00  (0.00, 0.00) | 0.00  (0.00, 0.00) |
| Marshall Islands | 0.00  (0.00, 0.00) | 0.00  (0.00, 0.00) | 14.76  (9.17, 20.34) | 0.00  (0.00, 0.00) | 14.76  (9.17, 20.34) | 0.00  (0.00, 0.00) | 100.28  (0.00, 316.51) | 0.00  (0.00, 0.00) | 0.00  (0.00, 0.00) |
| Mauritania | 531.98  (23.09, 1040.87) | 0.00  (0.00, 0.00) | 0.00  (0.00, 0.00) | 0.00  (0.00, 0.00) | 0.00  (0.00, 0.00) | 0.00  (0.00, 0.00) | 0.42  (0.00, 1.92) | 14.74  (0.00, 37.36) | 0.00  (0.00, 0.00) |
| Mauritius | 0.00  (0.00, 0.00) | 0.00  (0.00, 0.00) | 0.00  (0.00, 0.00) | 0.00  (0.00, 0.00) | 0.00  (0.00, 0.00) | 0.00  (0.00, 0.00) | 9.83  (0.00, 31.19) | 0.00  (0.00, 0.00) | 0.00  (0.00, 0.00) |
| Mexico | 0.08  (0.07, 0.10) | 8.64  (5.45, 11.83) | 0.00  (0.00, 0.00) | 0.00  (0.00, 0.00) | 0.00  (0.00, 0.00) | 0.00  (0.00, 0.00) | 11.27  (6.30, 16.24) | 0.00  (0.00, 0.00) | 0.02  (0.00, 0.06) |
| Micronesia  (Federated States of) | 0.00  (0.00, 0.00) | 0.00  (0.00, 0.00) | 42.77  (17.00, 68.54) | 0.00  (0.00, 0.00) | 42.77  (17.00, 68.54) | 0.00  (0.00, 0.00) | 4.72  (0.00, 11.16) | 0.00  (0.00, 0.00) | 0.00  (0.00, 0.00) |
| Monaco | 0.00  (0.00, 0.00) | 0.00  (0.00, 0.00) | 0.00  (0.00, 0.00) | 0.00  (0.00, 0.00) | 0.00  (0.00, 0.00) | 0.00  (0.00, 0.00) | 0.00  (0.00, 0.00) | 0.00  (0.00, 0.00) | 0.00  (0.00, 0.00) |
| Mongolia | 0.00  (0.00, 0.00) | 0.00  (0.00, 0.00) | 0.00  (0.00, 0.00) | 0.00  (0.00, 0.00) | 0.00  (0.00, 0.00) | 0.00  (0.00, 0.00) | 0.00  (0.00, 0.00) | 0.00  (0.00, 0.00) | 0.00  (0.00, 0.00) |
| Montenegro | 0.00  (0.00, 0.00) | 0.00  (0.00, 0.00) | 0.00  (0.00, 0.00) | 0.00  (0.00, 0.00) | 0.00  (0.00, 0.00) | 0.00  (0.00, 0.00) | 0.00  (0.00, 0.00) | 0.00  (0.00, 0.00) | 0.00  (0.00, 0.00) |
| Morocco | 0.00  (0.00, 0.00) | 0.00  (0.00, 0.00) | 0.00  (0.00, 0.00) | 0.00  (0.00, 0.00) | 0.00  (0.00, 0.00) | 0.00  (0.00, 0.00) | 0.00  (0.00, 0.00) | 0.00  (0.00, 0.00) | 0.00  (0.00, 0.00) |
| Mozambique | 5637.63  (437.74, 10837.52) | 0.00  (0.00, 0.00) | 28.34  (11.82, 44.85) | 0.00  (0.00, 0.00) | 28.34  (11.82, 44.85) | 0.00  (0.00, 0.00) | 1.15  (0.00, 5.83) | 0.00  (0.00, 0.00) | 0.00  (0.00, 0.00) |
| Myanmar | 47.32  (7.92, 86.72) | 0.00  (0.00, 0.00) | 34.51  (14.00, 55.01) | 0.00  (0.00, 0.00) | 34.51  (14.00, 55.01) | 0.00  (0.00, 0.00) | 49.86  (13.77, 85.95) | 0.00  (0.00, 0.00) | 0.00  (0.00, 0.00) |
| Namibia | 97.75  (0.00, 302.45) | 0.00  (0.00, 0.00) | 0.00  (0.00, 0.00) | 0.00  (0.00, 0.00) | 0.00  (0.00, 0.00) | 0.00  (0.00, 0.00) | 0.00  (0.00, 0.00) | 0.00  (0.00, 0.00) | 0.00  (0.00, 0.00) |
| Nauru | 0.00  (0.00, 0.00) | 0.00  (0.00, 0.00) | 0.00  (0.00, 0.00) | 0.00  (0.00, 0.00) | 0.00  (0.00, 0.00) | 0.00  (0.00, 0.00) | 10.68  (0.00, 33.77) | 0.00  (0.00, 0.00) | 0.00  (0.00, 0.00) |
| Nepal | 2.12  (0.86, 3.37) | 0.00  (0.00, 0.00) | 43.84  (18.59, 69.09) | 0.00  (0.00, 0.00) | 43.84  (18.59, 69.09) | 0.00  (0.00, 0.00) | 52.12  (14.62, 89.61) | 0.00  (0.00, 0.00) | 0.00  (0.00, 0.00) |
| Netherlands | 0.00  (0.00, 0.00) | 0.03  (0.02, 0.05) | 0.00  (0.00, 0.00) | 0.00  (0.00, 0.00) | 0.00  (0.00, 0.00) | 0.00  (0.00, 0.00) | 0.00  (0.00, 0.00) | 0.00  (0.00, 0.00) | 0.00  (0.00, 0.00) |
| New Zealand | 0.00  (0.00, 0.00) | 0.00  (0.00, 0.00) | 0.00  (0.00, 0.00) | 0.00  (0.00, 0.00) | 0.00  (0.00, 0.00) | 0.00  (0.00, 0.00) | 0.00  (0.00, 0.00) | 0.00  (0.00, 0.00) | 0.00  (0.00, 0.00) |
| Nicaragua | 76.36  (11.57, 141.14) | 7.76  (5.23, 10.30) | 0.00  (0.00, 0.00) | 0.00  (0.00, 0.00) | 0.00  (0.00, 0.00) | 0.00  (0.00, 0.00) | 11.93  (0.00, 25.03) | 0.00  (0.00, 0.00) | 0.04  (0.01, 0.07) |
| Niger | 7344.92  (1933.76, 12756.08) | 0.00  (0.00, 0.00) | 33.73  (12.87, 54.60) | 0.00  (0.00, 0.00) | 33.73  (12.87, 54.60) | 0.22  (0.00, 0.55) | 0.38  (0.00, 1.93) | 55.59  (0.00, 143.88) | 0.00  (0.00, 0.00) |
| Nigeria | 6016.99  (906.29, 11127.70) | 0.00  (0.00, 0.00) | 33.50  (14.24, 52.75) | 0.00  (0.00, 0.00) | 33.50  (14.24, 52.75) | 118.97  (57.55, 180.38) | 7.17  (0.00, 21.07) | 33.89  (3.72, 64.07) | 0.00  (0.00, 0.00) |
| Niue | 0.00  (0.00, 0.00) | 0.00  (0.00, 0.00) | 33.45  (13.10, 53.80) | 0.00  (0.00, 0.00) | 33.45  (13.10, 53.80) | 0.00  (0.00, 0.00) | 1.61  (0.00, 3.55) | 0.00  (0.00, 0.00) | 0.00  (0.00, 0.00) |
| North Macedonia | 0.00  (0.00, 0.00) | 0.00  (0.00, 0.00) | 0.00  (0.00, 0.00) | 0.00  (0.00, 0.00) | 0.00  (0.00, 0.00) | 0.00  (0.00, 0.00) | 0.00  (0.00, 0.00) | 0.00  (0.00, 0.00) | 0.00  (0.00, 0.00) |
| Northern Mariana Islands | 0.00  (0.00, 0.00) | 0.00  (0.00, 0.00) | 0.00  (0.00, 0.00) | 0.00  (0.00, 0.00) | 0.00  (0.00, 0.00) | 0.00  (0.00, 0.00) | 8.02  (0.00, 20.11) | 0.00  (0.00, 0.00) | 0.00  (0.00, 0.00) |
| Norway | 0.00  (0.00, 0.00) | 0.00  (0.00, 0.00) | 0.00  (0.00, 0.00) | 0.00  (0.00, 0.00) | 0.00  (0.00, 0.00) | 0.00  (0.00, 0.00) | 0.00  (0.00, 0.00) | 0.00  (0.00, 0.00) | 0.00  (0.00, 0.00) |
| Oman | 1.38  (0.85, 1.91) | 0.00  (0.00, 0.00) | 0.00  (0.00, 0.00) | 0.00  (0.00, 0.00) | 0.00  (0.00, 0.00) | 0.00  (0.00, 0.00) | 13.84  (4.89, 22.78) | 0.00  (0.00, 0.00) | 0.00  (0.00, 0.00) |
| Pakistan | 239.57  (0.00, 564.56) | 0.00  (0.00, 0.00) | 0.00  (0.00, 0.00) | 0.00  (0.00, 0.00) | 0.00  (0.00, 0.00) | 0.00  (0.00, 0.00) | 29.82  (4.63, 55.01) | 0.00  (0.00, 0.00) | 0.00  (0.00, 0.00) |
| Palau | 0.00  (0.00, 0.00) | 0.00  (0.00, 0.00) | 14.64  (9.09, 20.18) | 0.00  (0.00, 0.00) | 14.64  (9.09, 20.18) | 0.00  (0.00, 0.00) | 34.75  (0.00, 130.26) | 0.00  (0.00, 0.00) | 0.00  (0.00, 0.00) |
| Palestine | 0.00  (0.00, 0.00) | 0.00  (0.00, 0.00) | 0.00  (0.00, 0.00) | 0.00  (0.00, 0.00) | 0.00  (0.00, 0.00) | 0.00  (0.00, 0.00) | 0.34  (0.00, 0.78) | 0.00  (0.00, 0.00) | 0.00  (0.00, 0.00) |
| Panama | 1.16  (0.00, 2.38) | 5.01  (3.07, 6.95) | 0.00  (0.00, 0.00) | 0.00  (0.00, 0.00) | 0.00  (0.00, 0.00) | 0.00  (0.00, 0.00) | 11.96  (0.00, 27.49) | 1.55  (0.00, 3.66) | 0.02  (0.00, 0.03) |
| Papua New Guinea | 1259.20  (357.80, 2160.60) | 0.00  (0.00, 0.00) | 187.50  (113.32, 261.68) | 0.00  (0.00, 0.00) | 187.50  (113.32, 261.68) | 0.00  (0.00, 0.00) | 0.76  (0.13, 1.40) | 0.00  (0.00, 0.00) | 0.00  (0.00, 0.00) |
| Paraguay | 0.00  (0.00, 0.00) | 20.48  (15.53, 25.44) | 0.00  (0.00, 0.00) | 0.00  (0.00, 0.00) | 0.00  (0.00, 0.00) | 0.00  (0.00, 0.00) | 35.62  (8.51, 62.73) | 3.92  (0.00, 9.10) | 0.00  (0.00, 0.00) |
| Peru | 4.99  (0.00, 13.82) | 7.63  (5.28, 9.98) | 0.00  (0.00, 0.00) | 0.00  (0.00, 0.00) | 0.00  (0.00, 0.00) | 0.00  (0.00, 0.00) | 6.05  (0.00, 13.17) | 7.28  (0.00, 17.35) | 0.08  (0.03, 0.14) |
| Philippines | 1.36  (0.00, 4.02) | 0.00  (0.00, 0.00) | 29.51  (12.01, 47.01) | 0.00  (0.00, 0.00) | 29.51  (12.01, 47.01) | 0.00  (0.00, 0.00) | 126.60  (92.09, 161.12) | 0.00  (0.00, 0.00) | 0.00  (0.00, 0.00) |
| Poland | 0.00  (0.00, 0.00) | 0.00  (0.00, 0.00) | 0.00  (0.00, 0.00) | 0.00  (0.00, 0.00) | 0.00  (0.00, 0.00) | 0.00  (0.00, 0.00) | 0.00  (0.00, 0.00) | 0.00  (0.00, 0.00) | 0.00  (0.00, 0.00) |
| Portugal | 0.00  (0.00, 0.00) | 0.18  (0.10, 0.25) | 0.00  (0.00, 0.00) | 0.00  (0.00, 0.00) | 0.00  (0.00, 0.00) | 0.00  (0.00, 0.00) | 0.00  (0.00, 0.00) | 0.00  (0.00, 0.00) | 0.00  (0.00, 0.00) |
| Puerto Rico | 0.00  (0.00, 0.00) | 0.20  (0.11, 0.29) | 0.00  (0.00, 0.00) | 0.00  (0.00, 0.00) | 0.00  (0.00, 0.00) | 0.00  (0.00, 0.00) | 17.01  (0.00, 54.02) | 0.00  (0.00, 0.00) | 0.01  (0.00, 0.01) |
| Qatar | 0.00  (0.00, 0.00) | 0.00  (0.00, 0.00) | 0.00  (0.00, 0.00) | 0.00  (0.00, 0.00) | 0.00  (0.00, 0.00) | 0.00  (0.00, 0.00) | 0.07  (0.01, 0.12) | 0.00  (0.00, 0.00) | 0.00  (0.00, 0.00) |
| Republic of Korea | 0.10  (0.08, 0.12) | 0.00  (0.00, 0.00) | 0.00  (0.00, 0.00) | 0.00  (0.00, 0.00) | 0.00  (0.00, 0.00) | 0.00  (0.00, 0.00) | 0.00  (0.00, 0.00) | 0.00  (0.00, 0.00) | 0.00  (0.00, 0.00) |
| Republic of Moldova | 0.00  (0.00, 0.00) | 0.00  (0.00, 0.00) | 0.00  (0.00, 0.00) | 0.00  (0.00, 0.00) | 0.00  (0.00, 0.00) | 0.00  (0.00, 0.00) | 0.00  (0.00, 0.00) | 0.00  (0.00, 0.00) | 0.00  (0.00, 0.00) |
| Romania | 0.00  (0.00, 0.00) | 0.00  (0.00, 0.00) | 0.00  (0.00, 0.00) | 0.00  (0.00, 0.00) | 0.00  (0.00, 0.00) | 0.00  (0.00, 0.00) | 0.00  (0.00, 0.00) | 0.00  (0.00, 0.00) | 0.00  (0.00, 0.00) |
| Russian Federation | 0.00  (0.00, 0.00) | 0.00  (0.00, 0.00) | 0.00  (0.00, 0.00) | 0.00  (0.00, 0.00) | 0.00  (0.00, 0.00) | 0.00  (0.00, 0.00) | 0.00  (0.00, 0.00) | 0.00  (0.00, 0.00) | 0.00  (0.00, 0.00) |
| Rwanda | 1455.25  (618.60, 2291.90) | 0.00  (0.00, 0.00) | 0.00  (0.00, 0.00) | 0.00  (0.00, 0.00) | 0.00  (0.00, 0.00) | 0.00  (0.00, 0.00) | 0.15  (0.00, 0.58) | 8.22  (0.00, 20.76) | 0.00  (0.00, 0.00) |
| Saint Kitts and Nevis | 0.00  (0.00, 0.00) | 0.00  (0.00, 0.00) | 0.00  (0.00, 0.00) | 0.00  (0.00, 0.00) | 0.00  (0.00, 0.00) | 0.00  (0.00, 0.00) | 0.81  (0.00, 3.62) | 0.00  (0.00, 0.00) | 0.03  (0.00, 0.09) |
| Saint Lucia | 0.00  (0.00, 0.00) | 0.01  (0.00, 0.02) | 0.00  (0.00, 0.00) | 0.00  (0.00, 0.00) | 0.00  (0.00, 0.00) | 0.00  (0.00, 0.00) | 4.24  (0.78, 7.70) | 0.00  (0.00, 0.00) | 0.00  (0.00, 0.00) |
| Saint Vincent and the Grenadines | 0.00  (0.00, 0.00) | 0.00  (0.00, 0.00) | 0.00  (0.00, 0.00) | 0.00  (0.00, 0.00) | 0.00  (0.00, 0.00) | 0.00  (0.00, 0.00) | 3.37  (0.00, 8.99) | 0.00  (0.00, 0.00) | 0.01  (0.00, 0.02) |
| Samoa | 0.00  (0.00, 0.00) | 0.00  (0.00, 0.00) | 135.65  (75.89, 195.41) | 0.00  (0.00, 0.00) | 135.65  (75.89, 195.41) | 0.00  (0.00, 0.00) | 5.83  (0.00, 14.01) | 0.00  (0.00, 0.00) | 0.00  (0.00, 0.00) |
| San Marino | 0.00  (0.00, 0.00) | 0.00  (0.00, 0.00) | 0.00  (0.00, 0.00) | 0.00  (0.00, 0.00) | 0.00  (0.00, 0.00) | 0.00  (0.00, 0.00) | 0.00  (0.00, 0.00) | 0.00  (0.00, 0.00) | 0.00  (0.00, 0.00) |
| Sao Tome and Principe | 292.22  (133.39, 451.04) | 0.00  (0.00, 0.00) | 53.70  (23.01, 84.40) | 0.00  (0.00, 0.00) | 53.70  (23.01, 84.40) | 0.00  (0.00, 0.00) | 7.75  (0.00, 36.71) | 16.29  (0.00, 39.90) | 0.00  (0.00, 0.00) |
| Saudi Arabia | 1.00  (0.62, 1.37) | 0.00  (0.00, 0.00) | 0.00  (0.00, 0.00) | 0.00  (0.00, 0.00) | 0.00  (0.00, 0.00) | 0.00  (0.00, 0.00) | 0.79  (0.22, 1.37) | 0.00  (0.00, 0.00) | 0.00  (0.00, 0.00) |
| Senegal | 1831.71  (877.61, 2785.81) | 0.00  (0.00, 0.00) | 26.14  (10.25, 42.03) | 0.00  (0.00, 0.00) | 26.14  (10.25, 42.03) | 0.39  (0.01, 0.77) | 0.73  (0.00, 2.04) | 12.39  (0.00, 30.28) | 0.00  (0.00, 0.00) |
| Serbia | 0.00  (0.00, 0.00) | 0.00  (0.00, 0.00) | 0.00  (0.00, 0.00) | 0.00  (0.00, 0.00) | 0.00  (0.00, 0.00) | 0.00  (0.00, 0.00) | 0.00  (0.00, 0.00) | 0.00  (0.00, 0.00) | 0.00  (0.00, 0.00) |
| Seychelles | 0.00  (0.00, 0.00) | 0.00  (0.00, 0.00) | 0.00  (0.00, 0.00) | 0.00  (0.00, 0.00) | 0.00  (0.00, 0.00) | 0.00  (0.00, 0.00) | 124.10  (0.00, 345.40) | 0.00  (0.00, 0.00) | 0.00  (0.00, 0.00) |
| Sierra Leone | 8940.31  (1775.95, 16104.67) | 0.00  (0.00, 0.00) | 75.53  (35.07, 115.99) | 0.00  (0.00, 0.00) | 75.53  (35.07, 115.99) | 404.67  (139.62, 669.72) | 5.02  (0.00, 25.99) | 43.57  (0.00, 102.03) | 0.00  (0.00, 0.00) |
| Singapore | 0.00  (0.00, 0.00) | 0.00  (0.00, 0.00) | 0.00  (0.00, 0.00) | 0.00  (0.00, 0.00) | 0.00  (0.00, 0.00) | 0.00  (0.00, 0.00) | 85.30  (0.00, 185.86) | 0.00  (0.00, 0.00) | 0.00  (0.00, 0.00) |
| Slovakia | 0.00  (0.00, 0.00) | 0.00  (0.00, 0.00) | 0.00  (0.00, 0.00) | 0.00  (0.00, 0.00) | 0.00  (0.00, 0.00) | 0.00  (0.00, 0.00) | 0.00  (0.00, 0.00) | 0.00  (0.00, 0.00) | 0.00  (0.00, 0.00) |
| Slovenia | 0.00  (0.00, 0.00) | 0.00  (0.00, 0.00) | 0.00  (0.00, 0.00) | 0.00  (0.00, 0.00) | 0.00  (0.00, 0.00) | 0.00  (0.00, 0.00) | 0.00  (0.00, 0.00) | 0.00  (0.00, 0.00) | 0.00  (0.00, 0.00) |
| Solomon Islands | 1432.10  (626.25, 2237.94) | 0.00  (0.00, 0.00) | 0.00  (0.00, 0.00) | 0.00  (0.00, 0.00) | 0.00  (0.00, 0.00) | 0.00  (0.00, 0.00) | 10.86  (0.00, 24.86) | 0.00  (0.00, 0.00) | 0.00  (0.00, 0.00) |
| Somalia | 696.36  (40.34, 1352.37) | 0.00  (0.00, 0.00) | 0.00  (0.00, 0.00) | 0.00  (0.00, 0.00) | 0.00  (0.00, 0.00) | 0.00  (0.00, 0.00) | 0.44  (0.00, 2.26) | 48.50  (0.00, 122.18) | 0.00  (0.00, 0.00) |
| South Africa | 6.79  (0.00, 18.03) | 0.00  (0.00, 0.00) | 0.00  (0.00, 0.00) | 0.00  (0.00, 0.00) | 0.00  (0.00, 0.00) | 0.00  (0.00, 0.00) | 0.00  (0.00, 0.00) | 0.00  (0.00, 0.00) | 0.00  (0.00, 0.00) |
| South Sudan | 4423.00  (1126.23, 7719.77) | 0.00  (0.00, 0.00) | 29.87  (11.87, 47.87) | 24.77  (9.87, 39.66) | 29.87  (11.87, 47.87) | 1387.88  (802.71, 1973.05) | 0.05  (0.00, 0.09) | 51.86  (0.00, 124.09) | 0.00  (0.00, 0.00) |
| Spain | 0.00  (0.00, 0.00) | 0.49  (0.28, 0.69) | 0.00  (0.00, 0.00) | 0.00  (0.00, 0.00) | 0.00  (0.00, 0.00) | 0.00  (0.00, 0.00) | 0.00  (0.00, 0.00) | 0.00  (0.00, 0.00) | 0.00  (0.00, 0.00) |
| Sri Lanka | 0.00  (0.00, 0.00) | 0.00  (0.00, 0.00) | 0.00  (0.00, 0.00) | 0.00  (0.00, 0.00) | 0.00  (0.00, 0.00) | 0.00  (0.00, 0.00) | 59.54  (22.30, 96.78) | 0.00  (0.00, 0.00) | 0.00  (0.00, 0.00) |
| Sudan | 595.33  (63.69, 1126.97) | 0.00  (0.00, 0.00) | 29.19  (11.39, 46.98) | 0.00  (0.00, 0.00) | 29.19  (11.39, 46.98) | 13.26  (7.96, 18.57) | 0.18  (0.00, 0.76) | 47.16  (0.00, 114.91) | 0.00  (0.00, 0.00) |
| Suriname | 2.87  (0.10, 5.63) | 0.11  (0.07, 0.16) | 0.00  (0.00, 0.00) | 0.00  (0.00, 0.00) | 0.00  (0.00, 0.00) | 0.00  (0.00, 0.00) | 9.23  (0.72, 17.74) | 8.89  (0.00, 21.46) | 0.00  (0.00, 0.01) |
| Sweden | 0.00  (0.00, 0.00) | 0.08  (0.05, 0.12) | 0.00  (0.00, 0.00) | 0.00  (0.00, 0.00) | 0.00  (0.00, 0.00) | 0.00  (0.00, 0.00) | 0.00  (0.00, 0.00) | 0.00  (0.00, 0.00) | 0.00  (0.00, 0.00) |
| Switzerland | 0.00  (0.00, 0.00) | 0.14  (0.08, 0.20) | 0.00  (0.00, 0.00) | 0.00  (0.00, 0.00) | 0.00  (0.00, 0.00) | 0.00  (0.00, 0.00) | 0.00  (0.00, 0.00) | 0.00  (0.00, 0.00) | 0.00  (0.00, 0.00) |
| Syrian Arab Republic | 0.00  (0.00, 0.00) | 0.00  (0.00, 0.00) | 0.00  (0.00, 0.00) | 0.00  (0.00, 0.00) | 0.00  (0.00, 0.00) | 0.00  (0.00, 0.00) | 0.05  (0.00, 0.12) | 0.00  (0.00, 0.00) | 0.00  (0.00, 0.00) |
| Tajikistan | 0.00  (0.00, 0.00) | 0.00  (0.00, 0.00) | 0.00  (0.00, 0.00) | 0.00  (0.00, 0.00) | 0.00  (0.00, 0.00) | 0.00  (0.00, 0.00) | 0.00  (0.00, 0.00) | 0.00  (0.00, 0.00) | 0.00  (0.00, 0.00) |
| Thailand | 0.56  (0.20, 0.93) | 0.00  (0.00, 0.00) | 0.00  (0.00, 0.00) | 0.00  (0.00, 0.00) | 0.00  (0.00, 0.00) | 0.00  (0.00, 0.00) | 20.79  (10.94, 30.64) | 0.00  (0.00, 0.00) | 0.00  (0.00, 0.00) |
| Timor-Leste | 0.00  (0.00, 0.00) | 0.00  (0.00, 0.00) | 225.37  (140.94, 309.80) | 0.00  (0.00, 0.00) | 225.37  (140.94, 309.80) | 0.00  (0.00, 0.00) | 19.96  (6.50, 33.43) | 0.00  (0.00, 0.00) | 0.00  (0.00, 0.00) |
| Togo | 4325.44  (1094.62, 7556.26) | 0.00  (0.00, 0.00) | 0.00  (0.00, 0.00) | 0.00  (0.00, 0.00) | 0.00  (0.00, 0.00) | 2.73  (0.69, 4.77) | 4.97  (0.00, 24.67) | 5.39  (0.00, 12.81) | 0.00  (0.00, 0.00) |
| Tokelau | 0.00  (0.00, 0.00) | 0.00  (0.00, 0.00) | 0.00  (0.00, 0.00) | 0.00  (0.00, 0.00) | 0.00  (0.00, 0.00) | 0.00  (0.00, 0.00) | 0.87  (0.00, 1.74) | 0.00  (0.00, 0.00) | 0.00  (0.00, 0.00) |
| Tonga | 0.00  (0.00, 0.00) | 0.00  (0.00, 0.00) | 25.31  (10.38, 40.24) | 0.00  (0.00, 0.00) | 25.31  (10.38, 40.24) | 0.00  (0.00, 0.00) | 168.65  (0.00, 443.21) | 0.00  (0.00, 0.00) | 0.00  (0.00, 0.00) |
| Trinidad and Tobago | 0.00  (0.00, 0.00) | 0.02  (0.01, 0.03) | 0.00  (0.00, 0.00) | 0.00  (0.00, 0.00) | 0.00  (0.00, 0.00) | 0.00  (0.00, 0.00) | 20.94  (4.52, 37.36) | 1.21  (0.00, 3.61) | 0.00  (0.00, 0.01) |
| Tunisia | 0.00  (0.00, 0.00) | 0.00  (0.00, 0.00) | 0.00  (0.00, 0.00) | 0.00  (0.00, 0.00) | 0.00  (0.00, 0.00) | 0.00  (0.00, 0.00) | 0.00  (0.00, 0.00) | 0.00  (0.00, 0.00) | 0.00  (0.00, 0.00) |
| Türkiye | 0.00  (0.00, 0.00) | 0.00  (0.00, 0.00) | 0.00  (0.00, 0.00) | 0.00  (0.00, 0.00) | 0.00  (0.00, 0.00) | 0.00  (0.00, 0.00) | 0.00  (0.00, 0.00) | 0.00  (0.00, 0.00) | 0.00  (0.00, 0.00) |
| Turkmenistan | 0.00  (0.00, 0.00) | 0.00  (0.00, 0.00) | 0.00  (0.00, 0.00) | 0.00  (0.00, 0.00) | 0.00  (0.00, 0.00) | 0.00  (0.00, 0.00) | 0.00  (0.00, 0.00) | 0.00  (0.00, 0.00) | 0.00  (0.00, 0.00) |
| Tuvalu | 0.00  (0.00, 0.00) | 0.00  (0.00, 0.00) | 0.00  (0.00, 0.00) | 0.00  (0.00, 0.00) | 0.00  (0.00, 0.00) | 0.00  (0.00, 0.00) | 4.81  (0.00, 15.30) | 0.00  (0.00, 0.00) | 0.00  (0.00, 0.00) |
| Uganda | 6008.63  (1498.65, 10518.60) | 0.00  (0.00, 0.00) | 23.05  (10.09, 36.00) | 0.17  (0.03, 0.31) | 23.05  (10.09, 36.00) | 79.76  (43.21, 116.31) | 0.51  (0.00, 2.21) | 12.59  (0.00, 31.67) | 0.00  (0.00, 0.00) |
| Ukraine | 0.00  (0.00, 0.00) | 0.00  (0.00, 0.00) | 0.00  (0.00, 0.00) | 0.00  (0.00, 0.00) | 0.00  (0.00, 0.00) | 0.00  (0.00, 0.00) | 0.00  (0.00, 0.00) | 0.00  (0.00, 0.00) | 0.00  (0.00, 0.00) |
| United Arab Emirates | 0.00  (0.00, 0.00) | 0.00  (0.00, 0.00) | 0.00  (0.00, 0.00) | 0.00  (0.00, 0.00) | 0.00  (0.00, 0.00) | 0.00  (0.00, 0.00) | 0.00  (0.00, 0.00) | 0.00  (0.00, 0.00) | 0.00  (0.00, 0.00) |
| United Kingdom | 0.00  (0.00, 0.00) | 0.00  (0.00, 0.01) | 0.00  (0.00, 0.00) | 0.00  (0.00, 0.00) | 0.00  (0.00, 0.00) | 0.00  (0.00, 0.00) | 0.00  (0.00, 0.00) | 0.00  (0.00, 0.00) | 0.00  (0.00, 0.00) |
| United Republic of Tanzania | 1623.13  (240.92, 3005.34) | 0.00  (0.00, 0.00) | 25.99  (10.70, 41.29) | 0.00  (0.00, 0.00) | 25.99  (10.70, 41.29) | 41.10  (22.13, 60.07) | 0.20  (0.00, 0.87) | 8.80  (0.00, 22.10) | 0.00  (0.00, 0.00) |
| United States of America | 0.00  (0.00, 0.00) | 0.00  (0.00, 0.00) | 0.00  (0.00, 0.00) | 0.00  (0.00, 0.00) | 0.00  (0.00, 0.00) | 0.00  (0.00, 0.00) | 1.92  (0.00, 7.82) | 0.00  (0.00, 0.00) | 0.00  (0.00, 0.01) |
| United States Virgin Islands | 0.00  (0.00, 0.00) | 0.57  (0.33, 0.82) | 0.00  (0.00, 0.00) | 0.00  (0.00, 0.00) | 0.00  (0.00, 0.00) | 0.00  (0.00, 0.00) | 0.02  (0.00, 0.03) | 0.00  (0.00, 0.00) | 0.00  (0.00, 0.00) |
| Uruguay | 0.00  (0.00, 0.00) | 5.50  (3.59, 7.41) | 0.00  (0.00, 0.00) | 0.00  (0.00, 0.00) | 0.00  (0.00, 0.00) | 0.00  (0.00, 0.00) | 0.00  (0.00, 0.00) | 0.00  (0.00, 0.00) | 0.00  (0.00, 0.00) |
| Uzbekistan | 0.00  (0.00, 0.00) | 0.00  (0.00, 0.00) | 0.00  (0.00, 0.00) | 0.00  (0.00, 0.00) | 0.00  (0.00, 0.00) | 0.00  (0.00, 0.00) | 0.00  (0.00, 0.00) | 0.00  (0.00, 0.00) | 0.00  (0.00, 0.00) |
| Vanuatu | 8.25  (5.78, 10.73) | 0.00  (0.00, 0.00) | 48.58  (20.64, 76.52) | 0.00  (0.00, 0.00) | 48.58  (20.64, 76.52) | 0.00  (0.00, 0.00) | 8.17  (0.00, 19.02) | 0.00  (0.00, 0.00) | 0.00  (0.00, 0.00) |
| Venezuela  (Bolivarian Republic of) | 86.74  (0.00, 191.14) | 81.21  (56.27, 106.16) | 0.00  (0.00, 0.00) | 0.00  (0.00, 0.00) | 0.00  (0.00, 0.00) | 1.63  (0.50, 2.77) | 25.01  (4.01, 46.02) | 1.22  (0.00, 2.91) | 0.00  (0.00, 0.01) |
| Viet Nam | 0.27  (0.16, 0.39) | 0.00  (0.00, 0.00) | 27.75  (11.04, 44.46) | 0.00  (0.00, 0.00) | 27.75  (11.04, 44.46) | 0.00  (0.00, 0.00) | 16.84  (2.58, 31.09) | 0.00  (0.00, 0.00) | 0.00  (0.00, 0.00) |
| Yemen | 1641.84  (0.00, 3423.57) | 0.00  (0.00, 0.00) | 24.83  (9.94, 39.72) | 0.00  (0.00, 0.00) | 24.83  (9.94, 39.72) | 0.03  (0.01, 0.05) | 0.26  (0.00, 0.94) | 0.00  (0.00, 0.00) | 0.00  (0.00, 0.00) |
| Zambia | 2216.20  (154.77, 4277.63) | 0.00  (0.00, 0.00) | 40.27  (16.70, 63.84) | 16.40  (0.00, 61.93) | 40.27  (16.70, 63.84) | 0.00  (0.00, 0.00) | 0.46  (0.00, 1.87) | 5.05  (0.00, 12.29) | 0.00  (0.00, 0.00) |
| Zimbabwe | 685.30  (144.67, 1225.93) | 0.00  (0.00, 0.00) | 27.70  (11.41, 43.99) | 0.00  (0.00, 0.00) | 27.70  (11.41, 43.99) | 0.00  (0.00, 0.00) | 0.08  (0.00, 0.33) | 0.00  (0.00, 0.00) | 0.00  (0.00, 0.00) |

ASDRs age-standardized DALYs rates

**Table I** ASDR AAPCs (1990–2021) for arthropod-borne diseases, protozoiasis, helminthiases, viral diseases and other than malaria in 204 countries and territories

| **Location** | **ABDs** | **Protozoiasis** | **Helminthiases** | **Viral diseases** | **Other than malaria** |
| --- | --- | --- | --- | --- | --- |
| Afghanistan | -1.53 (-3.15, 0.11) | -1.53 (-3.15, 0.11) | #N/A | 0.25 (0.07, 0.43) | -1.72 (-2.26, -1.17) |
| Albania | -4.41 (-5.37, -3.44) | -4.41 (-5.37, -3.44) | #N/A | #N/A | -4.41 (-5.37, -3.44) |
| Algeria | -0.64 (-0.99, -0.28) | -0.64 (-0.99, -0.28) | #N/A | #N/A | -0.41 (-0.78, -0.04) |
| American Samoa | -2.81 (-3.73, -1.88) | #N/A | -3.27 (-4.48, -2.04) | 0.50 (-0.48, 1.48) | -2.81 (-3.73, -1.88) |
| Andorra | 1.15 (0.85, 1.45) | 1.15 (0.85, 1.45) | #N/A | #N/A | 1.15 (0.85, 1.45) |
| Angola | -1.97 (-3.10, -0.83) | -1.72 (-2.86, -0.56) | -4.04 (-4.18, -3.91) | -6.00 (-6.97, -5.03) | -8.76 (-12.34, -5.03) |
| Antigua and Barbuda | -2.21 (-7.62, 3.53) | -1.29 (-1.71, -0.87) | #N/A | -2.23 (-7.70, 3.57) | -2.21 (-7.62, 3.53) |
| Argentina | -4.28 (-4.45, -4.10) | -4.29 (-4.50, -4.08) | #N/A | -4.19 (-4.52, -3.87) | -4.20 (-4.32, -4.08) |
| Armenia | -1.25 (-2.29, -0.20) | -1.25 (-2.29, -0.20) | #N/A | #N/A | -1.21 (-2.14, -0.27) |
| Australia | 1.51 (0.76, 2.27) | -3.09 (-3.74, -2.43) | #N/A | 2.49 (2.00, 2.98) | 1.51 (0.76, 2.27) |
| Austria | -2.45 (-3.42, -1.46) | -2.45 (-3.42, -1.46) | #N/A | #N/A | -2.45 (-3.42, -1.46) |
| Azerbaijan | -0.82 (-1.90, 0.28) | -0.82 (-1.90, 0.28) | #N/A | #N/A | -0.21 (-0.97, 0.56) |
| Bahamas | 0.45 (0.31, 0.58) | 3.25 (2.82, 3.68) | #N/A | 0.45 (0.31, 0.58) | 0.45 (0.31, 0.58) |
| Bahrain | -2.18 (-2.32, -2.04) | #N/A | #N/A | -2.18 (-2.32, -2.04) | -2.18 (-2.32, -2.04) |
| Bangladesh | -9.12 (-11.82, -6.33) | -11.29 (-14.87, -7.56) | -4.11 (-4.30, -3.92) | 2.62 (1.83, 3.42) | -6.81 (-7.60, -6.01) |
| Barbados | 1.19 (0.35, 2.03) | -5.65 (-5.93, -5.37) | #N/A | 1.19 (0.35, 2.03) | 1.19 (0.35, 2.03) |
| Belarus | #N/A | #N/A | #N/A | #N/A | #N/A |
| Belgium | -15.86 (-17.16, -14.55) | -15.86 (-17.16, -14.55) | #N/A | #N/A | -15.86 (-17.16, -14.55) |
| Belize | -4.55 (-7.57, -1.44) | -5.88 (-7.76, -3.95) | #N/A | 0.80 (-0.94, 2.58) | -1.48 (-2.03, -0.92) |
| Benin | -0.03 (-0.67, 0.61) | 0.55 (-0.07, 1.17) | -9.23 (-9.33, -9.13) | -5.19 (-5.77, -4.60) | -8.38 (-8.80, -7.96) |
| Bermuda | -0.78 (-7.02, 5.87) | -2.44 (-2.62, -2.27) | #N/A | #N/A | -0.78 (-7.02, 5.87) |
| Bhutan | -16.56 (-20.02, -12.96) | -18.72 (-23.34, -13.81) | #N/A | 2.20 (0.96, 3.45) | -6.84 (-8.34, -5.30) |
| Bolivia (Plurinational State of) | -4.12 (-4.38, -3.85) | -4.15 (-4.32, -3.98) | #N/A | -2.88 (-9.81, 4.59) | -3.83 (-4.00, -3.66) |
| Bosnia and Herzegovina | -8.17 (-9.01, -7.32) | -8.17 (-9.01, -7.32) | #N/A | #N/A | -8.17 (-9.01, -7.32) |
| Botswana | 0.99 (-8.34, 11.28) | 0.99 (-8.34, 11.28) | #N/A | #N/A | #N/A |
| Brazil | -3.73 (-4.49, -2.97) | -4.75 (-5.60, -3.89) | -6.50 (-6.91, -6.08) | 1.02 (0.46, 1.60) | -2.66 (-3.15, -2.17) |
| Brunei Darussalam | -0.70 (-0.99, -0.40) | #N/A | -0.80 (-0.97, -0.64) | -0.39 (-2.15, 1.40) | -0.70 (-0.99, -0.40) |
| Bulgaria | -11.43 (-11.92, -10.94) | -11.43 (-11.92, -10.94) | #N/A | #N/A | -11.43 (-11.92, -10.94) |
| Burkina Faso | -0.84 (-1.88, 0.20) | -0.50 (-1.26, 0.26) | -11.43 (-12.27, -10.57) | -4.33 (-5.86, -2.77) | -8.59 (-9.37, -7.80) |
| Burundi | -2.29 (-2.92, -1.66) | -2.26 (-2.91, -1.61) | -1.77 (-1.99, -1.54) | -4.20 (-4.63, -3.77) | -2.89 (-3.13, -2.65) |
| Cabo Verde | 1.00 (0.23, 1.77) | -0.17 (-0.76, 0.42) | #N/A | 1.36 (0.29, 2.44) | 1.36 (0.29, 2.44) |
| Cambodia | -10.03 (-13.05, -6.90) | -13.43 (-19.61, -6.78) | #N/A | -0.07 (-0.48, 0.35) | -2.85 (-5.03, -0.62) |
| Cameroon | -1.06 (-1.47, -0.65) | -0.64 (-1.11, -0.17) | -3.93 (-3.99, -3.88) | -8.52 (-10.72, -6.26) | -4.29 (-4.52, -4.06) |
| Canada | -1.67 (-1.74, -1.61) | -1.67 (-1.74, -1.61) | #N/A | #N/A | -1.67 (-1.74, -1.61) |
| Central African Republic | -0.82 (-2.10, 0.49) | -0.31 (-1.81, 1.21) | -3.72 (-3.94, -3.50) | -4.14 (-4.70, -3.58) | -5.42 (-6.55, -4.28) |
| Chad | -1.73 (-2.74, -0.71) | -1.55 (-2.67, -0.43) | -3.22 (-3.30, -3.15) | -4.54 (-5.51, -3.56) | -5.48 (-6.30, -4.66) |
| Chile | -2.84 (-2.96, -2.73) | -2.84 (-2.96, -2.73) | #N/A | #N/A | -2.84 (-2.96, -2.73) |
| China | -4.05 (-4.56, -3.53) | -6.31 (-7.23, -5.38) | #N/A | 0.86 (0.44, 1.29) | -2.65 (-2.99, -2.32) |
| Colombia | -3.82 (-4.47, -3.17) | -5.20 (-6.64, -3.74) | #N/A | 1.80 (0.95, 2.67) | 0.18 (-0.62, 0.97) |
| Comoros | -1.63 (-10.25, 7.81) | -3.01 (-14.05, 9.46) | -4.58 (-4.81, -4.34) | 0.16 (-0.16, 0.48) | -2.56 (-2.75, -2.36) |
| Congo | -3.38 (-4.29, -2.45) | -3.29 (-4.23, -2.34) | -3.82 (-3.97, -3.67) | -5.53 (-6.70, -4.36) | -10.11 (-14.01, -6.02) |
| Cook Islands | -23.32 (-27.41, -19.00) | #N/A | #N/A | -23.32 (-27.41, -19.00) | -23.32 (-27.41, -19.00) |
| Costa Rica | -0.22 (-0.71, 0.28) | -0.36 (-0.61, -0.11) | #N/A | -0.15 (-1.13, 0.84) | -0.18 (-0.60, 0.24) |
| Côte d'Ivoire | -1.70 (-2.62, -0.77) | -1.30 (-2.38, -0.20) | -7.64 (-8.06, -7.22) | -4.84 (-5.99, -3.67) | -8.13 (-9.19, -7.07) |
| Croatia | -3.63 (-4.40, -2.85) | -3.63 (-4.40, -2.85) | #N/A | #N/A | -3.63 (-4.40, -2.85) |
| Cuba | -1.77 (-3.07, -0.45) | -18.96 (-20.32, -17.58) | #N/A | -0.32 (-1.40, 0.76) | -1.77 (-3.07, -0.45) |
| Cyprus | -4.19 (-5.38, -2.98) | -4.19 (-5.38, -2.98) | #N/A | #N/A | -4.19 (-5.38, -2.98) |
| Czechia | #N/A | #N/A | #N/A | #N/A | #N/A |
| Democratic People's Republic of Korea | #N/A | #N/A | #N/A | #N/A | #N/A |
| Democratic Republic of the Congo | -2.85 (-3.09, -2.61) | #N/A | -2.16 (-2.32, -1.99) | -4.07 (-5.26, -2.86) | -3.57 (-4.16, -2.98) |
| Denmark | 0.60 (0.53, 0.67) | 0.60 (0.53, 0.67) | #N/A | #N/A | 0.60 (0.53, 0.67) |
| Djibouti | -1.79 (-5.95, 2.56) | -1.74 (-5.86, 2.56) | #N/A | -8.82 (-9.82, -7.81) | -8.35 (-9.22, -7.48) |
| Dominica | -0.28 (-5.05, 4.73) | 9.96 (8.82, 11.11) | #N/A | -0.29 (-5.07, 4.72) | -0.28 (-5.05, 4.73) |
| Dominican Republic | -3.97 (-5.74, -2.17) | -1.65 (-4.04, 0.81) | -4.38 (-5.16, -3.59) | 0.07 (-5.57, 6.04) | -3.61 (-5.46, -1.71) |
| Ecuador | -6.90 (-8.75, -5.00) | -7.34 (-9.65, -4.96) | #N/A | -1.36 (-2.44, -0.26) | -3.13 (-3.64, -2.62) |
| Egypt | -6.72 (-7.30, -6.13) | -5.00 (-5.10, -4.90) | #N/A | -3.09 (-3.88, -2.29) | -6.72 (-7.30, -6.13) |
| El Salvador | -2.05 (-2.61, -1.47) | -3.44 (-3.57, -3.31) | #N/A | 1.39 (-2.10, 5.00) | -1.89 (-2.46, -1.32) |
| Equatorial Guinea | -1.78 (-2.96, -0.59) | -1.38 (-2.64, -0.10) | -4.01 (-4.31, -3.71) | -10.95 (-11.64, -10.26) | -6.11 (-7.35, -4.85) |
| Eritrea | -3.71 (-5.78, -1.60) | -3.80 (-6.25, -1.30) | -0.89 (-1.11, -0.67) | -4.98 (-5.35, -4.61) | -7.23 (-7.98, -6.46) |
| Estonia | #N/A | #N/A | #N/A | #N/A | #N/A |
| Eswatini | -7.91 (-13.08, -2.43) | -7.91 (-13.08, -2.43) | #N/A | #N/A | #N/A |
| Ethiopia | -4.96 (-7.11, -2.76) | -5.05 (-7.58, -2.45) | -3.45 (-3.58, -3.31) | -6.18 (-6.45, -5.91) | -7.96 (-8.23, -7.69) |
| Fiji | -2.33 (-2.94, -1.72) | #N/A | -2.44 (-3.12, -1.76) | 0.44 (-0.78, 1.69) | -2.33 (-2.94, -1.72) |
| Finland | 4.45 (4.14, 4.75) | 4.45 (4.14, 4.75) | #N/A | #N/A | 4.45 (4.14, 4.75) |
| France | -5.61 (-8.32, -2.82) | -5.62 (-8.33, -2.83) | #N/A | 0.34 (-1.08, 1.78) | -4.86 (-5.05, -4.66) |
| Gabon | -2.62 (-4.30, -0.92) | -2.50 (-4.33, -0.64) | -2.88 (-3.42, -2.34) | -7.02 (-8.07, -5.96) | -6.19 (-7.50, -4.86) |
| Gambia | -3.40 (-10.22, 3.93) | -3.37 (-10.31, 4.11) | #N/A | -4.85 (-5.78, -3.91) | -4.04 (-5.01, -3.06) |
| Georgia | 1.20 (0.47, 1.94) | 1.20 (0.47, 1.94) | #N/A | #N/A | 1.20 (0.42, 1.98) |
| Germany | -9.10 (-26.21, 11.98) | -8.93 (-26.24, 12.45) | #N/A | #N/A | -6.70 (-12.53, -0.49) |
| Ghana | -2.51 (-3.30, -1.71) | -2.22 (-3.01, -1.41) | -9.51 (-9.67, -9.36) | -4.59 (-5.64, -3.53) | -8.40 (-9.26, -7.54) |
| Greece | -0.80 (-1.75, 0.15) | -0.80 (-1.75, 0.15) | #N/A | -2.19 (-2.53, -1.84) | -0.81 (-1.73, 0.12) |
| Greenland | #N/A | #N/A | #N/A | #N/A | #N/A |
| Grenada | 4.05 (1.44, 6.73) | 7.48 (7.16, 7.81) | #N/A | 4.01 (1.29, 6.79) | 4.05 (1.44, 6.73) |
| Guam | 0.29 (-2.35, 3.01) | #N/A | #N/A | 0.29 (-2.35, 3.01) | 0.29 (-2.35, 3.01) |
| Guatemala | -5.01 (-6.12, -3.90) | -6.16 (-7.29, -5.01) | #N/A | 7.54 (6.48, 8.61) | -3.33 (-3.81, -2.86) |
| Guinea | -0.65 (-1.23, -0.07) | -0.33 (-0.98, 0.33) | -7.74 (-7.96, -7.51) | -5.87 (-7.21, -4.51) | -4.82 (-5.47, -4.17) |
| Guinea-Bissau | -4.18 (-5.95, -2.39) | -3.93 (-5.21, -2.63) | -9.08 (-11.12, -7.00) | -5.27 (-5.77, -4.77) | -7.71 (-9.38, -6.00) |
| Guyana | -3.67 (-7.22, 0.01) | -4.39 (-10.73, 2.41) | -2.62 (-2.75, -2.48) | -3.99 (-4.48, -3.51) | -2.62 (-2.74, -2.51) |
| Haiti | -5.53 (-11.29, 0.60) | -6.89 (-14.86, 1.83) | -8.15 (-8.54, -7.75) | 1.17 (0.53, 1.82) | -7.75 (-8.15, -7.35) |
| Honduras | -1.49 (-4.14, 1.23) | -2.19 (-2.48, -1.91) | #N/A | 1.14 (-6.44, 9.34) | -0.60 (-2.80, 1.66) |
| Hungary | #N/A | #N/A | #N/A | #N/A | #N/A |
| Iceland | 11.20 (10.59, 11.82) | 11.20 (10.59, 11.82) | #N/A | #N/A | 11.20 (10.59, 11.82) |
| India | -5.58 (-6.47, -4.69) | -7.61 (-9.23, -5.97) | -5.23 (-5.70, -4.76) | 1.29 (1.11, 1.47) | -4.19 (-4.62, -3.76) |
| Indonesia | -2.42 (-2.98, -1.86) | -6.50 (-12.65, 0.08) | -6.39 (-6.77, -6.02) | -0.03 (-0.42, 0.35) | -1.91 (-2.24, -1.58) |
| Iran (Islamic Republic of) | -3.66 (-4.11, -3.20) | -3.66 (-4.11, -3.20) | #N/A | #N/A | -1.60 (-1.65, -1.55) |
| Iraq | -3.26 (-3.87, -2.65) | -3.26 (-3.87, -2.65) | #N/A | #N/A | -3.16 (-3.76, -2.56) |
| Ireland | 14.33 (13.69, 14.97) | 14.33 (13.69, 14.97) | #N/A | #N/A | 14.33 (13.69, 14.97) |
| Israel | -2.02 (-2.34, -1.69) | -2.02 (-2.34, -1.69) | #N/A | #N/A | -2.02 (-2.34, -1.69) |
| Italy | -2.05 (-3.02, -1.07) | -2.05 (-3.02, -1.07) | #N/A | #N/A | -2.05 (-3.02, -1.07) |
| Jamaica | -2.75 (-8.85, 3.75) | -21.87 (-23.97, -19.71) | #N/A | -1.14 (-12.35, 11.50) | -2.75 (-8.85, 3.75) |
| Japan | -0.56 (-0.94, -0.19) | 3.15 (2.66, 3.64) | #N/A | -4.55 (-5.11, -3.98) | -0.56 (-0.94, -0.19) |
| Jordan | -5.82 (-6.21, -5.42) | -5.98 (-6.50, -5.46) | #N/A | 0.68 (-0.79, 2.16) | -5.82 (-6.21, -5.42) |
| Kazakhstan | -1.10 (-1.39, -0.81) | -1.10 (-1.39, -0.81) | #N/A | #N/A | -1.10 (-1.39, -0.81) |
| Kenya | -3.51 (-6.63, -0.28) | -3.31 (-6.84, 0.35) | -6.80 (-7.19, -6.42) | -5.64 (-6.06, -5.21) | -5.97 (-6.66, -5.27) |
| Kiribati | -6.61 (-7.12, -6.10) | #N/A | -9.36 (-9.86, -8.87) | -0.14 (-0.44, 0.17) | -6.61 (-7.12, -6.10) |
| Kuwait | -2.35 (-2.92, -1.78) | -2.37 (-2.43, -2.32) | #N/A | -1.92 (-3.75, -0.06) | -2.35 (-2.92, -1.78) |
| Kyrgyzstan | -1.13 (-1.64, -0.62) | -1.13 (-1.64, -0.62) | #N/A | #N/A | -1.40 (-1.62, -1.19) |
| Lao People's Democratic Republic | -5.97 (-10.67, -1.03) | -11.22 (-13.10, -9.30) | -2.88 (-3.10, -2.66) | 0.69 (-1.67, 3.12) | -2.15 (-6.24, 2.11) |
| Latvia | #N/A | #N/A | #N/A | #N/A | #N/A |
| Lebanon | -6.40 (-6.82, -5.98) | -6.70 (-7.07, -6.33) | #N/A | 0.80 (-2.36, 4.06) | -6.40 (-6.82, -5.98) |
| Lesotho | #N/A | #N/A | #N/A | #N/A | #N/A |
| Liberia | -1.30 (-2.12, -0.48) | -1.21 (-2.14, -0.27) | -1.55 (-1.64, -1.46) | -6.73 (-8.07, -5.37) | -1.74 (-2.43, -1.04) |
| Libya | -0.57 (-0.94, -0.21) | -0.57 (-0.94, -0.21) | #N/A | #N/A | -0.57 (-0.94, -0.21) |
| Lithuania | #N/A | #N/A | #N/A | #N/A | #N/A |
| Luxembourg | -0.71 (-0.87, -0.55) | -0.71 (-0.87, -0.55) | #N/A | #N/A | -0.71 (-0.87, -0.55) |
| Madagascar | -2.11 (-3.32, -0.89) | -1.73 (-2.89, -0.56) | -7.45 (-8.36, -6.53) | -6.19 (-7.27, -5.09) | -7.43 (-8.37, -6.49) |
| Malawi | -4.38 (-5.58, -3.17) | -4.34 (-5.63, -3.03) | -6.36 (-6.81, -5.92) | -2.86 (-3.49, -2.22) | -5.57 (-7.90, -3.18) |
| Malaysia | -2.02 (-3.16, -0.86) | -18.40 (-23.66, -12.77) | -3.17 (-3.46, -2.87) | -0.82 (-2.08, 0.46) | -1.78 (-2.51, -1.04) |
| Maldives | -0.25 (-3.28, 2.86) | #N/A | -2.66 (-4.92, -0.35) | 1.18 (-1.39, 3.81) | -0.25 (-3.28, 2.86) |
| Mali | -2.36 (-3.63, -1.07) | -2.16 (-3.59, -0.72) | -9.55 (-10.43, -8.66) | -5.35 (-5.53, -5.17) | -8.57 (-9.33, -7.80) |
| Malta | -4.04 (-4.54, -3.54) | -4.04 (-4.54, -3.54) | #N/A | #N/A | -4.04 (-4.54, -3.54) |
| Marshall Islands | 0.04 (-0.17, 0.24) | #N/A | -2.23 (-2.32, -2.14) | 0.58 (0.32, 0.83) | 0.04 (-0.17, 0.24) |
| Mauritania | -0.25 (-5.27, 5.04) | 3.38 (-2.24, 9.32) | #N/A | -6.03 (-6.27, -5.78) | -5.50 (-5.67, -5.33) |
| Mauritius | 4.05 (3.67, 4.43) | #N/A | #N/A | 4.05 (3.67, 4.43) | 4.05 (3.67, 4.43) |
| Mexico | -1.68 (-2.17, -1.19) | -2.52 (-2.74, -2.30) | #N/A | 1.95 (1.04, 2.86) | -1.51 (-2.01, -1.01) |
| Micronesia (Federated States of) | -6.08 (-6.73, -5.43) | #N/A | -6.37 (-7.29, -5.44) | -0.28 (-0.72, 0.16) | -6.08 (-6.73, -5.43) |
| Monaco | 9.38 (7.35, 11.45) | 9.38 (7.35, 11.45) | #N/A | #N/A | 9.38 (7.35, 11.45) |
| Mongolia | #N/A | #N/A | #N/A | #N/A | #N/A |
| Montenegro | -0.83 (-1.78, 0.12) | -0.83 (-1.78, 0.12) | #N/A | #N/A | -0.83 (-1.78, 0.12) |
| Morocco | 1.59 (1.24, 1.93) | 1.59 (1.24, 1.93) | #N/A | #N/A | 1.87 (1.56, 2.19) |
| Mozambique | -2.72 (-3.88, -1.55) | -2.56 (-3.38, -1.74) | -10.81 (-11.73, -9.89) | -4.76 (-5.85, -3.66) | -10.75 (-11.63, -9.86) |
| Myanmar | -5.79 (-8.36, -3.16) | -7.18 (-10.70, -3.51) | -6.81 (-7.35, -6.26) | -0.38 (-0.72, -0.05) | -4.73 (-5.19, -4.28) |
| Namibia | -5.98 (-15.00, 3.99) | -5.98 (-15.00, 3.99) | #N/A | #N/A | -0.93 (-1.02, -0.85) |
| Nauru | 7.84 (6.58, 9.12) | #N/A | #N/A | 7.84 (6.58, 9.12) | 7.84 (6.58, 9.12) |
| Nepal | -5.69 (-6.37, -5.00) | -9.92 (-11.09, -8.74) | -6.13 (-6.28, -5.97) | 0.55 (0.30, 0.81) | -5.36 (-5.79, -4.92) |
| Netherlands | 1.32 (0.15, 2.50) | 1.32 (0.15, 2.50) | #N/A | #N/A | 1.32 (0.15, 2.50) |
| New Zealand | #N/A | #N/A | #N/A | #N/A | #N/A |
| Nicaragua | 1.14 (0.39, 1.89) | 0.93 (0.09, 1.78) | #N/A | 3.79 (0.78, 6.89) | 0.35 (-0.28, 0.99) |
| Niger | -1.34 (-1.83, -0.86) | -1.19 (-1.69, -0.69) | -8.07 (-8.34, -7.79) | -4.48 (-5.14, -3.81) | -5.46 (-6.03, -4.88) |
| Nigeria | -1.45 (-1.86, -1.04) | -1.24 (-1.69, -0.78) | -4.86 (-5.68, -4.04) | -6.93 (-7.33, -6.53) | -5.47 (-6.31, -4.62) |
| Niue | -3.41 (-3.62, -3.20) | #N/A | -3.54 (-3.81, -3.27) | 0.95 (0.85, 1.04) | -3.41 (-3.62, -3.20) |
| North Macedonia | -2.80 (-3.30, -2.30) | -2.80 (-3.30, -2.30) | #N/A | #N/A | -2.80 (-3.30, -2.30) |
| Northern Mariana Islands | 0.62 (-0.68, 1.94) | #N/A | #N/A | 0.62 (-0.68, 1.94) | 0.62 (-0.68, 1.94) |
| Norway | #N/A | #N/A | #N/A | #N/A | #N/A |
| Oman | -6.67 (-9.58, -3.66) | -12.95 (-19.64, -5.70) | #N/A | -1.72 (-2.21, -1.23) | -3.36 (-3.78, -2.94) |
| Pakistan | -2.52 (-6.06, 1.16) | -2.92 (-6.70, 1.01) | #N/A | 1.96 (1.69, 2.22) | -4.52 (-5.05, -4.00) |
| Palau | -0.11 (-0.47, 0.26) | #N/A | -0.29 (-0.31, -0.26) | 0.06 (-0.45, 0.57) | -0.11 (-0.47, 0.26) |
| Palestine | -4.84 (-5.12, -4.55) | -4.91 (-5.18, -4.63) | #N/A | -0.47 (-1.53, 0.60) | -4.84 (-5.12, -4.55) |
| Panama | -0.32 (-1.14, 0.52) | -0.41 (-1.22, 0.42) | #N/A | 0.20 (-1.63, 2.07) | -0.15 (-0.31, 0.01) |
| Papua New Guinea | -4.08 (-6.33, -1.78) | -3.87 (-6.75, -0.89) | -4.50 (-4.85, -4.15) | 0.18 (-0.11, 0.46) | -4.49 (-4.84, -4.14) |
| Paraguay | -1.66 (-2.48, -0.84) | -3.16 (-3.79, -2.51) | #N/A | 0.78 (-2.31, 3.97) | -1.59 (-2.39, -0.79) |
| Peru | -5.74 (-7.89, -3.53) | -6.57 (-7.38, -5.76) | #N/A | -4.84 (-5.84, -3.83) | -2.15 (-2.51, -1.79) |
| Philippines | -2.63 (-4.75, -0.46) | -10.00 (-19.56, 0.71) | -6.30 (-6.99, -5.61) | 2.21 (-0.52, 5.01) | -2.16 (-4.06, -0.23) |
| Poland | #N/A | #N/A | #N/A | #N/A | #N/A |
| Portugal | -5.25 (-5.98, -4.51) | -5.25 (-5.98, -4.51) | #N/A | #N/A | -4.86 (-5.35, -4.37) |
| Puerto Rico | -0.96 (-2.09, 0.18) | -12.27 (-17.49, -6.71) | #N/A | -0.22 (-1.07, 0.63) | -0.96 (-2.09, 0.18) |
| Qatar | -2.08 (-2.30, -1.87) | #N/A | #N/A | -2.08 (-2.30, -1.87) | -2.08 (-2.30, -1.87) |
| Republic of Korea | -11.59 (-12.12, -11.05) | -11.61 (-12.15, -11.07) | #N/A | -5.88 (-7.65, -4.08) | -5.88 (-7.65, -4.08) |
| Republic of Moldova | #N/A | #N/A | #N/A | #N/A | #N/A |
| Romania | -15.01 (-16.14, -13.86) | -15.01 (-16.14, -13.86) | #N/A | #N/A | -15.13 (-15.67, -14.58) |
| Russian Federation | #N/A | #N/A | #N/A | #N/A | #N/A |
| Rwanda | -5.00 (-7.62, -2.31) | -5.00 (-7.64, -2.28) | #N/A | -6.10 (-7.02, -5.16) | -6.10 (-7.02, -5.16) |
| Saint Kitts and Nevis | -2.43 (-11.65, 7.75) | #N/A | #N/A | -2.43 (-11.65, 7.75) | -2.43 (-11.65, 7.75) |
| Saint Lucia | 2.24 (1.52, 2.96) | -0.47 (-0.64, -0.30) | #N/A | 2.24 (1.52, 2.97) | 2.24 (1.52, 2.96) |
| Saint Vincent and the Grenadines | -0.67 (-2.83, 1.54) | -3.18 (-3.92, -2.43) | #N/A | -0.67 (-2.83, 1.54) | -0.67 (-2.83, 1.54) |
| Samoa | -2.97 (-3.99, -1.94) | #N/A | -3.10 (-4.11, -2.09) | 2.97 (2.51, 3.42) | -2.97 (-3.99, -1.94) |
| San Marino | #N/A | #N/A | #N/A | #N/A | #N/A |
| Sao Tome and Principe | -5.42 (-13.32, 3.20) | -5.93 (-14.72, 3.77) | -3.26 (-3.63, -2.89) | -5.26 (-5.94, -4.57) | -4.05 (-4.45, -3.66) |
| Saudi Arabia | -3.24 (-4.48, -1.98) | -3.33 (-4.56, -2.09) | #N/A | 2.74 (2.34, 3.14) | -2.42 (-2.49, -2.35) |
| Senegal | -2.74 (-6.34, 1.00) | -2.60 (-6.30, 1.24) | -5.33 (-5.81, -4.85) | -6.24 (-7.64, -4.81) | -4.85 (-6.21, -3.47) |
| Serbia | -11.94 (-12.89, -10.98) | -11.94 (-12.89, -10.98) | #N/A | #N/A | -11.94 (-12.89, -10.98) |
| Seychelles | 1.55 (1.40, 1.71) | #N/A | #N/A | 1.55 (1.40, 1.71) | 1.55 (1.40, 1.71) |
| Sierra Leone | -0.94 (-1.60, -0.27) | -0.76 (-1.39, -0.12) | -2.96 (-3.03, -2.88) | -4.01 (-5.26, -2.75) | -3.13 (-4.52, -1.71) |
| Singapore | 0.51 (-0.22, 1.25) | #N/A | #N/A | 0.51 (-0.23, 1.26) | 0.51 (-0.23, 1.26) |
| Slovakia | #N/A | #N/A | #N/A | #N/A | #N/A |
| Slovenia | -6.85 (-7.57, -6.12) | -6.85 (-7.57, -6.12) | #N/A | #N/A | -6.85 (-7.57, -6.12) |
| Solomon Islands | -4.29 (-6.40, -2.12) | -4.30 (-6.45, -2.10) | #N/A | 2.98 (2.01, 3.95) | 2.98 (2.01, 3.95) |
| Somalia | -6.95 (-8.37, -5.51) | -7.10 (-8.60, -5.57) | #N/A | -2.57 (-2.80, -2.33) | -11.23 (-11.91, -10.56) |
| South Africa | -5.42 (-8.04, -2.72) | -5.42 (-8.04, -2.72) | #N/A | #N/A | 0.21 (0.06, 0.36) |
| South Sudan | -1.57 (-2.30, -0.83) | -1.79 (-2.74, -0.83) | -0.36 (-0.43, -0.29) | -4.33 (-4.77, -3.89) | -3.96 (-6.48, -1.37) |
| Spain | -4.06 (-4.94, -3.18) | -4.06 (-4.94, -3.18) | #N/A | -4.96 (-5.36, -4.55) | -4.01 (-4.61, -3.41) |
| Sri Lanka | -7.72 (-17.65, 3.40) | -12.64 (-15.13, -10.07) | #N/A | -0.32 (-11.99, 12.90) | 1.86 (-0.06, 3.82) |
| Sudan | -3.58 (-4.02, -3.14) | -2.82 (-3.36, -2.28) | -3.57 (-3.78, -3.36) | -7.92 (-8.11, -7.74) | -5.87 (-6.15, -5.59) |
| Suriname | -5.51 (-10.72, 0.01) | -5.93 (-11.39, -0.13) | #N/A | -2.73 (-4.04, -1.40) | -1.09 (-1.38, -0.80) |
| Sweden | -1.88 (-1.93, -1.84) | -1.88 (-1.93, -1.84) | #N/A | #N/A | -1.88 (-1.93, -1.84) |
| Switzerland | 0.05 (-1.11, 1.21) | 0.05 (-1.11, 1.21) | #N/A | #N/A | 0.05 (-1.11, 1.21) |
| Syrian Arab Republic | 2.23 (1.95, 2.50) | 2.23 (2.00, 2.46) | #N/A | -0.32 (-3.76, 3.25) | 2.19 (2.09, 2.30) |
| Tajikistan | -9.18 (-12.63, -5.60) | -9.18 (-12.63, -5.60) | #N/A | #N/A | -3.34 (-3.56, -3.11) |
| Thailand | -7.74 (-8.55, -6.93) | -15.55 (-18.54, -12.45) | #N/A | -2.71 (-4.19, -1.21) | -5.98 (-7.02, -4.92) |
| Timor-Leste | -4.47 (-8.59, -0.15) | #N/A | -3.28 (-6.86, 0.45) | -2.06 (-7.99, 4.26) | -3.12 (-6.89, 0.80) |
| Togo | -1.45 (-2.02, -0.87) | -1.27 (-1.86, -0.68) | -14.61 (-15.22, -14.00) | -3.58 (-3.99, -3.18) | -10.21 (-11.33, -9.08) |
| Tokelau | 1.29 (0.33, 2.26) | #N/A | #N/A | 1.29 (0.33, 2.26) | 1.29 (0.33, 2.26) |
| Tonga | 2.20 (0.93, 3.47) | #N/A | -1.85 (-3.23, -0.45) | 4.34 (2.36, 6.37) | 2.20 (0.93, 3.47) |
| Trinidad and Tobago | 1.20 (0.15, 2.26) | -3.74 (-4.41, -3.06) | #N/A | 1.16 (0.12, 2.21) | 1.20 (0.15, 2.26) |
| Tunisia | 0.54 (0.35, 0.73) | 0.54 (0.35, 0.73) | #N/A | #N/A | 0.54 (0.35, 0.73) |
| Türkiye | -5.91 (-6.50, -5.31) | -5.91 (-6.50, -5.31) | #N/A | #N/A | -5.61 (-6.16, -5.06) |
| Turkmenistan | -2.42 (-2.55, -2.29) | -2.42 (-2.51, -2.34) | #N/A | #N/A | -2.44 (-2.51, -2.36) |
| Tuvalu | 4.41 (3.95, 4.87) | #N/A | #N/A | 4.41 (3.95, 4.87) | 4.41 (3.95, 4.87) |
| Uganda | -2.58 (-3.42, -1.73) | -2.50 (-3.41, -1.58) | -4.90 (-5.11, -4.69) | -6.44 (-7.28, -5.60) | -9.94 (-12.49, -7.31) |
| Ukraine | #N/A | #N/A | #N/A | #N/A | #N/A |
| United Arab Emirates | #N/A | #N/A | #N/A | #N/A | #N/A |
| United Kingdom | 3.92 (3.78, 4.06) | 3.92 (3.78, 4.06) | #N/A | #N/A | 3.92 (3.78, 4.06) |
| United Republic of Tanzania | -4.77 (-5.36, -4.17) | -4.58 (-5.20, -3.96) | -6.26 (-6.44, -6.09) | -6.02 (-6.48, -5.56) | -6.90 (-7.61, -6.17) |
| United States of America | -0.33 (-0.53, -0.12) | -0.42 (-0.60, -0.23) | #N/A | 5.75 (4.71, 6.80) | -0.33 (-0.53, -0.12) |
| United States Virgin Islands | -1.32 (-3.64, 1.05) | #N/A | #N/A | -1.32 (-3.64, 1.05) | -1.32 (-3.64, 1.05) |
| Uruguay | -3.44 (-3.56, -3.33) | -3.44 (-3.56, -3.33) | #N/A | #N/A | -3.44 (-3.56, -3.33) |
| Uzbekistan | -1.95 (-3.13, -0.76) | -1.95 (-3.13, -0.76) | #N/A | #N/A | -0.71 (-0.84, -0.58) |
| Vanuatu | -11.80 (-17.44, -5.78) | -16.38 (-23.87, -8.17) | -6.53 (-8.26, -4.76) | 3.75 (3.29, 4.21) | -6.15 (-7.97, -4.29) |
| Venezuela (Bolivarian Republic of) | -2.10 (-3.36, -0.83) | -1.97 (-3.66, -0.26) | -10.53 (-10.78, -10.29) | 1.15 (-0.87, 3.22) | -3.18 (-3.48, -2.88) |
| Viet Nam | -6.10 (-7.18, -5.00) | -16.15 (-19.35, -12.83) | -6.62 (-7.13, -6.10) | 2.06 (-0.32, 4.50) | -5.31 (-6.26, -4.34) |
| Yemen | -1.62 (-4.17, 1.00) | -1.65 (-4.25, 1.03) | -0.41 (-0.66, -0.17) | -2.76 (-4.21, -1.28) | -2.61 (-3.75, -1.45) |
| Zambia | -3.45 (-5.17, -1.70) | -3.31 (-5.19, -1.40) | -6.98 (-7.17, -6.79) | -6.41 (-6.83, -5.98) | -9.52 (-10.29, -8.74) |
| Zimbabwe | -0.72 (-8.23, 7.40) | -0.70 (-8.36, 7.60) | -0.80 (-1.15, -0.45) | -0.69 (-1.76, 0.39) | -1.08 (-1.65, -0.51) |

AAPCs average annual percentage changes

**Table J** ASDR AAPCs (1990–2021) for nine arthropod-borne diseases in 204 countries and territories

| **Location** | **Malaria** | **Chagas disease** | **Leishmaniasis** | **African**  **trypanosomiasis** | **Lymphatic**  **filariasis** | **Onchocerciasis** | **Dengue** | **Yellow**  **fever** | **Zika virus** |
| --- | --- | --- | --- | --- | --- | --- | --- | --- | --- |
| Afghanistan | -3.71 (-8.04, 0.83) | #N/A | -1.72  (-2.26, -1.17) | #N/A | #N/A | #N/A | 0.25  (0.07, 0.43) | #N/A | #N/A |
| Albania | #N/A | #N/A | -4.41  (-5.37, -3.44) | #N/A | #N/A | #N/A | #N/A | #N/A | #N/A |
| Algeria | #N/A | #N/A | -0.41  (-0.78, -0.04) | #N/A | #N/A | #N/A | #N/A | #N/A | #N/A |
| American Samoa | #N/A | #N/A | #N/A | #N/A | -3.27  (-4.48, -2.04) | #N/A | 0.51  (-0.45, 1.47) | #N/A | #N/A |
| Andorra | #N/A | 1.15 (0.85, 1.45) | #N/A | #N/A | #N/A | #N/A | #N/A | #N/A | #N/A |
| Angola | -0.17 (-1.16, 0.83) | #N/A | -11.11  (-13.27, -8.89) | -13.1  (-20.22, -5.35) | -5.52  (-5.8, -5.24) | -1.88  (-1.93, -1.83) | 6.38  (5.93, 6.84) | -6.09  (-7.08, -5.09) | #N/A |
| Antigua and Barbuda | #N/A | -1.29 (-1.71, -0.87) | #N/A | #N/A | #N/A | #N/A | -0.62  (-1.91, 0.68) | #N/A | #N/A |
| Argentina | #N/A | -4.23 (-4.4, -4.06) | -0.85  (-1.12, -0.58) | #N/A | #N/A | #N/A | 1.3  (1.12, 1.49) | -5.05  (-5.15, -4.95) | #N/A |
| Armenia | #N/A | #N/A | -1.21  (-2.14, -0.27) | #N/A | #N/A | #N/A | #N/A | #N/A | #N/A |
| Australia | #N/A | -3.06 (-3.14, -2.98) | #N/A | #N/A | #N/A | #N/A | 2.49  (2, 2.98) | #N/A | #N/A |
| Austria | #N/A | -1.67 (-1.76, -1.59) | -2.45  (-3.44, -1.46) | #N/A | #N/A | #N/A | #N/A | #N/A | #N/A |
| Azerbaijan | #N/A | #N/A | -0.21  (-0.97, 0.56) | #N/A | #N/A | #N/A | #N/A | #N/A | #N/A |
| Bahamas | #N/A | 3.25 (2.82, 3.68) | #N/A | #N/A | #N/A | #N/A | 0.52  (0.4, 0.63) | #N/A | #N/A |
| Bahrain | #N/A | #N/A | #N/A | #N/A | #N/A | #N/A | -2.18  (-2.32, -2.04) | #N/A | #N/A |
| Bangladesh | -12.61 (-17.11, -7.86) | #N/A | -13.35  (-14.13, -12.56) | #N/A | -4.11  (-4.3, -3.92) | #N/A | 2.62  (1.83, 3.42) | #N/A | #N/A |
| Barbados | #N/A | -5.65 (-5.93, -5.37) | #N/A | #N/A | #N/A | #N/A | 1.17  (0.42, 1.93) | #N/A | #N/A |
| Belarus | #N/A | #N/A | #N/A | #N/A | #N/A | #N/A | #N/A | #N/A | #N/A |
| Belgium | #N/A | -0.46 (-0.54, -0.39) | #N/A | #N/A | #N/A | #N/A | #N/A | #N/A | #N/A |
| Belize | #N/A | -7.67 (-8.15, -7.18) | -1.2  (-1.25, -1.16) | #N/A | #N/A | #N/A | 1.04  (-0.44, 2.55) | #N/A | #N/A |
| Benin | 0.55 (-0.06, 1.15) | #N/A | #N/A | #N/A | -1.91  (-2.15, -1.67) | -11.47  (-11.59, -11.35) | 0.16  (-0.33, 0.66) | -5.76  (-6.38, -5.14) | #N/A |
| Bermuda | #N/A | -2.44 (-2.62, -2.27) | #N/A | #N/A | #N/A | #N/A | #N/A | #N/A | #N/A |
| Bhutan | -21.52 (-24.86, -18.04) | #N/A | -9.03  (-10.98, -7.04) | #N/A | #N/A | #N/A | 2.2  (0.96, 3.45) | #N/A | #N/A |
| Bolivia (Plurinational State of) | -10.21 (-11.38, -9.02) | -4.25 (-4.36, -4.13) | -2.55  (-2.7, -2.4) | #N/A | #N/A | #N/A | 0.67  (-7.3, 9.33) | -6.73  (-7.89, -5.56) | #N/A |
| Bosnia and Herzegovina | #N/A | #N/A | -8.17  (-9.01, -7.32) | #N/A | #N/A | #N/A | #N/A | #N/A | #N/A |
| Botswana | 0.99 (-8.34, 11.28) | #N/A | #N/A | #N/A | #N/A | #N/A | #N/A | #N/A | #N/A |
| Brazil | -10.32 (-12.33, -8.27) | -4.96 (-5.13, -4.78) | -0.52  (-1.07, 0.03) | #N/A | -5.96  (-6.34, -5.57) | -14.36  (-14.75, -13.97) | 1.33  (0.9, 1.76) | -10.22  (-19.72, 0.39) | #N/A |
| Brunei Darussalam | #N/A | #N/A | #N/A | #N/A | -0.8  (-0.97, -0.64) | #N/A | -0.39  (-2.15, 1.4) | #N/A | #N/A |
| Bulgaria | #N/A | #N/A | -11.43  (-11.92, -10.94) | #N/A | #N/A | #N/A | #N/A | #N/A | #N/A |
| Burkina Faso | -0.5 (-1.26, 0.27) | #N/A | 1.66  (1.46, 1.86) | #N/A | -8.36  (-9.08, -7.62) | -22.16  (-23.29, -21.01) | 1.39  (0.52, 2.27) | -4.48  (-6.06, -2.87) | #N/A |
| Burundi | -2.26 (-2.91, -1.61) | #N/A | #N/A | #N/A | #N/A | -1.77  (-1.99, -1.54) | -8.77  (-9.9, -7.63) | -4.15  (-4.59, -3.72) | #N/A |
| Cabo Verde | -0.17 (-0.76, 0.42) | #N/A | #N/A | #N/A | #N/A | #N/A | 1.34  (0.27, 2.43) | #N/A | #N/A |
| Cambodia | -13.43 (-19.61, -6.78) | #N/A | #N/A | #N/A | #N/A | #N/A | -0.07  (-0.48, 0.35) | #N/A | #N/A |
| Cameroon | -0.63 (-1.11, -0.15) | #N/A | 1.96  (1.7, 2.23) | -12.02  (-21.4, -1.52) | -4.1  (-4.83, -3.36) | -3.95  (-4.03, -3.86) | 1.57  (1.37, 1.78) | -10.93  (-12.58, -9.24) | #N/A |
| Canada | #N/A | -1.67 (-1.74, -1.61) | #N/A | #N/A | #N/A | #N/A | #N/A | #N/A | #N/A |
| Central African Republic | 0.91 (0.48, 1.35) | #N/A | -7.38  (-9.3, -5.43) | -12.28  (-15.12, -9.35) | -5.44  (-5.71, -5.17) | -3.33  (-3.39, -3.27) | 0.3  (0.05, 0.55) | -4.54  (-5.15, -3.94) | #N/A |
| Chad | -1.28 (-1.81, -0.75) | #N/A | 5.42  (4.29, 6.57) | -20.15  (-24.11, -15.97) | -2.73  (-2.85, -2.61) | -3.34  (-3.49, -3.18) | 1.26  (1.05, 1.47) | -4.57  (-5.54, -3.59) | #N/A |
| Chile | #N/A | -2.84 (-2.96, -2.73) | #N/A | #N/A | #N/A | #N/A | #N/A | #N/A | #N/A |
| China | #N/A | #N/A | -5.98  (-6.5, -5.46) | #N/A | #N/A | #N/A | -5.84  (-7.1, -4.56) | #N/A | #N/A |
| Colombia | -6.95 (-11.5, -2.17) | -0.81 (-1.22, -0.4) | -0.36  (-0.5, -0.22) | #N/A | #N/A | #N/A | 2.17  (1.64, 2.7) | -5.47  (-7.3, -3.6) | #N/A |
| Comoros | -3.01 (-14.05, 9.46) | #N/A | #N/A | #N/A | -4.58  (-4.81, -4.34) | #N/A | 0.16  (-0.16, 0.48) | #N/A | #N/A |
| Congo | -1.35 (-1.92, -0.77) | #N/A | #N/A | -11.8  (-15.53, -7.9) | -3.49  (-3.99, -2.99) | -4.07  (-4.12, -4.01) | -0.68  (-1.01, -0.35) | -5.69  (-6.91, -4.46) | #N/A |
| Cook Islands | #N/A | #N/A | #N/A | #N/A | #N/A | #N/A | -22.49  (-24.83, -20.07) | #N/A | #N/A |
| Costa Rica | #N/A | -3.32 (-3.44, -3.2) | 0.62  (0.56, 0.67) | #N/A | #N/A | #N/A | -0.16  (-1.03, 0.72) | #N/A | #N/A |
| Côte d'Ivoire | -1.16 (-2.22, -0.08) | #N/A | 1.01  (0.82, 1.21) | -21.27  (-24.23, -18.19) | -4.54  (-5, -4.08) | -23.11  (-24.59, -21.6) | 1.65  (0.72, 2.58) | -5.51  (-6.75, -4.25) | #N/A |
| Croatia | #N/A | #N/A | -3.63  (-4.4, -2.85) | #N/A | #N/A | #N/A | #N/A | #N/A | #N/A |
| Cuba | #N/A | -18.96 (-20.32, -17.58) | #N/A | #N/A | #N/A | #N/A | -0.18  (-0.46, 0.11) | #N/A | #N/A |
| Cyprus | #N/A | #N/A | -4.19  (-5.38, -2.98) | #N/A | #N/A | #N/A | #N/A | #N/A | #N/A |
| Czechia | #N/A | #N/A | #N/A | #N/A | #N/A | #N/A | #N/A | #N/A | #N/A |
| Democratic People's  Republic of Korea | #N/A | #N/A | #N/A | #N/A | #N/A | #N/A | #N/A | #N/A | #N/A |
| Democratic Republic of the Congo | -2.71 (-2.88, -2.55) | #N/A | -8.4  (-10.34, -6.43) | -14.21  (-16.17, -12.21) | -6.44  (-6.73, -6.15) | -1.67  (-1.8, -1.54) | 0.45  (0.14, 0.75) | -4.3  (-5.58, -3) | #N/A |
| Denmark | #N/A | 0.6 (0.53, 0.67) | #N/A | #N/A | #N/A | #N/A | #N/A | #N/A | #N/A |
| Djibouti | -3.37 (-24.26, 23.28) | #N/A | -8.27  (-9.65, -6.88) | #N/A | #N/A | #N/A | -8.82  (-9.82, -7.81) | #N/A | #N/A |
| Dominica | #N/A | 9.96 (8.82, 11.11) | #N/A | #N/A | #N/A | #N/A | 0.66  (-2.82, 4.25) | #N/A | #N/A |
| Dominican Republic | -1.44 (-9.75, 7.63) | -4.21 (-4.29, -4.12) | -0.93  (-0.97, -0.9) | #N/A | -4.38  (-5.16, -3.59) | #N/A | 0.09  (-5.7, 6.23) | #N/A | #N/A |
| Ecuador | -18.79 (-30.79, -4.71) | -4.54 (-4.73, -4.35) | -0.92  (-1.01, -0.83) | #N/A | #N/A | #N/A | 2.09  (1.32, 2.86) | -6.52  (-7.63, -5.4) | #N/A |
| Egypt | #N/A | #N/A | -5  (-5.1, -4.9) | #N/A | #N/A | #N/A | -3.09  (-3.88, -2.29) | #N/A | #N/A |
| El Salvador | #N/A | -2.85 (-3.2, -2.5) | -3.44  (-3.53, -3.36) | #N/A | #N/A | #N/A | 1.25  (-2.33, 4.97) | #N/A | #N/A |
| Equatorial Guinea | -1.16 (-2.34, 0.04) | #N/A | #N/A | -8.86  (-18.93, 2.47) | -6.95  (-7.57, -6.32) | -3.09  (-3.27, -2.9) | 7.74  (4.96, 10.58) | -11.28  (-11.97, -10.6) | #N/A |
| Eritrea | -2.76 (-6.88, 1.55) | #N/A | -8.75  (-9.38, -8.12) | #N/A | -0.89  (-1.11, -0.67) | #N/A | -8.25  (-9.61, -6.87) | -4.9  (-5.31, -4.49) | #N/A |
| Estonia | #N/A | #N/A | #N/A | #N/A | #N/A | #N/A | #N/A | #N/A | #N/A |
| Eswatini | -7.91 (-13.08, -2.43) | #N/A | #N/A | #N/A | #N/A | #N/A | #N/A | #N/A | #N/A |
| Ethiopia | 0.58 (-3.04, 4.35) | #N/A | -11.77  (-12.96, -10.56) | #N/A | -4.5  (-4.71, -4.3) | -2.61  (-2.8, -2.42) | -4.52  (-6.89, -2.09) | -6.2  (-6.49, -5.91) | #N/A |
| Fiji | #N/A | #N/A | #N/A | #N/A | -2.44  (-3.12, -1.76) | #N/A | 0.44  (-0.79, 1.69) | #N/A | #N/A |
| Finland | #N/A | 4.45 (4.14, 4.75) | #N/A | #N/A | #N/A | #N/A | #N/A | #N/A | #N/A |
| France | #N/A | -1.58 (-1.65, -1.51) | -5.1  (-5.3, -4.9) | #N/A | #N/A | #N/A | 0.34  (-1.08, 1.78) | #N/A | #N/A |
| Gabon | -1.9 (-2.44, -1.36) | #N/A | #N/A | -6.18  (-7.94, -4.38) | -2.88  (-3.42, -2.34) | #N/A | 0.28  (0.01, 0.54) | -7.39  (-8.49, -6.27) | #N/A |
| Gambia | -3.38 (-10.33, 4.1) | #N/A | 4.4  (3.67, 5.14) | #N/A | #N/A | #N/A | 1.77  (1.29, 2.26) | -5.37  (-6.36, -4.36) | #N/A |
| Georgia | #N/A | #N/A | 1.2  (0.42, 1.98) | #N/A | #N/A | #N/A | #N/A | #N/A | #N/A |
| Germany | #N/A | -0.97 (-1.06, -0.88) | #N/A | #N/A | #N/A | #N/A | #N/A | #N/A | #N/A |
| Ghana | -2.22 (-3.01, -1.42) | #N/A | -0.68  (-0.77, -0.59) | #N/A | -5.84  (-6.28, -5.4) | -18.24  (-18.69, -17.78) | 1.66  (1.16, 2.17) | -6.18  (-7.32, -5.03) | #N/A |
| Greece | #N/A | -3.29 (-3.58, -3.01) | -0.81  (-1.72, 0.12) | #N/A | #N/A | #N/A | -2.19  (-2.53, -1.84) | #N/A | #N/A |
| Greenland | #N/A | #N/A | #N/A | #N/A | #N/A | #N/A | #N/A | #N/A | #N/A |
| Grenada | #N/A | 7.48 (7.16, 7.81) | #N/A | #N/A | #N/A | #N/A | 4.8  (4.09, 5.5) | #N/A | #N/A |
| Guam | #N/A | #N/A | #N/A | #N/A | #N/A | #N/A | 0.29  (-2.35, 3.01) | #N/A | #N/A |
| Guatemala | -26.14 (-33.56, -17.89) | -4 (-4.22, -3.78) | -2.58  (-2.64, -2.52) | #N/A | #N/A | #N/A | 7.36  (6.49, 8.23) | #N/A | #N/A |
| Guinea | -0.26 (-0.82, 0.31) | #N/A | 0.64  (0.54, 0.75) | -1.52  (-3.03, 0.01) | -4.97  (-5.25, -4.7) | -9.86  (-9.98, -9.74) | 1.13  (0.1, 2.16) | -5.93  (-7.29, -4.56) | #N/A |
| Guinea-Bissau | -3.93 (-5.21, -2.63) | #N/A | 0.77  (0.61, 0.93) | #N/A | -7.76  (-9.57, -5.91) | -23.06  (-24.21, -21.91) | 1.39  (0.55, 2.23) | -5.63  (-6.17, -5.1) | #N/A |
| Guyana | -4.46 (-11.05, 2.61) | -7.37 (-8.28, -6.46) | -1.07  (-1.12, -1.03) | #N/A | -2.62  (-2.75, -2.48) | #N/A | 1.5  (0.82, 2.18) | -5.66  (-6.17, -5.15) | #N/A |
| Haiti | -6.89 (-14.86, 1.83) | #N/A | #N/A | #N/A | -8.15  (-8.54, -7.75) | #N/A | 1.11  (0.51, 1.71) | #N/A | #N/A |
| Honduras | -9.59 (-17.74, -0.63) | -2.52 (-2.59, -2.44) | -0.72  (-0.77, -0.67) | #N/A | #N/A | #N/A | 1.14  (-6.54, 9.45) | #N/A | #N/A |
| Hungary | #N/A | #N/A | #N/A | #N/A | #N/A | #N/A | #N/A | #N/A | #N/A |
| Iceland | #N/A | 11.2 (10.59, 11.82) | #N/A | #N/A | #N/A | #N/A | #N/A | #N/A | #N/A |
| India | -7.38 (-9.27, -5.44) | #N/A | -10.31  (-12.08, -8.5) | #N/A | -5.23  (-5.7, -4.76) | #N/A | 1.29  (1.11, 1.47) | #N/A | #N/A |
| Indonesia | -6.5 (-12.65, 0.08) | #N/A | #N/A | #N/A | -6.39  (-6.77, -6.02) | #N/A | -0.03  (-0.42, 0.35) | #N/A | #N/A |
| Iran  (Islamic Republic of) | -16.97 (-22.13, -11.46) | #N/A | -1.6  (-1.65, -1.55) | #N/A | #N/A | #N/A | #N/A | #N/A | #N/A |
| Iraq | #N/A | #N/A | -3.16  (-3.76, -2.56) | #N/A | #N/A | #N/A | #N/A | #N/A | #N/A |
| Ireland | #N/A | 14.33 (13.69, 14.97) | #N/A | #N/A | #N/A | #N/A | #N/A | #N/A | #N/A |
| Israel | #N/A | -4.17 (-4.35, -3.99) | -1.44  (-1.83, -1.05) | #N/A | #N/A | #N/A | #N/A | #N/A | #N/A |
| Italy | #N/A | #N/A | -2.05  (-3.02, -1.07) | #N/A | #N/A | #N/A | #N/A | #N/A | #N/A |
| Jamaica | #N/A | -21.87 (-23.97, -19.71) | #N/A | #N/A | #N/A | #N/A | 1.87  (-0.99, 4.81) | #N/A | #N/A |
| Japan | #N/A | 3.15 (2.66, 3.64) | #N/A | #N/A | #N/A | #N/A | -4.55  (-5.11, -3.98) | #N/A | #N/A |
| Jordan | #N/A | #N/A | -5.98  (-6.5, -5.46) | #N/A | #N/A | #N/A | 0.68  (-0.79, 2.16) | #N/A | #N/A |
| Kazakhstan | #N/A | #N/A | -1.1  (-1.39, -0.81) | #N/A | #N/A | #N/A | #N/A | #N/A | #N/A |
| Kenya | -3.33 (-7.09, 0.59) | #N/A | -2.97  (-4.2, -1.73) | #N/A | -6.8  (-7.19, -6.42) | #N/A | -5.72  (-6.88, -4.54) | -5.53  (-5.89, -5.16) | #N/A |
| Kiribati | #N/A | #N/A | #N/A | #N/A | -9.36  (-9.86, -8.87) | #N/A | -0.14  (-0.44, 0.17) | #N/A | #N/A |
| Kuwait | #N/A | #N/A | -2.37  (-2.43, -2.32) | #N/A | #N/A | #N/A | -1.92  (-3.75, -0.06) | #N/A | #N/A |
| Kyrgyzstan | #N/A | #N/A | -1.4  (-1.62, -1.19) | #N/A | #N/A | #N/A | #N/A | #N/A | #N/A |
| Lao People's Democratic Republic | -11.22 (-13.1, -9.3) | #N/A | #N/A | #N/A | -2.88  (-3.1, -2.66) | #N/A | 0.69  (-1.67, 3.12) | #N/A | #N/A |
| Latvia | #N/A | #N/A | #N/A | #N/A | #N/A | #N/A | #N/A | #N/A | #N/A |
| Lebanon | #N/A | #N/A | -6.7  (-7.07, -6.33) | #N/A | #N/A | #N/A | 0.8  (-2.36, 4.06) | #N/A | #N/A |
| Lesotho | #N/A | #N/A | #N/A | #N/A | #N/A | #N/A | #N/A | #N/A | #N/A |
| Liberia | -1.21 (-2.14, -0.27) | #N/A | #N/A | #N/A | -1.65  (-1.93, -1.38) | -1.51  (-1.56, -1.47) | 0.82  (-0.03, 1.67) | -7.06  (-8.46, -5.63) | #N/A |
| Libya | #N/A | #N/A | -0.57  (-0.94, -0.21) | #N/A | #N/A | #N/A | #N/A | #N/A | #N/A |
| Lithuania | #N/A | #N/A | #N/A | #N/A | #N/A | #N/A | #N/A | #N/A | #N/A |
| Luxembourg | #N/A | -0.71 (-0.87, -0.55) | #N/A | #N/A | #N/A | #N/A | #N/A | #N/A | #N/A |
| Madagascar | -1.73 (-2.89, -0.56) | #N/A | #N/A | #N/A | -7.45  (-8.36, -6.53) | #N/A | -6.19  (-7.27, -5.09) | #N/A | #N/A |
| Malawi | -4.35 (-5.76, -2.92) | #N/A | -0.98  (-1.06, -0.91) | -5.27  (-11.8, 1.73) | -8.63  (-9.34, -7.92) | -3.55  (-3.61, -3.5) | -2.86  (-3.49, -2.22) | #N/A | #N/A |
| Malaysia | -18.4 (-23.66, -12.77) | #N/A | #N/A | #N/A | -3.17  (-3.46, -2.87) | #N/A | -0.82  (-2.08, 0.46) | #N/A | #N/A |
| Maldives | #N/A | #N/A | #N/A | #N/A | -2.66  (-4.92, -0.35) | #N/A | 1.18  (-1.39, 3.81) | #N/A | #N/A |
| Mali | -2.15 (-3.56, -0.73) | #N/A | 0.12  (0.02, 0.23) | #N/A | -8.71  (-9.42, -7.99) | -17.72  (-18.43, -16.99) | 0.75  (0.66, 0.85) | -5.49  (-5.68, -5.3) | #N/A |
| Malta | #N/A | #N/A | -4.04  (-4.54, -3.54) | #N/A | #N/A | #N/A | #N/A | #N/A | #N/A |
| Marshall Islands | #N/A | #N/A | #N/A | #N/A | -2.23  (-2.32, -2.14) | #N/A | 0.58  (0.32, 0.83) | #N/A | #N/A |
| Mauritania | 3.38 (-2.24, 9.33) | #N/A | 2.69  (2.13, 3.24) | #N/A | #N/A | #N/A | 1.7  (0.71, 2.71) | -6.11  (-6.36, -5.87) | #N/A |
| Mauritius | #N/A | #N/A | #N/A | #N/A | #N/A | #N/A | 4.05  (3.67, 4.43) | #N/A | #N/A |
| Mexico | -10.03 (-11.55, -8.48) | -2.2 (-2.45, -1.95) | -1.9  (-2.05, -1.75) | #N/A | #N/A | #N/A | 1.94  (1.04, 2.85) | #N/A | #N/A |
| Micronesia  (Federated States of) | #N/A | #N/A | #N/A | #N/A | -6.37  (-7.29, -5.44) | #N/A | -0.29  (-0.74, 0.17) | #N/A | #N/A |
| Monaco | #N/A | #N/A | 9.38  (7.35, 11.45) | #N/A | #N/A | #N/A | #N/A | #N/A | #N/A |
| Mongolia | #N/A | #N/A | #N/A | #N/A | #N/A | #N/A | #N/A | #N/A | #N/A |
| Montenegro | #N/A | #N/A | -0.83  (-1.78, 0.12) | #N/A | #N/A | #N/A | #N/A | #N/A | #N/A |
| Morocco | #N/A | #N/A | 1.87  (1.56, 2.19) | #N/A | #N/A | #N/A | #N/A | #N/A | #N/A |
| Mozambique | -2.43 (-3.5, -1.36) | #N/A | #N/A | #N/A | -10.81  (-11.73, -9.89) | #N/A | -4.76  (-5.85, -3.66) | #N/A | #N/A |
| Myanmar | -7.18 (-10.7, -3.51) | #N/A | #N/A | #N/A | -6.81  (-7.35, -6.26) | #N/A | -0.38  (-0.72, -0.05) | #N/A | #N/A |
| Namibia | -6.89 (-15.67, 2.8) | #N/A | -0.93  (-1.02, -0.85) | #N/A | #N/A | #N/A | #N/A | #N/A | #N/A |
| Nauru | #N/A | #N/A | #N/A | #N/A | #N/A | #N/A | 7.84  (6.58, 9.12) | #N/A | #N/A |
| Nepal | -12.07 (-14.53, -9.53) | #N/A | -9.51  (-10.3, -8.72) | #N/A | -6.13  (-6.28, -5.97) | #N/A | 0.55  (0.3, 0.81) | #N/A | #N/A |
| Netherlands | #N/A | 0.43 (0.36, 0.49) | #N/A | #N/A | #N/A | #N/A | #N/A | #N/A | #N/A |
| New Zealand | #N/A | #N/A | #N/A | #N/A | #N/A | #N/A | #N/A | #N/A | #N/A |
| Nicaragua | 3.38 (-3.15, 10.36) | -3.94 (-4.16, -3.71) | 1.2  (1.02, 1.38) | #N/A | #N/A | #N/A | 3.73  (0.65, 6.9) | #N/A | #N/A |
| Niger | -1.2 (-1.7, -0.7) | #N/A | 5.59  (4.92, 6.27) | #N/A | -7.78  (-8.1, -7.46) | -15.79  (-16.36, -15.21) | -0.32  (-0.73, 0.1) | -4.49  (-5.15, -3.82) | #N/A |
| Nigeria | -1.24 (-1.69, -0.78) | #N/A | -0.96  (-1.31, -0.62) | #N/A | -7.3  (-8.79, -5.78) | -3.66  (-3.73, -3.58) | 1.04  (0.8, 1.29) | -7.42  (-7.85, -6.99) | #N/A |
| Niue | #N/A | #N/A | #N/A | #N/A | -3.54  (-3.81, -3.27) | #N/A | 0.95  (0.85, 1.04) | #N/A | #N/A |
| North Macedonia | #N/A | #N/A | -2.8  (-3.3, -2.3) | #N/A | #N/A | #N/A | #N/A | #N/A | #N/A |
| Northern Mariana Islands | #N/A | #N/A | #N/A | #N/A | #N/A | #N/A | 0.62  (-0.68, 1.94) | #N/A | #N/A |
| Norway | #N/A | #N/A | #N/A | #N/A | #N/A | #N/A | #N/A | #N/A | #N/A |
| Oman | #N/A | #N/A | -8.24  (-9.27, -7.2) | #N/A | #N/A | #N/A | -1.72  (-2.21, -1.23) | #N/A | #N/A |
| Pakistan | -2.27 (-6.05, 1.67) | #N/A | -8.2  (-8.59, -7.8) | #N/A | #N/A | #N/A | 1.96  (1.69, 2.22) | #N/A | #N/A |
| Palau | #N/A | #N/A | #N/A | #N/A | -0.29  (-0.31, -0.26) | #N/A | 0.06  (-0.45, 0.57) | #N/A | #N/A |
| Palestine | #N/A | #N/A | -4.91  (-5.18, -4.63) | #N/A | #N/A | #N/A | -0.47  (-1.53, 0.6) | #N/A | #N/A |
| Panama | -6.1 (-14.45, 3.06) | -3.57 (-3.73, -3.42) | 0.31  (0.25, 0.36) | #N/A | #N/A | #N/A | 2.98  (2.18, 3.78) | -5.46  (-5.79, -5.13) | #N/A |
| Papua New Guinea | -3.87 (-6.75, -0.89) | #N/A | #N/A | #N/A | -4.5  (-4.85, -4.15) | #N/A | 0.18  (-0.11, 0.46) | #N/A | #N/A |
| Paraguay | #N/A | -4.4 (-4.68, -4.12) | 0.06  (-0.8, 0.93) | #N/A | #N/A | #N/A | 4.21  (0.49, 8.06) | -6.07  (-6.55, -5.59) | #N/A |
| Peru | -16.15 (-19.44, -12.73) | -3.9 (-4.2, -3.59) | 0.33  (0.29, 0.37) | #N/A | #N/A | #N/A | 2.25  (0.77, 3.76) | -6.92  (-8.19, -5.64) | #N/A |
| Philippines | -10 (-19.56, 0.71) | #N/A | #N/A | #N/A | -6.3  (-6.99, -5.61) | #N/A | 2.21  (-0.52, 5.01) | #N/A | #N/A |
| Poland | #N/A | #N/A | #N/A | #N/A | #N/A | #N/A | #N/A | #N/A | #N/A |
| Portugal | #N/A | 1.59 (1.43, 1.75) | -5.37  (-5.88, -4.86) | #N/A | #N/A | #N/A | #N/A | #N/A | #N/A |
| Puerto Rico | #N/A | -12.27 (-17.49, -6.71) | #N/A | #N/A | #N/A | #N/A | -0.56  (-0.82, -0.3) | #N/A | #N/A |
| Qatar | #N/A | #N/A | #N/A | #N/A | #N/A | #N/A | -2.08  (-2.3, -1.87) | #N/A | #N/A |
| Republic of Korea | -11.61 (-12.15, -11.07) | #N/A | #N/A | #N/A | #N/A | #N/A | -5.88  (-7.65, -4.08) | #N/A | #N/A |
| Republic of Moldova | #N/A | #N/A | #N/A | #N/A | #N/A | #N/A | #N/A | #N/A | #N/A |
| Romania | #N/A | #N/A | -15.13  (-15.67, -14.59) | #N/A | #N/A | #N/A | #N/A | #N/A | #N/A |
| Russian Federation | #N/A | #N/A | #N/A | #N/A | #N/A | #N/A | #N/A | #N/A | #N/A |
| Rwanda | -5 (-7.64, -2.28) | #N/A | #N/A | #N/A | #N/A | #N/A | -9.99  (-11.87, -8.08) | -5.91  (-6.64, -5.19) | #N/A |
| Saint Kitts and Nevis | #N/A | #N/A | #N/A | #N/A | #N/A | #N/A | -0.82  (-1.72, 0.09) | #N/A | #N/A |
| Saint Lucia | #N/A | -0.47 (-0.64, -0.3) | #N/A | #N/A | #N/A | #N/A | 2.77  (1.47, 4.09) | #N/A | #N/A |
| Saint Vincent and the Grenadines | #N/A | -3.18 (-3.92, -2.43) | #N/A | #N/A | #N/A | #N/A | 0.27  (-0.96, 1.51) | #N/A | #N/A |
| Samoa | #N/A | #N/A | #N/A | #N/A | -3.1  (-4.11, -2.09) | #N/A | 2.97  (2.51, 3.42) | #N/A | #N/A |
| San Marino | #N/A | #N/A | #N/A | #N/A | #N/A | #N/A | #N/A | #N/A | #N/A |
| Sao Tome and Principe | -5.93 (-14.72, 3.77) | #N/A | #N/A | #N/A | -3.26  (-3.63, -2.89) | #N/A | -0.1  (-0.4, 0.2) | -6.26  (-7.31, -5.21) | #N/A |
| Saudi Arabia | -9.1 (-15.37, -2.37) | #N/A | -2.49  (-2.55, -2.43) | #N/A | #N/A | #N/A | 2.74  (2.34, 3.14) | #N/A | #N/A |
| Senegal | -2.62 (-6.31, 1.22) | #N/A | 4.66  (3.88, 5.46) | #N/A | -5.24  (-5.63, -4.84) | -9.1  (-9.26, -8.93) | 1.82  (1.49, 2.15) | -6.39  (-7.83, -4.93) | #N/A |
| Serbia | #N/A | #N/A | -11.94  (-12.89, -10.98) | #N/A | #N/A | #N/A | #N/A | #N/A | #N/A |
| Seychelles | #N/A | #N/A | #N/A | #N/A | #N/A | #N/A | 1.55  (1.4, 1.71) | #N/A | #N/A |
| Sierra Leone | -0.76 (-1.39, -0.12) | #N/A | #N/A | #N/A | -6.23  (-6.4, -6.05) | -1.61  (-1.75, -1.47) | 0.09  (-0.19, 0.37) | -4.2  (-5.52, -2.86) | #N/A |
| Singapore | #N/A | #N/A | #N/A | #N/A | #N/A | #N/A | 0.51  (-0.23, 1.26) | #N/A | #N/A |
| Slovakia | #N/A | #N/A | #N/A | #N/A | #N/A | #N/A | #N/A | #N/A | #N/A |
| Slovenia | #N/A | #N/A | -6.85  (-7.57, -6.12) | #N/A | #N/A | #N/A | #N/A | #N/A | #N/A |
| Solomon Islands | -4.3 (-6.45, -2.1) | #N/A | #N/A | #N/A | #N/A | #N/A | 2.98  (2.01, 3.95) | #N/A | #N/A |
| Somalia | -5.08 (-6.42, -3.72) | #N/A | -13.56  (-14.73, -12.38) | #N/A | #N/A | #N/A | -8.29  (-9.08, -7.49) | -2.4  (-2.66, -2.13) | #N/A |
| South Africa | -5.43 (-8.06, -2.72) | #N/A | 0.21  (0.06, 0.36) | #N/A | #N/A | #N/A | #N/A | #N/A | #N/A |
| South Sudan | 0.35 (-0.37, 1.08) | #N/A | -7.58  (-11.82, -3.13) | -5.76  (-14.96, 4.44) | -3.27  (-3.55, -2.99) | -0.23  (-0.29, -0.18) | -14.28  (-15.78, -12.74) | -4.19  (-4.63, -3.76) | #N/A |
| Spain | #N/A | 4.11 (3.73, 4.49) | -5.01  (-5.89, -4.13) | #N/A | #N/A | #N/A | -4.96  (-5.36, -4.55) | #N/A | #N/A |
| Sri Lanka | #N/A | #N/A | 2.13  (1.89, 2.38) | #N/A | #N/A | #N/A | -0.32  (-11.99, 12.9) | #N/A | #N/A |
| Sudan | -2.31 (-3.01, -1.61) | #N/A | -4.63  (-5.56, -3.69) | #N/A | -3.57  (-3.75, -3.4) | -3.67  (-3.78, -3.56) | 1.08  (0.7, 1.45) | -7.93  (-8.12, -7.75) | #N/A |
| Suriname | -12.37 (-19.79, -4.26) | -9.51 (-10.09, -8.93) | -0.59  (-0.68, -0.5) | #N/A | #N/A | #N/A | 3.47  (1.37, 5.61) | -5.06  (-5.67, -4.45) | #N/A |
| Sweden | #N/A | -1.88 (-1.93, -1.84) | #N/A | #N/A | #N/A | #N/A | #N/A | #N/A | #N/A |
| Switzerland | #N/A | -0.31 (-0.42, -0.19) | #N/A | #N/A | #N/A | #N/A | #N/A | #N/A | #N/A |
| Syrian Arab Republic | #N/A | #N/A | 2.19  (2.09, 2.3) | #N/A | #N/A | #N/A | -0.32  (-3.76, 3.25) | #N/A | #N/A |
| Tajikistan | #N/A | #N/A | -3.34  (-3.56, -3.11) | #N/A | #N/A | #N/A | #N/A | #N/A | #N/A |
| Thailand | -16.71 (-19.88, -13.41) | #N/A | -3.53  (-3.94, -3.12) | #N/A | #N/A | #N/A | -2.71  (-4.19, -1.21) | #N/A | #N/A |
| Timor-Leste | #N/A | #N/A | #N/A | #N/A | -3.28  (-6.86, 0.45) | #N/A | -2.06  (-7.99, 4.26) | #N/A | #N/A |
| Togo | -1.27 (-1.86, -0.68) | #N/A | #N/A | #N/A | #N/A | -13.89  (-14.17, -13.62) | 0.82  (0.58, 1.06) | -5.25  (-5.75, -4.75) | #N/A |
| Tokelau | #N/A | #N/A | #N/A | #N/A | #N/A | #N/A | 1.29  (0.33, 2.26) | #N/A | #N/A |
| Tonga | #N/A | #N/A | #N/A | #N/A | -1.85  (-3.23, -0.45) | #N/A | 4.34  (2.36, 6.37) | #N/A | #N/A |
| Trinidad and Tobago | #N/A | -3.74 (-4.41, -3.06) | #N/A | #N/A | #N/A | #N/A | 3.17  (1.79, 4.58) | -5.58  (-5.82, -5.33) | #N/A |
| Tunisia | #N/A | #N/A | 0.54  (0.35, 0.73) | #N/A | #N/A | #N/A | #N/A | #N/A | #N/A |
| Türkiye | #N/A | #N/A | -5.61  (-6.16, -5.06) | #N/A | #N/A | #N/A | #N/A | #N/A | #N/A |
| Turkmenistan | #N/A | #N/A | -2.42  (-2.49, -2.35) | #N/A | #N/A | #N/A | #N/A | #N/A | #N/A |
| Tuvalu | #N/A | #N/A | #N/A | #N/A | #N/A | #N/A | 4.41  (3.95, 4.87) | #N/A | #N/A |
| Uganda | -1.94 (-2.67, -1.19) | #N/A | -11.28  (-14.21, -8.26) | -25.86  (-30.54, -20.88) | -6.44  (-6.94, -5.93) | -4.31  (-4.42, -4.2) | -9.23  (-10.44, -8.01) | -6.57  (-6.96, -6.19) | #N/A |
| Ukraine | #N/A | #N/A | #N/A | #N/A | #N/A | #N/A | #N/A | #N/A | #N/A |
| United Arab Emirates | #N/A | #N/A | #N/A | #N/A | #N/A | #N/A | #N/A | #N/A | #N/A |
| United Kingdom | #N/A | 3.92 (3.78, 4.06) | #N/A | #N/A | #N/A | #N/A | #N/A | #N/A | #N/A |
| United Republic of Tanzania | -4.47 (-5.09, -3.84) | #N/A | #N/A | #N/A | -8.16  (-8.54, -7.79) | -3.42  (-3.48, -3.36) | -11.62  (-14.11, -9.06) | -5.36  (-5.49, -5.24) | #N/A |
| United States of America | #N/A | -0.43 (-0.61, -0.24) | 0.35  (0.33, 0.38) | #N/A | #N/A | #N/A | 5.75  (4.69, 6.82) | #N/A | #N/A |
| United States Virgin Islands | #N/A | #N/A | #N/A | #N/A | #N/A | #N/A | -0.35  (-0.8, 0.11) | #N/A | #N/A |
| Uruguay | #N/A | -3.44 (-3.56, -3.33) | #N/A | #N/A | #N/A | #N/A | #N/A | #N/A | #N/A |
| Uzbekistan | #N/A | #N/A | -0.71  (-0.84, -0.58) | #N/A | #N/A | #N/A | #N/A | #N/A | #N/A |
| Vanuatu | -16.38 (-23.87, -8.17) | #N/A | #N/A | #N/A | -6.53  (-8.26, -4.76) | #N/A | 3.75  (3.29, 4.21) | #N/A | #N/A |
| Venezuela  (Bolivarian Republic of) | 1.38 (-5.24, 8.46) | -3.63 (-4.13, -3.13) | -0.57  (-0.81, -0.32) | #N/A | #N/A | -10.53  (-10.78, -10.29) | 2.8  (2.15, 3.45) | -4.94  (-5.75, -4.13) | #N/A |
| Viet Nam | -16.15 (-19.35, -12.83) | #N/A | #N/A | #N/A | -6.62  (-7.13, -6.1) | #N/A | 2.06  (-0.32, 4.5) | #N/A | #N/A |
| Yemen | -1.52 (-4.26, 1.29) | #N/A | -4.52  (-4.85, -4.19) | #N/A | -0.41  (-0.66, -0.17) | -2.52  (-2.56, -2.48) | -2.76  (-4.21, -1.28) | #N/A | #N/A |
| Zambia | -2.77 (-4.6, -0.9) | #N/A | -13.1  (-14.2, -11.99) | 14.3  (-40.43, 119.3) | -6.98  (-7.17, -6.79) | #N/A | -8.21  (-9.87, -6.52) | -6.12  (-6.41, -5.82) | #N/A |
| Zimbabwe | -0.7 (-8.37, 7.61) | #N/A | #N/A | #N/A | -0.8  (-1.15, -0.45) | #N/A | -0.69  (-1.76, 0.39) | #N/A | #N/A |

**Table K** Estimated ASDRs for selected infectious diseases through 2030

| Year | ABDs  mean | ABDs  sd | protozoiasis mean | protozoiasis sd | helminthiases mean | helminthiases sd | viral diseases mean | viral diseases sd |
| --- | --- | --- | --- | --- | --- | --- | --- | --- |
| 1990 | 0.0113359849428125 | 1.36328315222547e-06 | 0.00988139254633934 | 1.25367751341873e-06 | 0.00108313639611571 | 4.7205217489141e-07 | 0.00037143332387039 | 2.52645967179621e-07 |
| 1991 | 0.0113835208958794 | 1.35797953033304e-06 | 0.00997116958826214 | 1.25329954424368e-06 | 0.00105890524156018 | 4.61762305882555e-07 | 0.000353436468116601 | 2.44366865809589e-07 |
| 1992 | 0.0113219981797128 | 1.34795786274576e-06 | 0.00995048622025376 | 1.24754463647466e-06 | 0.00103665244959404 | 4.52073069159055e-07 | 0.000334870646478536 | 2.36351032846115e-07 |
| 1993 | 0.0112248037589423 | 1.33708710855727e-06 | 0.00987011106669396 | 1.23905421108974e-06 | 0.00101719050457729 | 4.43272615027116e-07 | 0.000337495844341732 | 2.35953650494848e-07 |
| 1994 | 0.0112323841340439 | 1.33403129269938e-06 | 0.00989489126470471 | 1.23882738171231e-06 | 0.00100101233912222 | 4.3543965781666e-07 | 0.000336486457688986 | 2.34468407472916e-07 |
| 1995 | 0.0113095356632282 | 1.3356623953735e-06 | 0.00997249929333589 | 1.24230012041268e-06 | 0.00098953551472739 | 4.28985099450126e-07 | 0.000347468804511018 | 2.37283246336732e-07 |
| 1996 | 0.0114420696772942 | 1.34023238190675e-06 | 0.0101210122233331 | 1.24988670747032e-06 | 0.00097907964934232 | 4.22864680022453e-07 | 0.000341971278391995 | 2.34222259426567e-07 |
| 1997 | 0.0116779195393903 | 1.35040301003671e-06 | 0.010382341392534 | 1.26397248981012e-06 | 0.000965498631073592 | 4.16063957024015e-07 | 0.000330103792372907 | 2.2922414328774e-07 |
| 1998 | 0.0118384483074033 | 1.35545590703792e-06 | 0.0105380492632909 | 1.27061904863308e-06 | 0.000950771574863093 | 4.0916019756309e-07 | 0.000349598209011135 | 2.34675733314175e-07 |
| 1999 | 0.0118734228697652 | 1.35322064346227e-06 | 0.0106086382481056 | 1.27183945039138e-06 | 0.000936257501151928 | 4.02396420634499e-07 | 0.000328556185260207 | 2.26783942053059e-07 |
| 2000 | 0.0118219922520317 | 1.34608505048229e-06 | 0.010563471159349 | 1.26596314707563e-06 | 0.00092387014547409 | 3.96296172793566e-07 | 0.000334653395413546 | 2.2795565133341e-07 |
| 2001 | 0.0119334266991897 | 1.34755568534156e-06 | 0.0106987255496681 | 1.2703177986067e-06 | 0.000901237392198047 | 3.88046742731993e-07 | 0.000333464436670675 | 2.26634096774166e-07 |
| 2002 | 0.0119465259352505 | 1.34320285542743e-06 | 0.0107542173247471 | 1.26964263088482e-06 | 0.000860733522648039 | 3.75896788127818e-07 | 0.000331587324039092 | 2.25079800794943e-07 |
| 2003 | 0.0119709282309264 | 1.33859497581777e-06 | 0.0108230994803837 | 1.26874762664559e-06 | 0.000812045444845654 | 3.61971898046168e-07 | 0.000335789247575436 | 2.25521267004817e-07 |
| 2004 | 0.0119343663950915 | 1.33007389699879e-06 | 0.0108185543578473 | 1.26289032007441e-06 | 0.000765396036205669 | 3.4846584868891e-07 | 0.000350402298174744 | 2.29242098333672e-07 |
| 2005 | 0.0116122373497731 | 1.30565269937165e-06 | 0.0105417639487372 | 1.24094388494298e-06 | 0.000731365977843275 | 3.37934536477636e-07 | 0.000339124395852176 | 2.24450338568109e-07 |
| 2006 | 0.0112384166345774 | 1.27769667276386e-06 | 0.0102110401322687 | 1.21515188896777e-06 | 0.000687720102094664 | 3.25182263693492e-07 | 0.000339658888298751 | 2.23537310200139e-07 |
| 2007 | 0.0109171954097727 | 1.25178496295862e-06 | 0.0099504370281155 | 1.19266652924672e-06 | 0.000616348329153493 | 3.05480968097042e-07 | 0.000350392525227894 | 2.25826178380701e-07 |
| 2008 | 0.0107301163322259 | 1.23336833132907e-06 | 0.00984532920412574 | 1.17935265962676e-06 | 0.000536159266469537 | 2.82756158060245e-07 | 0.000348635350232709 | 2.24032045417929e-07 |
| 2009 | 0.0104721960910572 | 1.21074074358294e-06 | 0.00964733410114343 | 1.16024088040945e-06 | 0.000467194670759047 | 2.61978609085168e-07 | 0.000357680473646676 | 2.25672130034469e-07 |
| 2010 | 0.0102204610651115 | 1.18982885214206e-06 | 0.00942047177118363 | 1.14065460157586e-06 | 0.000430841251877558 | 2.4981992039809e-07 | 0.000369159857129158 | 2.28087598577347e-07 |
| 2011 | 0.0096680502700707 | 1.15219392584631e-06 | 0.00886055937972845 | 1.10147182161632e-06 | 0.000424457114489482 | 2.46292725858616e-07 | 0.000383020910419708 | 2.31274307209537e-07 |
| 2012 | 0.00918306190422155 | 1.11825073193464e-06 | 0.00838549249233335 | 1.06718462931334e-06 | 0.000427032770315938 | 2.4538388556755e-07 | 0.000370544762834913 | 2.26351566735812e-07 |
| 2013 | 0.00856853837676184 | 1.0755450691616e-06 | 0.00776263265262249 | 1.02241608845049e-06 | 0.00043442600983662 | 2.45910558773018e-07 | 0.000371482491487576 | 2.25483976826656e-07 |
| 2014 | 0.00832113863591892 | 1.05583811689068e-06 | 0.00750949781581434 | 1.00187824456504e-06 | 0.000442280776125978 | 2.46626403661e-07 | 0.000369357171361824 | 2.23757985794158e-07 |
| 2015 | 0.0082024523784052 | 1.04410325648385e-06 | 0.00739154121491627 | 9.90141724015904e-07 | 0.000445394475819809 | 2.4610074243973e-07 | 0.000365519057855121 | 2.21513975593787e-07 |
| 2016 | 0.00783634777191475 | 1.01596492942924e-06 | 0.00704053718945372 | 9.62060573826289e-07 | 0.000430294080033868 | 2.40575284261228e-07 | 0.000365507066556322 | 2.20484170526435e-07 |
| 2017 | 0.00752368076781352 | 9.92085678775304e-07 | 0.00676527978041505 | 9.40037060765745e-07 | 0.000395675945405274 | 2.29420299559361e-07 | 0.000362710171436908 | 2.18632049476425e-07 |
| 2018 | 0.00738684925096716 | 9.81254536466125e-07 | 0.00668905288374478 | 9.33383836248051e-07 | 0.000359236778097174 | 2.17394431085916e-07 | 0.000338571928777984 | 2.10410650879054e-07 |
| 2019 | 0.00728837366370241 | 9.74649089109265e-07 | 0.00661508655764922 | 9.28575532686671e-07 | 0.000339051028135556 | 2.10097844109591e-07 | 0.000334235907787809 | 2.08397777512336e-07 |
| 2020 | 0.00802746058489243 | 1.02517066644533e-06 | 0.00737967128461256 | 9.83458078808716e-07 | 0.000333563992884182 | 2.07419692903703e-07 | 0.00031422051384308 | 2.01616574716245e-07 |
| 2021 | 0.00806337627477074 | 1.03169186341509e-06 | 0.00742665287268284 | 9.91205187423112e-07 | 0.000328568015288915 | 2.05072117465902e-07 | 0.000308144988256563 | 1.99521128093805e-07 |
| 2022 | 0.00824677554895474 | 0.0019929679859494 | 0.00767869916833145 | 0.00188025835367067 | 0.000325619226161991 | 1.13256216094434e-05 | 0.000298750059763668 | 9.15843383272428e-06 |
| 2023 | 0.00835756261170427 | 0.00411972143053848 | 0.00784059922327187 | 0.00354661941901905 | 0.000321196506554948 | 2.12282277892348e-05 | 0.000290117593643985 | 1.39150675510105e-05 |
| 2024 | 0.00848355321389888 | 0.00681462393710739 | 0.0080180703931162 | 0.00571494518376948 | 0.000317500508293122 | 3.33622101862601e-05 | 0.000281777337423265 | 1.988666500406e-05 |
| 2025 | 0.00866339946546204 | 0.0100425034119771 | 0.0082350892281172 | 0.00834599977450061 | 0.000314579694079061 | 4.72125547703763e-05 | 0.000273680146227616 | 2.65751865268645e-05 |
| 2026 | 0.00879358198525929 | 0.0137449509890398 | 0.00842027889735412 | 0.0113942009899679 | 0.000312428217185473 | 6.25765360757915e-05 | 0.000265803139293004 | 3.37252215975843e-05 |
| 2027 | 0.00893819572560266 | 0.0179822160613142 | 0.00861245994072095 | 0.0148865362982201 | 0.000311003569504463 | 7.93748413466215e-05 | 0.000258105658984572 | 4.1178376358982e-05 |
| 2028 | 0.00907389757734067 | 0.0227179911451164 | 0.00880562919230069 | 0.0188137161783482 | 0.000310254913049618 | 9.75673779998848e-05 | 0.00025064752632872 | 4.88250427491264e-05 |
| 2029 | 0.00922297043084219 | 0.0279692071750475 | 0.00901458810775654 | 0.0231995932047298 | 0.00031021841158451 | 0.000117182427029325 | 0.000243443149218873 | 5.65861677161655e-05 |
| 2030 | 0.00941656551054078 | 0.0337944854164888 | 0.00925956364502952 | 0.0280985851226246 | 0.000310943002578343 | 0.000138299832690778 | 0.000236461216171209 | 6.43948712550665e-05 |

ABDs arthropod-borne diseases, ASDRs age-standardized death rates, sd standard deviation

**Table L** Projections of age-standardized death rates (ASDR) for ABDs, 1990–2030, with 95% credible intervals

| **Year** | **mean** | **Lower 95%CI** | **Upper 95%CI** |
| --- | --- | --- | --- |
| **ABDs** |  |  |  |
| 1990 | 0.011336 | 0.011333 | 0.011339 |
| 1991 | 0.011384 | 0.011381 | 0.011386 |
| 1992 | 0.011322 | 0.011319 | 0.011325 |
| 1993 | 0.011225 | 0.011222 | 0.011227 |
| 1994 | 0.011232 | 0.01123 | 0.011235 |
| 1995 | 0.01131 | 0.011307 | 0.011312 |
| 1996 | 0.011442 | 0.011439 | 0.011445 |
| 1997 | 0.011678 | 0.011675 | 0.011681 |
| 1998 | 0.011838 | 0.011836 | 0.011841 |
| 1999 | 0.011873 | 0.011871 | 0.011876 |
| 2000 | 0.011822 | 0.011819 | 0.011825 |
| 2001 | 0.011933 | 0.011931 | 0.011936 |
| 2002 | 0.011947 | 0.011944 | 0.011949 |
| 2003 | 0.011971 | 0.011968 | 0.011974 |
| 2004 | 0.011934 | 0.011932 | 0.011937 |
| 2005 | 0.011612 | 0.01161 | 0.011615 |
| 2006 | 0.011238 | 0.011236 | 0.011241 |
| 2007 | 0.010917 | 0.010915 | 0.01092 |
| 2008 | 0.01073 | 0.010728 | 0.010733 |
| 2009 | 0.010472 | 0.01047 | 0.010475 |
| 2010 | 0.01022 | 0.010218 | 0.010223 |
| 2011 | 0.009668 | 0.009666 | 0.00967 |
| 2012 | 0.009183 | 0.009181 | 0.009185 |
| 2013 | 0.008569 | 0.008566 | 0.008571 |
| 2014 | 0.008321 | 0.008319 | 0.008323 |
| 2015 | 0.008202 | 0.0082 | 0.008204 |
| 2016 | 0.007836 | 0.007834 | 0.007838 |
| 2017 | 0.007524 | 0.007522 | 0.007526 |
| 2018 | 0.007387 | 0.007385 | 0.007389 |
| 2019 | 0.007288 | 0.007286 | 0.00729 |
| 2020 | 0.008027 | 0.008025 | 0.008029 |
| 2021 | 0.008063 | 0.008061 | 0.008065 |
| 2022 | 0.008247 | 0.004341 | 0.012153 |
| 2023 | 0.008358 | 0.000283 | 0.016432 |
| 2024 | 0.008484 | -0.004873 | 0.02184 |
| 2025 | 0.008663 | -0.01102 | 0.028347 |
| 2026 | 0.008794 | -0.018147 | 0.035734 |
| 2027 | 0.008938 | -0.026307 | 0.044183 |
| 2028 | 0.009074 | -0.035453 | 0.053601 |
| 2029 | 0.009223 | -0.045597 | 0.064043 |
| 2030 | 0.009417 | -0.056821 | 0.075654 |
| **Prorozoa** |  |  |  |
| 1990 | 0.009881 | 0.009879 | 0.009884 |
| 1991 | 0.009971 | 0.009969 | 0.009974 |
| 1992 | 0.00995 | 0.009948 | 0.009953 |
| 1993 | 0.00987 | 0.009868 | 0.009873 |
| 1994 | 0.009895 | 0.009892 | 0.009897 |
| 1995 | 0.009972 | 0.00997 | 0.009975 |
| 1996 | 0.010121 | 0.010119 | 0.010123 |
| 1997 | 0.010382 | 0.01038 | 0.010385 |
| 1998 | 0.010538 | 0.010536 | 0.010541 |
| 1999 | 0.010609 | 0.010606 | 0.010611 |
| 2000 | 0.010563 | 0.010561 | 0.010566 |
| 2001 | 0.010699 | 0.010696 | 0.010701 |
| 2002 | 0.010754 | 0.010752 | 0.010757 |
| 2003 | 0.010823 | 0.010821 | 0.010826 |
| 2004 | 0.010819 | 0.010816 | 0.010821 |
| 2005 | 0.010542 | 0.010539 | 0.010544 |
| 2006 | 0.010211 | 0.010209 | 0.010213 |
| 2007 | 0.00995 | 0.009948 | 0.009953 |
| 2008 | 0.009845 | 0.009843 | 0.009848 |
| 2009 | 0.009647 | 0.009645 | 0.00965 |
| 2010 | 0.00942 | 0.009418 | 0.009423 |
| 2011 | 0.008861 | 0.008858 | 0.008863 |
| 2012 | 0.008385 | 0.008383 | 0.008388 |
| 2013 | 0.007763 | 0.007761 | 0.007765 |
| 2014 | 0.007509 | 0.007508 | 0.007511 |
| 2015 | 0.007392 | 0.00739 | 0.007393 |
| 2016 | 0.007041 | 0.007039 | 0.007042 |
| 2017 | 0.006765 | 0.006763 | 0.006767 |
| 2018 | 0.006689 | 0.006687 | 0.006691 |
| 2019 | 0.006615 | 0.006613 | 0.006617 |
| 2020 | 0.00738 | 0.007378 | 0.007382 |
| 2021 | 0.007427 | 0.007425 | 0.007429 |
| 2022 | 0.007679 | 0.003993 | 0.011364 |
| 2023 | 0.007841 | 0.000889 | 0.014792 |
| 2024 | 0.008018 | -0.003183 | 0.019219 |
| 2025 | 0.008235 | -0.008123 | 0.024593 |
| 2026 | 0.00842 | -0.013912 | 0.030753 |
| 2027 | 0.008612 | -0.020565 | 0.03779 |
| 2028 | 0.008806 | -0.028069 | 0.045681 |
| 2029 | 0.009015 | -0.036457 | 0.054486 |
| 2030 | 0.00926 | -0.045814 | 0.064333 |
| **Helminths** |  |  |  |
| 1990 | 0.001083 | 0.001082 | 0.001084 |
| 1991 | 0.001059 | 0.001058 | 0.00106 |
| 1992 | 0.001037 | 0.001036 | 0.001038 |
| 1993 | 0.001017 | 0.001016 | 0.001018 |
| 1994 | 0.001001 | 0.001 | 0.001002 |
| 1995 | 0.00099 | 0.000989 | 0.00099 |
| 1996 | 0.000979 | 0.000978 | 0.00098 |
| 1997 | 0.000965 | 0.000965 | 0.000966 |
| 1998 | 0.000951 | 0.00095 | 0.000952 |
| 1999 | 0.000936 | 0.000935 | 0.000937 |
| 2000 | 0.000924 | 0.000923 | 0.000925 |
| 2001 | 0.000901 | 9e-04 | 0.000902 |
| 2002 | 0.000861 | 0.00086 | 0.000861 |
| 2003 | 0.000812 | 0.000811 | 0.000813 |
| 2004 | 0.000765 | 0.000765 | 0.000766 |
| 2005 | 0.000731 | 0.000731 | 0.000732 |
| 2006 | 0.000688 | 0.000687 | 0.000688 |
| 2007 | 0.000616 | 0.000616 | 0.000617 |
| 2008 | 0.000536 | 0.000536 | 0.000537 |
| 2009 | 0.000467 | 0.000467 | 0.000468 |
| 2010 | 0.000431 | 0.00043 | 0.000431 |
| 2011 | 0.000424 | 0.000424 | 0.000425 |
| 2012 | 0.000427 | 0.000427 | 0.000428 |
| 2013 | 0.000434 | 0.000434 | 0.000435 |
| 2014 | 0.000442 | 0.000442 | 0.000443 |
| 2015 | 0.000445 | 0.000445 | 0.000446 |
| 2016 | 0.00043 | 0.00043 | 0.000431 |
| 2017 | 0.000396 | 0.000395 | 0.000396 |
| 2018 | 0.000359 | 0.000359 | 0.00036 |
| 2019 | 0.000339 | 0.000339 | 0.000339 |
| 2020 | 0.000334 | 0.000333 | 0.000334 |
| 2021 | 0.000329 | 0.000328 | 0.000329 |
| 2022 | 0.000326 | 0.000303 | 0.000348 |
| 2023 | 0.000321 | 0.00028 | 0.000363 |
| 2024 | 0.000318 | 0.000252 | 0.000383 |
| 2025 | 0.000315 | 0.000222 | 0.000407 |
| 2026 | 0.000312 | 0.00019 | 0.000435 |
| 2027 | 0.000311 | 0.000155 | 0.000467 |
| 2028 | 0.00031 | 0.000119 | 0.000501 |
| 2029 | 0.00031 | 8.1e-05 | 0.00054 |
| 2030 | 0.000311 | 4e-05 | 0.000582 |
| **Viruses** |  |  |  |
| 1990 | 0.000371 | 0.000371 | 0.000372 |
| 1991 | 0.000353 | 0.000353 | 0.000354 |
| 1992 | 0.000335 | 0.000334 | 0.000335 |
| 1993 | 0.000337 | 0.000337 | 0.000338 |
| 1994 | 0.000336 | 0.000336 | 0.000337 |
| 1995 | 0.000347 | 0.000347 | 0.000348 |
| 1996 | 0.000342 | 0.000342 | 0.000342 |
| 1997 | 0.00033 | 0.00033 | 0.000331 |
| 1998 | 0.00035 | 0.000349 | 0.00035 |
| 1999 | 0.000329 | 0.000328 | 0.000329 |
| 2000 | 0.000335 | 0.000334 | 0.000335 |
| 2001 | 0.000333 | 0.000333 | 0.000334 |
| 2002 | 0.000332 | 0.000331 | 0.000332 |
| 2003 | 0.000336 | 0.000335 | 0.000336 |
| 2004 | 0.00035 | 0.00035 | 0.000351 |
| 2005 | 0.000339 | 0.000339 | 0.00034 |
| 2006 | 0.00034 | 0.000339 | 0.00034 |
| 2007 | 0.00035 | 0.00035 | 0.000351 |
| 2008 | 0.000349 | 0.000348 | 0.000349 |
| 2009 | 0.000358 | 0.000357 | 0.000358 |
| 2010 | 0.000369 | 0.000369 | 0.00037 |
| 2011 | 0.000383 | 0.000383 | 0.000383 |
| 2012 | 0.000371 | 0.00037 | 0.000371 |
| 2013 | 0.000371 | 0.000371 | 0.000372 |
| 2014 | 0.000369 | 0.000369 | 0.00037 |
| 2015 | 0.000366 | 0.000365 | 0.000366 |
| 2016 | 0.000366 | 0.000365 | 0.000366 |
| 2017 | 0.000363 | 0.000362 | 0.000363 |
| 2018 | 0.000339 | 0.000338 | 0.000339 |
| 2019 | 0.000334 | 0.000334 | 0.000335 |
| 2020 | 0.000314 | 0.000314 | 0.000315 |
| 2021 | 0.000308 | 0.000308 | 0.000309 |
| 2022 | 0.000299 | 0.000281 | 0.000317 |
| 2023 | 0.00029 | 0.000263 | 0.000317 |
| 2024 | 0.000282 | 0.000243 | 0.000321 |
| 2025 | 0.000274 | 0.000222 | 0.000326 |
| 2026 | 0.000266 | 2e-04 | 0.000332 |
| 2027 | 0.000258 | 0.000177 | 0.000339 |
| 2028 | 0.000251 | 0.000155 | 0.000346 |
| 2029 | 0.000243 | 0.000133 | 0.000354 |
| 2030 | 0.000236 | 0.00011 | 0.000363 |
| **Chagas disease** |  |  |  |
| 1990 | 9.6e-05 | 9.6e-05 | 9.7e-05 |
| 1991 | 9.1e-05 | 9.1e-05 | 9.2e-05 |
| 1992 | 8.8e-05 | 8.8e-05 | 8.8e-05 |
| 1993 | 8.5e-05 | 8.4e-05 | 8.5e-05 |
| 1994 | 8e-05 | 8e-05 | 8.1e-05 |
| 1995 | 7.7e-05 | 7.6e-05 | 7.7e-05 |
| 1996 | 7.3e-05 | 7.2e-05 | 7.3e-05 |
| 1997 | 6.9e-05 | 6.9e-05 | 6.9e-05 |
| 1998 | 6.6e-05 | 6.6e-05 | 6.6e-05 |
| 1999 | 6.3e-05 | 6.3e-05 | 6.3e-05 |
| 2000 | 6e-05 | 5.9e-05 | 6e-05 |
| 2001 | 5.7e-05 | 5.7e-05 | 5.7e-05 |
| 2002 | 5.5e-05 | 5.4e-05 | 5.5e-05 |
| 2003 | 5.3e-05 | 5.3e-05 | 5.3e-05 |
| 2004 | 5.1e-05 | 5.1e-05 | 5.1e-05 |
| 2005 | 4.9e-05 | 4.8e-05 | 4.9e-05 |
| 2006 | 4.7e-05 | 4.7e-05 | 4.7e-05 |
| 2007 | 4.5e-05 | 4.5e-05 | 4.5e-05 |
| 2008 | 4.3e-05 | 4.3e-05 | 4.3e-05 |
| 2009 | 4.1e-05 | 4.1e-05 | 4.1e-05 |
| 2010 | 4e-05 | 3.9e-05 | 4e-05 |
| 2011 | 3.8e-05 | 3.8e-05 | 3.8e-05 |
| 2012 | 3.6e-05 | 3.6e-05 | 3.6e-05 |
| 2013 | 3.5e-05 | 3.5e-05 | 3.5e-05 |
| 2014 | 3.4e-05 | 3.4e-05 | 3.4e-05 |
| 2015 | 3.3e-05 | 3.2e-05 | 3.3e-05 |
| 2016 | 3.2e-05 | 3.2e-05 | 3.2e-05 |
| 2017 | 3.1e-05 | 3.1e-05 | 3.1e-05 |
| 2018 | 3e-05 | 2.9e-05 | 3e-05 |
| 2019 | 2.9e-05 | 2.9e-05 | 2.9e-05 |
| 2020 | 2.9e-05 | 2.8e-05 | 2.9e-05 |
| 2021 | 2.8e-05 | 2.8e-05 | 2.8e-05 |
| 2022 | 2.6e-05 | 2.6e-05 | 2.7e-05 |
| 2023 | 2.5e-05 | 2.5e-05 | 2.6e-05 |
| 2024 | 2.5e-05 | 2.3e-05 | 2.6e-05 |
| 2025 | 2.4e-05 | 2.2e-05 | 2.6e-05 |
| 2026 | 2.3e-05 | 2.1e-05 | 2.5e-05 |
| 2027 | 2.2e-05 | 2e-05 | 2.5e-05 |
| 2028 | 2.2e-05 | 1.9e-05 | 2.4e-05 |
| 2029 | 2.1e-05 | 1.8e-05 | 2.4e-05 |
| 2030 | 2e-05 | 1.7e-05 | 2.4e-05 |
| **African trypanosomiasis** |  |  |  |
| 1990 | 0.000199 | 0.000198 | 0.000199 |
| 1991 | 0.000174 | 0.000174 | 0.000174 |
| 1992 | 0.000205 | 0.000204 | 0.000205 |
| 1993 | 0.000208 | 0.000207 | 0.000208 |
| 1994 | 0.000241 | 0.000241 | 0.000242 |
| 1995 | 0.000256 | 0.000255 | 0.000256 |
| 1996 | 0.000284 | 0.000283 | 0.000284 |
| 1997 | 0.000336 | 0.000336 | 0.000337 |
| 1998 | 0.000284 | 0.000284 | 0.000285 |
| 1999 | 0.000232 | 0.000232 | 0.000232 |
| 2000 | 0.000205 | 0.000204 | 0.000205 |
| 2001 | 0.000202 | 0.000202 | 0.000203 |
| 2002 | 0.000188 | 0.000188 | 0.000189 |
| 2003 | 0.000143 | 0.000143 | 0.000144 |
| 2004 | 0.000121 | 0.000121 | 0.000121 |
| 2005 | 0.000112 | 0.000112 | 0.000113 |
| 2006 | 8.1e-05 | 8.1e-05 | 8.1e-05 |
| 2007 | 6.8e-05 | 6.8e-05 | 6.8e-05 |
| 2008 | 7.1e-05 | 7.1e-05 | 7.1e-05 |
| 2009 | 6.3e-05 | 6.3e-05 | 6.3e-05 |
| 2010 | 4.6e-05 | 4.6e-05 | 4.7e-05 |
| 2011 | 4.1e-05 | 4.1e-05 | 4.1e-05 |
| 2012 | 4.3e-05 | 4.3e-05 | 4.3e-05 |
| 2013 | 3.7e-05 | 3.7e-05 | 3.7e-05 |
| 2014 | 2.5e-05 | 2.5e-05 | 2.5e-05 |
| 2015 | 1.8e-05 | 1.8e-05 | 1.8e-05 |
| 2016 | 1.6e-05 | 1.6e-05 | 1.6e-05 |
| 2017 | 1.2e-05 | 1.2e-05 | 1.2e-05 |
| 2018 | 8e-06 | 8e-06 | 8e-06 |
| 2019 | 7e-06 | 7e-06 | 7e-06 |
| 2020 | 7e-06 | 7e-06 | 7e-06 |
| 2021 | 8e-06 | 8e-06 | 8e-06 |
| 2022 | 9e-06 | 6e-06 | 1.2e-05 |
| 2023 | 1e-05 | 2e-06 | 1.9e-05 |
| 2024 | 1.2e-05 | -4.00E-06 | 2.8e-05 |
| 2025 | 1.4e-05 | -1.20E-05 | 4.1e-05 |
| 2026 | 1.6e-05 | -2.50E-05 | 5.8e-05 |
| 2027 | 1.9e-05 | -4.30E-05 | 8.1e-05 |
| 2028 | 2.2e-05 | -6.70E-05 | 0.000111 |
| 2029 | 2.5e-05 | -9.90E-05 | 0.000149 |
| 2030 | 2.9e-05 | -0.000141 | 0.000199 |
| **Leishmaniasis** |  |  |  |
| 1990 | 0.000795 | 0.000794 | 0.000795 |
| 1991 | 0.000738 | 0.000737 | 0.000739 |
| 1992 | 0.000622 | 0.000621 | 0.000623 |
| 1993 | 0.000502 | 0.000501 | 0.000503 |
| 1994 | 0.000411 | 0.00041 | 0.000411 |
| 1995 | 0.000324 | 0.000323 | 0.000324 |
| 1996 | 0.000276 | 0.000276 | 0.000276 |
| 1997 | 0.000239 | 0.000239 | 0.000239 |
| 1998 | 0.000215 | 0.000215 | 0.000216 |
| 1999 | 0.000202 | 0.000202 | 0.000202 |
| 2000 | 0.000195 | 0.000194 | 0.000195 |
| 2001 | 0.00019 | 0.000189 | 0.00019 |
| 2002 | 0.000192 | 0.000192 | 0.000192 |
| 2003 | 2e-04 | 0.000199 | 2e-04 |
| 2004 | 0.000209 | 0.000208 | 0.000209 |
| 2005 | 0.000221 | 0.000221 | 0.000222 |
| 2006 | 0.000225 | 0.000225 | 0.000225 |
| 2007 | 0.000218 | 0.000217 | 0.000218 |
| 2008 | 0.000205 | 0.000205 | 0.000206 |
| 2009 | 0.000195 | 0.000195 | 0.000195 |
| 2010 | 0.000192 | 0.000192 | 0.000192 |
| 2011 | 0.000185 | 0.000184 | 0.000185 |
| 2012 | 0.000168 | 0.000168 | 0.000168 |
| 2013 | 0.000151 | 0.000151 | 0.000152 |
| 2014 | 0.00014 | 0.00014 | 0.00014 |
| 2015 | 0.000128 | 0.000128 | 0.000129 |
| 2016 | 0.00012 | 0.000119 | 0.00012 |
| 2017 | 0.000114 | 0.000113 | 0.000114 |
| 2018 | 0.000108 | 0.000108 | 0.000108 |
| 2019 | 0.000105 | 0.000105 | 0.000105 |
| 2020 | 0.000102 | 0.000102 | 0.000102 |
| 2021 | 0.000103 | 0.000103 | 0.000103 |
| 2022 | 9.7e-05 | 8.9e-05 | 0.000105 |
| 2023 | 9.4e-05 | 8.1e-05 | 0.000107 |
| 2024 | 9.1e-05 | 7.3e-05 | 0.00011 |
| 2025 | 8.9e-05 | 6.3e-05 | 0.000115 |
| 2026 | 8.7e-05 | 5.4e-05 | 0.000119 |
| 2027 | 8.4e-05 | 4.4e-05 | 0.000124 |
| 2028 | 8.2e-05 | 3.4e-05 | 0.000129 |
| 2029 | 7.9e-05 | 2.4e-05 | 0.000135 |
| 2030 | 7.7e-05 | 1.4e-05 | 0.00014 |
| **Lymphaticfilariasis** |  |  |  |
| 1990 | 0.000791 | 0.00079 | 0.000792 |
| 1991 | 0.000781 | 0.000781 | 0.000782 |
| 1992 | 0.000772 | 0.000771 | 0.000773 |
| 1993 | 0.000764 | 0.000763 | 0.000765 |
| 1994 | 0.000758 | 0.000757 | 0.000758 |
| 1995 | 0.000753 | 0.000752 | 0.000754 |
| 1996 | 0.000749 | 0.000748 | 0.000749 |
| 1997 | 0.000741 | 0.000741 | 0.000742 |
| 1998 | 0.000732 | 0.000732 | 0.000733 |
| 1999 | 0.000722 | 0.000722 | 0.000723 |
| 2000 | 0.000713 | 0.000712 | 0.000714 |
| 2001 | 0.000691 | 0.00069 | 0.000692 |
| 2002 | 0.000651 | 0.00065 | 0.000651 |
| 2003 | 0.000601 | 0.000601 | 0.000602 |
| 2004 | 0.000554 | 0.000554 | 0.000555 |
| 2005 | 0.000521 | 0.00052 | 0.000521 |
| 2006 | 0.000482 | 0.000481 | 0.000482 |
| 2007 | 0.000422 | 0.000421 | 0.000422 |
| 2008 | 0.000355 | 0.000355 | 0.000356 |
| 2009 | 0.000298 | 0.000298 | 0.000298 |
| 2010 | 0.000267 | 0.000267 | 0.000267 |
| 2011 | 0.00026 | 0.00026 | 0.00026 |
| 2012 | 0.000261 | 0.000261 | 0.000261 |
| 2013 | 0.000266 | 0.000266 | 0.000267 |
| 2014 | 0.000272 | 0.000272 | 0.000273 |
| 2015 | 0.000274 | 0.000274 | 0.000275 |
| 2016 | 0.000259 | 0.000259 | 0.00026 |
| 2017 | 0.000227 | 0.000227 | 0.000228 |
| 2018 | 0.000193 | 0.000193 | 0.000194 |
| 2019 | 0.000175 | 0.000174 | 0.000175 |
| 2020 | 0.00017 | 0.000169 | 0.00017 |
| 2021 | 0.000165 | 0.000165 | 0.000165 |
| 2022 | 0.000165 | 0.00015 | 0.00018 |
| 2023 | 0.000163 | 0.000134 | 0.000192 |
| 2024 | 0.000161 | 0.000115 | 0.000206 |
| 2025 | 0.00016 | 9.5e-05 | 0.000225 |
| 2026 | 0.000159 | 7.3e-05 | 0.000246 |
| 2027 | 0.00016 | 4.9e-05 | 0.00027 |
| 2028 | 0.000161 | 2.4e-05 | 0.000298 |
| 2029 | 0.000162 | -4.00E-06 | 0.000329 |
| 2030 | 0.000165 | -3.40E-05 | 0.000364 |
| **Onchocerciasis** |  |  |  |
| 1990 | 0.000267 | 0.000266 | 0.000267 |
| 1991 | 0.000253 | 0.000252 | 0.000253 |
| 1992 | 0.00024 | 0.00024 | 0.000241 |
| 1993 | 0.00023 | 0.000229 | 0.00023 |
| 1994 | 0.000221 | 0.00022 | 0.000221 |
| 1995 | 0.000214 | 0.000214 | 0.000214 |
| 1996 | 0.000208 | 0.000208 | 0.000209 |
| 1997 | 0.000203 | 0.000202 | 0.000203 |
| 1998 | 0.000198 | 0.000197 | 0.000198 |
| 1999 | 0.000193 | 0.000193 | 0.000194 |
| 2000 | 0.000191 | 0.000191 | 0.000192 |
| 2001 | 0.000191 | 0.000191 | 0.000191 |
| 2002 | 0.000192 | 0.000192 | 0.000192 |
| 2003 | 0.000194 | 0.000193 | 0.000194 |
| 2004 | 0.000195 | 0.000195 | 0.000196 |
| 2005 | 0.000196 | 0.000195 | 0.000196 |
| 2006 | 0.000192 | 0.000192 | 0.000192 |
| 2007 | 0.000182 | 0.000182 | 0.000183 |
| 2008 | 0.000171 | 0.00017 | 0.000171 |
| 2009 | 0.00016 | 0.00016 | 0.000161 |
| 2010 | 0.000156 | 0.000156 | 0.000156 |
| 2011 | 0.000157 | 0.000156 | 0.000157 |
| 2012 | 0.000158 | 0.000158 | 0.000159 |
| 2013 | 0.00016 | 0.00016 | 0.00016 |
| 2014 | 0.000162 | 0.000162 | 0.000162 |
| 2015 | 0.000163 | 0.000163 | 0.000163 |
| 2016 | 0.000163 | 0.000163 | 0.000163 |
| 2017 | 0.000161 | 0.000161 | 0.000162 |
| 2018 | 0.00016 | 0.000159 | 0.00016 |
| 2019 | 0.000159 | 0.000158 | 0.000159 |
| 2020 | 0.000158 | 0.000158 | 0.000158 |
| 2021 | 0.000158 | 0.000157 | 0.000158 |
| 2022 | 0.000154 | 0.000148 | 0.000161 |
| 2023 | 0.000153 | 0.000141 | 0.000164 |
| 2024 | 0.000151 | 0.000134 | 0.000169 |
| 2025 | 0.00015 | 0.000125 | 0.000174 |
| 2026 | 0.000148 | 0.000116 | 0.00018 |
| 2027 | 0.000146 | 0.000106 | 0.000186 |
| 2028 | 0.000145 | 9.6e-05 | 0.000193 |
| 2029 | 0.000143 | 8.5e-05 | 0.000201 |
| 2030 | 0.000141 | 7.4e-05 | 0.000208 |
| **Dengue** |  |  |  |
| 1990 | 0.000217 | 0.000216 | 0.000217 |
| 1991 | 0.000222 | 0.000222 | 0.000223 |
| 1992 | 0.000221 | 0.00022 | 0.000221 |
| 1993 | 0.000223 | 0.000223 | 0.000224 |
| 1994 | 0.000225 | 0.000225 | 0.000225 |
| 1995 | 0.000228 | 0.000228 | 0.000229 |
| 1996 | 0.000237 | 0.000236 | 0.000237 |
| 1997 | 0.000233 | 0.000233 | 0.000234 |
| 1998 | 0.000256 | 0.000255 | 0.000256 |
| 1999 | 0.000238 | 0.000237 | 0.000238 |
| 2000 | 0.000239 | 0.000239 | 0.00024 |
| 2001 | 0.000243 | 0.000242 | 0.000243 |
| 2002 | 0.000246 | 0.000246 | 0.000247 |
| 2003 | 0.000251 | 0.00025 | 0.000251 |
| 2004 | 0.000264 | 0.000264 | 0.000265 |
| 2005 | 0.000263 | 0.000263 | 0.000264 |
| 2006 | 0.000269 | 0.000269 | 0.00027 |
| 2007 | 0.000284 | 0.000284 | 0.000285 |
| 2008 | 0.000286 | 0.000286 | 0.000287 |
| 2009 | 0.000299 | 0.000299 | 3e-04 |
| 2010 | 0.00031 | 0.00031 | 0.000311 |
| 2011 | 0.00031 | 0.00031 | 0.000311 |
| 2012 | 0.00032 | 0.00032 | 0.000321 |
| 2013 | 0.000324 | 0.000324 | 0.000325 |
| 2014 | 0.000325 | 0.000325 | 0.000326 |
| 2015 | 0.000324 | 0.000324 | 0.000324 |
| 2016 | 0.000314 | 0.000314 | 0.000315 |
| 2017 | 0.000322 | 0.000322 | 0.000323 |
| 2018 | 0.000299 | 0.000299 | 0.000299 |
| 2019 | 0.000302 | 0.000301 | 0.000302 |
| 2020 | 0.000282 | 0.000282 | 0.000282 |
| 2021 | 0.000276 | 0.000276 | 0.000277 |
| 2022 | 0.000266 | 0.000251 | 0.000281 |
| 2023 | 0.000258 | 0.000236 | 0.000281 |
| 2024 | 0.000251 | 0.00022 | 0.000282 |
| 2025 | 0.000244 | 0.000202 | 0.000285 |
| 2026 | 0.000237 | 0.000184 | 0.000289 |
| 2027 | 0.00023 | 0.000165 | 0.000294 |
| 2028 | 0.000223 | 0.000147 | 0.000299 |
| 2029 | 0.000216 | 0.000128 | 0.000304 |
| 2030 | 0.00021 | 0.00011 | 0.00031 |
| **Yellow fever** |  |  |  |
| 1990 | 0.000171 | 0.00017 | 0.000171 |
| 1991 | 0.000147 | 0.000146 | 0.000147 |
| 1992 | 0.000129 | 0.000129 | 0.000129 |
| 1993 | 0.000129 | 0.000129 | 0.000129 |
| 1994 | 0.000126 | 0.000126 | 0.000127 |
| 1995 | 0.000135 | 0.000134 | 0.000135 |
| 1996 | 0.000121 | 0.00012 | 0.000121 |
| 1997 | 0.000112 | 0.000112 | 0.000112 |
| 1998 | 0.00011 | 0.00011 | 0.000111 |
| 1999 | 0.000106 | 0.000106 | 0.000106 |
| 2000 | 0.00011 | 0.00011 | 0.000111 |
| 2001 | 0.000106 | 0.000106 | 0.000106 |
| 2002 | 1e-04 | 1e-04 | 0.000101 |
| 2003 | 1e-04 | 1e-04 | 0.000101 |
| 2004 | 0.000102 | 0.000102 | 0.000102 |
| 2005 | 9.1e-05 | 9.1e-05 | 9.1e-05 |
| 2006 | 8.6e-05 | 8.6e-05 | 8.6e-05 |
| 2007 | 8.2e-05 | 8.2e-05 | 8.3e-05 |
| 2008 | 7.9e-05 | 7.8e-05 | 7.9e-05 |
| 2009 | 7.5e-05 | 7.4e-05 | 7.5e-05 |
| 2010 | 7.5e-05 | 7.5e-05 | 7.5e-05 |
| 2011 | 8.9e-05 | 8.9e-05 | 9e-05 |
| 2012 | 6.6e-05 | 6.6e-05 | 6.6e-05 |
| 2013 | 6.3e-05 | 6.3e-05 | 6.3e-05 |
| 2014 | 6e-05 | 6e-05 | 6e-05 |
| 2015 | 5.7e-05 | 5.6e-05 | 5.7e-05 |
| 2016 | 6.5e-05 | 6.5e-05 | 6.5e-05 |
| 2017 | 5.5e-05 | 5.5e-05 | 5.5e-05 |
| 2018 | 5.3e-05 | 5.3e-05 | 5.3e-05 |
| 2019 | 4.6e-05 | 4.6e-05 | 4.6e-05 |
| 2020 | 4.4e-05 | 4.4e-05 | 4.4e-05 |
| 2021 | 4.3e-05 | 4.3e-05 | 4.3e-05 |
| 2022 | 4.2e-05 | 3e-05 | 5.4e-05 |
| 2023 | 4.2e-05 | 1.5e-05 | 6.9e-05 |
| 2024 | 4.1e-05 | -3.00E-06 | 8.6e-05 |
| 2025 | 4.1e-05 | -2.40E-05 | 0.000105 |
| 2026 | 4e-05 | -4.60E-05 | 0.000126 |
| 2027 | 3.9e-05 | -6.90E-05 | 0.000148 |
| 2028 | 3.9e-05 | -9.40E-05 | 0.000172 |
| 2029 | 3.8e-05 | -0.00012 | 0.000197 |
| 2030 | 3.8e-05 | -0.000147 | 0.000223 |
| **Zika virus** |  |  |  |
| 1990 | 0 | 0 | 0 |
| 1991 | 0 | 0 | 0 |
| 1992 | 0 | 0 | 0 |
| 1993 | 0 | 0 | 0 |
| 1994 | 0 | 0 | 0 |
| 1995 | 0 | 0 | 0 |
| 1996 | 0 | 0 | 0 |
| 1997 | 0 | 0 | 0 |
| 1998 | 0 | 0 | 0 |
| 1999 | 0 | 0 | 0 |
| 2000 | 0 | 0 | 0 |
| 2001 | 0 | 0 | 0 |
| 2002 | 0 | 0 | 0 |
| 2003 | 0 | 0 | 0 |
| 2004 | 0 | 0 | 0 |
| 2005 | 0 | 0 | 0 |
| 2006 | 0 | 0 | 0 |
| 2007 | 0 | 0 | 0 |
| 2008 | 0 | 0 | 0 |
| 2009 | 0 | 0 | 0 |
| 2010 | 0 | 0 | 0 |
| 2011 | 0 | 0 | 0 |
| 2012 | 0 | 0 | 0 |
| 2013 | 0 | 0 | 0 |
| 2014 | 0 | 0 | 0 |
| 2015 | 0 | 0 | 0 |
| 2016 | 1e-06 | 1e-06 | 1e-06 |
| 2017 | 0 | 0 | 0 |
| 2018 | 0 | 0 | 0 |
| 2019 | 0 | 0 | 0 |
| 2020 | 0 | 0 | 0 |
| 2021 | 0 | 0 | 0 |
| 2022 | 0 | 0 | 0 |
| 2023 | 0 | 0 | 0 |
| 2024 | 0 | 0 | 0 |
| 2025 | 0 | 0 | 0 |
| 2026 | 0 | 0 | 0 |
| 2027 | 0 | 0 | 0 |
| 2028 | 0 | 0 | 0 |
| 2029 | 0 | 0 | 0 |
| 2030 | 0 | 0 | 0 |

CI credible intervals














**Fig A** Global and five SDI regions’ changes in ASPRs per 100,000 population for arthropod-borne diseases from 1990 to 2021. ASPR age-standardized prevalence rates, APC annual percentage change, AAPC average annual percentage change, SDI Socio-demographic Index, **P* < 0.05. No data available for Zika virus






















**Fig B** Global and five SDI regions’ changes in ASDRs per 100,000 population for arthropod-borne diseases from 1990 to 2021. ASDR age-standardized DALYs rates, APC annual percentage change, AAPC average annual percentage change, SDI Socio-demographic Index, **P* < 0.05. No data available for Zika virus


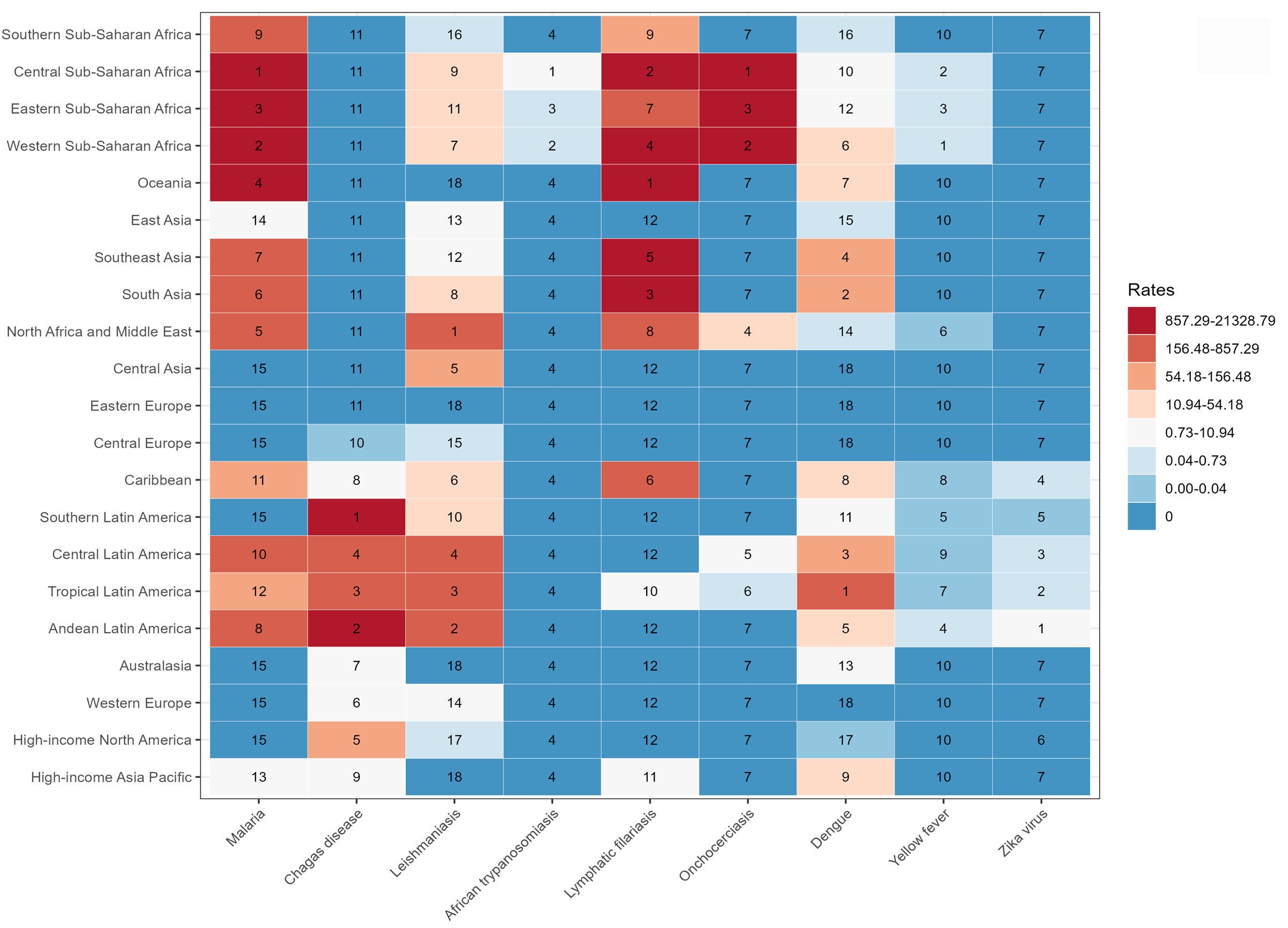


**Fig C** Ranking of ASPRs for Arthropod-Borne Diseases by 21 Regions. ASPRs age-standardized prevalence rates


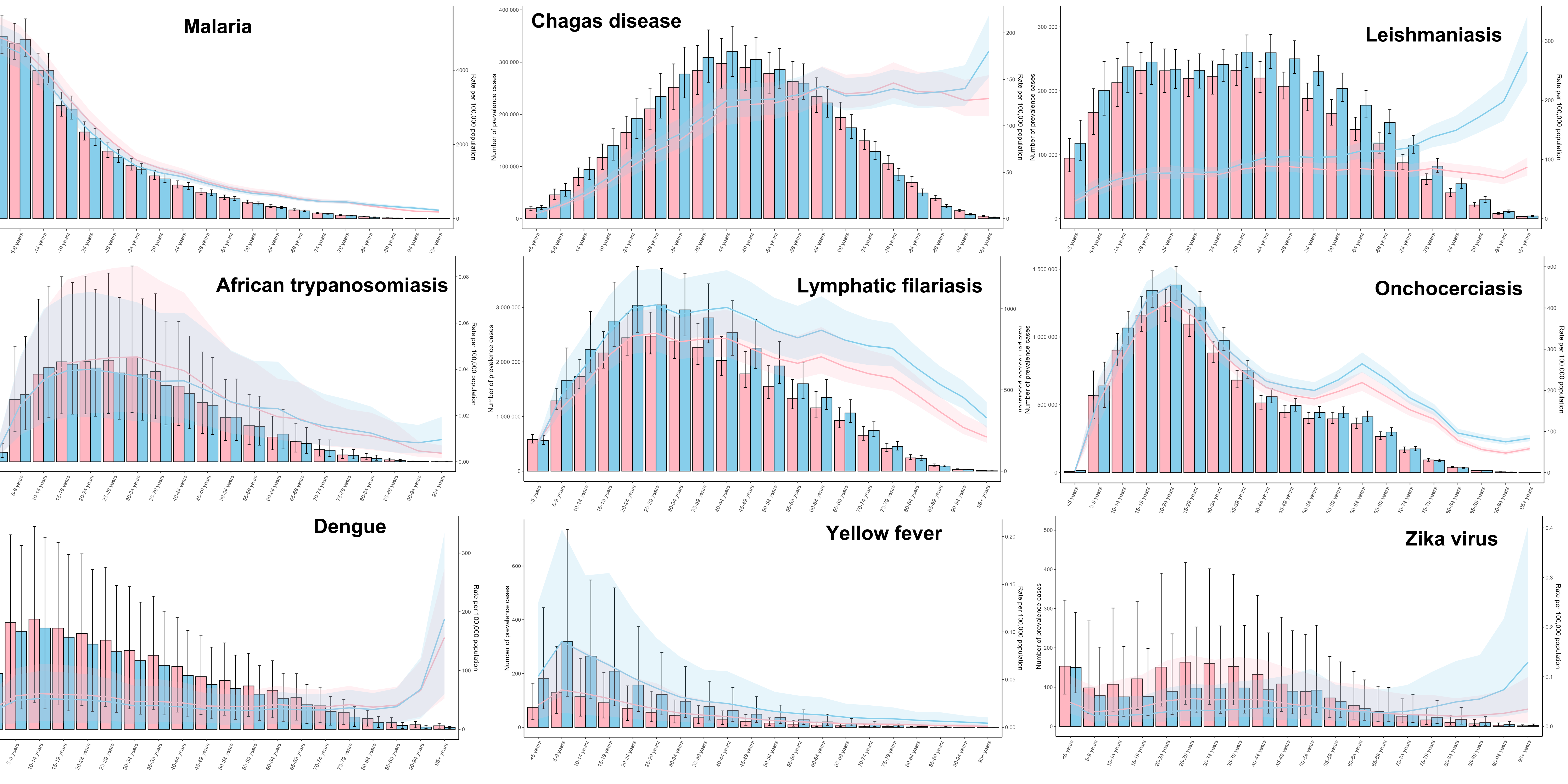


**Fig D** Number of prevalence and ASPRs of nine arthropod-borne diseases across different age groups globally for both sexes in 2021. ASPRs age-standardized prevalence rates


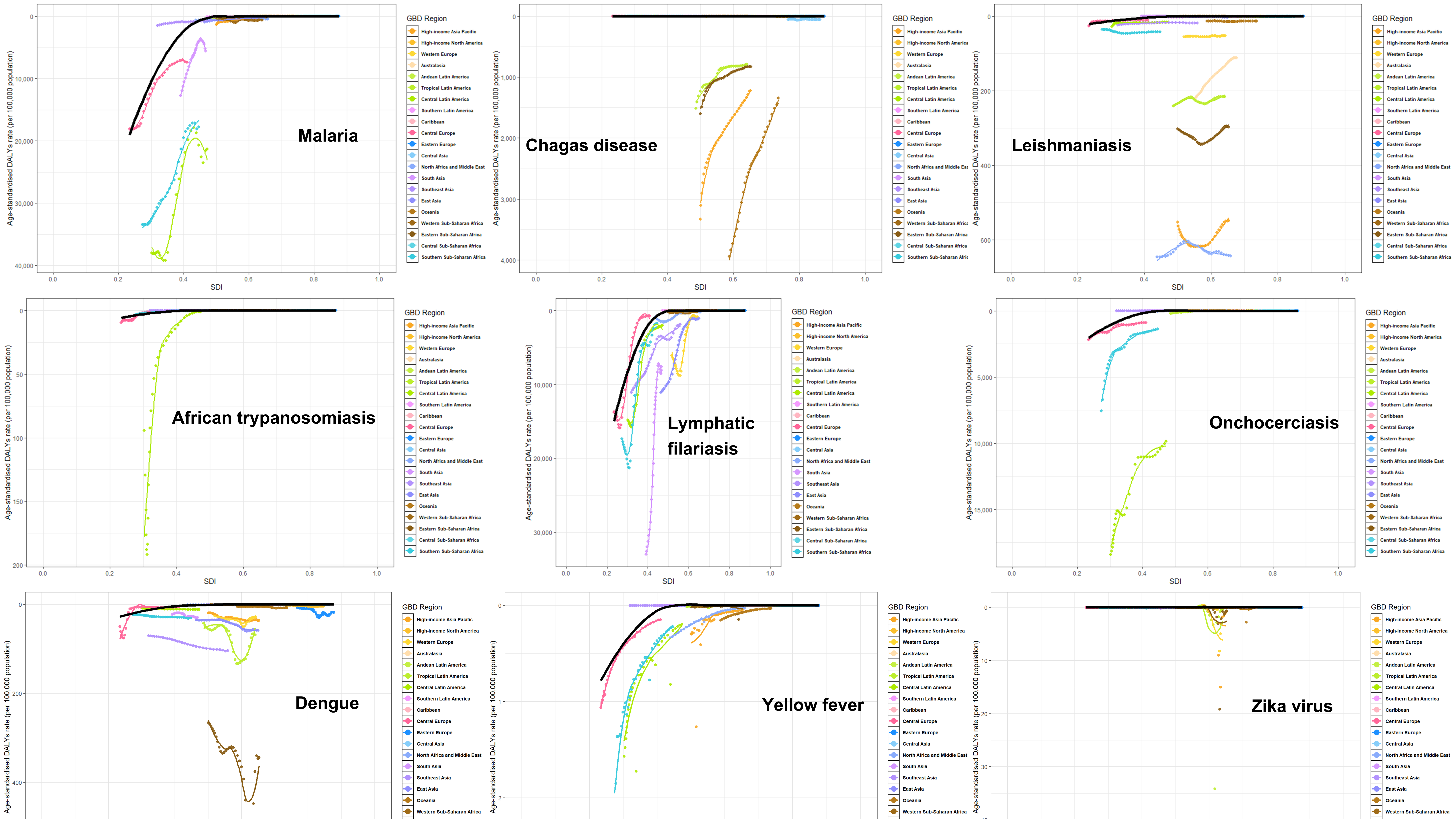


**Fig E** Frontier Analysis of ASDRs of Arthropod-Borne Diseases Across 21 Regions in Relation to SDI. ASPRs age-standardized prevalence rates, SDI Socio-demographic Index





**Fig F** Predicted ASPRs of nine arthropod-borne diseases worldwide from 2022 to 2030. ASPRs age-standardized prevalence rates
